# Evolutionary trajectories of small cell lung cancer under therapy

**DOI:** 10.1038/s41586-024-07177-7

**Published:** 2024-03-13

**Authors:** Julie George, Lukas Maas, Nima Abedpour, Maria Cartolano, Laura Kaiser, Rieke N. Fischer, Andreas H. Scheel, Jan-Philipp Weber, Martin Hellmich, Graziella Bosco, Caroline Volz, Christian Mueller, Ilona Dahmen, Felix John, Cleidson Padua Alves, Lisa Werr, Jens Peter Panse, Martin Kirschner, Walburga Engel-Riedel, Jessica Jürgens, Erich Stoelben, Michael Brockmann, Stefan Grau, Martin Sebastian, Jan A. Stratmann, Jens Kern, Horst-Dieter Hummel, Balazs Hegedüs, Martin Schuler, Till Plönes, Clemens Aigner, Thomas Elter, Karin Toepelt, Yon-Dschun Ko, Sylke Kurz, Christian Grohé, Monika Serke, Katja Höpker, Lars Hagmeyer, Fabian Doerr, Khosro Hekmath, Judith Strapatsas, Karl-Otto Kambartel, Geothy Chakupurakal, Annette Busch, Franz-Georg Bauernfeind, Frank Griesinger, Anne Luers, Wiebke Dirks, Rainer Wiewrodt, Andrea Luecke, Ernst Rodermann, Andreas Diel, Volker Hagen, Kai Severin, Roland T. Ullrich, Hans Christian Reinhardt, Alexander Quaas, Magdalena Bogus, Cornelius Courts, Peter Nürnberg, Kerstin Becker, Viktor Achter, Reinhard Büttner, Jürgen Wolf, Martin Peifer, Roman K. Thomas

**Affiliations:** 1 Department of Translational Genomics, Faculty of Medicine and University Hospital Cologne, University of Cologne, Cologne, Germany; 2 Department of Otorhinolaryngology, Head and Neck Surgery, Faculty of Medicine and University Hospital Cologne, University Hospital of Cologne, Cologne, Germany; 3 Department I of Internal Medicine, Centre for Integrated Oncology Aachen Bonn Cologne Duesseldorf, University Hospital Cologne, Cologne, Germany; 4 Cancer Research Centre Cologne Essen, Faculty of Medicine and University Hospital Cologne, University of Cologne, Cologne, Germany; 5 Centre for Molecular Medicine, University of Cologne, Cologne, Germany; 6 Department I of Internal Medicine, Lung Cancer Group Cologne, University Hospital Cologne, Cologne, Germany; 7 Institute of Pathology, Medical Faculty, University Hospital Cologne, University of Cologne, Cologne, Germany; 8 Institute of Medical Statistics, and Computational Biology, Faculty of Medicine and University Hospital Cologne, University of Cologne, Cologne, Germany; 9 Department of Haematology, Oncology, Haemostaseology and Stem Cell Transplantation, University Hospital RWTH Aachen, Aachen, Germany; 10 Centre for Integrated Oncology, Aachen Bonn Cologne Düsseldorf, Aachen, Germany; 11 Department of Pneumology, City of Cologne Municipal Hospitals, Lung Hospital Cologne Merheim, Cologne, Germany; 12 Thoraxclinic Cologne, Thoracic Surgery, St. Hildegardis-Krankenhaus, Cologne, Germany; 13 Department of Pathology, City of Cologne Municipal Hospitals, Witten/Herdecke University, Cologne, Germany; 14 Department of General Neurosurgery, Centre of Neurosurgery, University Hospital Cologne, Cologne, Germany; 15 University Medicine Marburg – Campus Fulda, Department of Neurosurgery, Fulda, Germany; 16 Department of Medicine II, Haematology/Oncology, University Hospital Frankfurt, Goethe University, Frankfurt, Germany; 17 Frankfurt Cancer Institute, Goethe University Frankfurt, Frankfurt, Germany; 18 DKFZ, German Cancer Research Centre, German Cancer Consortium, Heidelberg, Germany; 19 Klinikum Würzburg Mitte – Missioklinik site, Pneumology and Respiratory Medicine, Würzburg, Germany; 20 Translational Oncology/Early Clinical Trial Unit, Comprehensive Cancer Centre Mainfranken, University Hospital Wuerzburg, Wuerzburg, Germany; 21 Department of Thoracic Surgery, University Medicine Essen – Ruhrlandklinik, University Duisburg-Essen, Essen, Germany; 22 Department of Medical Oncology, West German Cancer Centre Essen, University Duisburg-Essen, Essen, Germany; 23 Division of Thoracic Surgery, Department of General, Thoracic and Vascular Surgery, University Hospital Carl Gustav Carus, Dresden, Germany; 24 Department of Thoracic Surgery, Medical University of Vienna, Vienna General Hospital, Vienna, Austria; 25 Comprehensive Cancer Centre CIO Bonn, Bonn, Germany; 26 Department of Respiratory Diseases, Evangelische Lungenklinik, Berlin, Germany; 27 DGD Lungenklinik Hemer, Internal Medicine, Pneumology and Oncology, Hemer, Germany; 28 Clinic III for Internal Medicine, Faculty of Medicine and University Hospital Cologne, University of Cologne, Cologne, Germany; 29 Clinic of Pneumology and Allergology, Centre for Sleep Medicine and Respiratory Care, Bethanien Hospital Solingen, Solingen, Germany; 30 Department of Cardiothoracic Surgery, University Hospital of Cologne, Cologne, Germany; 31 Department of Haematology, Oncology and Clinical Immunology, University Hospital of Duesseldorf, Düsseldorf, Germany; 32 Krankenhaus Bethanien Moers, Moers, Germany; 33 Praxis für Hämatologie und Onkologie, Koblenz, Germany; 34 Medical Clinic III for Oncology, Haematology, Immune-Oncology and Rheumatology, Centre for Integrative Medicine, University Hospital Bonn, Bonn, Germany; 35 Pius-Hospital Oldenburg, Department of Haematology and Oncology, University Department Internal Medicine-Oncology, University Medicine Oldenburg, Oldenburg, Germany; 36 Pulmonary Division, Department of Medicine A, Münster University Hospital, Münster, Germany; 37 Onkologie Rheinsieg, Praxisnetzwerk Hämatologie und Internistische Onkologie, Troisdorf, Germany; 38 Clinic II for Internal Medicine, St.-Johannes-Hospital Dortmund, Dortmund, Germany; 39 Haematologie und Onkologie Köln MV-Zentrum, Cologne, Germany; 40 Department of Haematology and Stem Cell Transplantation, University Hospital Essen, Essen, Germany; 41 West German Cancer Centre, University Hospital Essen, Essen, Germany; 42 Institute of Legal Medicine, University of Cologne, Cologne, Germany; 43 Cologne Centre for Genomics, West German Genome Centre, University of Cologne, Cologne, Germany; 44 Computing Centre, University of Cologne, Cologne, Germany

**Keywords:** Tumour heterogeneity, Small-cell lung cancer, Cancer genomics, Chemotherapy, Cancer therapeutic resistance

## Abstract

The evolutionary processes that underlie the marked sensitivity of small cell lung cancer (SCLC) to chemotherapy and rapid relapse are unknown^1–3^. Here we determined tumour phylogenies at diagnosis and throughout chemotherapy and immunotherapy by multiregion sequencing of 160 tumours from 65 patients. Treatment-naive SCLC exhibited clonal homogeneity at distinct tumour sites, whereas first-line platinum-based chemotherapy led to a burst in genomic intratumour heterogeneity and spatial clonal diversity. We observed branched evolution and a shift to ancestral clones underlying tumour relapse. Effective radio- or immunotherapy induced a re-expansion of founder clones with acquired genomic damage from first-line chemotherapy. Whereas *TP53* and *RB1* alterations were exclusively part of the common ancestor, *MYC* family amplifications were frequently not constituents of the founder clone. At relapse, emerging subclonal mutations affected key genes associated with SCLC biology, and tumours harbouring clonal *CREBBP*/*EP300* alterations underwent genome duplications. Gene-damaging *TP53* alterations and co-alterations of *TP53* missense mutations with *TP73*, *CREBBP*/*EP300* or *FMN2* were significantly associated with shorter disease relapse following chemotherapy. In summary, we uncover key processes of the genomic evolution of SCLC under therapy, identify the common ancestor as the source of clonal diversity at relapse and show central genomic patterns associated with sensitivity and resistance to chemotherapy.

## Abstract (web summary)

We uncover key processes of the genomic evolution of small cell lung cancer under therapy, identify the common ancestor as the source of clonal diversity at relapse and show central genomic patterns associated with drug response.

## Main

Small cell lung cancer (SCLC) is one of the deadliest human cancers, with a 5 year survival rate of less than 7%^1–4^. The standard of care for extensive-stage SCLC consists of systemic treatment with platinum and etoposide, recently combined with programmed death-ligand 1 (PD-L1) immune checkpoint inhibitors (ICIs)^2^. One peculiarity of SCLC is its typically high sensitivity to platinum-based chemotherapy followed by rapid recurrence, which distinguishes it from most other human cancers. Unfortunately, second-line treatment with other chemotherapeutics or immunotherapy is only marginally effective and patients ultimately succumb to their disease^1,2,4^.

We and others have previously performed large-scale genome sequencing to comprehensively characterize cancer genome alterations in SCLC, which showed universal biallelic losses of the tumour suppressors *TP53* and *RB1*, additional alterations to histone-modifying enzymes and cell cycle regulators, and *MYC* transcription factor amplifications^5–7^. Furthermore, SCLC subgroups were defined on the basis of the expression of neuroendocrine lineage transcription factors, which impact tumour biology and treatment outcome^4,8,9^. Finally, preliminary studies have provided initial clues in regard to molecular pathways associated with resistance to chemotherapy^10,11^.

Despite progress in characterization of the molecular basis of SCLC, the underlying patterns of clonal evolution and the mechanisms causing drug resistance have remained unclear. We suggest that cancer genome alterations not only drive malignant transformation in SCLC but also influence the clinical phenotypes of chemotherapy sensitivity, tumour progression and relapse. We therefore performed comprehensive multiregional and longitudinal studies of tumours obtained from 65 patients to decipher the evolutionary and genomic principles that govern response and resistance to therapy in SCLC.

## Tumour specimens and clinical data

**Fig. 1:**
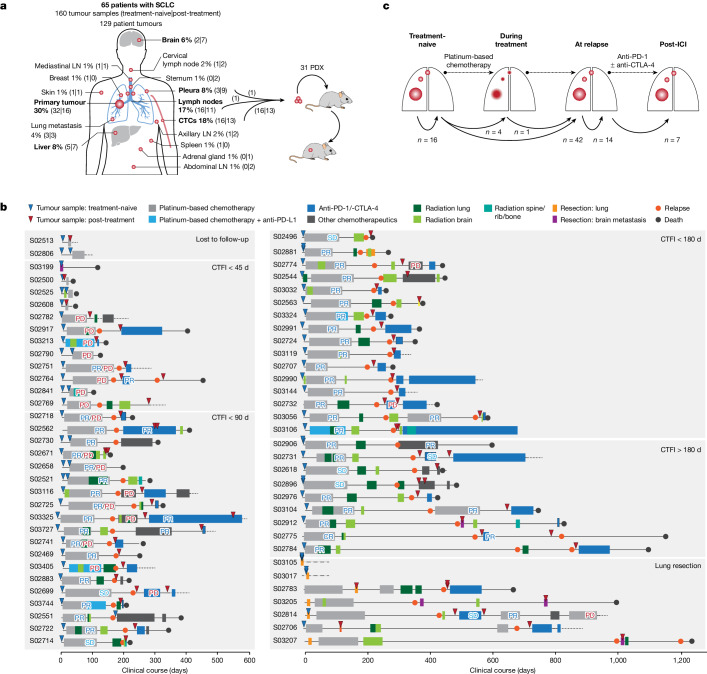
Tumour samples and clinical history of 65 patients with SCLC. **a**, Tumour sites sampled from 65 patients with SCLC. Frequently sampled sites are highlighted in bold. Tumours were acquired either at the time of first diagnosis (treatment-naive) or following initiation of treatment (post-treatment). Tumour samples analysed as patient-derived xenotransplant (PDX) models are indicated. **b**, Schematic overview of the clinical course of 65 patients with SCLC. Patients were ordered according to their duration of response to first-line platinum-based chemotherapy, referring to a CTFI of 45, 90 and 180 days (National Comprehensive Cancer Network (NCCN) guidelines). Patients who, following initiation of first-line treatment, were either lost to follow-up or underwent surgical resection of the primary tumour were sorted to separate panels. The treatment administered to each patient is annotated and the clinical response is described as either complete response (CR), partial response (PR), stable disease (SD), progressive disease (PD) or mixed response (PR/PD). A detailed description of all clinical characteristics is provided in Supplementary Table 1 and Methods. **c**, Schematic overview showing the analysis of paired, patient-matched tumour sites: paired studies of spatially distinct tumours at the time of first diagnosis (treatment-naive, *n* = 16); paired studies of tumour sites pretreatment and during treatment (*n* = 5) or at clinical relapse following completion of first-line platinum-based chemotherapy (*n* = 42); paired analyses of spatially distinct tumour sites at relapse (*n* = 14); and analyses of tumours acquired before and after subsequent lines of treatment with ICIs (*n* = 7). The scheme shows tumour sites in the lung, referring to primary and metastatic sites (larger and smaller red circles, respectively). LN, lymph node.

We collected 160 tumour specimens from 65 patients with SCLC under institutional review board approval and performed whole-exome, genome and transcriptome sequencing of samples with an average tumour purity of 85% (Fig. 1a–c and Supplementary Tables 1–3). We most frequently sampled the primary lung tumour, pulmonary lymph nodes, liver, pleura and brain metastases. Furthermore, patient-derived xenotransplants were established from fine-needle biopsies or circulating tumour cells (CTCs), which have been previously shown to recapitulate the genomic profiles of patients’ tumours^12,13^ (Fig. 1a and Methods). The histology of SCLC was confirmed in all cases; additional components of adenocarcinoma or large-cell neuroendocrine carcinoma (LCNEC) were identified in three patients (Supplementary Table 1). The clinical history was typical of SCLC and the majority of patients had received first-line treatment with platinum-based chemotherapy, achieving a median relapse-free interval of 88 days (Fig. 1b, Extended Data Table 1 and Supplementary Table 1). In line with clinical guidelines^14^ we grouped patients according to their duration of response to first-line chemotherapy, referring to the chemotherapy-free interval (CTFI) of 45, 90 and 180 days (Fig. 1b). At relapse, 80% of these patients (*n* = 44 of 55) received additional lines of therapy, which included other chemotherapeutics or treatment with anti-PD-1 and/or anti-CTLA-4 ICIs (Supplementary Table 1).

We analysed at least two tumour samples per patient, obtained at either single or multiple time points throughout the course of treatment. For interpatient comparisons we focused on paired studies of tumours acquired under distinct scenarios throughout the clinical course of the patients: (1) spatially distinct tumour samples in the treatment-naive setting at the time of first diagnosis (*n* = 16); (2) temporally distinct tumours acquired at first diagnosis before initiation of therapy and either during first-line platinum-based chemotherapy (*n* = 5) or following completion of chemotherapy (*n* = 42); (3) spatially, but not temporally, separate tumours analysed solely at the time of relapse (*n* = 14); and (4) tumours obtained before and after subsequent lines of treatment with immunotherapy (*n* = 7) (Fig. 1c, Extended Data Table 1 and Methods).

## Tumour phylogenies of metastatic SCLC

Aiming to shed light on the dynamics of genome evolution in metastatic SCLC, we performed genome sequencing of all tumour specimens to identify genomic alterations. Whole-exome sequencing data at an average coverage of 127-fold were used to compute cancer cell fraction (CCF) for somatic mutations, a metric of relative abundance of mutant alleles corrected for purity, ploidy and absolute copy number, which affords the assignment of mutations to individual tumour clones and enables tracking of single clones in spatially and temporally distinct tumours^15^ (Methods). We assigned mutations to the most recent common ancestor (C0) if mutations were shared and clonal with CCFs of 100% across all samples analysed, and to subclones (C1, C2 and C3) if clusters of mutations were either private to specific tumour sites or found at lower CCF. We thus reconstructed the clonal lineage and determined tumour phylogenies for all 65 patients (Supplementary Appendix, Supplementary Tables 2 and 4 and Methods).

**Fig. 2:**
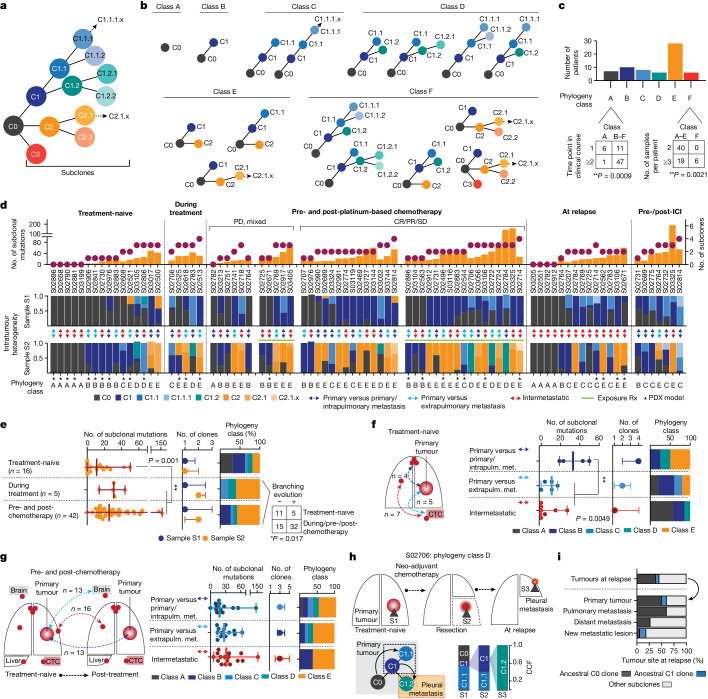
Tumour phylogenies and clonal dynamics in 65 patients with SCLC. **a**, Schematic of clone phylogeny depicting the most recent common ancestral clone, C0, descending C1, C2 and C3 and subsequent subclones numbered accordingly. **b**, Phylogeny classes: class A, no subclones; linear phylogenies with one subclone (class B) or at least two subclones (class C); phylogenies with one branching event from C1 subclones (class D) or the common ancestral clone C0 (class E), or at least two branching events (class F). **c**, Number of samples and distinct time points associated with phylogeny class for each patient (Fisher’s exact test, two-sided, **P < 0.01). **d**, Tumour phylogenies at distinct clinical scenorios determined for each patient from paired analyses of WES data (samples S1 and S2; Fig. 1c and Methods), sorted according to the number of clones and subclonal mutations (top), showing site-specific CCF of identified clones (intratumour heterogeneity, middle) and phylogeny class (bottom). Cases pre- and post-platinum-based chemotherapy are sorted according to clinical response and exposure of tumour sites to radiation (Rx, green line). Double-headed arrows represent comparisons of distinct samples from the primary tumour and either intrapulmonary metastases (dark blue) or extrapulmonary metastases (light blue), or within intermetastatic sites (red). Asterisks mark samples from PDX models. **e**–**g**, Subclonal mutations, tumour clones and phylogeny class (median with whiskers representing minimum and maximum values) under distinct clinical scenarios. **e**, Branching evolution (classes D and E). Fisher’s exact test, two-sided, *P < 0.05. **f**,**g**, Spatially distinct sites from treatment-naive setting (**f**) and pre-/post-first-line platinum-based chemotherapy (**g**). **P < 0.01; Mann–Whitney *U*-test, two-sided, not significant. **h**, Clonal dynamics of patient S02706 for tumours acquired before (S1), after neo-adjuvant chemotherapy (S2) and at relapse (S3). **i**, Proportion of ancestral C0 or C1 clones in relapsing tumours.

**Extended Data Fig. 1:**
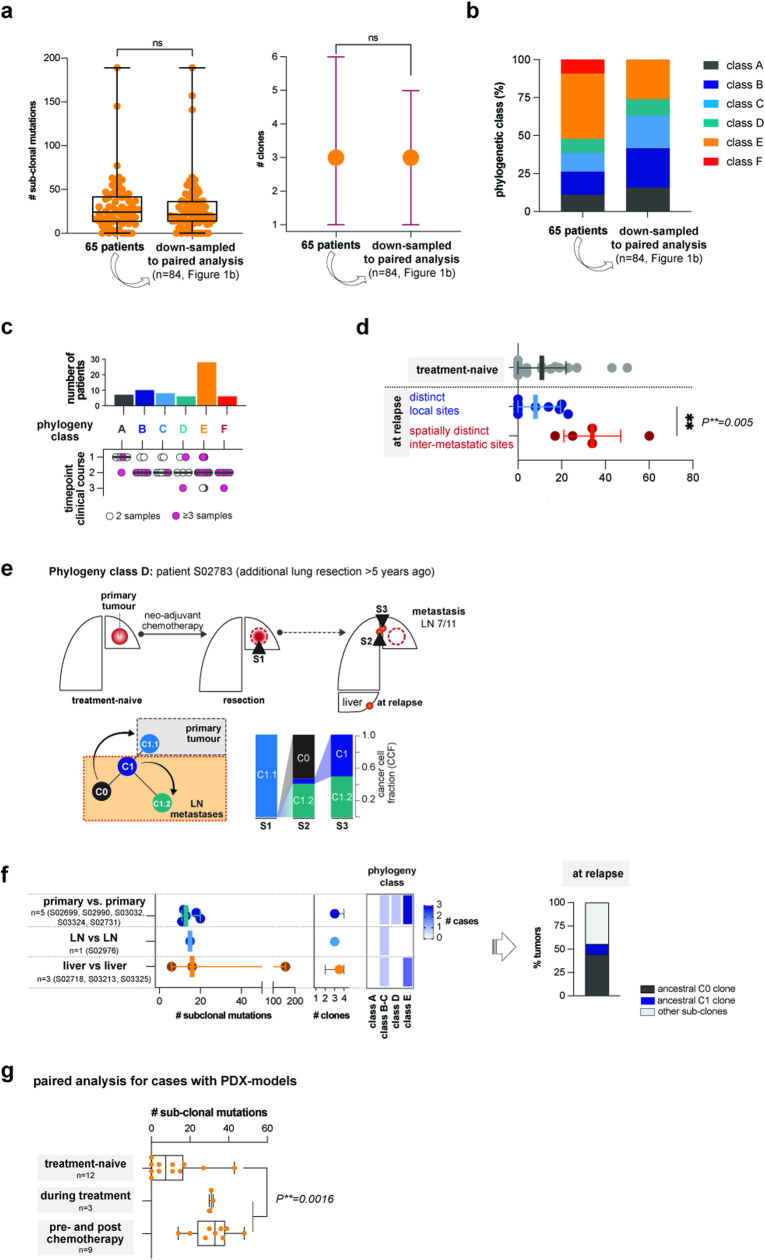
Clonality analysis on tumours from 65 patients with SCLC. **a**, Number of subclonal mutations (left panel, box plot displaying median with interquartile range) and number of subclones (right panel, displaying the median); whiskers indicate the range of minimum and maximum value. Mann-Whitney *U*-test, two-sided, at P < 0.05. NS, not significant. **b**, the assignment of phylogeny classes (**b**) for 65 patients with SCLC and for the analyses of n = 84 paired tumour analyses to permit interpatient comparisons for distinct the clinical scenarios described in Fig. 1b (Methods, Supplementary Table 4). **c**, Patient cases assigned to distinct classes of tumour phylogenies plotting the number of distinct timepoints and samples considered for the assignment. The median is indicated by grey lines. **d**, Level of subclonal mutations determined from multi-regional samples at relapse, focusing on paired analyses of distinct samples acquired from a given tumour site (n = 9 patients, dark blue) and for spatially distinct inter-metastatic sites (n = 5 patients, red). For comparisons, subclonal mutations determined from spatially distinct sites in treatment-naïve patients (n = 16) are plotted (grey). Data is presented as median with whiskers indicating the interquartile range. Mann-Whitney *U*-test, two-sided, P** < 0.01. **e**, Clonal dynamics in patient S02783 with phylogeny class D, tracking tumour clones at the site of the primary after neo-adjuvant chemotherapy (sample S1) and at relapse (samples S2 and S3). The clonal dynamics and the clonal composition are provided for all three samples. **f**, Level of subclonal diversity determined from the same tumour site (primary tumour, LN or liver metastases) sampled pre- and post-first-line chemotherapy referring to the number of subclonal mutations (left panel), the number of clones (middle panel), and to the assignment of phylogeny classes (right panel). Relapsing tumours revealing ancestral C0 and C1 tumour clones are plotted (figure at the right). Data is presented as median with whiskers indicating the interquartile range. **g**, Level of subclonality referring to the number of subclonal mutations assigned for distinct clinical settings. The data presented is a subset of the data presented in Fig. 2e, and refers only to cases for which the paired include samples from PDX models. Mann-Whitney *U*-test, two-sided, P** < 0.01;.

Previous genomic studies conducted for single tumour sites obtained from treatment-naive patients indicated low levels of genomic intratumour heterogeneity in SCLC compared with lung adenocarcinoma^5^. Through analysis of spatially and temporally distinct tumours, we now observed a wide range in the absolute number of subclonal mutations and subclones previously observed in other cancers as well^16^ (Extended Data Fig. 1a,b). Tumour phylogenies across all patients exhibited patterns of linear and branched evolution, in some cases indicating a sequential acquisition of genome alterations and thereby giving rise to a dominant clone. In other patients, emerging subclones branched from ancestral clones thus creating multiple lineages^16^. For systematic study of the evolutionary patterns we assigned tumour phylogenies to distinct classes (Fig. 2b,c): class A, if no subclones were identified, which was frequently observed when comparing more than one anatomic site at a single time point (Fisher’s exact test, **P < 0.01; Fig. 2c); classes B and C, with one or at least two subclones, respectively, compatible with linear phylogenies; classes D and E, phylogenies with one branching event in which tumour clones descend from either C1 subclones (class D) or the common ancestral clone C0 (class E); and class F, phylogenies with at least two branching events exclusively identified in patients, with higher numbers of specimens referring to at least three spatially or temporally distinct tumours (Fisher’s exact test, *P* < 0.001; Fig. 2c and Extended Data Fig. 1c), thus providing further information on phylogenetic complexity. To permit interpatient comparisons we therefore sought to perform paired analyses, considering a maximum of two samples per patient (Fig. 2d and Extended Data Table 1), which did not show any significant change in the absolute number of subclonal mutations but led to reduced phylogenetic complexities assigned to classes A–E (Methods and Extended Data Fig. 1a,b). We thus observed a significantly lower clonal diversity in treatment-naive patients across different tumour sites than in temporally and spatially distinct tumours from patients undergoing treatment (**P < 0.01; Fig. 2d,e). Consequently the genomic heterogeneity of the tumour—although limited at diagnosis—increased markedly as a result of therapeutic intervention.

We sought to determine the subclonal composition at the time of first diagnosis to study the evolutionary dynamics of tumour progression in a highly metastatic disease. Our analysis in these treatment-naive patients included spatially distinct intra- and extrapulmonary sites exhibiting either no evidence of subclones (class A) or limited mutational changes with patterns of linear evolution (Fig. 2d, left). Clonal diversity was lower when comparing metastatic sites with one another, which frequently included CTC-derived tumours and confirmed earlier observations^12,13^. However, tumour regions simultaneously obtained from the primary site and intrapulmonary metastases exhibited increased subclonal mutations (**P < 0.01) and branched evolutionary processes (classes D and E; Fig. 2e). Thus, following the successful establishment of metastases, the subclonal composition appeared largely unchanged. Additionally, increased clonal heterogeneity and ongoing evolution appeared to occur during the first steps of metastatic seeding in the physical proximity of the original founder clone, driving the outgrowth of one rapidly expanding tumour.

We next analysed the impact of chemotherapy on the dynamics of tumour evolution and compared tumours before therapy with tumour sites acquired during treatment and at the time of relapse. Most tumours exhibited clonal branching from ancestral clones C1 or C0 (67%, *n* = 31 of 46, *P* < 0.05) under therapy, causing increased site-specific intratumour heterogeneity (sample 2; Fig. 2d,e) and spatial clonal diversity when comparing specimens sampled simultaneously from different sites at relapse (**P < 0.01; Extended Data Fig. 1d). In two patients we tracked the evolutionary processes at the site of the primary tumour before and during therapy, following neo-adjuvant chemotherapy and at the time of subsequent relapse (Fig. 2h and Extended Data Fig. 1e). In both cases we found phylogenies of class D showing several distinct clones at the site of the primary tumour and repression of the initial dominating clone by chemotherapy, followed by the emergence and expansion of subclones descending from ancestral clone C1 that had caused relapse. Class D phylogenies were frequently identified in comparison with primary lung specimens (Fig. 2g; *n* = 6 of seven cases), again emphasizing that the site of the primary tumour serves as a source for ancestral clones that cause metastatic seeding and tumour recurrence. At relapse, both tumour sites exposed to treatment and newly formed metastatic lesions harboured a substantial fraction of pre-existing ancestral clones, most frequently the common ancestor C0 (*n* = 16 of 42, 38%), confirming its critical role in relapse (Fig. 2i). Because these branching events were frequently detected in comparisons from different sites, we next analysed repeated biopsies from the same site over time (*n* = 9) and found branching events and the presence of ancestral clones at relapse in these as well (Extended Data Fig. 1f). Furthermore, focusing the analysis on samples derived from xenotransplant models similarly showed a significant increase in subclonal mutations following treatment (Extended Data Fig. 1g), suggesting no bias with regard to sample type.

Our data thus suggest that neither is the observed level of evolutionary heterogeneity driven by different anatomic sites nor does first-line chemotherapy primarily drive linear evolution of tumour clones to the state of relapse. By contrast, our data support the view that one highly proliferating clone dominates the tumour at the time of first diagnosis, representing pseudo-clonality^16^ which is then suppressed and eliminated by therapy. At clinically overt recurrence, a multitude of subclones has emerged that are driven by the most recent common ancestor, which markedly increases spatial and intratumour heterogeneity.

## Mutation signatures of clonal diversity

**Fig. 3:**
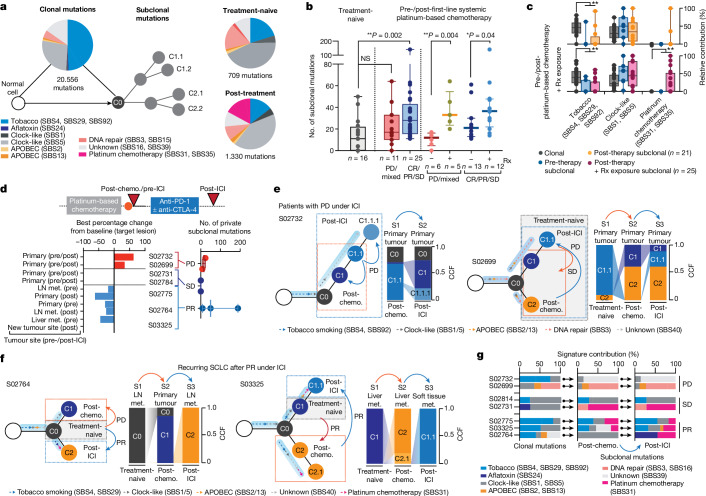
Mutation signatures of subclones. **a**, Mutational signatures of SBS assigned to clonal (ancestral clone C0) and subclonal mutations in treatment-naive and post-treatment tumours. **b**, Subclonal mutations determined for multiregional samples from treatment-naive patients (grey, left) and for tumours pre-/post-first-line systemic platinum-based chemotherapy (middle and right). Patients are grouped according to clinical response (middle) and exposure of relapsing tumours to previous radiation. Median and interquartile range, minimum and maximum values. Mann–Whitney *U*-test, two-sided, *P < 0.05, **P < 0.01. **c**, Mutational signatures for paired pre-/post-treatment samples from patients receiving chemotherapy. Relative contributions assigned to clonal (grey) and subclonal mutations of pre-therapy (blue) and post-therapy tumours exposed to platinum-based chemotherapy (blue, *n* = 21) and to additional site-specific radiation (pink, *n* = 25). Median and interquartile range and whiskers (minimum and maximum values). Paired two-sided Wilcoxon test, **P < 0.01. **d**, Seven patients were receiving second- or third-line treatment with ICI, and the scheme for their clinical course is shown in Fig. 1b. Waterfall plot showing tumour site-specific response to ICI (lower right). Numbers of private subclonal mutations pre- and post-ICI, grouped according to clinical response (lower right, median with maximum and minimum values). **e**,**f**, Clonal dynamics at first diagnosis (treatment-naive, grey box), at relapse following first-line chemotherapy (post-chemotherapy, orange arrows and dashed box) and following treatment with ICI (post-ICI, blue arrows and dashed box). Arrows assigned to branches of clone trees indicate the relative contribution of mutational signatures in ancestral clone C0 and subclones. Site-specific CCFs of tumour clones are plotted. Clinical response to the respective treatment is indicated, distinguishing patients with progressive disease (**e**) and partial response (**f**) under ICI. **g**, Relative contribution of mutational signatures in patients receiving ICI assigned to clonal and subclonal mutations of tumours post-chemotherapy and post-ICI. NS, not significant.

**Extended Data Fig. 2:**
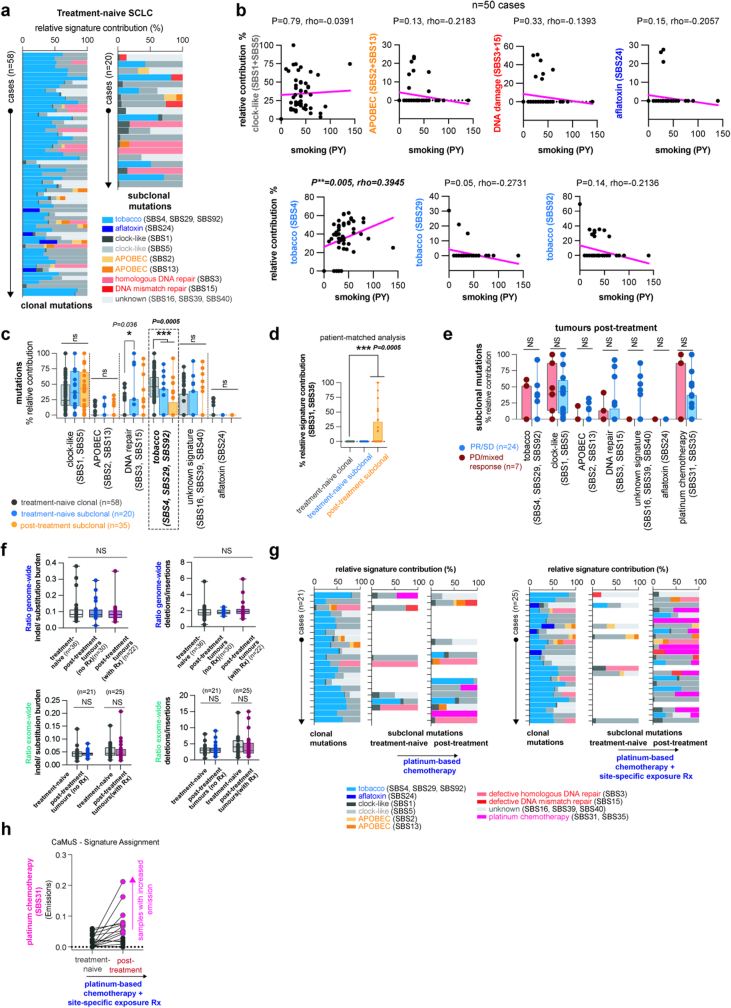
Mutational signatures in 65 patients with SCLC. **a**, Relative contribution of mutational processes in treatment-naïve tumours, referring to clonal mutations as part of the common ancestral clone (left panel, determined for n = 58 treatment-naïve patient tumours) and to subclonal mutations (right panel, determined for n = 20/58 patient tumours). Mutational signatures refer to single base substitutions (SBS) defined in COSMIC (*Alexandrov et al, Nature 2020*; Supplementary Table 5). **b**, Correlation of the amount of smoking determined as packyears (PY) with the relative contribution of mutational processes defined for clonal mutations. Correlations with tobacco exposure were performed for n = 48 cases for which the amount of smoking was documented. Spearman’s rho correlation coefficient determined, significance at P* < 0.05. **c**, Activity of mutational signatures assigned to clonal (grey, n = 58) and subclonal proportion of tumours determined for n = 20 treatment-naïve (blue) and n = 35 post-treatment tumours (orange). Data is presented as box plot displaying median with interquartile range and whiskers indicating maximum and minimum values. Mann-Whitney *U*-tests, two-sided, at P* < 0.05 and P*** < 0.001. Significant differences for all groups are highlighted by dashed boxes. NS, not significant. **d**, Activity of platinum-based mutational signatures (SBS31, SBS35) in patient-matched paired analysis of the clonal proportion, and the subclonal proportion of treatment-naïve tumours and of tumours after first-line platinum-based therapy. Significance determined for n = 24 patients with pre-/post-therapy tumours, Wilcoxon test, two-sided, P*** < 0.001. Data is presented as box plot showing median with interquartile range and whiskers indicating maximum and minimum values. **e**, Activity of mutational signatures assigned to the subclonal proportion of post-treatment tumours following first-line platinum-based therapy. Patient samples are grouped according to clinical remissions or stable disease (blue, PR, SD) or progressive disease (red, PD and mixed responses). Mann Whitney *U*-test, two-sided, significance determined at P* < 0.05, NS not signficant. Data is presented as box plot showing median with interquartile range and whiskers indicating maximum and minimum values. **f**, Rates of insertions and deletions (indels) versus single base substitutions (SBS), and rates for deletions versus insertions in tumours acquired from treatment-naïve patients and post-treatment following platinum-based chemotherapy without and with additional site-specific exposure to radiation (“no Rx” and “with Rx”). Data presented as box plot with median and interquartile range and whiskers for maximum and minimum value. Ratios were determined for whole genome sequencing data (genome-wide, top plots, Mann Whitney *U*-test, two-sided,) and whole exome sequencing data (exome-wide, bottom plots, Wilcoxon matched pair test). NS, not significant at P < 0.05. **g**, Relative contribution of mutational processes assigned to clonal and subclonal mutations in paired studies of pre-treatment (treatment-naïve) and post-treatment tumours following platinum-based chemotherapy (cases, left panel) and additional site-specific exposure to radiation (Rx) (cases, right panel). **h**, Relative contribution of mutational signatures assigned to platinum chemotherapy (SBS31) for paired studies of tumours from n = 25 patients with subclonal mutations identified in the treatment-naïve setting and after treatment with chemotherapy (post-chemotherapy) and additional site-specific exposure to radiation (Rx). The analysis was performed with CaMuS (*Cartolano et al., Scientific Reports, 2020*; right plot; Supplementary Table 5, Methods).

To pinpoint the underlying processes that cause the observed treatment-dependent increase in clonal diversity, we determined signatures for mutations defining the common ancestor and subclones^17^. Confirming previous studies in lung cancer^18–21^, age-like and tobacco-associated processes dominated within the mutations of the common ancestor, which correlated with the level of smoking in these patients (Spearman correlation = 0.39, **P < 0.01; Fig. 3a and Extended Data Fig. 2a,b). Furthermore, clonal mutational processes in some patients were related to apolipoprotein B messenger RNA-editing enzyme, catalytic polypeptide (APOBEC), defective DNA repair and aflatoxin, the latter previously associated with lung cancer^22^. Mutational processes assigned to subclones were less frequently associated with tobacco exposure, and we observed a predominance of clock-like signatures shaping subclonal mutations in both treatment-naive and recurring tumours (Fig. 3a–c and Extended Data Fig. 2c), implying that branching from ancestral clones involved acquisition of mutations at a steady rate, which may have happened earlier throughout the patient’s lifetime^21^. We furthermore identified, in a subset of patients, mutational patterns associated with platinum-based chemotherapy (single-base substitutions SBS31 and SBS35), which were presumably acquired during first-line chemotherapy^23,24^ (Fig. 3a and Extended Data Fig. 2d).

We proposed that the extent of subclonal diversity and associated mutational signatures at relapse relate directly to the type and efficacy of previous treatment. Patients with clinical response to systemic treatment with first-line platinum-based chemotherapy exhibited a significant increase in subclonal mutations when analysing tumours before treatment and at relapse (**P < 0.01); by contrast, the number of subclonal mutations in specimens before and after chemotherapy from patients with refractory SCLC did not differ significantly compared with the level of subclonality determined for multiregional samples in treatment-naive patients (Fig. 3b). These observations support the notion that treatment fails to suppress the original dominating clone in chemorefractory patients whereas successful chemotherapy eliminates the most abundant clone, which is followed by the observed expansion of a multitude of subclones.

The level of subclonal mutations differed substantially across samples (Fig. 3b), and we could not identify specific mutational processes that related to the efficacy of chemotherapy in these patients (Extended Data Fig. 2e). By contrast, independent of the overall clinical response, we found a significant increase in subclonal mutations when analysing those tumour sites at relapse that had also been exposed to radiotherapy (Fig. 3b and Methods). Ionizing radiation does not typically induce signatures marked by single-base substitutions, and we could not identify other signs of radiation-induced DNA damage in tumours at relapse^25^ (Extended Data Fig. 2f). To our surprise, however, paired studies of pre- and post-therapy tumours frequently showed platinum-associated genomic scars in those sites previously exposed to radiotherapy (Fig. 3c and Extended Data Fig. 2g–i). The mutational patterns that underlie platinum damage have previously been identified both analytically and experimentally^23,24^, and our own confidence in the respective assignments is based on both the large number of specimens (26%, *n* = 12 of 46) and significant increase in platinum damage in tumours at relapse (**P < 0.01; Fig. 3c, Extended Data Fig. 2d and Supplementary Table 5). Although we have no formal explanation for this observation, our data are compatible with the view that marked tumour growth suppression by radiotherapy permits the outgrowth of diverse subclones, including tumour clones that had acquired genomic scars from previous lines of chemotherapy^23^.

## Tumour evolution under immunotherapy

**Extended Data Fig. 3:**
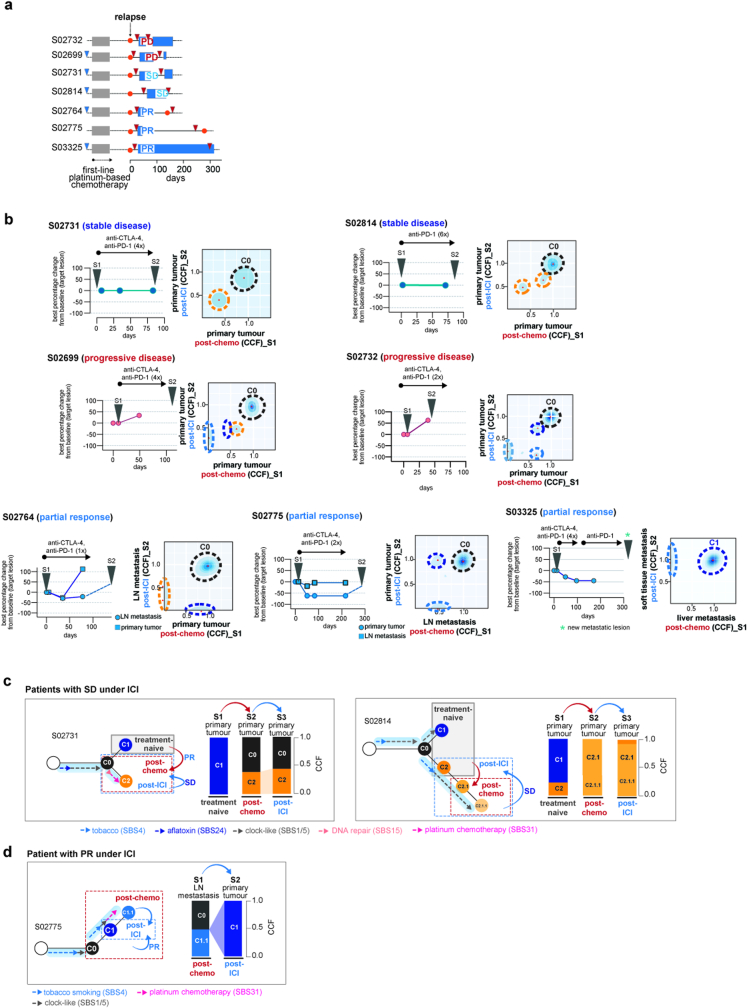
Clinical response and clonal dynamics in patients receiving second- or third-line treatment with immunotherapy. **a**, Scheme for clinical course of seven patients receiving 2^nd^ or 3^rd^ line treatment with ICI. Annotation as in Fig. 1b. Wedges indicate sample acquisitons at first diagnosis (blue) and pre- and post-ICI (red). **b**, Clinical response in seven patients receiving 2^nd^ or 3^rd^ line treatment with immune checkpoint inhibitors (ICI). Patients revealed either a stable disease (n = 2), progressive disease (n = 2) or a partial response (n = 3). Timepoints for sample acquisitions are indicated (grey wedges). Site-specific tumour responses are plotted. Genomic analysis of tumours pre- and post-ICI is described as 2-dimensional contour plots in which cancer cell fractions (CCF) for each mutation are plotted and assigned to clusters of mutations as ancestral clones C0 (grey) and subclones (colored dashed circles, compare “Supplementary Appendix-Patient Cases”). **c,d**, Tumour phylogenies for patients with stable disease (SD, **c**) or for one patient with partial response (PR, **d**) following ICI treatment. Samples were acquired at first diagnosis (treatment-naïve, grey box), at tumour recurrence after first-line platinum-based chemotherapy (pre-ICI, red arrows, red dashed box) and after treatment with ICI (post-ICI, blue arrows, blue dashed box). The response to the respective treatment is indicated. Dashed arrows assigned to the branches of the phylogenetic trees refer to the relative contribution of mutational signatures assigned to the common ancestral clone C0 and to subclones. Temporal distinct tumours are numbered S1, S2 and S3, and the site-specific clonal composition is plotted for all samples.

We reasoned that the burst in clonal diversity induced by chemotherapy might impact the efficacy of any subsequent treatments such as ICI. We therefore analysed the evolutionary dynamics in seven patients who had received, as second- or third-line treatment, the PD-1 inhibitor nivolumab, alone or in combination with the CTLA-4 inhibitor ipilimumab (clinical trial no. NCT03083691). We sampled tumour biopsies before and after treatment with ICI, and in five patients we also performed comparisons with the treatment-naive tumour acquired at the time of first diagnosis (Figs. 1b,c, 2d and 3d and Extended Data Fig. 3a). Two patients experienced disease stabilization throughout treatment with ICI and, in agreement with radiological disease assessment, subclonal tumour cell populations before and throughout immunotherapy were conserved (Fig. 3d and Extended Data Fig. 3b,c). Two patients who progressed under immunotherapy exhibited a limited but detectable change in subclonal mutations, and assignment of tumour clones showed shifts to ancestral clones already existing before the initiation of ICI (Fig. 3d,e and Extended Data Fig. 3b). Thus, tumour progression under immunotherapy led to the expansion of subclones already extant at the time of relapse. This was similarly observed in one patient who experienced an initial clinical response to ICI (S02775; Extended Data Fig. 3d). By contrast, two patients who experienced tumour shrinkage under ICI showed an increase in subclonal mutations at the time of relapse (S02764 and S03325; Fig. 3d and Extended Data Fig. 3b). In comparison with corresponding treatment-naive tumours, we found that these subclones originated from ancestral clones that were dominant at the time of first diagnosis in these patients (Fig. 3f). Thus, tumour clones that initially dominated tumour sites at the time of first diagnosis—and that had effectively been suppressed by first-line chemotherapy and not identified at the time of relapse—reappeared and provided the seed for tumours causing relapse following subsequent lines of immunotherapy. Furthermore, similar to our observation in irradiated tumours, recurring tumour clones dominating at relapse following effective immunotherapy exhibited imprints of platinum-based DNA damage (*n* = 4 of five patients with stable disease and partial response; Fig. 3f,g, Extended Data Fig. 2c and Supplementary Table 5). The emerging subclone with signs of platinum-based DNA damage was not detectable at the time of relapse from first-line chemotherapy in two patients—before initiation of successful treatment with ICI (Fig. 3g). Of note, the tumour obtained before immunotherapy from patient S03325 contained a subclone with a signature of platinum-based DNA damage, which was different from that detected at the time of relapse post immunotherapy. Furthermore, patient S02764 was refractory to chemotherapy, with a limited subclonal drift following first-line chemotherapy (Fig. 3g and Supplementary Appendix). However, in both patients, at relapse from initially effective second-line immunotherapy, ancestral clones emerged with acquired platinum-related DNA damage, presumably acquired throughout ineffective first-line treatment with chemotherapy.

Taken together, our data show that derivatives of earlier ancestral clones persisted, despite the disappearance of the original dominating clone following first-line therapy, and then reappeared under subsequent lines of therapy thus causing clinical relapse. We could not identify any specific mutational processes or genomic patterns that resulted only from treatment with ICI and that might be indicative for effective ICI therapy. However, our data emphasize that, regardless of the efficacy of first-line treatment, ancestral clones appear to acquire platinum-induced DNA damage throughout first-line chemotherapy. Radiation, or other effective second- or third-line line therapies, can permit the subsequent expansion of these clones, even in the evolutionary short time interval of clinical care.

## Clonality of central genome alterations

**Fig. 4:**
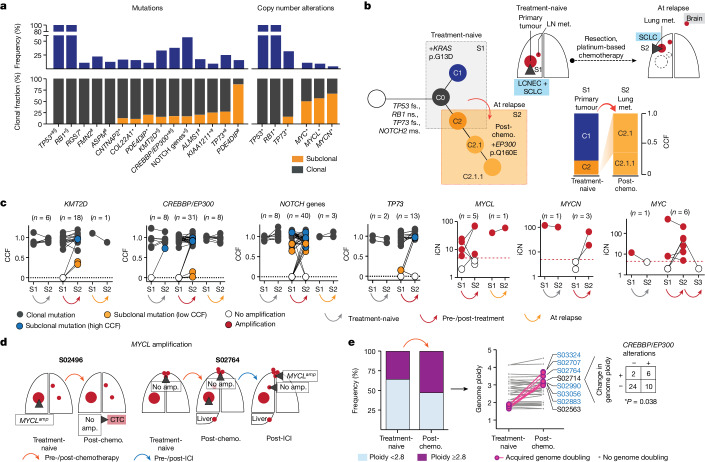
Clonal occurence of key gene alterations. **a**, Gene alterations referring to significant mutations (*), hotspots (#) and damaging mutations (§), and copy number alterations. NOTCH genes include all alterations affecting NOTCH family members (Methods and Extended Data Fig. 4). Corrected *Q* <0.05. **b**, Tumour phylogeny of patient S02814 with mixed SCLC/LCNEC histology harbouring *KRAS* p.G13D at first diagnosis, and SCLC histology and acquired *EP300* p.Q160E at relapse. Additional mutations annotated as ms (missense), fs (frameshift) or ns (nonsense). **c**, Change in CCF of key gene mutations across distinct tumour samples in a patient (S1, S2, S3) acquired either at first diagnosis (treatment-naive), post-treatment or at relapse. Mutations are shown as either clonal (part of the common most recent ancestor, grey), subclonal with lower CCFs (yellow) or higher CCFs identified in distinct samples (blue). For amplifications, changes in integral copy number (iCN) are plotted for distinct patient-matched samples, indicating either no amplification (white) or focal amplifications (red) exceeding iCN > 5 (red dashed line). **d**, Scheme for patients with subclonal occurrence of focal *MYCL* amplifications annotated for sampled tumour sites (dark grey wedges). **e**, Genome ploidy observed in paired tumours from patients at first diagnosis (treatment-naive) and following chemotherapy (post-chemo., *n* = 42). Tumours with acquired genome doubling are highlighted (pink, right), and cases with *CREBBP*/*EP300* alterations are indicated (blue). Fisher’s exact test, two-sided, *P < 0.05.

**Extended Data Fig. 4:**
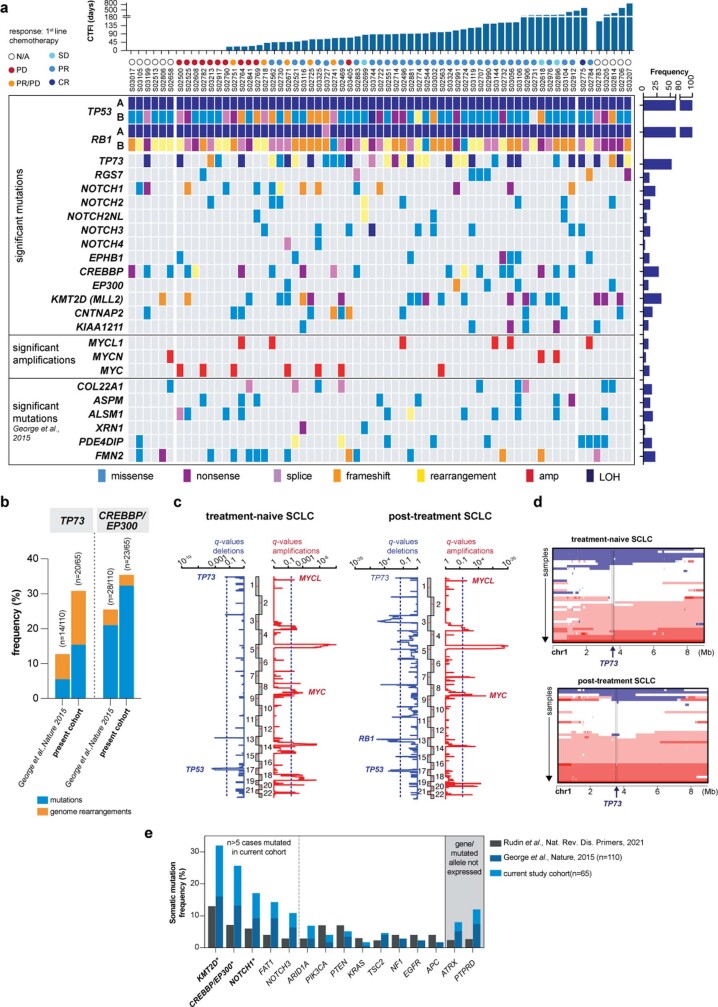
Genomic alterations in 65 patients with SCLC. **a**, Significant genome alterations identified in 65 patients with SCLC. Patients are arranged according to the chemotherapy-free interval (CTFI). The response to first-line chemotherapy is annotated as circles referring to complete response (CR, dark blue), partial response (PR, blue), stable disease (SD, light blue), progressive disease (PD, red) and mixed response (PR/PD, orange). Patient cases at the far left received lung resections and at the far right were alive or lost-to-follow up and the first-line response to chemotherapy was not assessed (N/A), Significant mutations and copy number alterations are annotated according to Supplementary Tables 8 and 9. For *TP53* and *RB1*, alterations for alleles A and B are provided. Additional annotation for significant alterations previously published (*George et al, Nature, 2015*) is provided. Somatic alteration frequencies are provided in the panel on the right side. **b**, Frequency of genome alterations in *TP73* and *CREBBP/EP300* identified in a previous cohort enriched for early-stage SCLC (n = 110, *George et al, Nature, 2015*) and in the present cohort of n = 65 patients with mostly advanced stage SCLC. **c**, Focal copy number alterations identified in treatment-naïve tumours (left panel) and in post-treatment (right panel). Amplifications are plotted in red, and deletions in blue. Dashed blue lines refer to the significance threshold at corrected *Q*-values < 0.05. Genes which are part of chromosomal segments with significant focal copy number changes are highlighted. **d**, Focal copy number loss of the chromosomal segment encompassing *TP73*. The copy number state is displayed as a heatmap for treatment-naïve and post-treatment tumours. **e**, Comparisons of somatic mutation frequencies identified in this cohort (blue) with our previous WGS sequencing dataset (dark blue, *George et al, Nature, 2015)* and with mutation frequencies determined by targeted sequencing panels (*Rudin et al., Nat. Rev. Dis. Primers, 2021*). Genes affected in more than 5 patients within this current cohort are arranged to the left. Gene alterations which were found to not show expression are displayed on the right. Significant gene alterations are highlighted in bold (marked with *, Supplementary Table 8).

**Extended Data Fig. 5:**
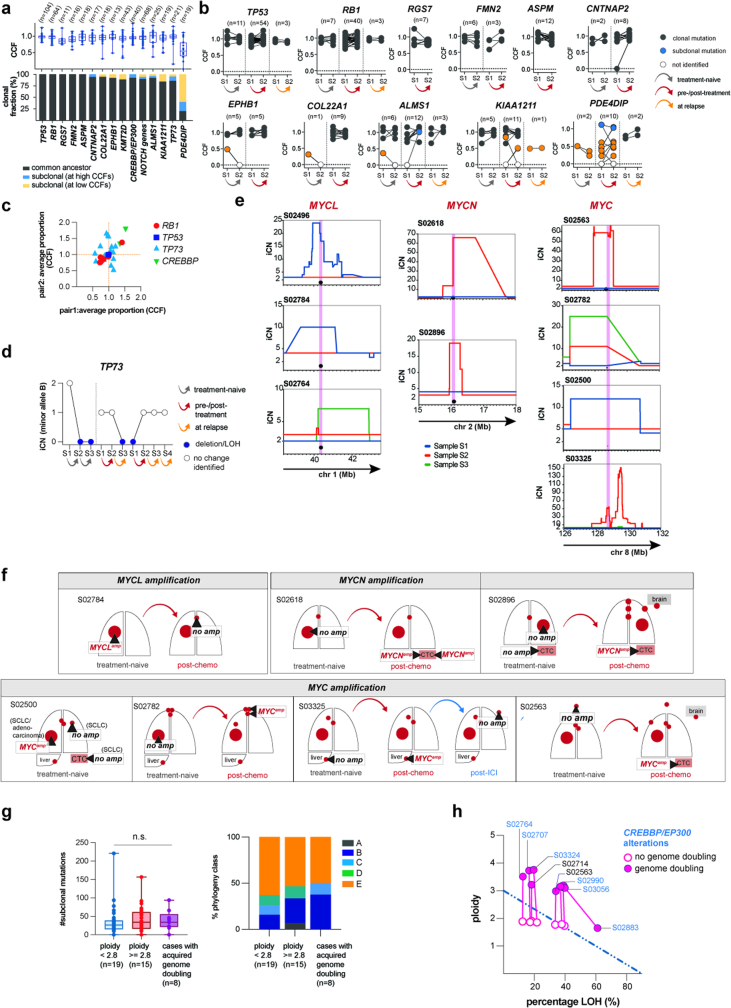
Key genome alterations as part of clonal and subclonal proportions in patient tumours. **a**, Clonal occurrence of significantly mutated genes distinguishing alterations as part of the common ancestor or of subclones with mutations identified at high CCFs (blue) or low CCFs (yellow). The respective CCFs were determined for somatic single nucleotide variants occurring affecting key genes (top panel). Data is presented as median with interquartile range and whiskers to minimum and maximum value. **b**, CCFs determined for significantly mutated genes in distinct tumour samples (S1, S2). Alterations in these genes were assigned to the common ancestor (grey) or identified as subclonal in only one patient case either at higher CCFs (blue) or lower CCFs (yellow). Connected lines describe the change of the CCF of a given mutation across different sites in a patient in the treatment-naïve setting, pre-/post-treatment or at relapse. **c**, CCFs determined for genomic rearrangements identified in key genes. CCFs were determined with SVclone (Methods) referring to whole genome sequencing data (Supplementary Table 7). All gene alterations were assigned to the clonal proportion of the respective sample. **d**, Copy number states for *TP73* plotting copy number losses of one allele (referred to as the minor allele B with loss of heterozygosity, LOH). Copy numbers were determined as integral copy numbers (iCN) for patient-matched samples (S1, S2, S3). **e,f**, Copy number states of *MYC* transcription factors. Copy numbers were determined as integral copy numbers (iCN) and plotted for all samples (S1, S2, S3) analysed in patients with private focal and high-level copy number amplifications. The respective chromosomal position for *MYCL* (chromosome 1), *MYCN* (chromosome 2) and *MYC* (chromosome 8) is indicated and the gene locus is highlighted (pink) (**e**). A schematic overview of the affected sites in patients with subclonal occurrence of focal *MYCL*, *MYCN* and *MYC* transcription factor amplifications is provided and the sampled tumour site is indicated (dark grey wedges) (**f**). **g**, Level of subclonal mutations (left panel) and distribution of phylogeny classes (right panel) in the paired analysis of tumours pre- and post-chemotherapy (n = 42), further distinguishing ploidy states of cancer genomes (lower ploidy with ploidy <2.8, and higher ploidy with ploidy 2.8 or above) and cases which acquired genome doubling. Data is presented as median with interquartile range and whiskers to minimum and maximum value. Two-sided Mann-Whitney *U*-test, NS, not significant at P* < 0.05. **h**, Whole genome doubling (WGD) determined according to genome ploidy (y-axis) and the fraction of the genome with LOH (x-axis). The dotted blue line distinguishes tumours with (pink) and without (white) genome doubling, following previously described approaches (*Dentro et al., Cell, 2021; Bielski et al. Nature Genetics, 2018*). Connected lines refer to paired tumours pre- and post-treatment. Sample IDs are provided highlighting in blue patients with *CREBBP*/*EP300* alterations.

We next sought to identify those genomic alterations that segregate with treatment-associated clonal diversity in SCLC. We confirmed a key role of *TP53* and *RB1*, which were altered as part of the common ancestral clone in all patients (Fig. 4a, Extended Data Figs. 4a and 5a–c and Methods). In agreement with previous studies^26^, tumours with a combined histology at the time of first diagnosis (S02500, S02814 and S02917) also harboured *TP53* and *RB1* alterations as part of the common ancestor (Supplementary Appendix) whereas oncogenic mutations, such as in *KRAS*, were no longer apparent in relapsing tumours with SCLC histology^19,27^ (Fig. 4b).

**Extended Data Fig. 6:**
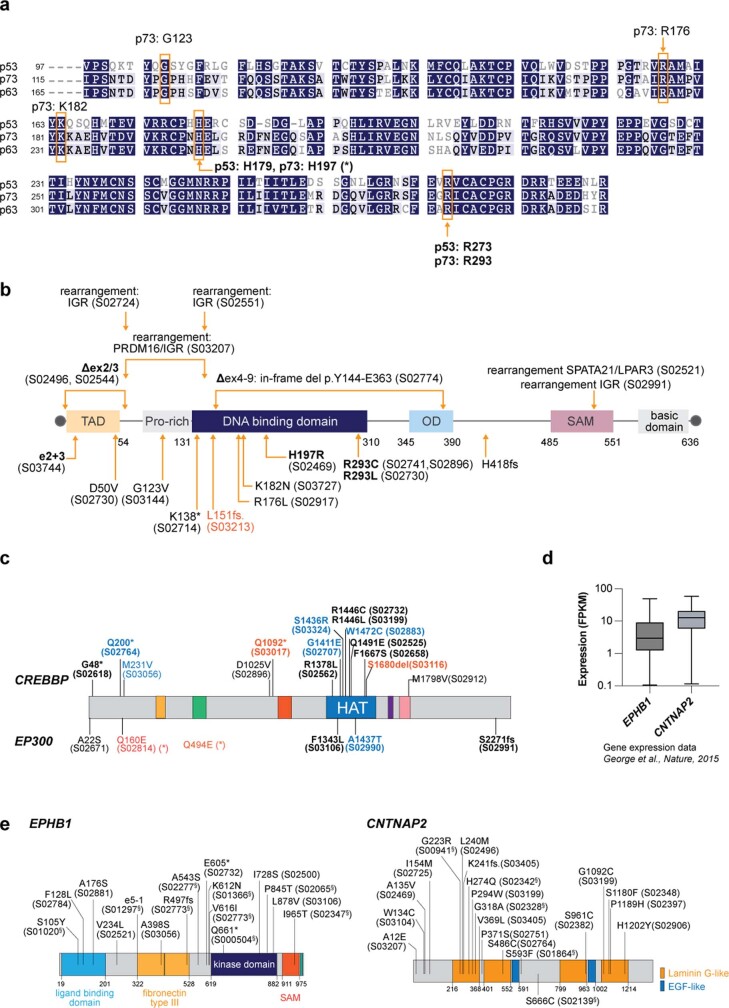
Genome alterations in *TP73*, *CREBBP*/*EP300* and novel key genes. **a**, Protein sequence alignment of p53 family members referring to the DNA binding domain of p53 (NP_000537), p63 (NP_003713) and p73 (NP_005418). Conserved residues are highlighted in dark blue. Orange boxes indicate hotspot residues in p53 (*Stiewe et al., Drug Resistance Updates, 2018*), for which equivalent positions in p73 were mutated in our cohort. **b**, Overview *TP73* alterations. Patient samples identified with genome alterations are indicated. Recurrent changes included *TP73*-deltaEx2/3 (*George et al., Nature 2015*), and mutations affecting residue R293. **c**, Schematic representation of protein domains in Crebbp and Ep300 with information on mutated regions identified in *CREBBP* (annotated above) and in *EP300* (annotated below). Damaging alterations are highlighted in red. Hotspot and damaging alterations are presented in bold. Mutations found in tumours with acquired whole genome doubling during treatment are highlighted in blue. **d**, Expression levels of key gene alterations in *EPHB1* and *CNTNAP2* identified in the combined analysis of this cohort and earlier studies (Supplementary Table 8). Gene expression levels were determined based on the transcriptome data of SCLC tumours (n = 81, *George et al., Nature 2015*), and displayed as median with interquartile range and whiskers to minimum and maximum value. **e**, Schematic representation of mutated residues affecting protein domains in *EPHB1* (left panel) and *CNTNAP2* (right panel). Patient samples identified with genome alterations are indicated. Samples denoted with § were studied as part of earlier cohorts (*George et al., Nature 2015)*.

Our genome data confirmed a significant role of key genes previously identified in cohorts enriched for early-stage tumours^5,6^. We also applied different approaches to identify significantly mutated genes with various levels of stringency and found that the core set of mutated genes was shared between other models and ours (Methods, Supplementary Tables 6–9 and Extended Data Figs. 4 and 6). Whereas the functional relevance of *CREBBP*/*EP300* and *TP73* was identified previously when analysing locally clustered hotspot and damaging mutations^5,6^, our present cohort enriched for metastatic SCLC showed higher mutation frequencies of these genes (*Q* < 0.01; Methods and Extended Data Fig. 4b). We also identified significant focal chromosomal losses of *TP73* and recurrent mutations of position R273 and other conserved residues in *TP73*, which are homologous to known hotspot mutations of *TP53* (ref. ^28^) (Extended Data Figs. 4c,d and 6a,b). Our data thus further emphasize the functional relevance of *TP73* and *CREBBP*/*EP300* in advanced-stage SCLC.

We performed a combined analysis of this cohort and previously published datasets^4–6^ (Methods), which showed significant mutations in ephrin-type B receptor 1 (*EPHB1*) and neuronal cell-adhesion gene *CNTNAP2* (Supplementary Table 8 and Extended Data Figs. 4a,e and 6d,e). Although the majority of these significantly mutated genes were frequently part of the common ancestor (Fig. 4a), some exhibited signs of ongoing subclonal evolution including protein-damaging alterations, hotspot mutations and focal losses affecting *CREBBP*/*EP300*, *TP73*, *KMT2D* and NOTCH genes (Fig. 4c and Extended Data Fig. 5a–d). Several of these alterations were enriched in the outgrowing tumour at relapse, thus further indicating a role in conferring acquired resistance to chemotherapy. To our surprise, significant high-level focal amplifications of all three *MYC* family genes (*MYC*, *MYCL1* and *MYCN*) were frequently identified as subclonal events private to one tumour site sampled (56%, *n* = 9 of 16 cases), occurring either before (*n* = 3) or after therapy (*n* = 6), whereas patient-matched spatially or temporally distinct tumours lacked the amplification event (Fig. 4c,d and Extended Data Figs. 4c and 5e,f). Thus, despite their undoubted role in SCLC^29–32^, *MYC* gene amplifications are often not part of the most recent common ancestor.

SCLC genomes are frequently polyploid, which is typically associated with inferior clinical outcome in cancer^33,34^. In our cohort, 36% of untreated tumours (*n* = 15 of 42) exhibited with higher ploidy, which had no impact on clinical response to first-line therapy and clonal diversity throughout treatment (Extended Data Fig. 5g). However, in these 42 pairs of tumours obtained before and after chemotherapy, tumours in eight patients exhibited events of acquired genome duplication at the time of recurrence. The majority of these tumours harboured either functionally relevant HAT domain mutations^6^ or damaging alterations in *CREBBP*/*EP300*, all of which were part of the common ancestor (*n* = 6 of 8, *P < 0.05; Fig. 4e and Extended Data Figs. 5g and 6c). Thus, acquired resistance in tumours bearing clonal *CREBBP*/*EP300* alterations may be driven by genome duplication, which could potentiate the oncogenic functions of *CREBBP*/*EP300* already present in the founder clone^33,34^.

We could not identify significant mutations that occurred exclusively in subclonal fractions across all patients, or those that may be related to specific mutational processes. Thus, overall, our observations provide further support for a central role of the founder clone, universally defined by mutations of *TP53* and *RB1*, in driving relapse. Furthermore, in several instances specific somatic alterations in genes implied in the biology of SCLC are enriched—but not exclusively—in recurring tumours and are therefore also likely to play a mechanistic role in the processes of drug sensitivity.

## Impact of mutations on drug sensitivity

**Extended Data Fig. 7:**
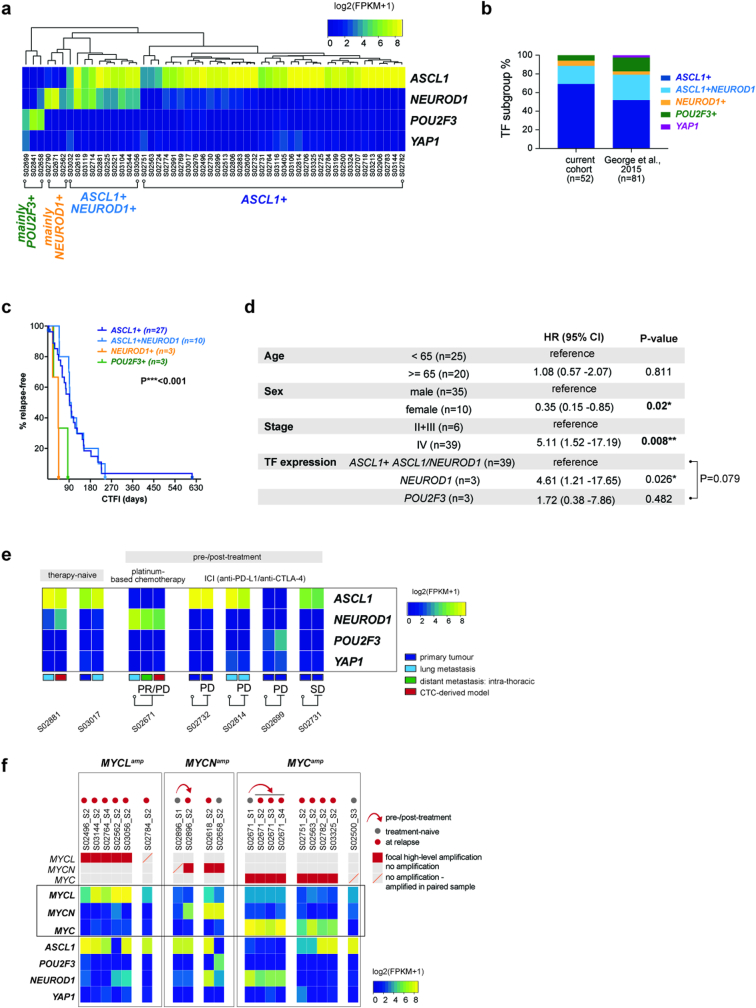
Gene expression of key lineage transcription factors in SCLC. **a,b** Expression of the four key lineage transcription factors *ASCL1*, *NEUROD1*, *POU2F3* and *YAP1* in our cohort (n = 52 patients), categorized as: *ASCL1*+, *ASCL1*+/*NEUROD1*+ double-positive, mainly *NEUROD1*+ and mainly *POU2F3*+ positive tumours (heatmap representation, **a**). The distribution of patient samples with the expression of key transcription factors to an independent larger cohort of n = 81 samples (*George et al. Nature 2015*, **b**). **c,d** Kaplan-Meier curve and Cox regression model for relapse-free survival in patients stratified according to the expression of key transcription factors. **c**, Log-rank Mantel-Cox test at P*** = 0.0009, **d**,Significant associations are highlighted in bold. Hazard ratios (HR) were determined at 95% confidence interval and significance was determined by Log-rank tests at P** < 0.01. **e**, Expression of *ASCL1*, *NEUROD1*, *POU2F3* and *YAP1* in patient-matched spatially or temporally distinct tumours. Samples were analysed across distinct sites in treatment-naïve patients, or pre- and post-treatment with platinum-based chemotherapy or immune checkpoint inhibition (ICI). Sampled tumour sites and the response to the respective treatment is indicated. **f**, Expression of MYC transcription factor family members and lineage transcription factors in cases found with focal and high-level amplification of MYC genes in at least one of the multi-regional tumour sites. Sample names and the MYC gene amplification status is provided in the top panel further indicating if the tumour was samples in the treatment-naïve setting or at relapse after treatment with platinum-based chemotherapy. The expression levels are provided as a heatmap.

We next sought to study how molecular features in SCLC determine the response of patients to first-line platinum-based chemotherapy. Recent studies have proposed a major role for the expression of lineage transcription factors in treatment response in SCLC^8,9,32,35^. In the present study, too, cases with predominant expression of *POU2F3* or *NEUROD1* showed a trend towards inferior relapse-free survival; however, sample size was small (*n* = 3) and correlations did not remain significant following correction for clinical parameters (Extended Data Fig. 7a–d and Supplementary Table 10). Furthermore, although studies in mice have suggested a plasticity in the expression of lineage transcription factors due to tumour progression and chemotherapeutic intervention^32,35^, spatially and temporally distinct tumours from patients with SCLC in our cohort did not show changes in the expression of these key transcription factors^32,35^ (Extended Data Fig. 7e). Finally, we could not observe a correlation of *MYC* family gene amplification with the expression of key transcription factors or subtype conversion in our cohort (Extended Data Fig. 7f).

**Fig. 5:**
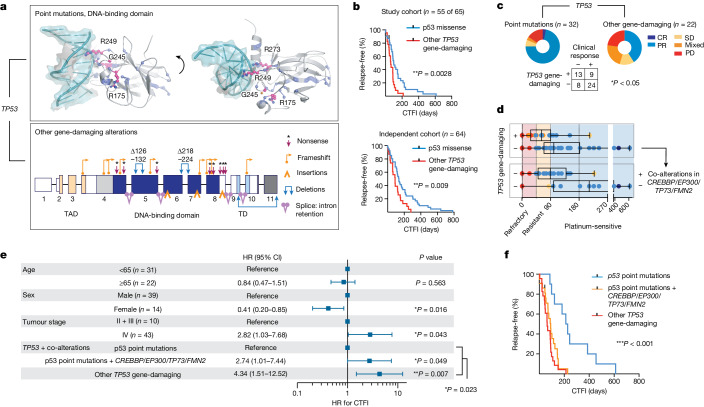
*TP53* and significant co-alterations impacting chemotherapeutic response. **a**, Somatic alterations in *TP53*. Point mutations mapped to the protein structure (DNA-binding domain, PDB-ID: 2AHI, top). Hotspots (pink, residues annotated), other point mutations (blue) and interaction with DNA (teal) are shown. Damaging gene alterations creating deletions, insertions and destructive transcripts are described (bottom; transactivation and tetramerization domains (TAD, TD, respectively); transcript ID: NM_000546). **b**, Kaplan–Meier curve of patients grouped for p53 point mutations (blue) and other gene-damaging *TP53* alterations (red). Relapse-free survival refers to CTFI and is plotted for patients in this cohort who received only first-line systemic platinum-based chemotherapy (top, *n* = 55 of 65 patients; grey points, *n* = 2 censored subjects); and for an independent cohort (bottom, *n* = 64 patients). Log-rank test, **P < 0.01. **c**,**d**, Clinical response (defined as complete response/partial response) to first-line systemic chemotherapy for *n* = 54 of 65 patients grouped for p53 point mutations and other gene-damaging *TP53* alterations. Fisher’s exact test, two-sided, *P = 0.022. Patients with information available for relapse-free survival (*n* = 53) were grouped for *TP53* gene-damaging (*n* = 22) or p53 point mutations (*n* = 31) (**c**) and further stratified for co-alterations in *CREBBP*/*EP300*, *TP73* or *FMN2* (*n* = 20) or none (*n* = 11) (**d**). CTFI range was 45, 90 and 180 days (red, yellow and light blue background, respectively). Boxplot, median and interquartile range, minimum and maximum values. **e**,**f**, Relapse-free survival in patients of this cohort receiving only first-line systemic platinum-based chemotherapy (*n* = 55 of 65, *n* = 2 patients censored). **e**, Patients are grouped according to clonal other gene-damaging alterations in *TP53* and p53 point mutations that were further stratified for significant co-alterations of *CREBBP*/*EP300*, *TP73* and *FMN2*. Cox regression model adjusting for age, sex and tumour stage. HR showing the median and 95% confidence interval (CI). **f**, Kaplan–Meier curve (*n* = 55 of 65 patients; grey points, censored subjects, *n* = 2); log-rank Mantel–Cox test, ***P = 0.0003.

**Extended Data Fig. 8:**
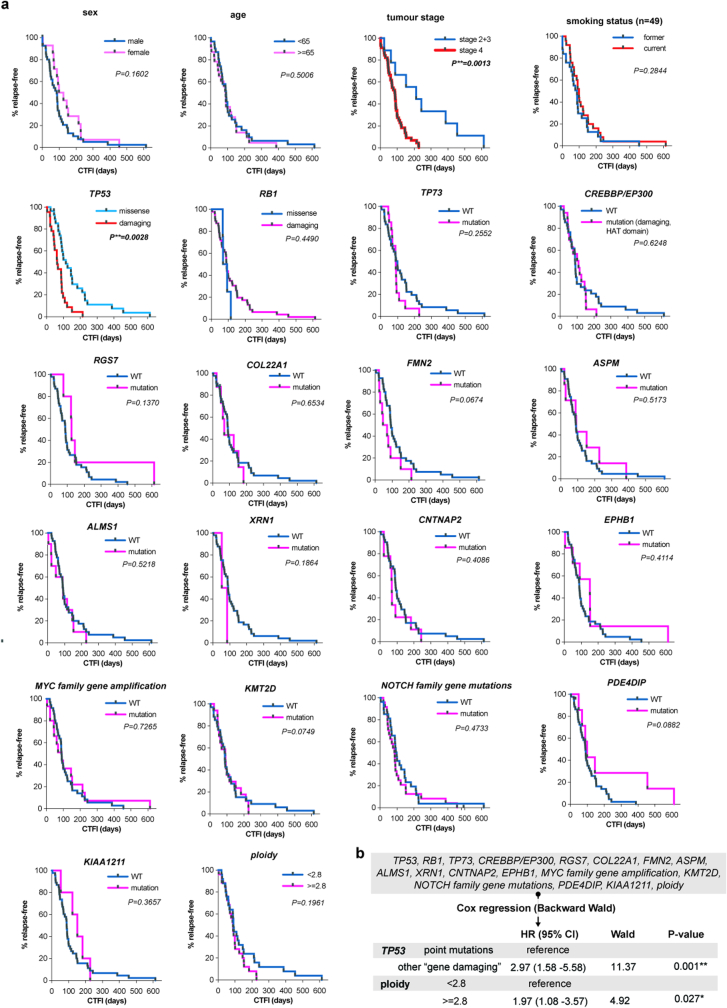
Genomic alterations associating with duration of response to first-line platinum-based chemotherapy. **a**, Kaplan-Meier curves of relapse-free survival for patients with SCLC receiving first-line systemic treatment with platinum-based chemotherapy. Patient were grouped based on clinical variables and significant genome alterations identified in Extended Data Fig. 5. P-values and hazard ratios determined by Log-rank tests and at 95% confidence interval univariable Cox regression models. **b**, Backward Wald tests within Cox models (retention threshold P < 0.05, two-sided) testing all genome alterations described in (**a**).

**Extended Data Fig. 9:**
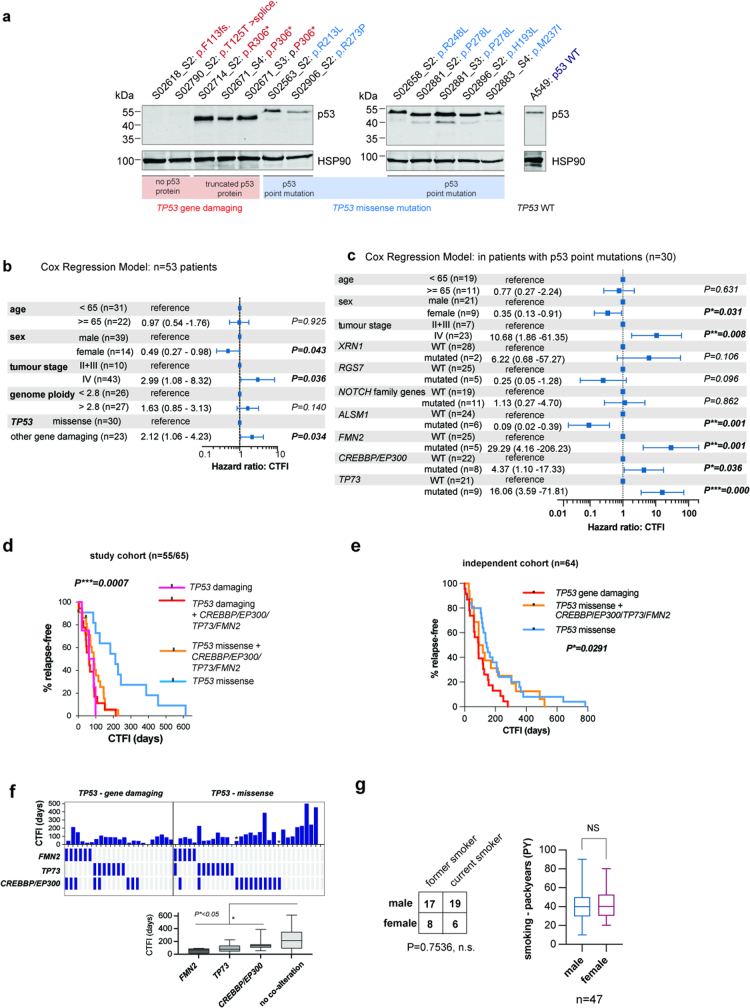
Associations of *TP53* and other key patterns with duration of response to first-line platinum-based chemotherapy. **a**, p53 protein expression in tumour cell lysates of this cohort. Western blot analysis probing with anti-p53 and anti-HSP90 (loading control). *TP53* alterations resulting in gene damaging (red) or other point mutations (blue) are indicated. Lysates of the NSCLC cell line A549 served as a control for wild-type p53 (right lane). Samples were analyzed on two blots, quantitave comparisons are performed on the same blot. Gel source data, see Supplementary Fig. S1. **b,c** Cox regression model for relapse-free survival in patients (n = 53) with SCLC stratifying for gene alterations in *TP53* and adjusting for age, sex and stage (**b**), and analyzing those patients without gene damaging alterations in *TP53* (n = 30) **(c)** with respect to significant genome alterations and clinical variables. Significant associations are highlighted in bold. Hazard ratio (HR) displaying median, 5% and 95% confidence intervals. Log-rank Mantel-Cox test at P* < 0.05, P** < 0.01 and P*** < 0.001. **d–e**, Genome alterations significantly associating with an increased hazard of disease recurrence for patients receiving first-line systemic therapy with platinum-based chemotherapy referring to the current study cohort (n =  n = 55/65 patients, Kaplan-Meier curve: grey points for censored subjects n = 2). Log-rank tests at P*** < 0.001 (**d**), and for the independent cohort of patients with SCLC (**e**). **f**, *TP53*, *FMN2*, *TP73* and *CREBBP*/*EP300* alteration status in patients receiving first-line systemic treatment with chemotherapy (n = 55). Cases are arranged from left to right, and the chemotherapy-free interval (CTFI) is provided for each patient (*n = 2 patients censored). The bar graph at the bottom plots the CTFI for patients with *TP53* point mutations grouped according to the indicated co-alterations. Data is presented as median with interquartile range and whiskers to minimum and maximum value, P* = 0.036; Mann-Whitney U-test, two-sided. **g**, Associations of male and female patients with the smoking status documented as “former” or “current” smoker (Fisher’s exact test, P* < 0.05; left panel). The smoking quantity as measured by the number of packyears is plotted for male and female patients with median and interquartile range, whiskers to minimum and maximum value (Mann-Whitney *U*-test at P* < 0.05, two-sided; right panel). n.s. = not significant.

We therefore proposed that the overall genomic make-up of the common ancestral clone is the main driver of the sensitivity of patients to first-line chemotherapy. *TP53* and *RB1* alterations were universally part of the common ancestral clone, and we sought to further classify alterations in both genes according to their impact on the functionality of the encoded protein. We distinguished between missense mutations creating a full-length protein and other somatic alterations as probably ‘gene damaging’ due to either out-of-frame transcription, early termination or larger insertions or deletions impacting protein expression (Fig. 5a, Supplementary Table 11 and Methods). When assessing clinical outcome as a function of the qualitative nature of all significant gene alterations, we thus identified a higher risk of relapse in patients with these ‘other gene-damaging’ alterations in *TP53* (**P < 0.01; Fig. 5b and Extended Data Fig. 8a,b), which had similarly been observed in other lung cancers^36^. Although patients frequently harboured point mutations in the DNA-binding domain of *TP53* affecting well-known hotspot sites^28^, gene-damaging alterations occurred in 40% of patients and we confirmed either truncated or absent protein products in tumours of these patients (Fig. 5a and Extended Data Fig. 9a). By contrast, damaging alterations constituted the vast majority of all *RB1* lesions (95%; Supplementary Table 11) and no difference in response could be identified. Although frequently part of subclones, *MYC* gene amplifications were also not found to correlate with chemotherapeutic response (Extended Data Fig. 8). *TP53* gene-damaging alterations associated with marginal or no response to chemotherapy (*P < 0.05; Fig. 5c) resulted in a median time to disease recurrence of 63 days and almost all patients relapsed within 6 months (*n* = 22 of 23; Fig. 5d). This observation remained significant in Cox regression models considering all genomic patterns after adjusting for age, sex and tumour stage (hazard ratio 2.12 and 95% confidence interval 1.06–4.23; Extended Data Fig. 9b,c). On the basis of these findings, we analysed an independent cohort of 63 patients with SCLC who were treated with first-line platinum-based chemotherapy, to validate the clinical relevance of destructive *TP53* mutations. In this cohort, too, damaging alterations of *TP53* segregated with a short duration of relapse-free interval (Fig. 5b and Supplementary Table 12).

Because some key mutations were acquired throughout the course of treatment, we next proposed that co-alterations of relevant genes might also impact patient survival. We therefore performed regression models and found that co-alterations of *CREBBP*/*EP300*, *TP73* or *FMN2* increased the relative risk of disease recurrence in patients without *TP53-*damaging alterations, which remained significant when adjusting for clinical parameters (HR 2.74, 95% confidence interval 1.01–7.44, *P < 0.05); a similar trend was observed in the independent patient cohort (Fig. 5e,f and Extended Data Fig. 9c–e). Furthermore, co-alterations of *CREBBP*/*EP300*, *TP73* or *FMN2* suggested epistasis (Extended Data Fig. 9f). Of note, in addition to stage, our data showed longer relapse-free survival in women not related to smoking behaviour in these patients, and may point to a sex bias (Fig. 5e and Extended Data Fig. 9g). Taken together, our genome analyses show that *TP53-*damaging alterations associate with resistance to chemotherapy and that coexisting alterations of *TP73*, *CREBBP*/*EP300* or *FMN2* compromise the clinical efficacy of chemotherapy in patients with SCLC—even in the absence of gene-damaging *TP53* alterations.

## Discussion

Our findings provide a mechanistic explanation for the clinical phenomenon of the initial high sensitivity of SCLC to first-line platinum-based chemotherapy followed by rapid relapse. We show that effective chemotherapy leads to elimination of a rapidly growing, pseudo-clonal population of cancer cells that dominates the tumour at diagnosis, followed by expansion of a large number of subclones derived from the common ancestor. We identify the primary tumour as a site with ongoing evolutionary adaption: following treatment-induced evolutionary pressure, ancestral clones already present in the primary tumour emerge from the common ancestor and give rise to subclones shaping clinical relapse. Our study thus establishes a critical role for the genomic context of the common ancestor in drug resistance, and we uncover its genomic portrait that is largely confined to biallelic losses of *TP53* and *RB1*. Gene-damaging alterations in *TP53* associate with a particularly chemotherapy-resistant state in patients with SCLC, which is in line with studies establishing a role of functionally distinct *TP53* alterations impacting the response to chemotherapy and clinical outcome in cancer^36–38^. However, patients with *TP53* missense mutations can suffer a similarly poor response if co-occurring alterations of *TP73*, *CREBBP/EP300* or *FMN2* complement the dysfunction of *TP53*. Of note, the high frequency of mutations in these genes in the advanced-stage population of this study corroborates our previous reports of an important role of *TP73*, *CREBBP/EP300* or *FMN2* in SCLC^5,6^. Furthermore, adding to our previous discovery of somatic rearrangements of *TP73* (ref. ^5^), we now report recurrent *TP73* hotspot mutations at highly conserved residues. Although it is known that genome ploidy contributes to tumour malignancy and inferior survival^33,34^, we found that clonal mutations of *CREBBP*/*EP300* associated with acquired genome duplication cause relapse and thereby provide a clear genetic mechanism of drug resistance^39^. Finally we demonstrate that although *MYC* family genes play an important role in SCLC biology, amplification events were often not part of the founder clone, and, furthermore, no associations with selection pressure and drug resistance were identified. Thus, our data provide a core set of recurrently altered genes that have a particular impact on drug sensitivity and resistance in SCLC.

Recent studies have established an impact of the expression of lineage transcription factors on drug response^8,9,32,35^. In this study we could not identify notable transcriptional subtype conversion or correlation of major subtypes with treatment response, but found a strong relationship of certain genome alterations with clinical outcome. Future studies focused on combining genome evolutionary processes with single-cell transcriptome data are therefore warranted to elucidate the interplay of genomic and transcriptional heterogeneity in SCLC.

New drugs are typically tested in the second or third line of treatment, almost always with limited efficacy. We speculate that this phenomenon may be due, at least partially, to the massive increase in clonal heterogeneity following first-line chemotherapy described herein. Subclonal diversity following treatment was largely attributable to clock-like mutational processes, thus indicating that subclones at clinical relapse had existed before therapy. Independent of the sensitivity to first-line platinum-based chemotherapy, we found that ancestral clones acquire platinum-induced DNA damage throughout first-line therapy and emerge at relapse, which is more pronounced after effective radiation. We similarly observed platinum-based genomic damage in patients relapsing following effective ICI. Although we could not determine genomic or molecular patterns associated with response to ICI in these patients, our data demonstrate that genomic damage from first-line chemotherapy can complicate the efficacy and duration of response to other treatments initiated in subsequent lines. Despite the overall short time window of clinical care, effective treatment—including radiation or successful immunotherapy—can accelerate the emergence of ancestral clones with platinum-induced genomic scars that subsequently cause relapse. Although we could not identify specific gene mutations associated with these mutational processes, our data warrant studies focused on the consequences of platinum-induced changes on genome integrity and maintenance.

Overall, our findings related to the most recent common ancestor also have clinical implications. First, it may be an attractive concept for the discovery of new therapies to focus on alterations specifically present in the common ancestor. These may also serve as markers to monitor response and resistance to treatment. Second, we consider effective first-line treatment to be critical also for subsequent lines of therapy, because of the sheer increase in clonal diversity that drives drug resistance. Third, drugs in development may still be efficacious when tested early in the first-line setting, without being affected by a clonally diverse relapse that complicates subsequent lines of treatment.

In summary, we uncover genomic alterations underlying poor response and rapid relapse, which put the most recent common ancestral clone at the centre of cancer genome evolution. Our study therefore emphasizes the need for future therapeutic strategies to be tailored to target the detrimental cellular component of the founder clone to improve the outcome of patients with SCLC.

## Methods

### Human lung tumour specimens

This study was approved by the institutional review board of the University of Cologne. We analysed 160 tumours and patient-matched blood samples from 65 patients with SCLC (Fig. 1a). The samples were collected from multiple collaborating hospitals and clinical facilities under institutional review board-approved protocols, and all patients provided written informed consent. For some patients the material was collected as part of an ongoing clinical trial (BIOLUMA, study no. NCT03083691), and those patients received as second- or third-line treatment anti-PD-1 either alone or in combination with anti-CTLA-4 immune checkpoint inhibitors. The course of treatment for all patients and information on all samples are detailed below and summarized in Extended Data Table 1 and Supplementary Tables 1 and 2.

All tumour samples were pathologically reviewed by at least two independent expert pathologists who inspected the histomorphology based on haematoxylin and eosin and immunohistochemical staining. All tumours were confirmed with SCLC histology; tumours from three patients were diagnosed with additional morphological components of LCNEC or adenocarcinoma (Extended Data Table 1 and Supplementary Table 1). All patient-matched, multiregional tumour and normal blood samples were confirmed as belonging to the same patient by short tandem repeat (STR) analysis conducted at the Institute of Legal Medicine at the University of Cologne, Germany, and further confirmed by genome sequencing data.

In the majority of cases we analysed at least two tumour samples per patient, which were acquired at either single or multiple timepoints throughout the clinical course of treatment (Supplementary Table 2). More than two tumour samples were acquired for 37% of patients (*n* = 24 of 65). For five patients we analysed tumour samples at three distinct time points (*n* = 5 of 65, 8%; Extended Data Table 1 and Supplementary Table 2). Samples were acquired as biopsies and lung resections, and we additionally engrafted tumour tissue from fine-needle biopsies (*n* = 2, one pleural and one lymph node metastasis) and CTCs (*n* = 29 of 160, 18%) onto immune-compromised mice (NSG mice) to establish PDX (in total *n* = 31 of 160, 19%; Fig. 1a); this approach allowed for enrichment of limited tumour material for in-depth genomic studies. Samples analysed as PDX are listed in Supplementary Tables 2 and 3 and are highlighted in Fig. 2d. As previously described^12,13^, sampling a patient’s blood for CTCs provides a minimally invasive approach towards analysis of tumour cells under therapy, and xenotransplant models have been shown to recapitulate the genomic profiles of the patient’s tumour. Xenotransplant models were established following an approach previously described^12^; tumour cells were engrafted subcutaneously into the flanks of 7–14-week-old NSG mice (male and female, NOD.Cg-Prkdcscid Il2rgtm1Wjl/SzJ; Jackson Laboratories), and tumours were harvested at a maximum volume of 1.5 mm^3^. Tumour histology was confirmed by pathological review, and STR profiling with patient-matched normal and tumour samples confirmed the identity of the engrafted patient-derived material. All animals were housed in a specific-pathogen-free facility under ambient temperature and humidity while maintaining a 12/12 h light/dark cycle. Animal experiments were approved by, and conducted in accordance with, the regulations of the local animal welfare authorities (State Agency for Nature, Environment and Consumer Protection of the State of North Rhine-Westphalia, nos. AZ: 84-02.04.2012.A281, 84-02.04.2015.A172 and 84-02.04.2018.A002).

Samples were categorized by location: we referred to the primary lung tumour and grouped metastatic sites as intrapulmonary metastases, including pulmonary and lung and mediastinal lymph node metastases; tumour sites grouped as extrapulmonary metastases include intrathoracic distant metastases of the pleura and extrathoracic distant metastases affecting abdominal sites, the brain or other less common metastatic sites (breast, skin, sternum), as well as CTCs propagated as CTC-derived xenotransplant models, which represent cells that spread to the bloodstream with the potential to seed distant metastases. In patients with highly metastatic disease we furthermore assessed whether, based on radiological images, tumour sites sampled throughout therapy were pre-existing at the time of first diagnosis or before treatment, and whether these sites were exposed to any given therapy (chemotherapy, radiation or immune checkpoint blockade). Furthermore, we assessed whether any samples were taken from a newly formed metastatic site which, according to radiological imaging, was not pre-existing at the time of first diagnosis or before any other treatment exposure. For CTC-derived models, because we had no information regarding whether the tumour site may have shed cells to the bloodstream, we classified any CTC-derived sample as tumour cells that may have been exposed to any given treatment. A schematic overview of the acquired samples and affected organ sites is depicted for each patient in the Supplementary Appendix.

### Clinical characteristics

The clinical characteristics of the patients in our cohort are in line with those typically found in SCLC (Fig. 1b, Extended Data Table 1 and Supplementary Table 1). Median age at the time of first diagnosis was 64 years, and patients were predominantly male (*n* = 43 of 65, 66%) with a history of heavy smoking and a median number of 40 pack years (smoking history was known for 89% of patients, *n* = 58 of 65; the number of pack years was determined for 85% of patients, *n* = 55 of 65). For clinical correlations the following categories were defined: age groups of 65 years or more and under 65. Smoking status was classified as ‘current smoker’, ‘former smoker’ or ‘never smoker’.

The majority of the patients presented with a highly metastatic tumour classified as stages III and IV (*n* = 57 of 65, 88%; additional information on tumour, node and metastasis staging is provided in Supplementary Table 1). Seven patients were diagnosed with limited-stage disease or with tumour stage I, II or IIIA, and were therefore amenable to surgical lung resection.

Although one patient declined further therapy, all other patients in our cohort received systemic treatment with platinum-based chemotherapy. The majority of patients were treated with a combination of cisplatin/carboplatin and etoposide (*n* = 61 of 65; 94%); with regard to recent changes in the treatment of SCLC^2^, additional PD-L1 inhibition was administered to five of these patients. Due to the initial diagnosis with histological components of non-SCLC (adenocarcinoma or LCNEC), two patients were treated with cisplatin/carboplatin combined with vinorelbine (patients S02814 and S02917). Furthermore, one patient received only monotherapy with carboplatin. Throughout the course of treatment 72% of patients (*n* = 47 of 65) received additional radiation, mainly of the chest/lung/mediastinum (*n* = 35 or 47) or brain (*n* = 38 of 47); four patients underwent stereotactic surgery of brain metastases.

The clinical response to treatment was assessed by radiological imaging and classified as either complete response (CR), partial response (PR), stable disease (SD), progressive disease (PD) or mixed response (PR/PD). The clinical response to systemic first-line platinum-based chemotherapy was analysed for *n* = 55 patients; these patients receiving treatment with only systemic chemotherapy and were therefore considered for subsequent correlations of genomic and molecular phenotypes with clinical response. Genomic and molecular correlations with clinical response to chemotherapy were not considered for *n* = 10 patients in our cohort, because these patients were either lost to follow-up (*n* = 2), declined further treatment (*n* = 1) or received a lung resection resulting in differences in the dynamics of disease progression (*n* = 7).

Of the 55 patients who received only first-line systemic platinum-based chemotherapy, 60% (*n* = 33 of 55) responded to treatment with PR (*n* = 32) or CR (*n* = 1), 9% had stable disease (*n* = 5 of 55), 11% showed mixed response (*n* = 6 of 55) and 20% (*n* = 11 of 55) experienced a progressive disease, of which three succumbed to the disease during first-line treatment. Following treatment, two patients experienced fatal sepsis (patient S02608 while receiving treatment and experiencing disease progression; and patient S02658 following completion of chemotherapy; Supplementary Table 1); both patients were consequently censored when performing correlations with relapse-free survival, and the therapy response of patient S02608 was not evaluated. Median progression-free survival was 6.3 months. In addition we determined CTFI as an independent measure of sensitivity and duration of response to first-line platinum-based chemotherapy; median CTFI was 88 days. Fifty-three per cent of patients (*n* = 28 of 53) either did not respond, relapsed or succumbed to the tumour disease within 90 days following completion of first-line chemotherapy (following the guidelines of NCCN)^14^, and these patients were thus clinically classified as either chemorefractory or -resistant). Of the remaining patients who, based on NCCN guidelines, were considered as ‘platinum-sensitive’, 30% (*n* = 16 of 53) relapsed within 6 months following completion of chemotherapy and 17% were relapse-free for more than 6 months (*n* = 9 of 53). At relapse, 83% of patients (*n* = 44 of 53) received second-line systemic therapies that included treatment with anti-PD-1 and/or anti-CTLA-4 immune checkpoint inhibitors (*n* = 27) or other chemotherapeutics, including topotecan (*n* = 8), rechallenge with carboplatin and etoposide (*n* = 2) or combinations of adriamycin, cyclophosphamide and vincristine (*n* = 7) (Fig. 1 and Supplementary Table 1). Following tumour progression, ten patients were amenable to additional lines of therapy including immune checkpoint inhibitors (*n* = 6) or chemotherapeutics (*n* = 4).

The analysis of multiregional and longitudinal tumour sites from 65 patients with SCLC focused on distinct clinical scenarios. For interpatient comparisons we focused on studies of tumour pairs (‘Analysis of clonal architecture from multiregional and longitudinal tumour samples’; Fig. 1c). We focused on distinct clinical scenarios: (1) analysis of tumour samples from spatially distinct sites obtained from treatment-naive patients at the time of first diagnosis (*n* = 16); (2) analysis of temporally distinct tumour sites referring to samples acquired before treatment and during therapy, including those from patients undergoing neo-adjuvant treatment (*n* = 5); and (3) samples acquired before treatment and at relapse following completion of first-line platinum-based chemotherapy (that is, either following an initial response or disease progression despite treatment, *n* = 42). The analysis further focused on (4) spatially, but not temporally, separate tumours analysed solely at the time of relapse (*n* = 14), and (5) tumour sites acquired at the time of relapse from platinum-based therapy and following subsequent lines of treatment with immune checkpoint inhibitors (pre- and post-treatment with ICI, *n* = 7). We thus performed in total *n* = 84 paired analyses of tumour sites in 65 patients with SCLC (Supplementary Table 4).

In addition we performed clinical correlations in an independent cohort of patients with SCLC, who all received first-line systemic treatment with platinum-based chemotherapy; we performed whole-exome sequencing of the tumour samples and identified key genome alterations (*n* = 64 patients; Supplementary Table 12). This cohort was analysed to validate findings described in Fig. 5b; at least 56 samples are required to validate the findings at a significance level of 5% and a power of 80%; thus, we validated our findings at a power of greater than 80%.

### DNA and RNA extraction

Nucleic acids were extracted from fresh-frozen blood or tissue or from formalin-fixed, paraffin-embedded (FFPE) tissue specimens (Supplementary Table 3). Tumour tissues were analysed by haematoxylin and eosin staining and nucleic acids were extracted from regions with a tumour content of at least 70%. All tumour samples derived from murine xenotransplant models showed a tumour content of at least 95% with no discernible innervation of murine cells, which was similarly observed in previous studies^12,13^. Fresh-frozen samples were processed by preparation of tissue sections, each of 20 μm thickness, on a cryostat (Leica) while maintaining a temperature of −20 °C. In the case of FFPE samples, sections of 20 μm thickness were prepared on slides on a microtome. DNA was extracted from both fresh-frozen tissues and EDTA blood with the Gentra Puregene DNA extraction kit (Qiagen) according to the protocol of the manufacturer.

To allow for high-quality sequencing data of FFPE material we applied ultrasonic acoustic energy, using the adaptive focused acoustics technology from Covaris and following the protocol of the manufacturer. DNA isolation was then performed with a bead-based approach (AMPure XP Beads, Beckman) and any fractions containing paraffin material were excluded from subsequent DNA isolation steps.

For samples with limited tumour material we further adjusted protocols, which included repeated rounds of protein and nucleic acid precipitation, to increase the DNA yield for subsequent sequencing studies. All DNA isolates were hydrated in TE buffer and molecular weight was assessed using the Agilent TapeStation system (Genomic DNA ScreenTape no. 5067-5365, Agilent Technologies). DNA isolates from fresh-frozen samples were confirmed as being of high molecular weight (above 10 kb), and samples with evident signs of degradation were excluded from further sequencing studies.

For RNA extraction, tissue sections were first lysed and homogenized with the Tissue Lyzer (Qiagen). Subsequent RNA extraction was performed with the Qiagen RNAeasy Mini Kit according to the instructions of the manufacturer. Alternatively we used the RNAeasy Micro Kit to extract RNA from small tissue biopsies. RNA quality was assessed with RNA Screen Tape (no. 5067-5576, Agilent Technologies) at the TapeStation. Samples with RNA integrity number above 7 were further analysed by RNA sequencing (RNA-seq).

### Next-generation sequencing

All sequencing reactions were performed on either the Illumina HiSeq or NovaSeq sequencing platform. Details on genome sequencing data and quality metrics are provided in Supplementary Table 3. Sequencing data are deposited in the European Genome-Phenome Archive (accession no. EGAS50000000169).

#### Whole-exome sequencing

We performed whole-exome sequencing for all patient samples with the SureSelect Human All Exon V6 Kit (Agilent) following the protocol of the manufacturer. Exon-enriched libraries were subjected to paired-end sequencing on either the Illumina NovaSeq or Illumina HiSeq platform. For the former, libraries were prepared to reach a mean insert size of 200 base pairs (bp) for sequencing with a read length of 2× 100 bp. For the latter, DNA was prepared with a mean insert size of 160 bp for 2× 75 bp paired-end sequencing. Both tumour and normal DNA material were sequenced aiming for a coverage of at least 150× which, following filtering of PCR-duplicated reads and alignment to the annotated human genome (hg19), resulted in an average coverage of 127×. Tumour samples showed a median purity of 88% (interquartile range 78–96%), thus minimizing problems in the assessment of tumour-specific mutations. This allowed for sufficient sequencing depth for reliable analysis for allelic fractions and clonality, as described below. Median genome ploidy was determined at 2.5 (interquartile range 1.9–3.2; Supplementary Table 3).

#### WGS

Whole-genome sequencing (WGS) was performed for samples with sufficient DNA material and quality, additionally providing information on genomic rearrangements not identified by WES. Short-insert DNA libraries from fresh-frozen samples were prepared with the TruSeq DNA Nano PCRfree sample preparation kit (Illumina), and FFPE samples were prepared with the Aceel-NGS 2S Plus DNA library Kit. Paired-end sequencing at a minimum read length of 2× 150 bp was performed, and human DNA libraries were sequenced to an average coverage of 31× for both tumour and matched normal tissue (Supplementary Table 3).

#### RNA-seq

Whole-transcriptome sequencing was performed to determine expression profiles for SCLC tumours in this cohort. RNA-seq was performed with RNA extracted from fresh-frozen human tumour tissue samples. Complementary DNA libraries were prepared from poly-A-selected RNA, applying the Illumina TruSeq protocol for messenger RNA. Libraries were then sequenced with a 2× 100 bp paired-end protocol, generating 50 Mio reads and thus accounting for a minimum mean coverage of 30× of the annotated transcriptome. Samples analysed by transcriptome sequencing are shown in Supplementary Table 2.

#### Dideoxynucleotide sequencing for validation of somatic alterations

If available, transcriptome or additional genome sequencing data were used to validate somatic mutations determined by genome sequencing. In cases without additional sequencing data, dideoxynucleotide chain termination sequencing (Sanger sequencing) was performed to validate key mutations, genomic rearrangements and chimeric fusion transcripts. Specifically, shared clonal mutations of key mutated genome alterations were confirmed by Sanger sequencing as being present in all tumour samples from a patient. For genomic rearrangements determined by WGS in a subset of samples per patient (Supplementary Tables 2 and 3), PCR reactions were performed and the genomic breakpoint was probed and analysed in that subset of samples. Complex genome alterations affecting *TP53*, *RB1* and *TP73* were thus confirmed in all samples of the respective patient (annotation provided in Extended Data Fig. 4). Clonal assessment of genomic rearrangement affecting key genes was determined with SVclone^40^ (see below). For subclonal and private mutations of key gene alterations, Sanger sequencing was performed to confirm both the mutation call and absence of these alterations in matching tumour samples. Primer pairs were designed to amplify the target region encompassing the somatic alteration. PCR reactions were performed with either genomic DNA, whole-genome-amplified DNA or cDNA. Amplified products were subjected to Sanger sequencing and the respective electropherogram was analysed with Geneious v.8 (www.geneious.com).

### Data processing of transcriptome sequencing data

As previously described^5,41^, transcriptome sequencing data were processed with TRUP (tumour-specimen suited RNA-seq unified pipeline). Paired-end reads were mapped to the human reference genome (GRCh37/hg19). Samples obtained from patient-derived xenotransplant models were mapped to a combined human and murine reference genome (GRCh37/hg19 and GRCm38/mm10). Expression levels were determined for uniquely mapped paired-end reads using Cufflinks referring to the human reference genome, and expression levels were quantified as fragments per kilobase exon per million mapped reads (Supplementary Table 10).

### Data processing of genome sequencing data

Raw sequencing reads were processed as previously described^5,6,15^. Reads were aligned to the human reference genome (GRCh37/hg19). Our cohort additionally included patient tumours expanded in immune-compromised mice (*n* = 32 samples; Fig. 1a and Supplementary Table 2). In these cases, sequencing reads of all samples from a given patient (including the normal reference sample and tumour samples obtained directly from the patient and derived from murine xenotransplant models) were aligned to a combined human and murine reference genome (GRCh37/hg19 and GRCm38/mm10), to exclude sequencing reads from murine cells and to allow for uniform processing of all samples from a given patient. Concordant read-pairs were identified as potential PCR duplicates and were subsequently masked in the alignment file and annotated as the number of masked reads. The quality of the sequencing data is summarized in Supplementary Table 3.

Human sequencing reads (mapped to the human reference genome) were analysed for tumour purity, tumour ploidy, somatic mutations and copy number alterations^15^. In addition, WGS data were analysed for genomic rearrangements with the previously described analysis pipeline^5,6,15,42^. Mutation calling was performed as previously described^5,6,43^. In brief, variant counts were assessed for tumour and matching normal samples, corrected for sequencing noise and compared with a database of 300 whole-exome and genome sequenced normal samples to filter and determine somatic mutation calls. Variants at low allelic fractions are often prone to result from sequencing artefacts, which occur as a consequence of sequencing noise arising from high-coverage WES due to either fragmented DNA as part of FFPE material or low-level contamination with murine reads in tumours derived from murine xenograft models. We therefore implemented strict filtering criteria for mutations occurring at allelic fractions of less than 0.2. Mutations were then filtered out if (1) the forward–reverse score was below 0.2 (forward–reverse score is 1.0 if 50% of variant reads are found on the forward or reverse read, and 0 if all variant reads are on one orientation); and (2) the allelic fraction of the variant *v* in consideration of minimal coverage *C* of the normal or matching tumour sample at position *i* (*C*_*i*_^min(tumour/normal)^) did not exceed the read count (rc) threshold with a default value of 10. This was calculated as *C*_*i*_^min(tumour/normal)^ × *v*_*i*_ < rc. We thus introduced a decision boundary that filters out mutations at relatively low allelic fractions and low sequencing coverage; mutations with low allelic fractions but high coverage were retained for further analyses. In addition we adjusted the stringency of this cut-off for individual samples. Although this stringent cut-off limits the identification of subclonal mutations, we have thus controlled for potential sequencing noise and false-positive mutation calls. As described below, multiregional studies may suggest mutations at very low allele fractions in one tumour that might be more abundant at another tumour site. In this instance, truly subclonal mutations at low allelic fractions that were filtered out in one sample at this step of the analysis were reintroduced as somatic mutation calls if the same mutation passed all stringent filtering criteria in another matched tumour sample.

### Analysis of clonal architecture from multiregional and longitudinal tumour samples

We have developed a computational approach to identify individual clones from tumour sequencing data by applying a model that assigns an expected allelic fraction to each mutation under the assumption of clonality (that is, all tumour cells carry this mutation). The expected allelic fraction is corrected for tumour purity, average tumour ploidy and copy number state at the respective genomic coordinates of the said mutation. Relating the observed to the expected allelic fraction results in an estimated CCF that is a specific metric pertinent to each mutation^15,44,45^. Subsequent clustering of CCFs enabled identification of cell clones represented by subsets of individual mutations. The CCFs and associated clones present in a given tumour thus define the overall clonal composition at the time point of sampling. Through a one-dimensional approach to CCF clustering, we determined for each single tumour its clonal composition (one-dimensional mutation clustering^15^), a method benchmarked in pan-cancer studies for tumour heterogeneity^44,45^.

To study tumour evolution from multiregional or longitudinal tumour samples from a given patient, we further developed a two-dimensional approach to analysing pairs of samples from the same patient (two-dimensional clustering) and thus to the reconstruction of clonal dynamics^15,43^ (manuscript in preparation). Information on tumour phylogenies, subclonal mutations, subclones and clonal composition of sites is summarized in Fig. 2 and detailed information is provided in Supplementary Table 4. In addition, tumour phylogenies determined for each patient are provided in the Supplementary Appendix.

The sequencing data of tumour samples in our cohort showed an average purity of 85% (Supplementary Table 3). Thus WES at an average coverage of 127× provided the required sequencing depth to determine subclones in our data. The analysis of tumour subclones focused on mutation calls as determined by exome sequencing in each sample to track individual tumour clones.

The computational method for tumour phylogeny reconstruction starts by executing an extensive set of comparisons and quality controls of copy number states, and a set of mutations and their respective CCFs for each sample. Rather than working with mutation calls and copy number states assessed individually for each tumour sample, we first performed comparisons and adjustments across all samples of a given patient. This included generating a unified copy number segmentation for all samples, which is critical for assigning within each chromosomal segment allele-specific mutation calls, and subsequently to compute CCFs for each mutation. We furthermore created for each patient a unified list of all somatic single-nucleotide mutations (SNMs) determined from each sample, and in all samples we reprobed the presence of somatic mutations of the unified list with relaxed filter criteria for calling somatic mutations at low allelic fractions from sequencing data. This approach allowed us to confirm whether high-confidence mutation calls from one sample were either private events or also present in other patient-matched samples but occurring at lower allelic fractions. Following the refined assessment of copy number states and somatic mutation calls, we determined for each mutation both the observed and expected allele frequency under the assumption of clonality (that is, a cancer cell fraction of 1), so that the CCF of the mutation can be calculated as the ratio between observed and expected allele frequency^15^. We applied additional filter criteria to mark somatic mutations calls occurring near telomers (that is, located in the tails determined by 1.5% of chromosome length) or centromeric regions on the chromosome, where copy number estimations are frequently error prone and therefore lead to a potentially incorrect calculation of CCF.

Somatic insertions and deletions (indel calls) can lead to additional false-positive calls of SNMs as a consequence of improper mapping of reads with inserted or deleted bases. To reduce the number of false-positive SNM calls resulting from indels, we filtered out all SNMs in close proximity (less than 10 bp away) to any mutation call for insertions and deletions.

We applied filtering criteria for mutation calls present on chromosomal areas and which, in multiregional analyses, were found to undergo loss of heterozygosity (LOH) in at least one, but not all, of the samples of a given patient^45–47^. Samples with LOH may not harbour certain mutational calls due to the LOH event, whereas patient-matched samples without LOH may show those mutations. Consequently observed private, or almost private (CCF < 0.2), mutations in one sample lacking LOH events (whereas other patient-matched samples show the LOH event) may indicate a shared clone that undergoes copy number losses, and argue against the subclonal private acquisition of these mutations in this chromosomal area. A clear phylogenetic reconstruction in these cases is not straightforward: due to the inherent uncertainty if the mutations were not present in the other sample (that is, truly private) or lost via the LOH event, these mutations were excluded from phylogenetic tumour clone reconstructions. Following the same criteria, mutations in areas with subclonal copy number events in which one of the copy number clones was hit by an LOH event were also filtered out to avoid further uncertainty in the reconstruction of tumour phylogenies. As previously described^15^, our method also considered subclonal copy number changes in single-tumour samples. In consideration of copy number status and the observed allele frequency, the number of mutated copies was estimated and the CCF of the mutation determined. Somatic mutations that were found as clonal and that were the subject of subclonal copy number changes within single samples were filtered out.

In addition, we used the mapping qualities of the aligner (bwa mem, v.0.7.13-r1126) to filter out mutations in regions where more than 10% of uniquely mapped reads had a mapping quality below 10 (that is, less than 90% probability of having identified the correct mapping position).

With regard to potentially shared mutations, we also performed a power analysis to compare the CCFs of a given mutation between two samples with regard to their sequencing depth: we calculated a score per sample to consider the contribution of a single mutated read to the CCF. Per sample, the distribution of these scores could be estimated by a log-normal distribution whose 2.5% tails (*z*-score = 1.96) were cut off to filter out subsets of over- and underpowered mutations.

Last, to check further whether mutations observed as being private to one of the samples were truly private or simply not detected in the other sample (for example, due to insufficient coverage), we applied this statistical test: under the null hypothesis, the mutation is shared with an allelic fraction at least as high as that observed in one of the samples, and the probability (*P* value) of not detecting it within the given number of sequencing reads can be estimated using a binomial model. If the null hypothesis is rejected, the mutation is considered as being truly private, or otherwise is being filtered out. To determine those mutations that are rejected we apply the false discovery rate control at 5% by Benjamini–Hochberg correction.

Subsequent two-dimensional cluster analyses were performed with the set of mutations that passed all filters. This set was binned into a two-dimensional histogram of CCFs representing the observed data, which were modelled as a surface using two-dimensional smoothing splines with a common smoothing parameter. Based on an error estimate of the samples’ CCFs, this method deconvolutes part of the sequencing noise from the data. Subsequently the peaks of the surface were identified and interpreted as cluster centres (marked as red triangles in the cluster images for each patient; Supplementary Appendix), and all mutations were assigned to their nearest cluster centre by Euclidean distance. During the assignment procedure we require that shared mutations are assigned only to shared clusters whereas private mutations (that is, those exclusively called in one of the two samples) are assigned only to private clusters. Moreover, we set a minimum threshold of four mutations per cluster and disregarded identified surface peaks otherwise. Considering the cluster centre’s CCF as being representative of the corresponding cell clone, we applied the infinite sites hypothesis assuming that mutations appear once in the evolutionary history, and then determined the CCF sum rule^46,47^ to infer the most probable phylogenetic tree and, in particular, clonal composition per sample at the time point when sampling was derived. In the rare event that tumour phylogenetic rules allow for multiple solutions of tumour phylogeny, we assume maximum parsimony and prefer linear evolution over branched evolution within one sample.

In the case of CCF clusters that conflicted with phylogenetic rules we reanalysed somatic mutation calls initially computed with expected allele frequencies under the assumption of clonality. However, chromosomal segments with polyploidy allow for multiple values of absolute numbers of mutated copies (the so-called mutation multiplicity of each mutation call^15,48^). We therefore accounted for all potential solutions for mutation multiplicity of a given somatic mutation call and computed CCFs that rejected the assumption of clonality (null hypothesis) within the sample and which, in subsequent paired two-dimensional cluster analyses, resolved conflicts in phylogenetic tumour clone reconstruction.

### Analysis of tumour phylogenies

Our approach thus enabled us to assign tumour phylogenies for all 65 patients, and to track individual clones from multiregional and longitudinal data. We assigned mutations to the most recent common ancestor (C0) if they were shared and found to be clonal across all tumour sites sampled (that is, having CCFs of approximately 1.0). Alterations with lower CCFs, or those found to be private to single-tumour sites, were determined as subclones. Clusters of at least *n* = 5 subclonal mutations were defined and labelled as subclone C1, C2 or C3, and derivates of these subclones were assigned accordingly (Fig. 2a). The resulting tumour phylogenies for all 65 patients are provided in the Supplementary Appendix, detailing all spatially and temporally distinct sites analysed and depicting the clinical treatment history for each patient. Additional information is provided in Supplementary Table 4.

To study patterns of tumour evolution we assigned tumour phylogenetic trees to the following classes (Fig. 2a): class A if no subclones were identified; class B if one subclone was identified, allowing only for linear evolvement of this subclone; class B if at least two subclones were found with linear phylogenies; class D, phylogenies with one branching event from C1 subclones; class E, phylogenies with one branching event from the most recent common ancestor clone C0; and class F, tumour phylogenies showing two or more branching events.

In this regard, increasing the number of tumour samples per patient will enhance the ability to determine subclonal mutations and subclones^16^. Because we analysed various numbers of samples for each patient (in 37% of cases, more than two samples per patient) we additionally downscaled our analyses to only two samples per patient to permit interpatient comparisons (Fig. 2d); we thus performed a total of *n* = 84 paired analyses (Fig. 1c and Supplementary Table 4). In the paired analysis for each patient we chose as representative the analysis showing the highest level of subclonal complexity, defined by the number of subclones and subclonal mutations identified. Downscaling the number of tumour samples per patient did not show any significant change in the absolute number of subclonal mutations but led to reduced numbers of assigned subclones with phylogenetic complexity of classes A–E only (Extended Data Fig. 1a,b). Downscaling the analysis to two samples per patient for interpatient comparison enabled the study of distinct scenarios throughout the clinical course of the patients (Fig. 1b). To study the full complexity of a patient’s tumour, all available samples were taken into consideration (Supplementary Appendix).

### Analysis of cancer cell fractions for structural rearrangements

The analysis of the clonal architecture from multiregional and longitudinal tumour samples focused on the study of CCFs assigned to SNMs. In addition, to assess the clonality of structural rearrangement we applied SVclone (with default settings) to the whole-genome sequencing data of cases harbouring genomic rearrangements in key genes including *RB1*, *TP53*, *TP73* and *CREBBP*/*EP300*. We first performed local remapping to the human genome for genomic rearrangements identified by our in-house pipeline^42^ and assigned CCFs for both chromosomal pairs of a given rearrangement with SVclone^40^ (Supplementary Table 7). The data are presented in Extended Data Fig. 5c; the gene alterations identified were found to be part of the clonal proportion of the respective sample.

### Analysis of mutational signatures

We analysed our data for the activity of mutational signatures available in COSMIC, referring to SBS (COSMIC_v3.3_SBS_GRChr37_exome^17^).

Mutational signatures were analysed for the following categories: (1) the clonal proportion of all treatment-naive tumours, (2) the subclonal proportion of all treatment-naive tumours and (3) the subclonal proportion of all post-treatment tumours acquired following first-line platinum-based chemotherapy (Fig. 3a). The analysis of treatment-naive tumours refers to all naive samples available in this cohort (*n* = 58); signatures assigned to post-treatment tumours included all patients who received first-line platinum-based chemotherapy (*n* = 45), and we further distinguished whether tumour sites were exposed to chemotherapy alone (*n* = 20) or were potentially exposed to additional ionizing radiation (*n* = 25; Supplementary Table 2). Due to the high tumour mutational burden, signature assignments to clonal mutations were performed in cases with a median of over 300 mutations. To avoid overfitting and noise, assignments for subclonal mutations were performed only for cases with at least *n* > 20 mutations.

To fit mutational signatures to our samples we applied SigProfilerAssignment (that is, Analyze.cosmic_fit function^17,49^) to identify a representative subset of signatures. We initially fitted SBS mutational signatures to the mutation catalogue of each sample assigned to the categories. Selecting mutational signatures found in at least *n* = 5 cases, we thus identified the most prevalent subset of signatures in the clonal and subclonal proportions of treatment-naive and post-treatment tumours (SBS1, SBS2, SBS3, SBS4, SBS5, SBS13, SBS15, SBS16, SBS24, SBS29, SBS39, SBS40 and SBS92), to which all mutations were then fitted. Post-treatment samples additionally showed platinum-based signatures (SBS31 and SBS35), which were therefore included for the assignment of signatures for the subclonal proportion of post-treatment tumours. In addition we applied the in-house-developed computational tool CaMuS^50^ to confirm signature assignments. With CaMuS we first linearly fitted the COSMIC signatures to all mutations for each sample (including clonal and subclonal mutations) using a backward selection procedure. We next selected only those signatures that markedly reduced the cost of the model calculated over the whole dataset. Both tools generated similar results. The results of SigProfilerAssignment are provided in Fig. 3 and Extended Data Figs. 2 and 3. Comparisons with CaMuS are provided in Extended Data Fig. 2h and the data are summarized in Supplementary Table 5.

To track the dynamic activity of mutational signatures in patient-matched tumour samples over the course of the disease, we specifically assigned the subset of signatures identified with SigProfilerAssignment to patient-matched clonal and subclonal mutations pre- and post-treatment, including SBS31 and SBS35 (both related to platinum chemotherapy treatment) for all assignments of signatures. We thus confirmed the presence of platinum-based signatures only in post-treatment subclonal mutations of tumour samples but not in the patient-matched treatment-naive clonal or subclonal proportion of the tumour. In addition we analysed tumour samples from a cohort of patients undergoing subsequent second- or third-line treatment with immune checkpoint inhibition (*n* = 7). Tumour samples acquired before treatment with ICI were analysed in the categories above (corresponding to samples acquired at the time of relapse following first-line platinum-based chemotherapy). Samples pre- and post-treatment with ICI were analysed with the subset described above (Supplementary Table 5).

We furthermore tested our whole-genome and whole-exome sequencing data for mutational processes related to ionizing radiation. Following previous studies in this field^25^, we determined the ratio of insertions to deletions (indels) versus substitution burden and the ratio of deletions versus insertions based on exome- and genome-wide data (Extended Data Fig. 2f).

### Analysis of significant mutations, copy number alterations and genome ploidy

To assess the relevance of key gene alterations in our cohort we referred to our previous study of significant gene alterations determined for 110 human SCLC samples^5^ (Supplementary Table 8). In addition we expanded this analysis to our present cohort of 65 patients. We determined the mutational landscape for each patient by creating the union of all mutations identified in multiple samples—this refers to the sum of mutually inclusive and private events (Supplementary Table 6). We combined the data from our current cohort of 65 patients with mutational data for 110 human SCLC samples^5^ (*n* = 175 patients) and determined significant gene alterations at a significance threshold of *Q* < 0.05 following our previously described method^5^. In brief, our approach estimates the background mutation rate for each gene and corrects for both synonymous mutations and the expression in human SCLC, referring to the transcriptional data of human SCLC^5^. The analysis included genes with fragments per kilobase exon per million mapped reads values of over 1 in at least 50 samples. Furthermore we analysed the data for significant mutational hotspots and significant enrichment of gene-damaging mutations. Mutations that significantly cluster within a gene were determined at *Q* < 0.05 (mutational hotspots). The analysis of gene-damaging mutations refers to (1) nonsense mutations resulting in early stop codons, (2) splice site mutations resulting in aberrant splicing, intron retention or in-frame losses of larger regions within the protein product and (3) frameshift mutations leading to early stop codons and thus resulting in greater changes in the gene and encoded transcript, presumably leading to either no protein product, to proteins with larger deletions within the protein structure or to truncated proteins. The enrichment of gene-damaging alterations was determined at *Q* < 0.05. We focused our studies on genes recurrently mutated in at least 8% of cases (affecting at least *n* = 14 patients in the combined analysis of this cohort and the previous cohort^5^); this allowed us to perform interpatient comparisons and to study a sufficient number of cases in our present cohort of *n* = 65 patients. To complement our analytical approach we also used other computational tools to study significant gene alterations, including MutSig2CV^51^, dNdSCV^52^ and OncodriveFML^53^. In brief, MutSig2CV and dNdSCV were run using their default configuration; for OncodriveFML we used the ‘complement’ method for the signature and ‘amean’ as statistics. Taking into account different levels of stringency, all computational models showed a high degree of overlap. All relevant and significant gene alterations are listed in Supplementary Table 8. In addition we studied gene alterations previously reported for targeted sequencing data from larger cohorts of patients with SCLC^4^; we scored the frequency and significance level of reported alterations for the samples in our cohort. Comparison of these data is provided in Extended Data Fig. 4e and Supplementary Table 8.

With regard to frequent alterations affecting *TP53*, *RB1* and *TP73* (Supplementary Table 8), which also included larger genomic rearrangements of these genes (Supplementary Table 7), we further analysed the gene-damaging effect of alterations. The impact of any genome alterations was evaluated in combination with the transcriptome sequencing data of these tumours, thus further informing on the presumed damage to the gene transcript and resulting protein product (Supplementary Table 11).

Significant copy number alterations were determined from uncorrected unsegmented copy number signals obtained from whole-exome sequencing data by applying the method CGARS^54^. We determined the analysis separately for pre- and post-treatment tumour samples, referring to one sample per patient case in both scenarios. Significant amplifications were determined with the upper quantiles 0.30, 0.10 and 0.05; deletions were computed in reference to lower quartiles 0.30, 0.15 and 0.05. Significance threshold was set at *Q* = 0.05. Significant copy number alterations are listed in Supplementary Table 9.

Overall genome ploidy was assigned for all patient tumours (Supplementary Table 3), with a threshold of 2.8 or above set to define those with higher genome ploidy^33^. Higher ploidy in cancer genomes can result either from multiple successive and independent copy number gains or through events of whole-genome doubling. To further determine events of genome duplication (or whole-genome doubling), tumours found to undergo ploidy changes were further analysed for the fraction of the genome with LOH to assign an event of genome doubling^45^ (Extended Data Fig. 5g,h).

### Clinical correlations with chemotherapy relapse-free survival

We studied correlations of genomic subsets with relapse-free survival in patients receiving first-line systemic treatment with platinum-based chemotherapy. The analysis focused on the study of *n* = 55 patients for whom the clinical response to first-line platinum-based chemotherapy was determined. Ten patients from our cohort were not considered for this analysis because of either loss to follow-up (*n* = 2), declined further treatment and no longer in clinical care (*n* = 1) or received a lung resection resulting in longer disease-free survival and differences in the dynamics of disease progression (*n* = 7). We determined relapse-free survival by referring to CTFI, defined as the time between the end of chemotherapy and tumour recurrence, including for patients with disease progression resulting in death. Two patients in our cohort were reported with sepsis-related mortality and were censored in the analysis for recurrence-free survival, leaving a final total of *n* = 53 patients. All survival analyses were performed with SPSS. Survival distributions were plotted as Kaplan–Meier curves, with *P* values determined by log-rank test (Extended Data Fig. 8a). Hazard ratios with a 95% confidence interval and *P* values were further derived from Cox proportional hazard models. We performed correlations with key genomic parameters referring to significant gene mutations identified in Extended Data Fig. 4 and, in addition, we stratified patients according to genome ploidy (information available for *n* = 53 patients). We included in our analysis as clinical characteristics information on sex, age and tumour stage. We performed additional analyses on both smoking status and pack years of patients (available for *n* = 50 and *n* = 47 patients, respectively). Furthermore we included in our analyses the gene expression of key lineage transcription factors *ASCL1*, *NEUROD1* and *POU2F3* (available for *n* = 45 patients).

We checked that the assumption of proportional hazards was provided by log-minus-log survival plots and by the addition of time-dependent covariates to models. We performed multicollinearity assessment of predictors. We identified relevant gene alterations by performing regressions with backward elimination of insignificant predictors (backwards Wald, at a retention threshold of *P* < 0.05). The results of the Cox proportional hazard model are shown as forest plots.

Clinical correlations of genomic alterations with relapse-free survival were additionally analysed in an independent cohort of patients with SCLC (*n* = 64) who all received first-line systemic treatment with platinum-based chemotherapy. Note that we used WES and WGS to determine the full spectrum of alterations in key genes in our discovery cohort. By contrast, data for the independent cohort refer to WES data, which limits the detection of complex gene rearrangements that frequently affect *CREBBP*, *EP300*, *TP73* and, to some extent, *TP53* (ref. ^5^) (Extended Data Fig. 4a). The somatic alteration status for *TP53*, *TP73*, *CREBBP*, *EP300* and *FMN2* as determined by WES is provided in Supplementary Table 12.

### Immunoblot analysis

Immunoblots were performed to probe tumour cell lysates for the expression of p53 (Extended Data Fig. 9a). Tissue samples from this cohort containing sufficient material were processed to 5 μm sections on a cryostat maintained at −20 °C. The non-SCLC cell line A549 served as control for the expression of wild-type p53 (ref. ^55^); we confirmed the identity of this cell line by STR profiling and performed tests to ensure no contamination with mycoplasma. Between 40 and 50 tissue sections per sample were sonicated for 3× 10 min and incubated for an additional 30 min in RIPA buffer supplemented with protease inhibitors (cOmplete Mini Protease Inhibitor Cocktail, Roche) and nuclease (benzonase, Millipore) at 4 °C. A549 cells were incubated in RIPA for 30 min at 4 °C. Supernatants were collected following centrifugation at 4 °C for 10 min at 20,000*g* and protein concentrations determined by bicinchoninic acid assay (Pierce). Either 15 μg (tissue samples) or 90 μg (A549) of protein in 3× Laemmli buffer was separated on 4–12% Tris-glycine SDS–polyacrylamide gel electrophoresis gels (Thermo Fisher Scientific) and transferred to polyvinylidene difluoride membranes (Millipore). PageRuler 10–180 kDa (Thermo Scientific) served as the protein ladder for size determination. Membranes were blocked with Tris buffered saline with 5% milk powder for 1 h at room temperature and incubated overnight with a 1:1,000 dilution of anti-p53 (clone D07, mouse monoclonal antibody, abcam, no. ab80644) and anti-HSP90 (clone C45G5, rabbit monoclonal antibody, Cell Signaling, no. 4877) at 4 °C, washed in Tris buffered saline with Tween 20 and incubated for 1 h with a 1:10,000 dilution of fluorescence-labelled secondary anti-mouse (IRDye 800CW goat anti-mouse, LI-COR, no. 926-32210) and anti-rabbit (IRDye 800CW goat anti-rabbit, LI-COR, no. 926-32211) antibodies. Blots were analysed with the Odyssey CLx imaging system (LI-COR).

### Reporting summary

Further information on research design is available in the Nature Portfolio Reporting Summary linked to this article.

## Online content

Any methods, additional references, Nature Portfolio reporting summaries, source data, extended data, supplementary information, acknowledgements, peer review information; details of author contributions and competing interests; and statements of data and code availability are available at 10.1038/s41586-024-07177-7.

### Supplementary information

Supplementary Information Clinical course and tumour phylogeny determined for 65 patients with SCLC.

Reporting Summary

Supplementary Fig. 1 Uncropped immunoblots from Extended Data Fig. 10a.

Supplementary Tables Supplementary Tables 1–12.

## Figures and Tables

**Extended Data Table 1:**
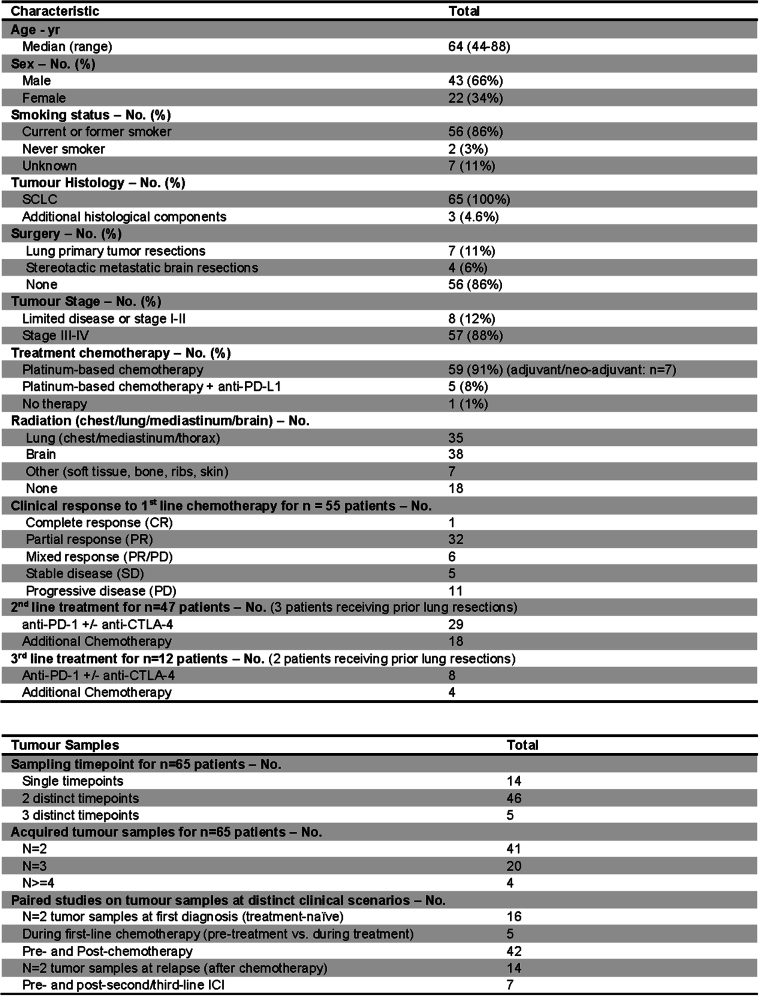
Information on clinical characteristics and tumour samples acquired from 65 patients with SCLC

Upper panel, overview on the clinical information for 65 patients with SCLC. Additional information is provided in Supplementary Table 1.

Lower panel, overview on multi-regional tumour sites acquired from 65 patients with SCLC. The number of tumour samples acquired at distinct timepoints throughout the clinical course of treatment is indicated. Paired tumour analyses were performed focusing on distinct clinical scenarios to thus permit interpatient comparisons (compare Fig. 1b, Supplementary Table 2).

## Data Availability

The raw sequencing data are deposited in the European Genome-Phenome Archive under accession no. EGAS50000000169. Supporting data are provided as Supplementary Tables.

## Code availability

Computational approaches applied in this manuscript are described in Methods and were previously published^5,6,15,42,43^. Our genome data-processing workflow ‘peiflyne’, which we use to perform duplicate masking, mutation calling, mutation filtering and annotation, as well as the algorithm used to identify significant mutational drivers, are available at http://www.uni-koeln.de/med-fak/peiflyne/peiflyne.tgz.
