# Supplementary material for: Evolutionary trajectories of small cell lung cancer under therapy
Source: Nature. 2024 Mar 13;627(8005):880–9. doi: 10.1038/s41586-024-07177-7 (PMC10972747; doi:10.1038/s41586-024-07177-7)

nature portfolio

https://doi.org/10.1038/s41586-024-07177-7

Supplementary information

Evolutionary trajectories of small cell lung cancer under therapy

In the format provided by the authors and unedited

Supplementary Appendix – Clinical course and tumour phylogeny determined for 65 patients with SCLC.

Schematic depiction for each patient detailing tumour stage, clinically affected organ sites, treatments and the clinical response of the patient. Grey wedges describe the site from where tumour samples were acquired labelled as S1, S2, S3 etc.

Key genomic alterations were determined by either whole exome or whole genome sequencing. The Cancer Cell Fraction (CCF) of each mutation was determined for all tumour samples by whole exome sequencing. 2-dimensional cluster analyses were performed and is provided for each paired tumour analysis. Red triangles mark peaks of cluster centers and cluster of mutations were assigned to the common ancestral clone (C0) or to subclones following the color scheme and annotation provided in **Figure 2a**. Phylogenetic trees were reconstructed across multiple samples for each patient and the clonal composition at each tumour site is provided. The phylogenetic tree provides information for significantly mutated genes (**Supplementary Table 6, Extended Data Figure 4**) assigned to branches as part of the common ancestor, and if identified as subclonal mutations assigned to respective branches.

S02469 T4NxM1, stage IV

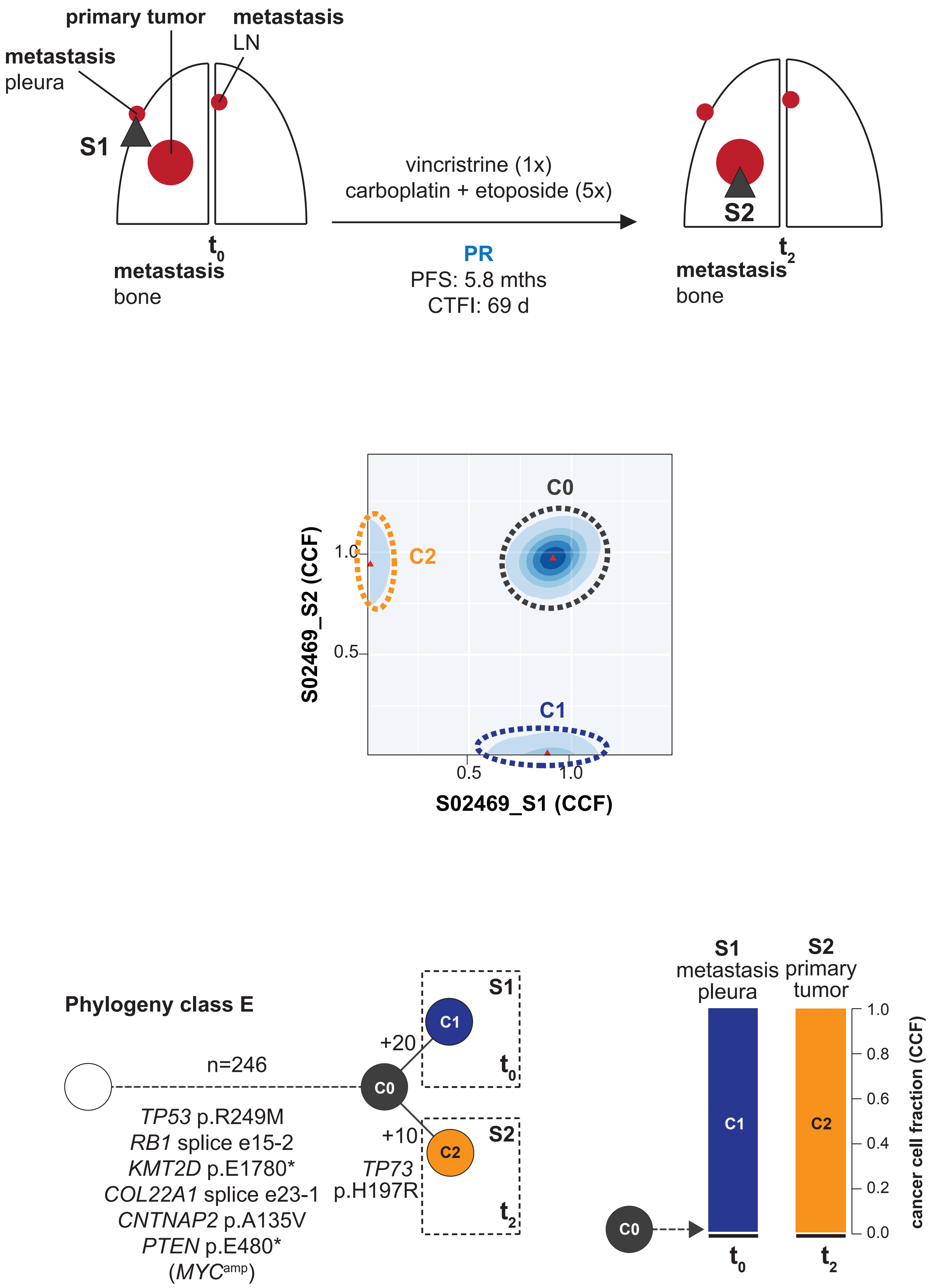

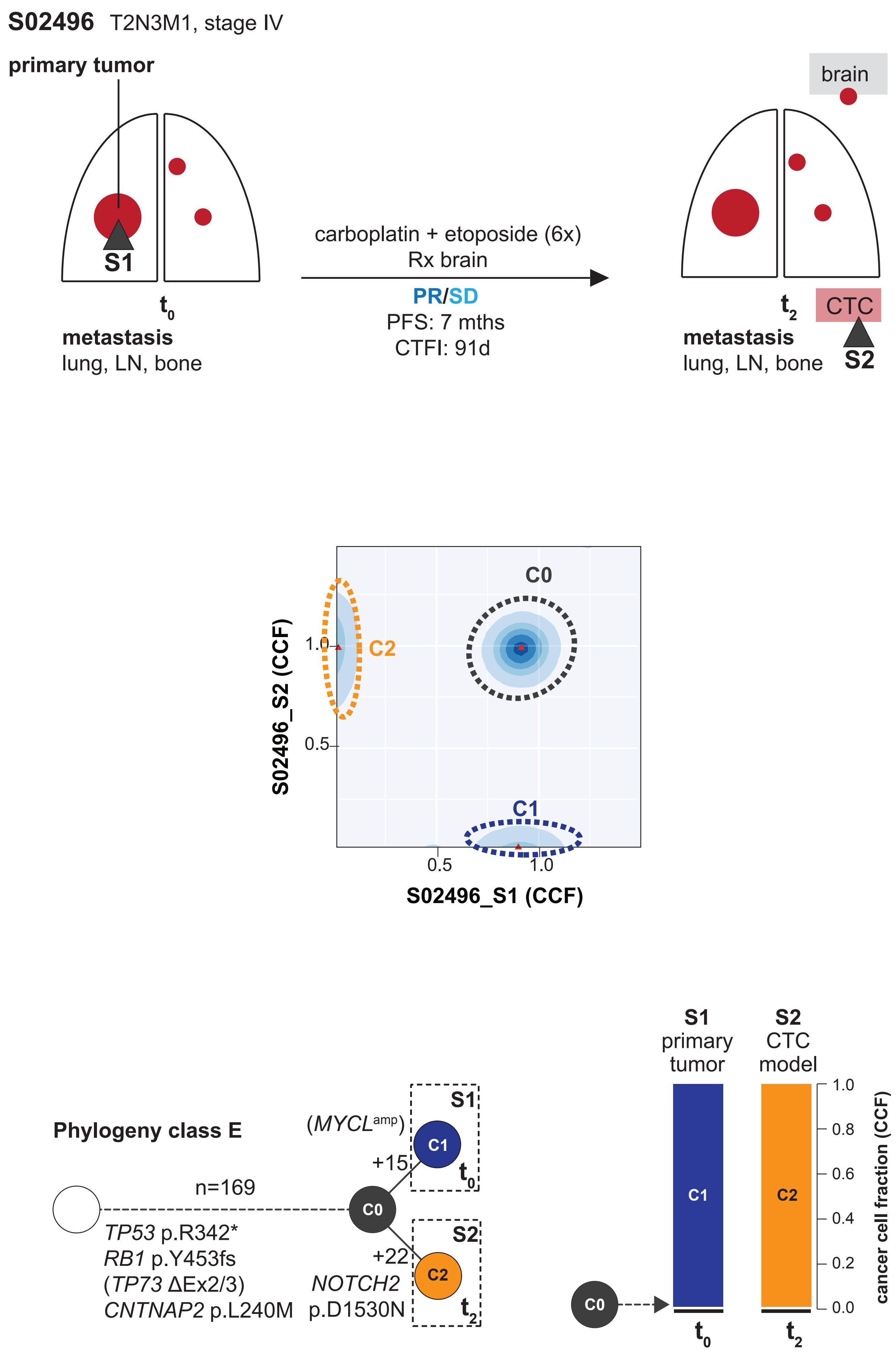

S02496 T2N3M1, stage IV
primary tumor
S1
t0
metastasis
lung, LN, bone
carboplatin + etoposide (6x)
Rx brain
PR/SD
PFS: 7 mths
CTFI: 91d
brain
t2
CTC
S2
metastasis
lung, LN, bone
C0
C2
C1
1.0
0.5
0.5
1.0
S02496_S2 (CCF)
S02496_S1 (CCF)
Phylogeny class E
n=169
TP53 p.R342*
RB1 p.Y453fs
(TP73 ΔEx2/3)
CNTNAP2 p.L240M
(MYCL amp)
+15
+22
NOTCH2
p.D1530N
C0
C1
C2
S1
S2
t0
t2
S1
primary
tumor
S2
CTC
model
C1
C2
C0
1.0
0.8
0.6
0.4
0.2
0.0
cancer cell fraction (CCF)
t0
t2

S02500 T3N3M1, stage IV

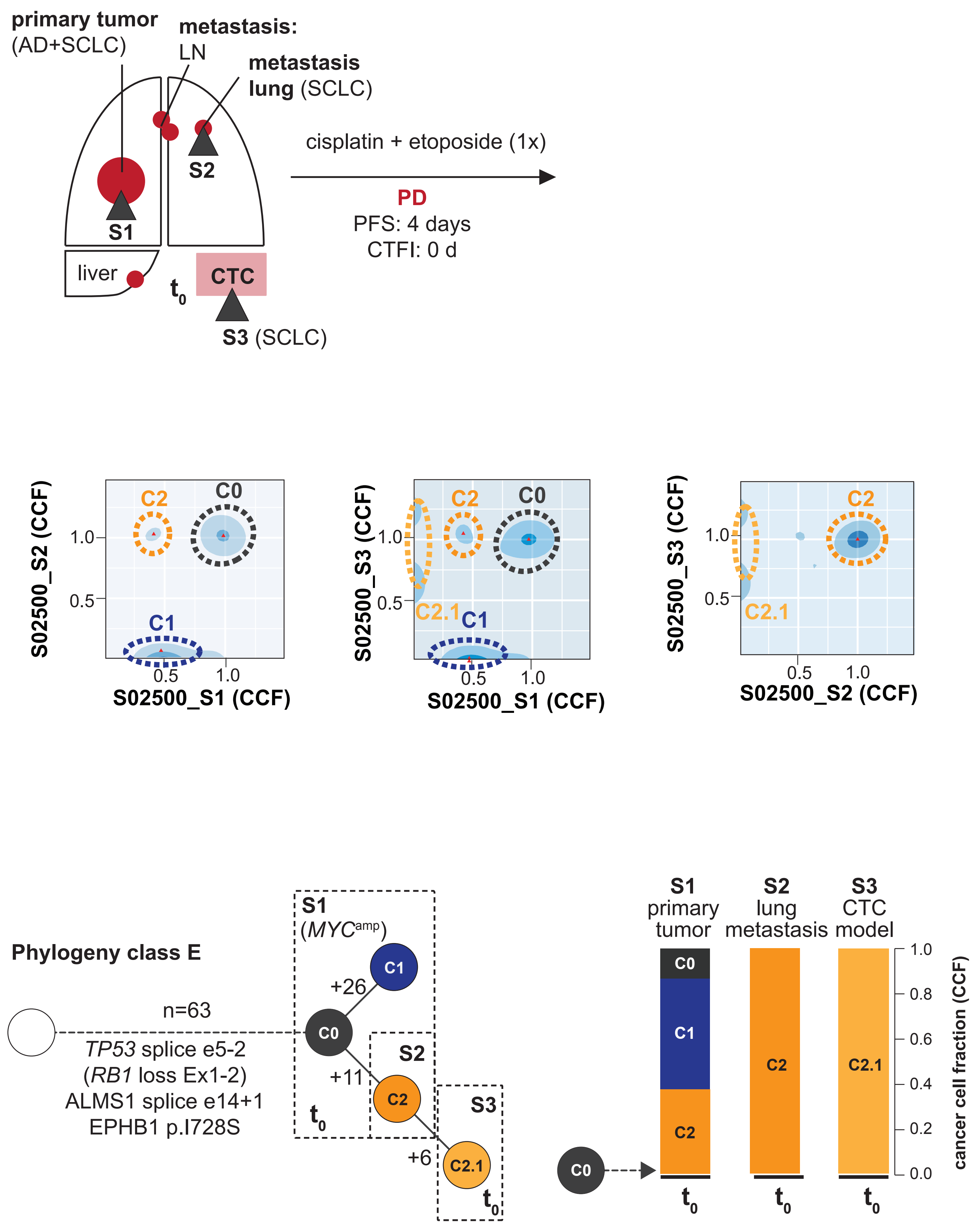

S02513 T4N2M1, stage IV

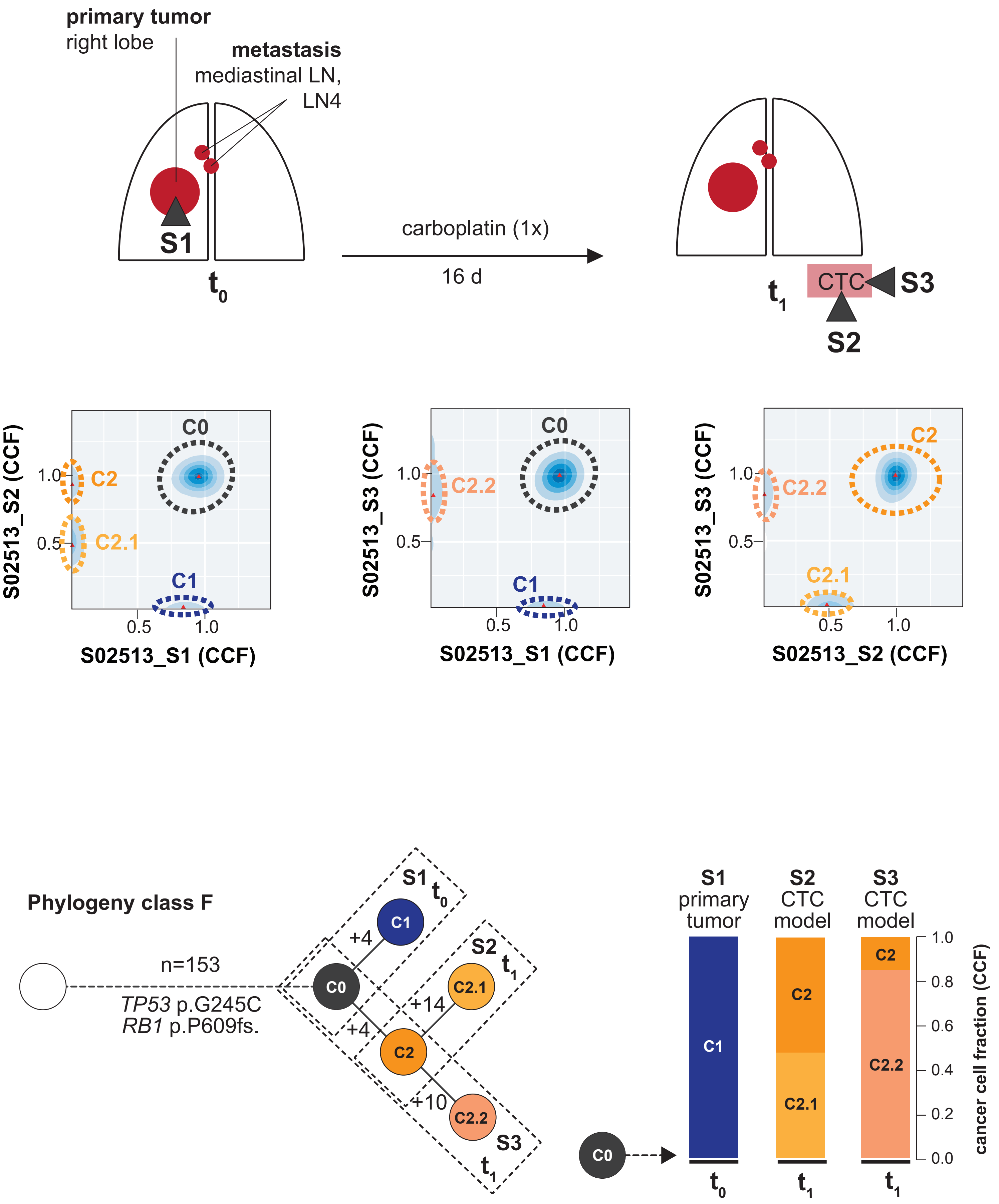

S02521 stage IV

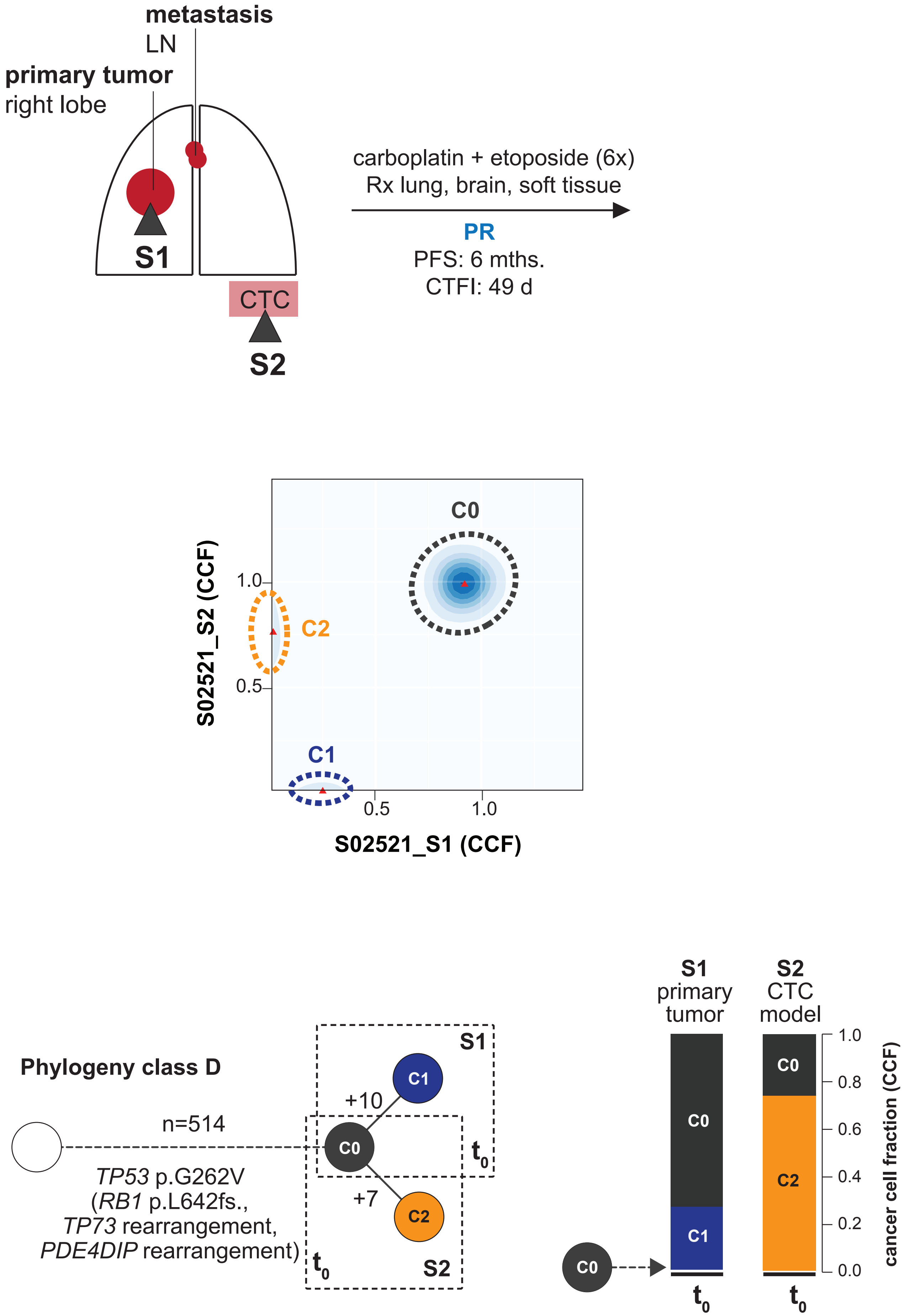

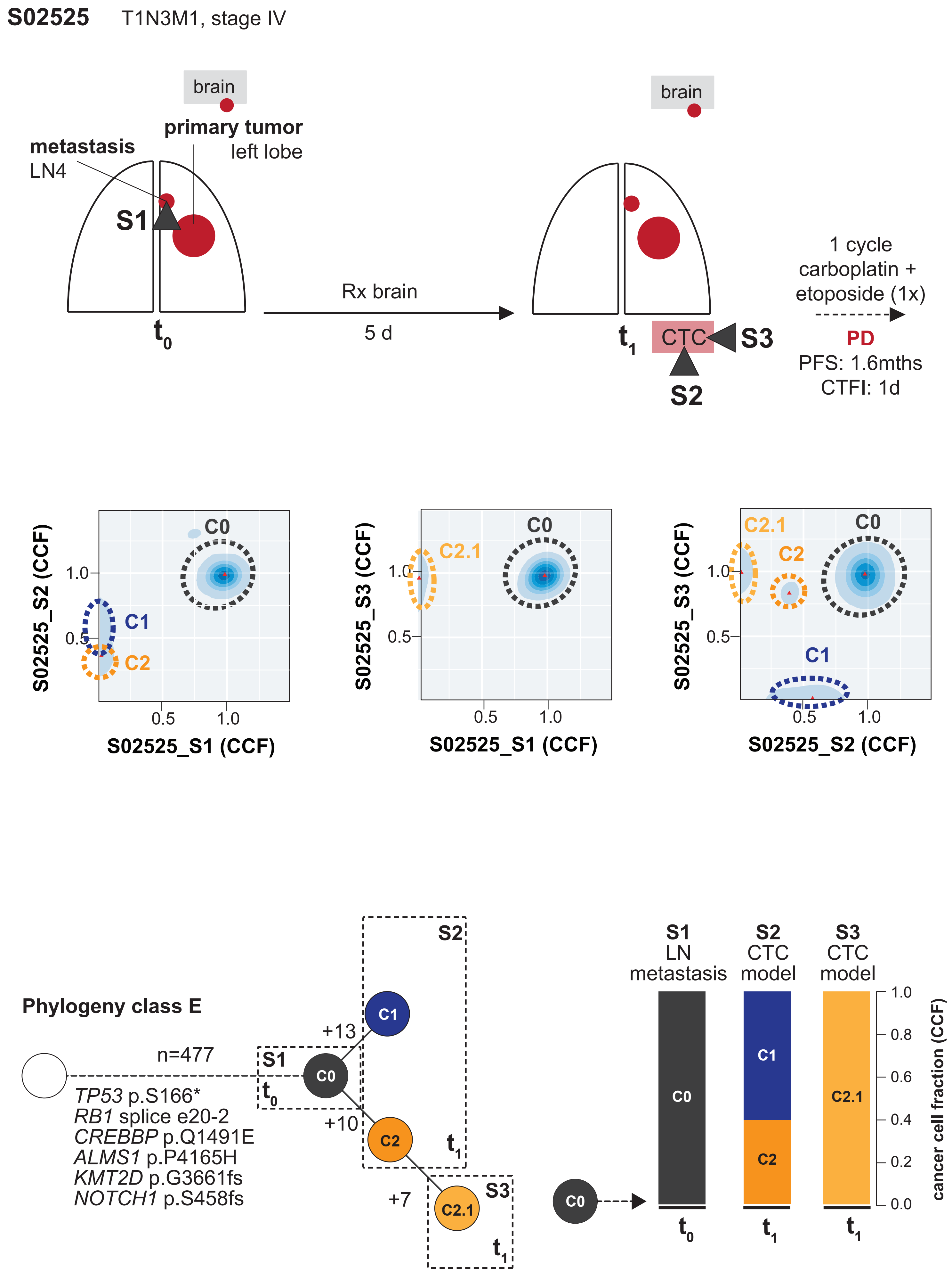
S02525
T1N3M1, stage IV
brain
primary tumor
left lobe
metastasis
LN4
S1
t_0
Rx brain
5 d
brain
t_1
CTC
S3
S2
1 cycle
carboplatin +
etoposide (1x)
PD
PFS: 1.6mths
CTFI: 1d
S02525_S2 (CCF)
S02525_S1 (CCF)
C0
C1
C2
S02525_S3 (CCF)
S02525_S1 (CCF)
C0
C2.1
S02525_S3 (CCF)
S02525_S2 (CCF)
C2.1
C0
C2
C1
Phylogeny class E
n=477
TP53 p.S166*
RB1 splice e20-2
CREBBP p.Q1491E
ALMS1 p.P4165H
KMT2D p.G3661fs
NOTCH1 p.S458fs
S1
t_0
C0
+13
C1
+10
C2
S2
t_1
+7
C2.1
S3
t_1
S1
LN
metastasis
S2
CTC
model
S3
CTC
model
C0
C1
C2
C2.1
t_0
t_1
t_1
cancer cell fraction (CCF)

S02544 stage IV

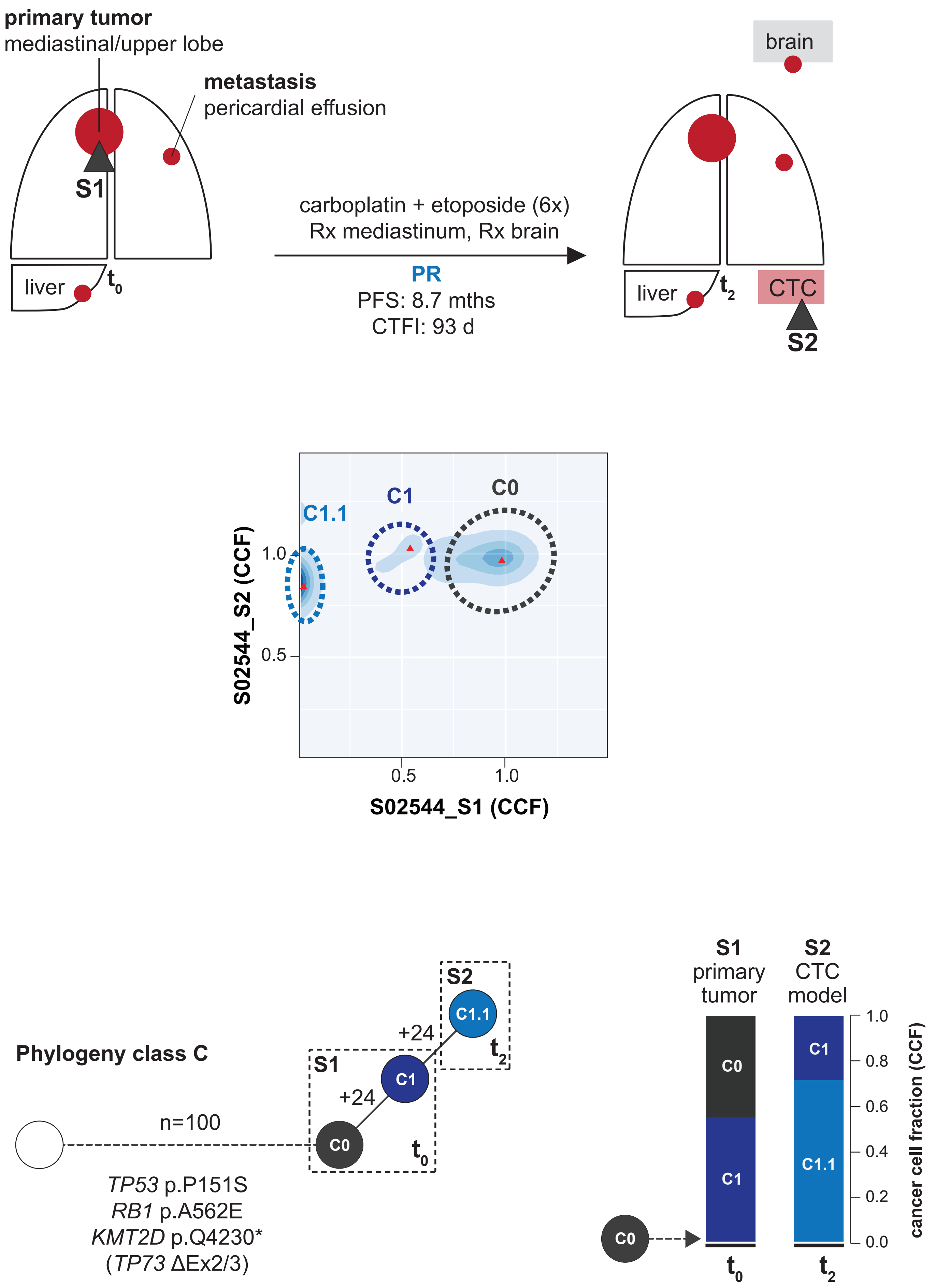

S02551 T4N3M1, stage IV

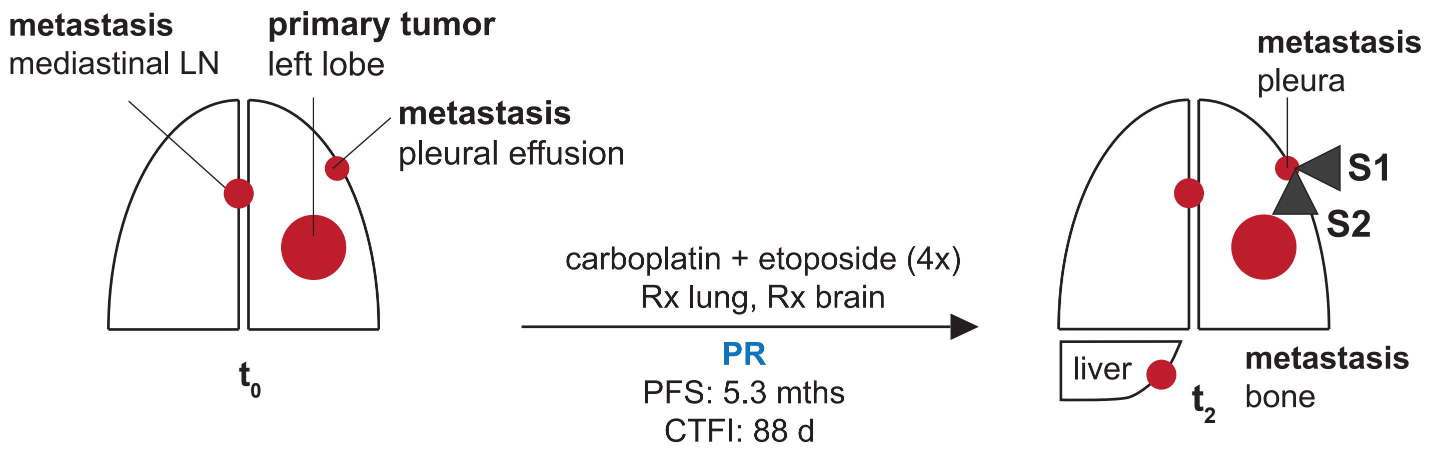

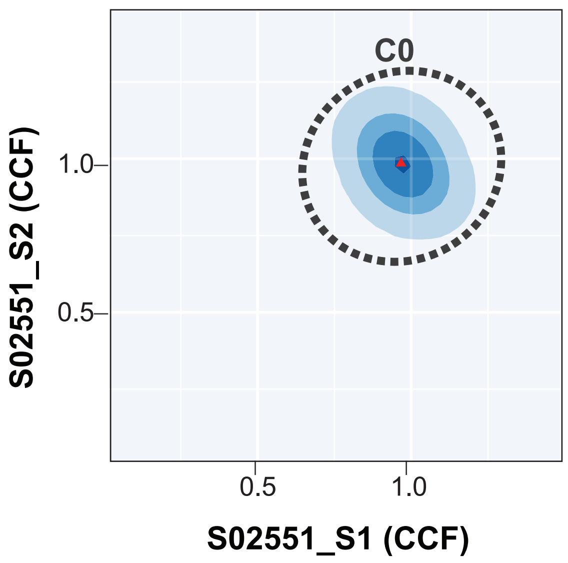

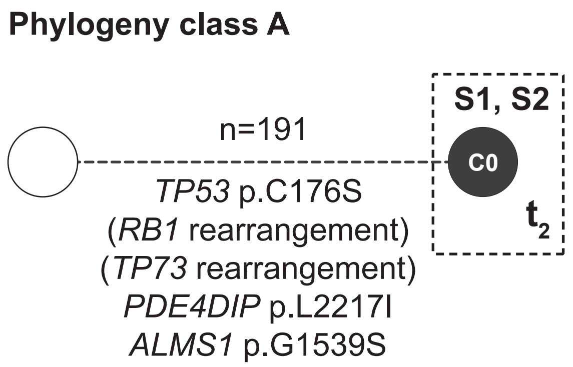

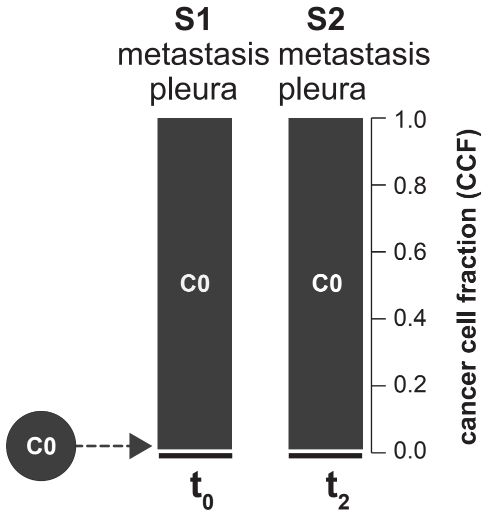

S02562 T4N3M1, stage IV

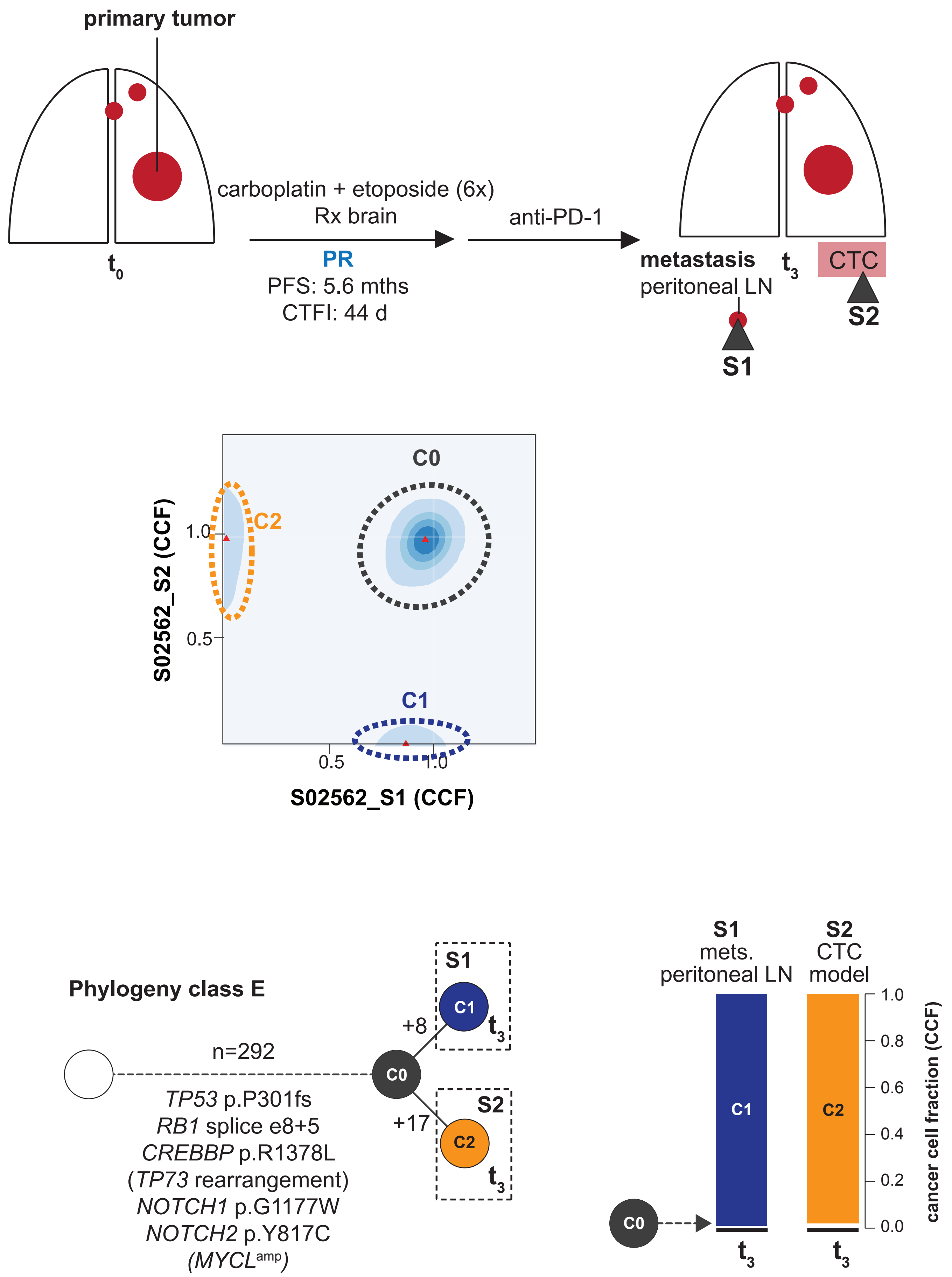

S02563 T4N3M1, stage IV

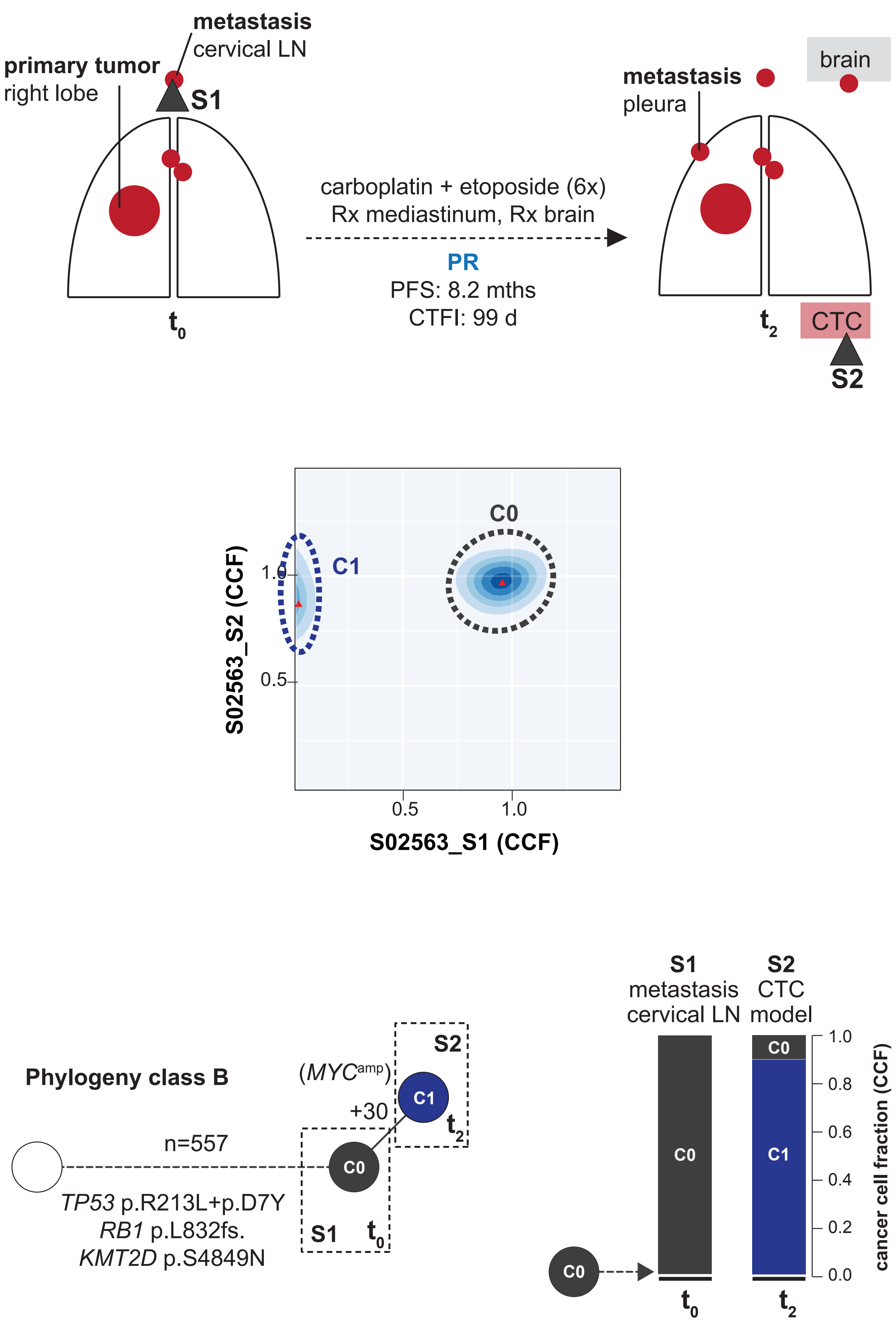

S02608 TXNXM1, stage IV

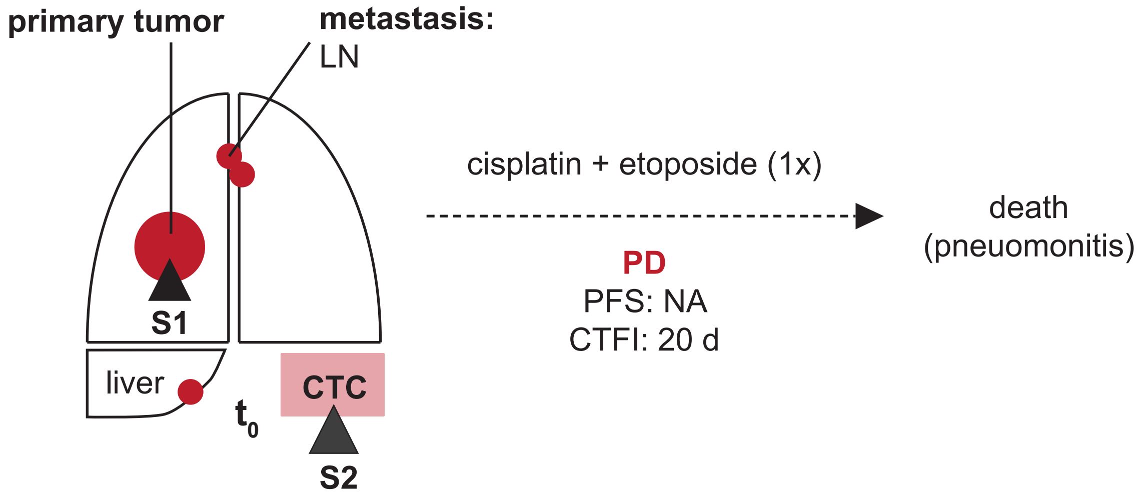

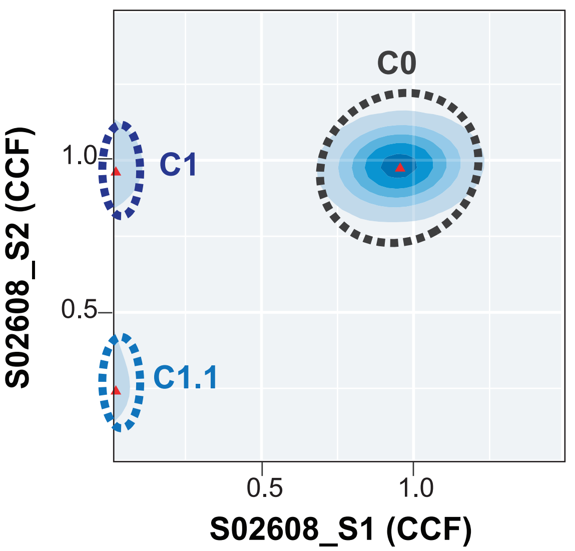

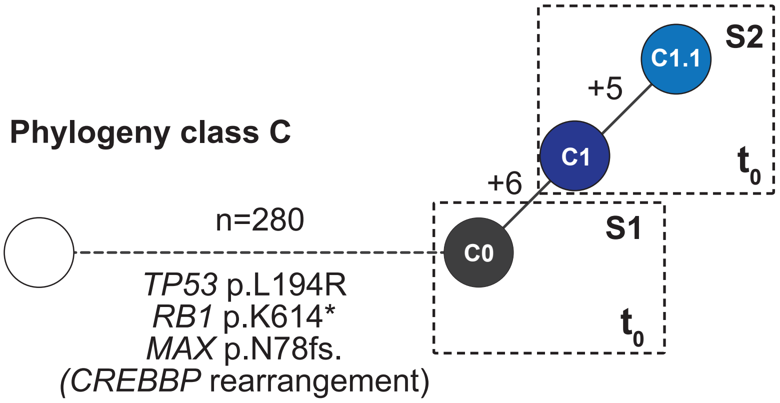

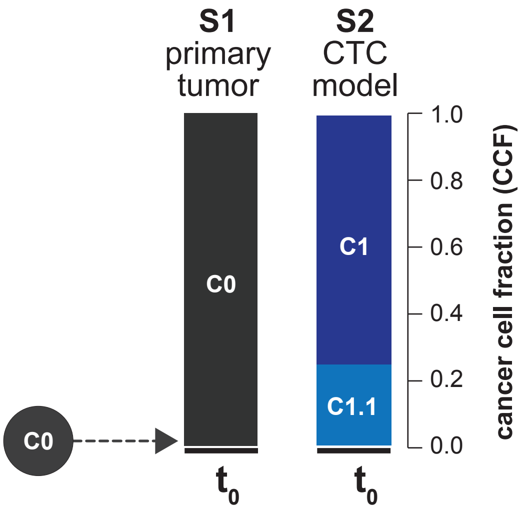

S02618 T4NXM1, stage IV

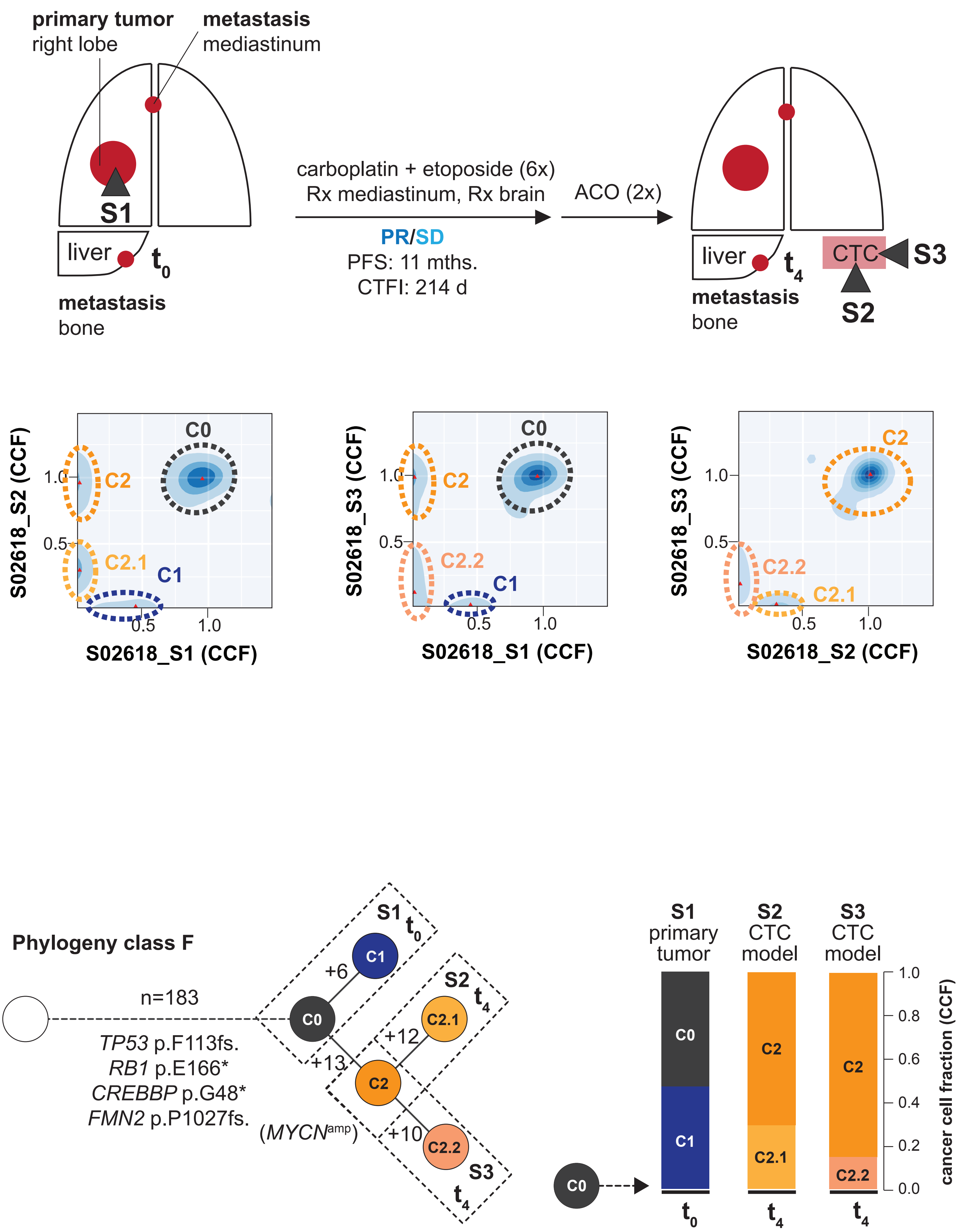

S02658 T4N3M1, stage IV

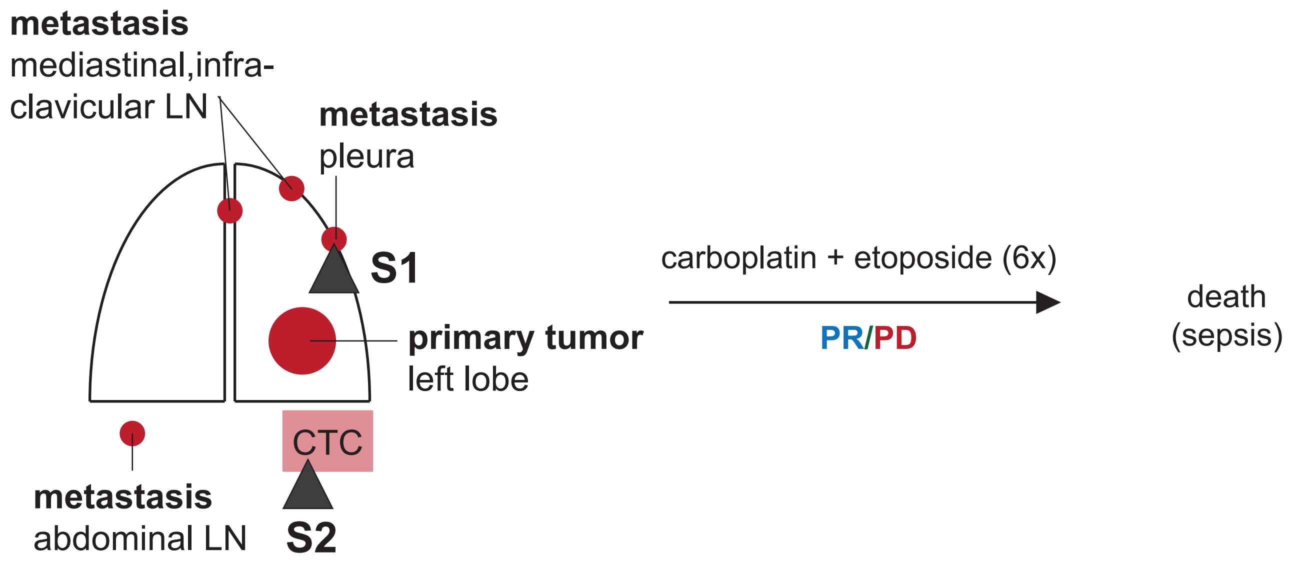

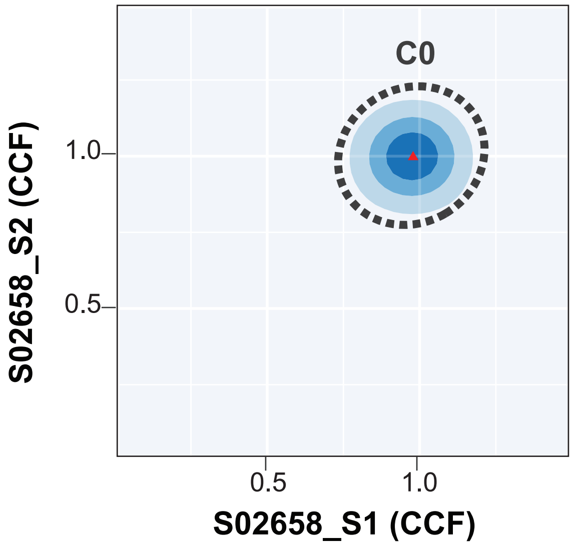

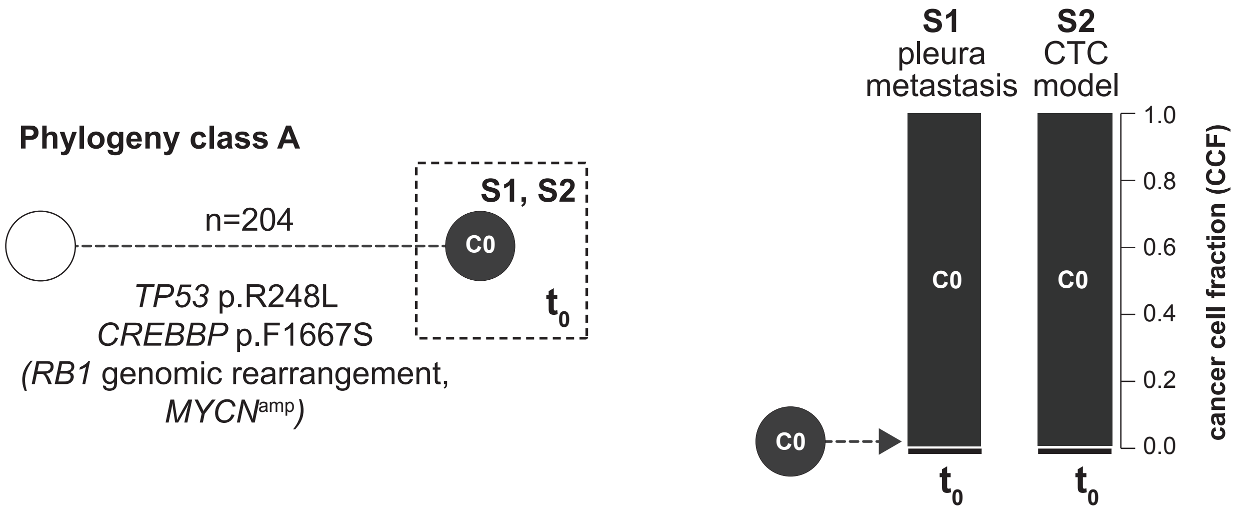

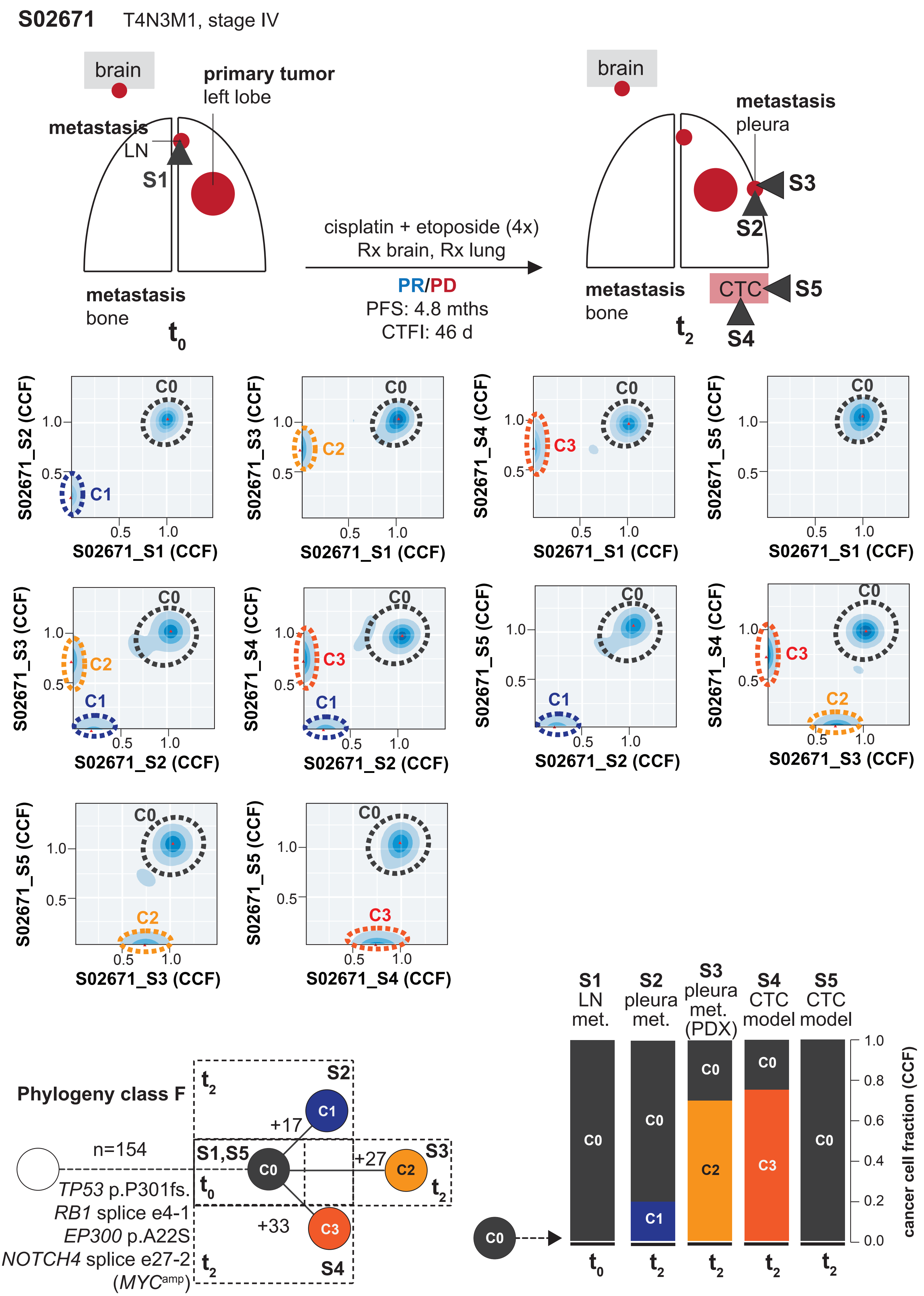
S02671 T4N3M1, stage IV
brain
primary tumor
left lobe
metastasis
LN
S1
metastasis
bone
t0
cisplatin + etoposide (4x)
Rx brain, Rx lung
PR/PD
PFS: 4.8 mths
CTFI: 46 d
brain
metastasis
pleura
S3
S2
CTC
S5
S4
metastasis
bone
t2
C0
C1
C2
C3
S02671_S1 (CCF)
S02671_S2 (CCF)
S02671_S3 (CCF)
S02671_S4 (CCF)
S02671_S5 (CCF)
Phylogeny class F
n=154
TP53 p.P301fs.
RB1 splice e4-1
EP300 p.A22S
NOTCH4 splice e27-2
(MYCamp)
S1,S5
t0
+17
+27
+33
S1 LN met.
S2 pleura met.
S3 pleura met. (PDX)
S4 CTC model
S5 CTC model
cancer cell fraction (CCF)

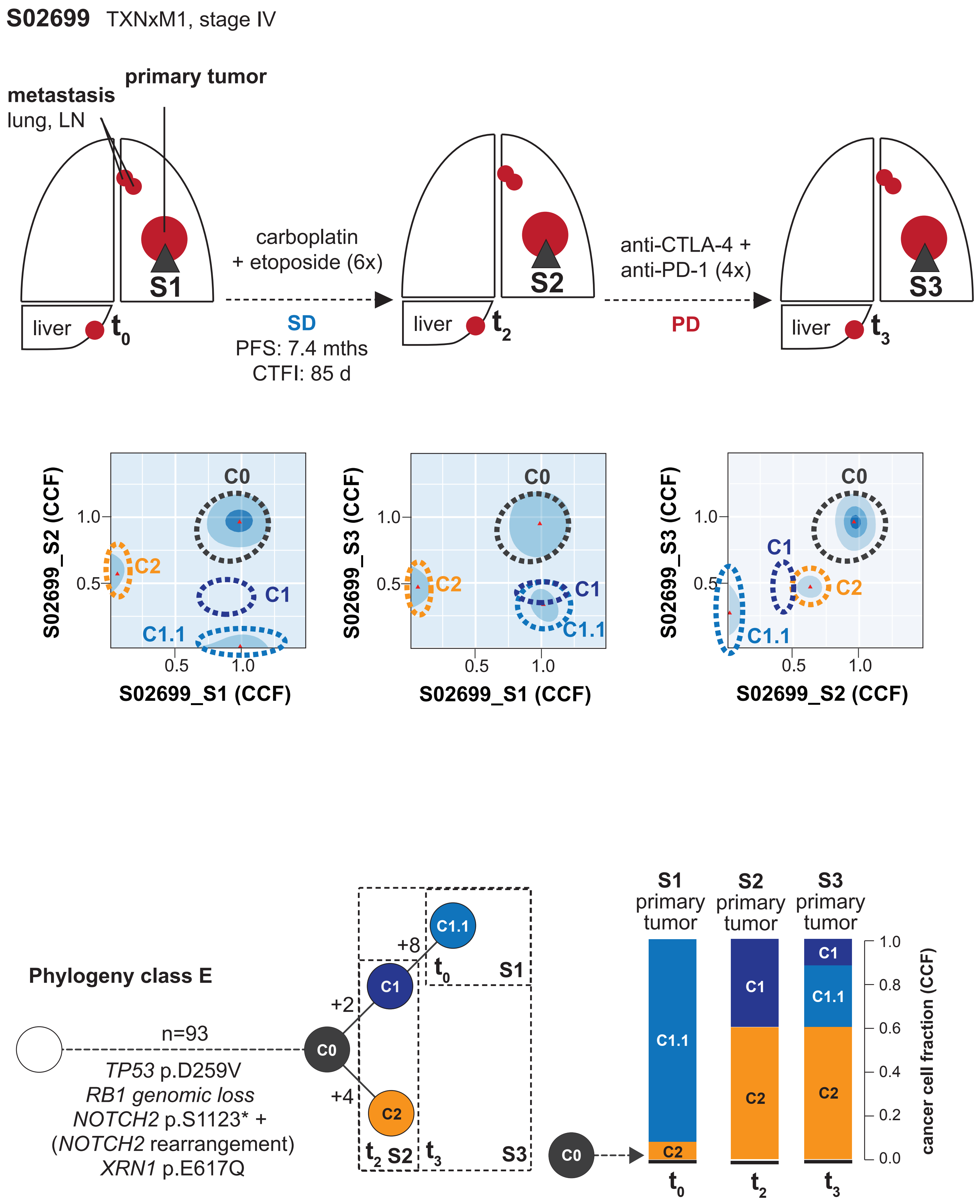
S02699 TXNxM1, stage IV
primary tumor
metastasis
lung, LN
S1
liver
t_0
carboplatin
+ etoposide (6x)
SD
PFS: 7.4 mths
CTFI: 85 d
S2
liver
t_2
anti-CTLA-4 +
anti-PD-1 (4x)
PD
S3
liver
t_3
S02699_S2 (CCF)
S02699_S1 (CCF)
C0
C2
C1
C1.1
S02699_S3 (CCF)
S02699_S1 (CCF)
C0
C2
C1
C1.1
S02699_S3 (CCF)
S02699_S2 (CCF)
C0
C1
C2
C1.1
Phylogeny class E
n=93
TP53 p.D259V
RB1 genomic loss
NOTCH2 p.S1123* +
(NOTCH2 rearrangement)
XRN1 p.E617Q
C0
+2
C1
+8
C1.1
+4
C2
t_0 S1
t_2 S2
t_3 S3
S1
primary
tumor
S2
primary
tumor
S3
primary
tumor
C1.1
C2
C1
C2
C1
C1.1
C2
C0
t_0
t_2
t_3
cancer cell fraction (CCF)

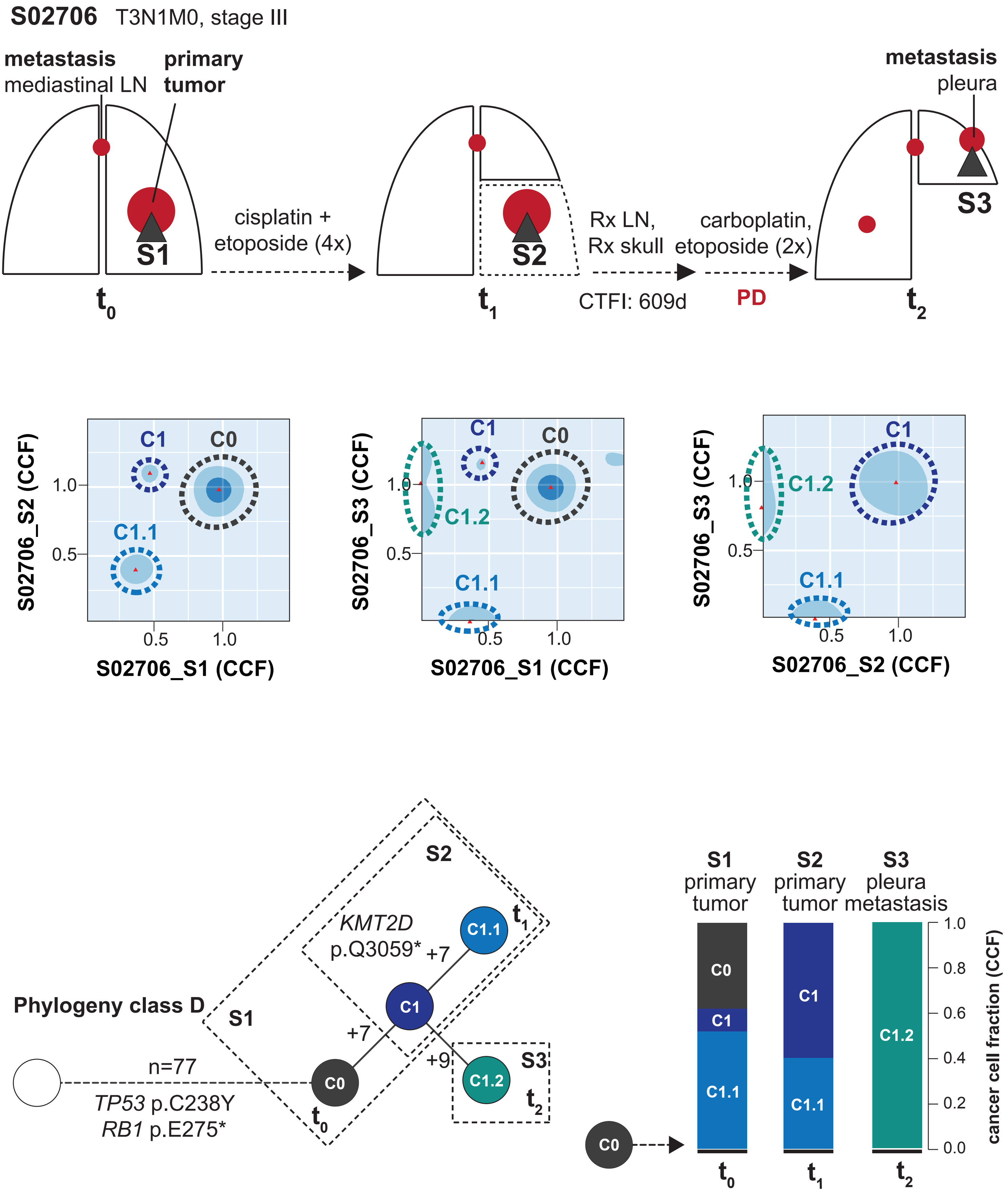

S02706 T3N1M0, stage III
metastasis
mediastinal LN
primary
tumor
S1
t0
cisplatin +
etoposide (4x)
S2
t1
Rx LN,
Rx skull
CTFI: 609d
carboplatin,
etoposide (2x)
PD
metastasis
pleura
S3
t2
C1
C0
C1.1
1.0
0.5
S02706_S2 (CCF)
S02706_S1 (CCF)
C1.2
S02706_S3 (CCF)
S02706_S2 (CCF)
Phylogeny class D
n=77
TP53 p.C238Y
RB1 p.E275*
+7
KMT2D
p.Q3059*
+7
+9
S1
S2
S3
C0
C1
C1.1
C1.2
t0
t1
t2
S1
primary
tumor
S2
primary
tumor
S3
pleura
metastasis
1.0
0.8
0.6
0.4
0.2
0.0
cancer cell fraction (CCF)

S02707 T2N2M1, stage IV

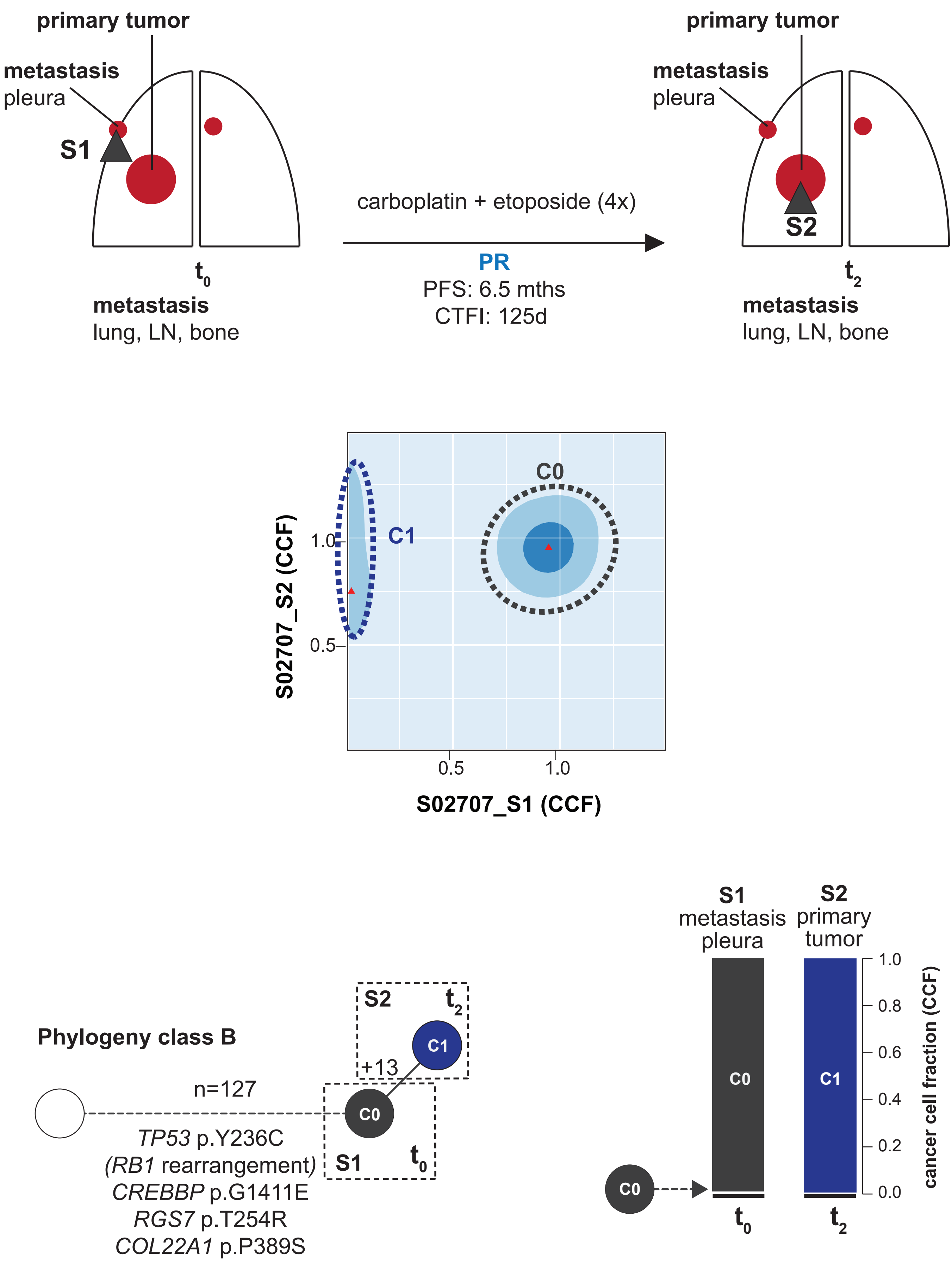

S02714 T3N2M1, stage IV

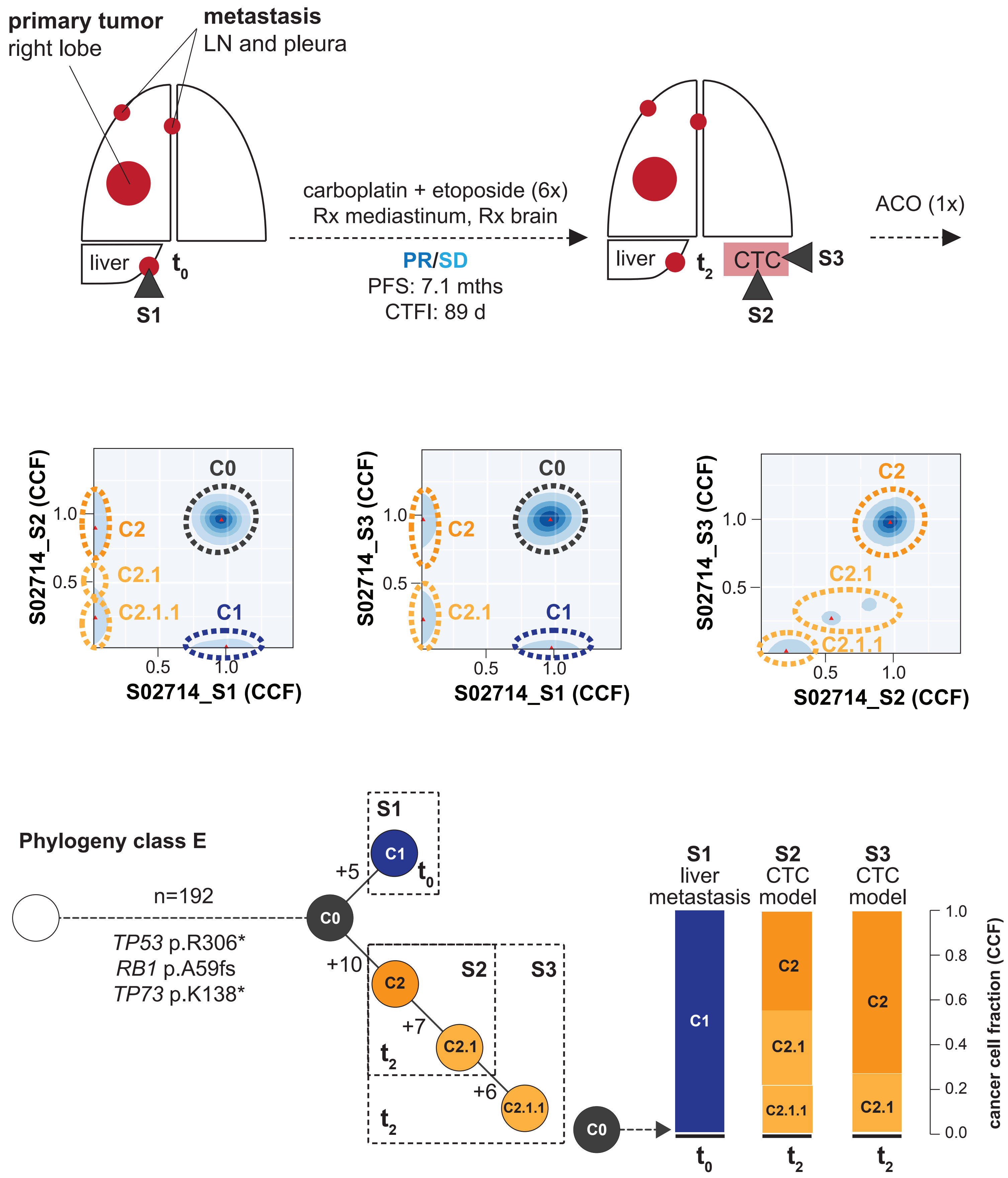

S02718 TxN2M1, stage IV

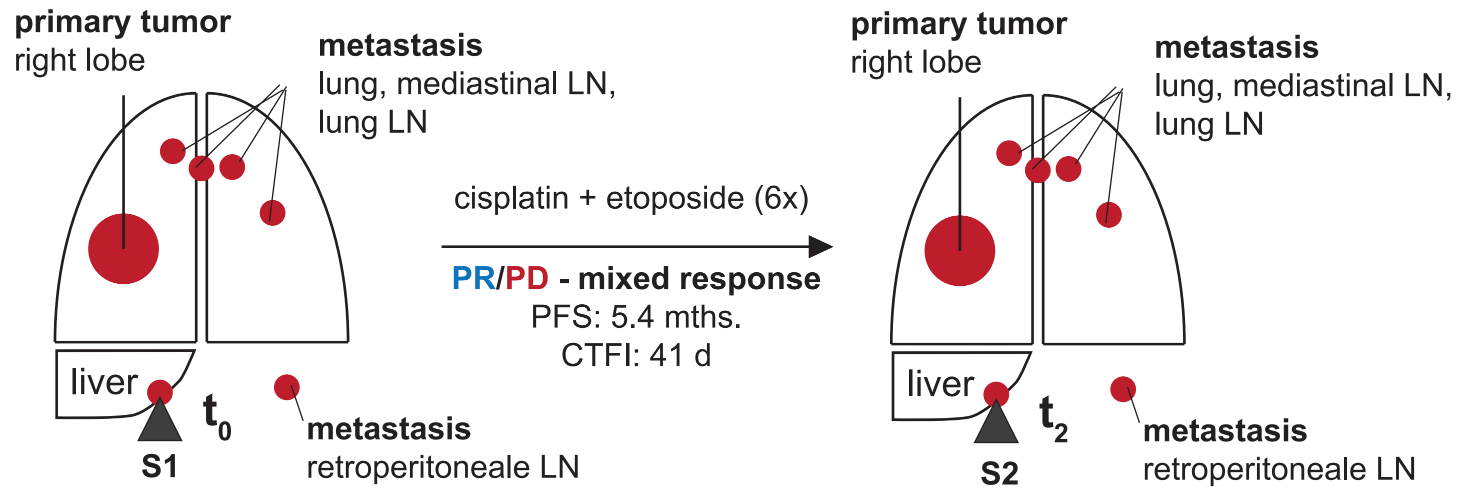

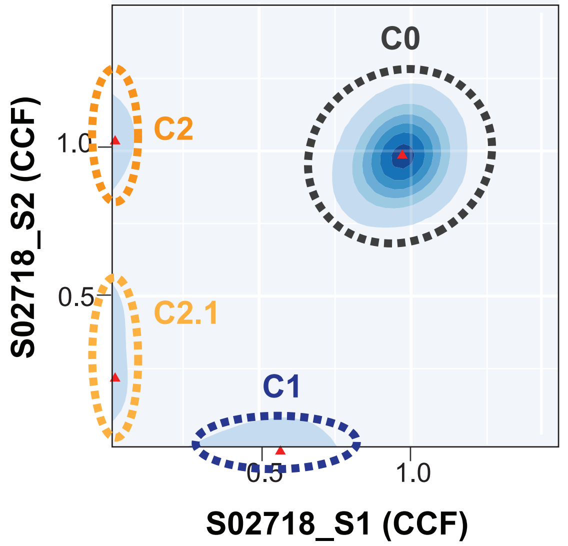

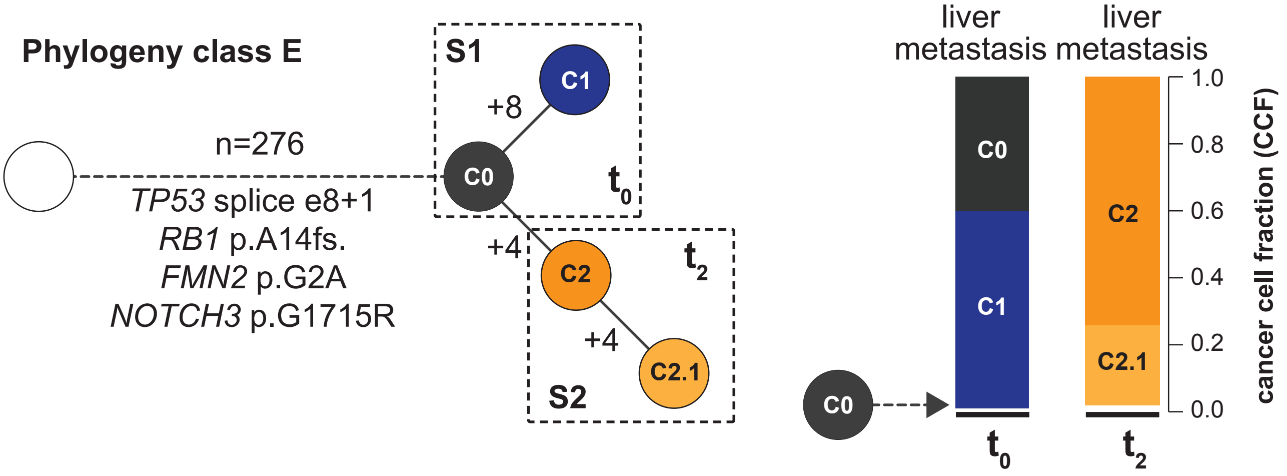

S02722 T1N1M0, stage II

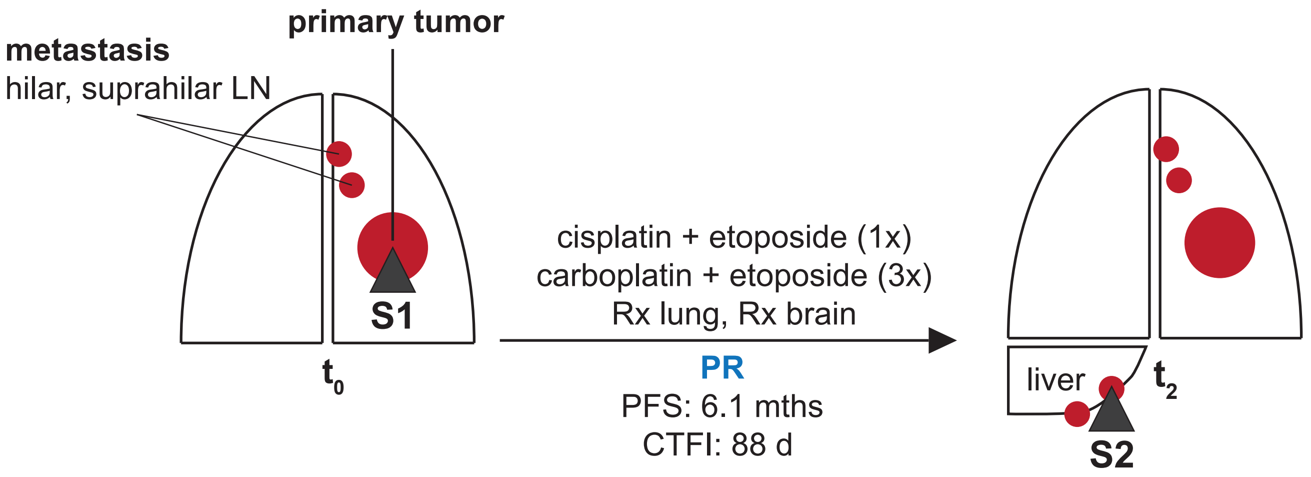

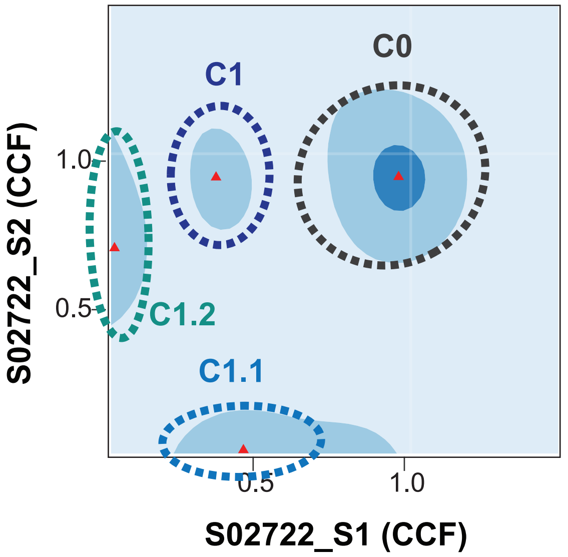

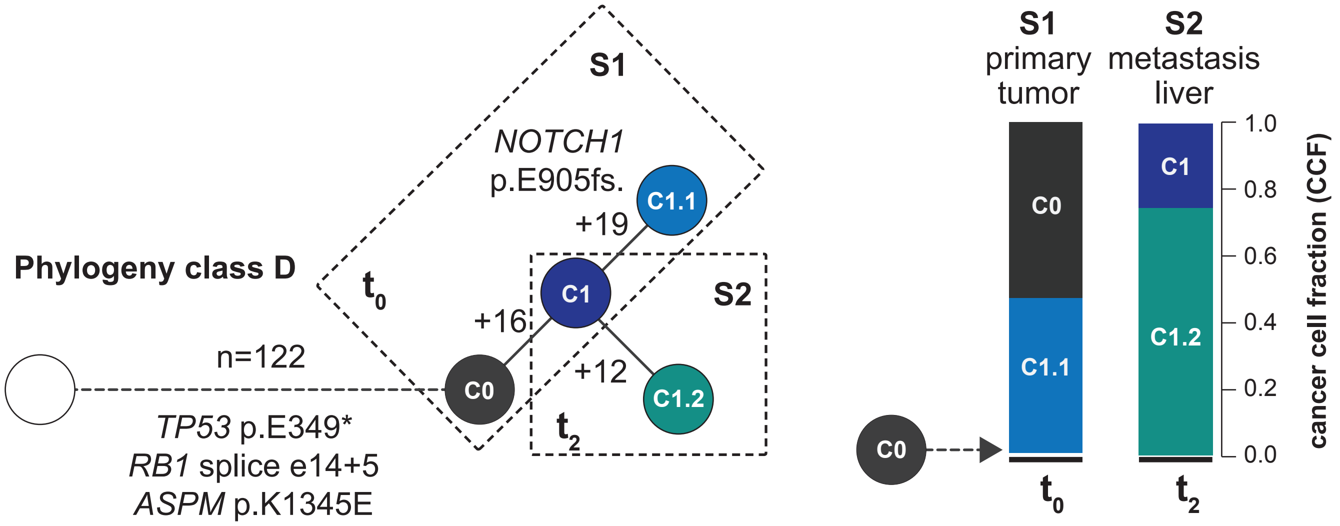

S02724 T3N1M0, stage III

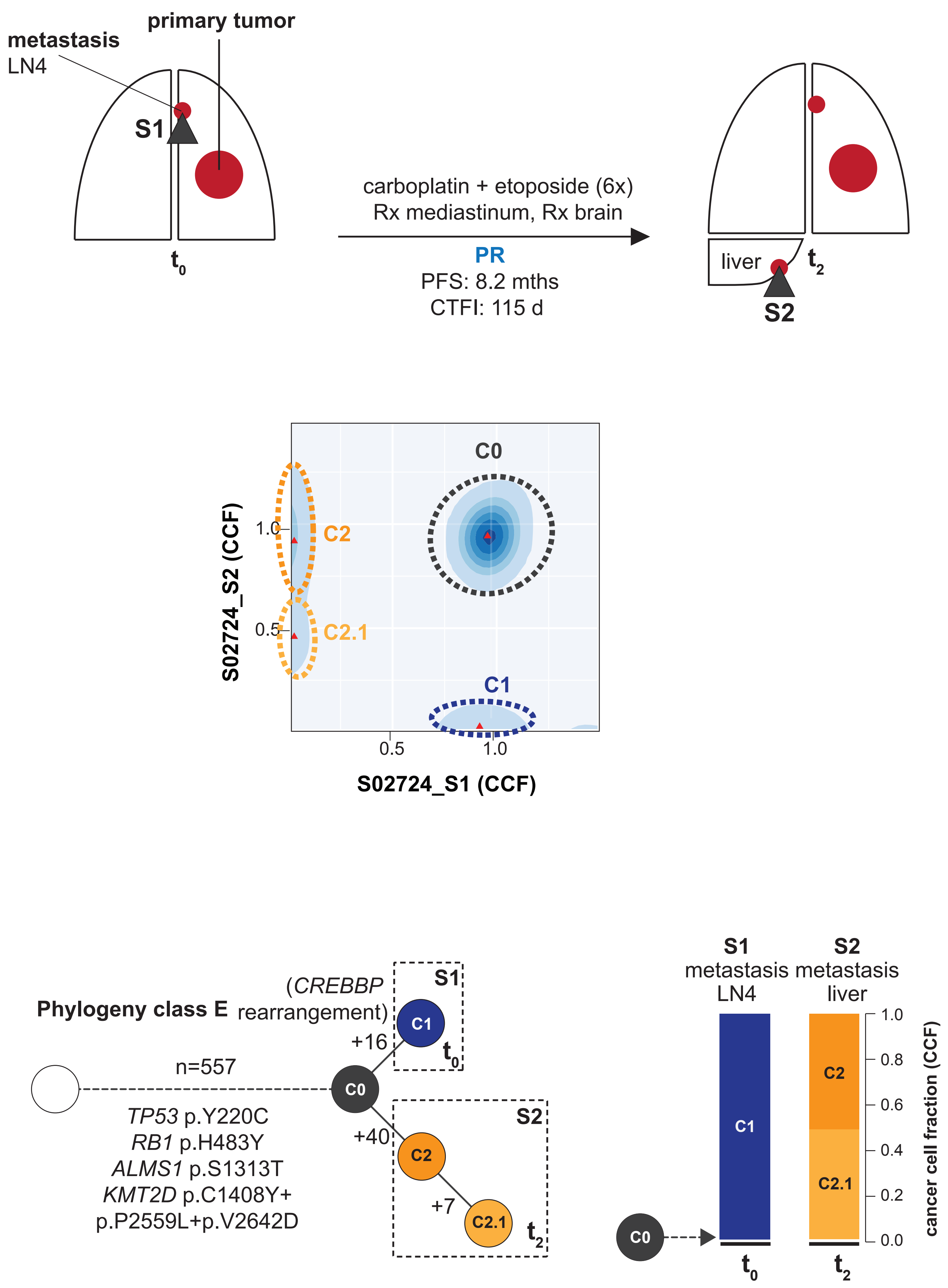

S02725 stage IV

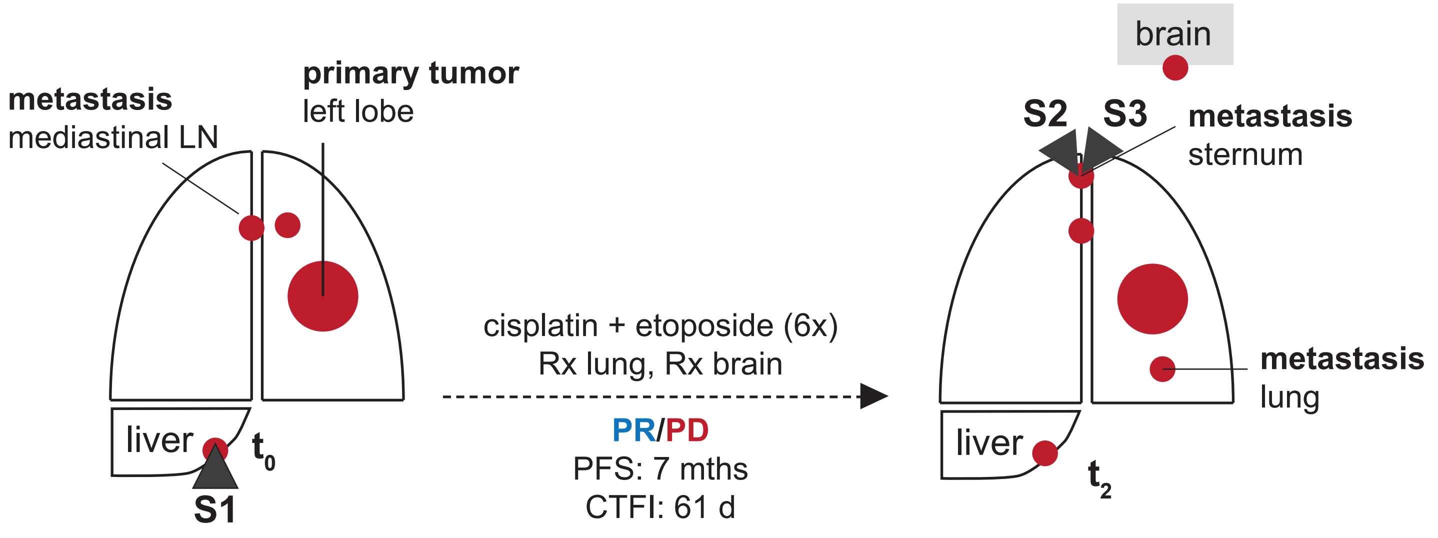

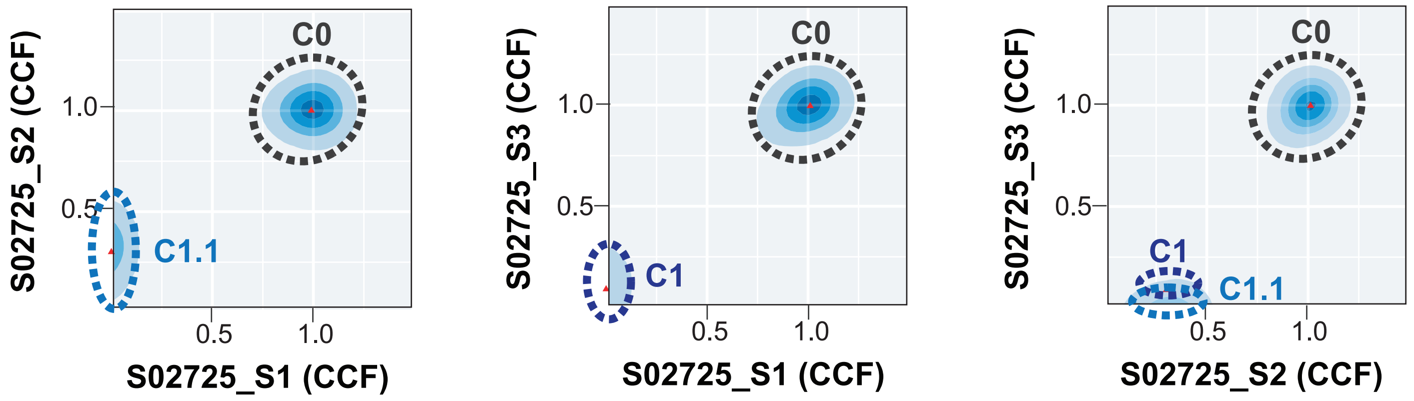

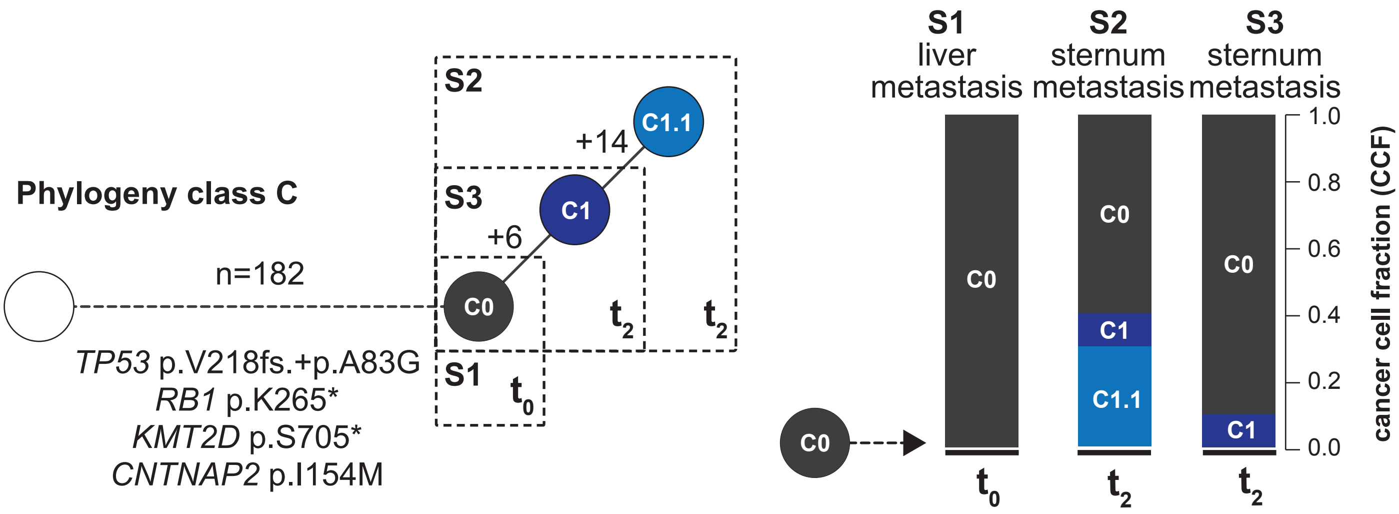

S02730 T4N2M1, Stage IV

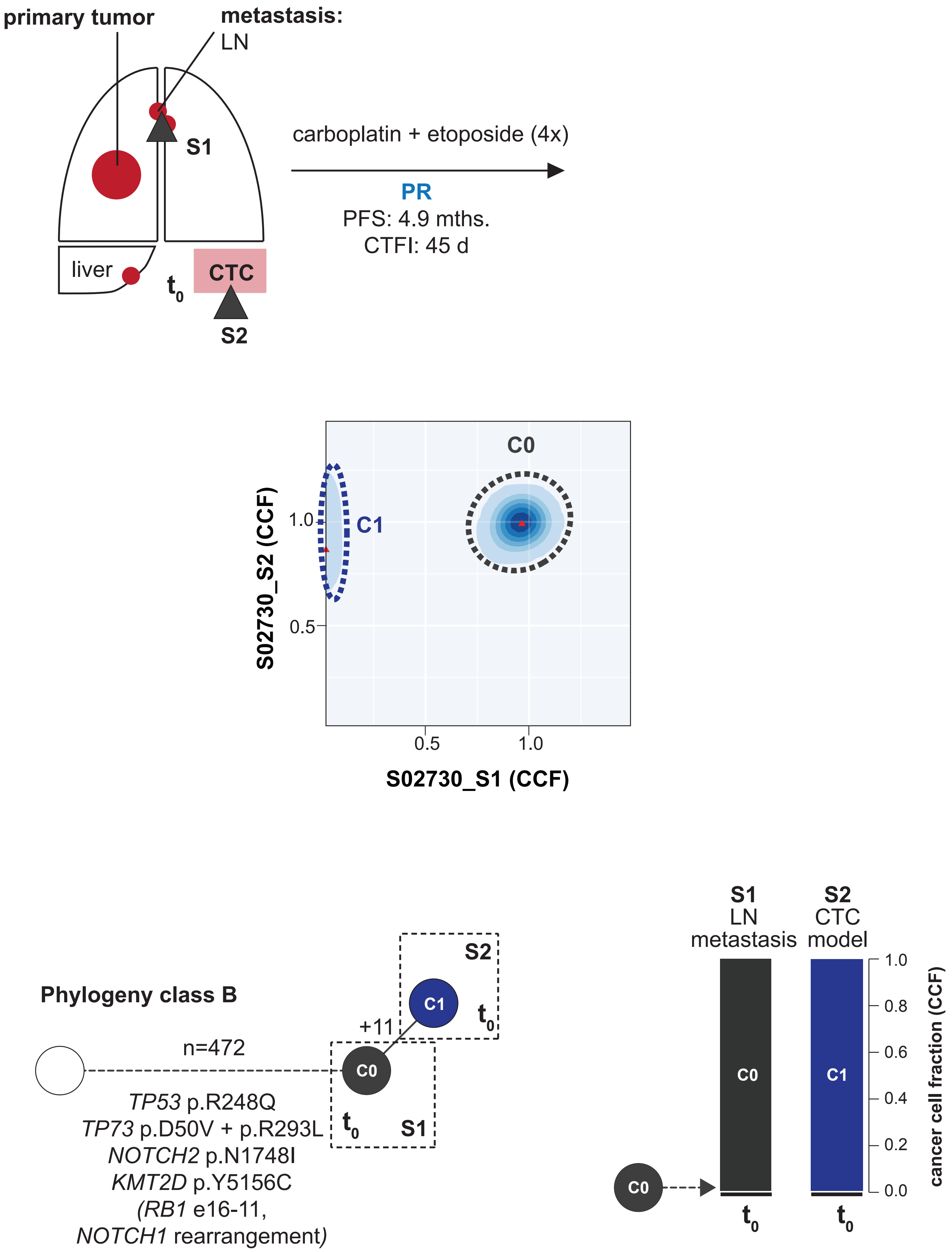

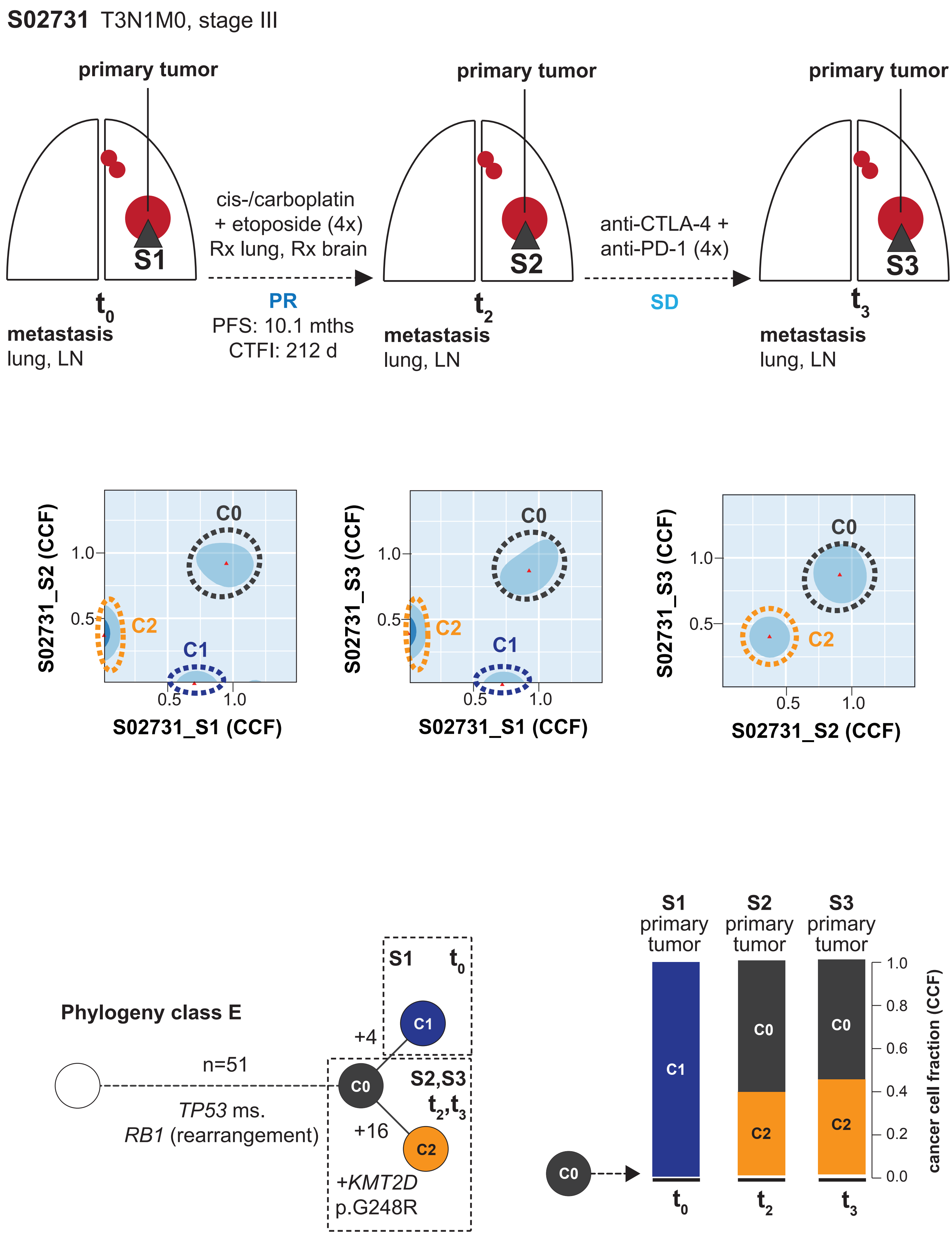

S02731 T3N1M0, stage III
primary tumor
S1
t0
metastasis
lung, LN
cis-/carboplatin
+ etoposide (4x)
Rx lung, Rx brain
PR
PFS: 10.1 mths
CTFI: 212 d
primary tumor
S2
t2
metastasis
lung, LN
anti-CTLA-4 +
anti-PD-1 (4x)
SD
primary tumor
S3
t3
metastasis
lung, LN
S02731_S2 (CCF)
S02731_S1 (CCF)
C0
C1
C2
0.5
1.0
S02731_S3 (CCF)
S02731_S1 (CCF)
C0
C1
C2
S02731_S3 (CCF)
S02731_S2 (CCF)
C0
C2
Phylogeny class E
n=51
TP53 ms.
RB1 (rearrangement)
C0
+4
C1
S1 t0
+16
C2
S2,S3
t2,t3
+KMT2D
p.G248R
S1
primary
tumor
S2
primary
tumor
S3
primary
tumor
C1
C0
C2
C0
C2
t0
t2
t3
1.0
0.8
0.6
0.4
0.2
0.0
cancer cell fraction (CCF)

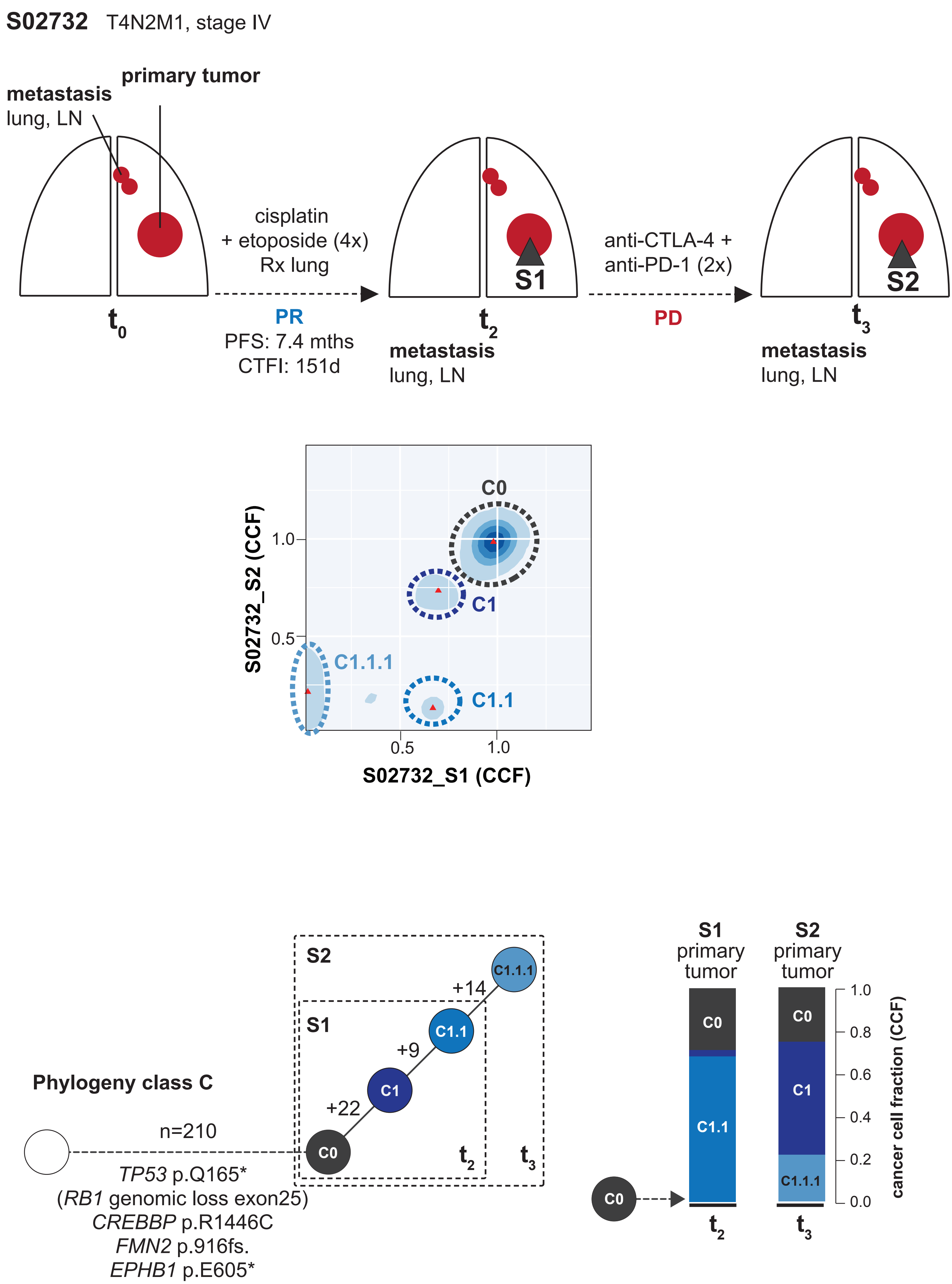

S02732 T4N2M1, stage IV
primary tumor
metastasis
lung, LN
cisplatin
+ etoposide (4x)
Rx lung
PR
PFS: 7.4 mths
CTFI: 151d
S1
metastasis
lung, LN
anti-CTLA-4 +
anti-PD-1 (2x)
PD
S2
metastasis
lung, LN
C0
C1
C1.1.1
C1.1
1.0
0.5
S02732_S2 (CCF)
S02732_S1 (CCF)
Phylogeny class C
n=210
TP53 p.Q165*
(RB1 genomic loss exon25)
CREBBP p.R1446C
FMN2 p.916fs.
EPHB1 p.E605*
+22
+9
+14
S1
primary
tumor
S2
primary
tumor
0.0
0.2
0.4
0.6
0.8
cancer cell fraction (CCF)

S02741 T4N2M1, stage IV

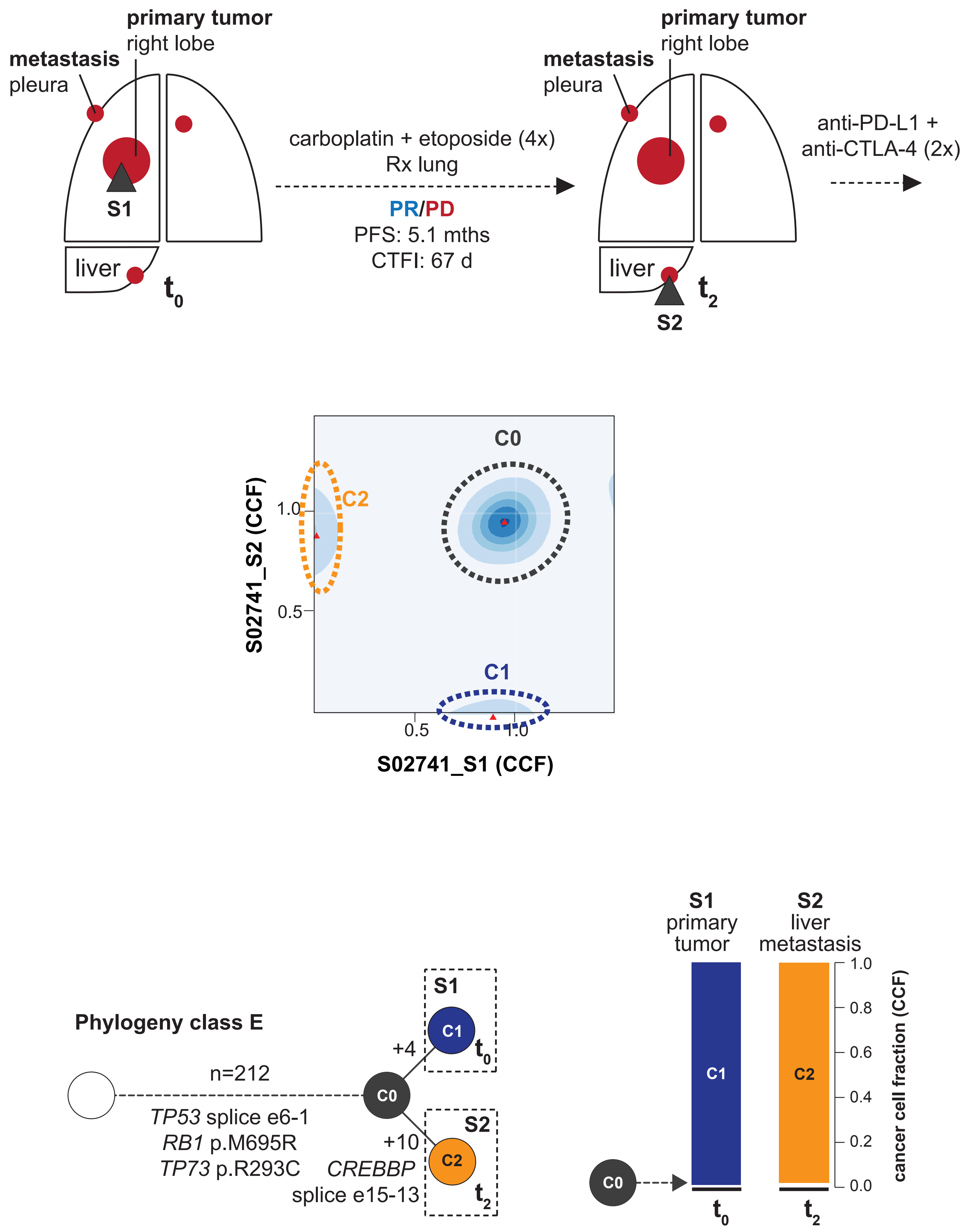

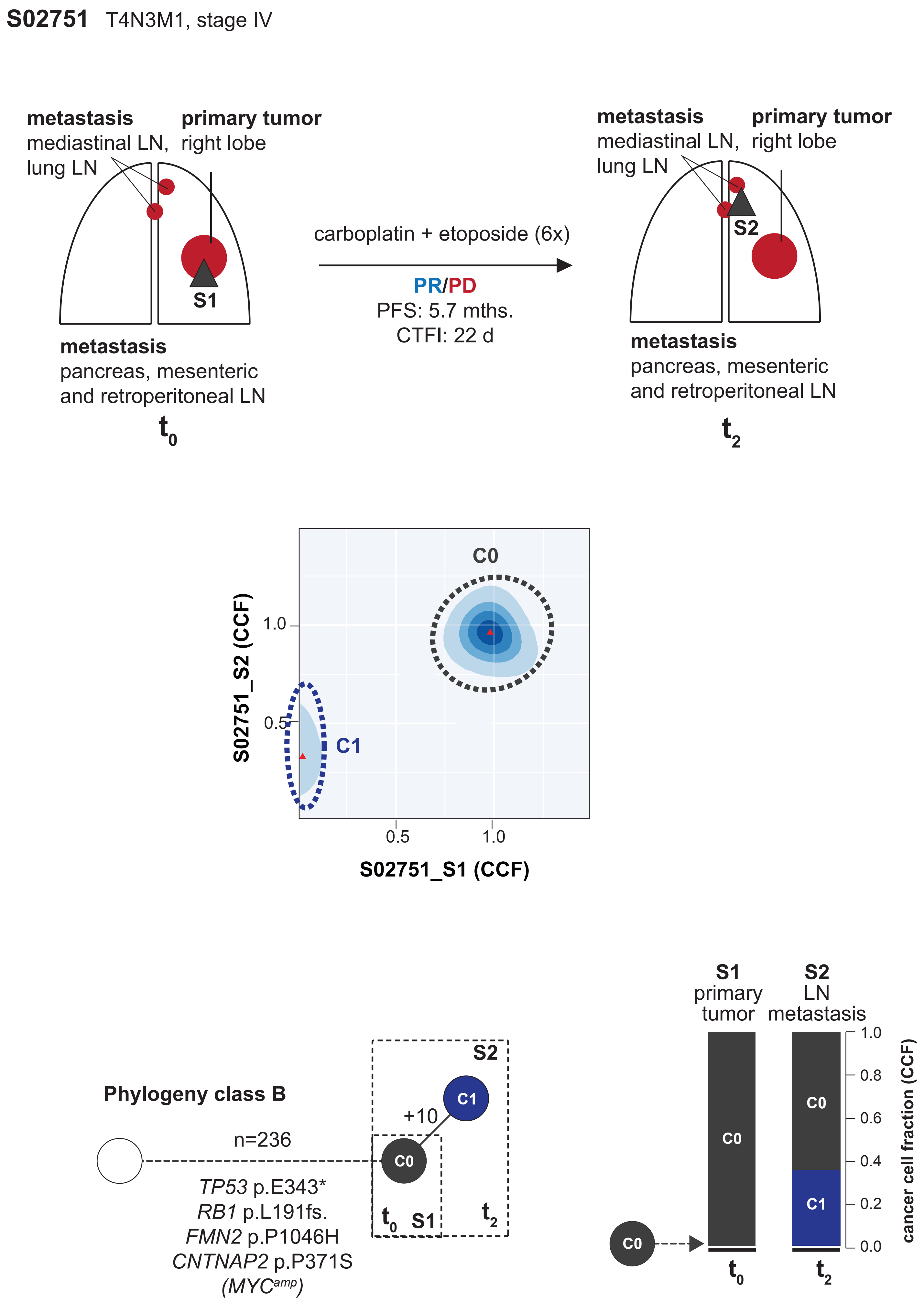
S02751 T4N3M1, stage IV
metastasis
mediastinal LN,
lung LN
primary tumor
right lobe
S1
metastasis
pancreas, mesenteric
and retroperitoneal LN
t0
carboplatin + etoposide (6x)
PR/PD
PFS: 5.7 mths.
CTFI: 22 d
metastasis
mediastinal LN,
lung LN
primary tumor
right lobe
S2
metastasis
pancreas, mesenteric
and retroperitoneal LN
t2
C0
C1
1.0
0.5
S02751_S2 (CCF)
0.5
1.0
S02751_S1 (CCF)
Phylogeny class B
n=236
TP53 p.E343*
RB1 p.L191fs.
FMN2 p.P1046H
CNTNAP2 p.P371S
(MYCamp)
+10
C1
C0
S2
t0 S1
t2
S1
primary
tumor
S2
LN
metastasis
C0
C0
C1
C0
1.0
0.8
0.6
0.4
0.2
0.0
cancer cell fraction (CCF)
t0
t2

S02764 T2N3M0, stage III

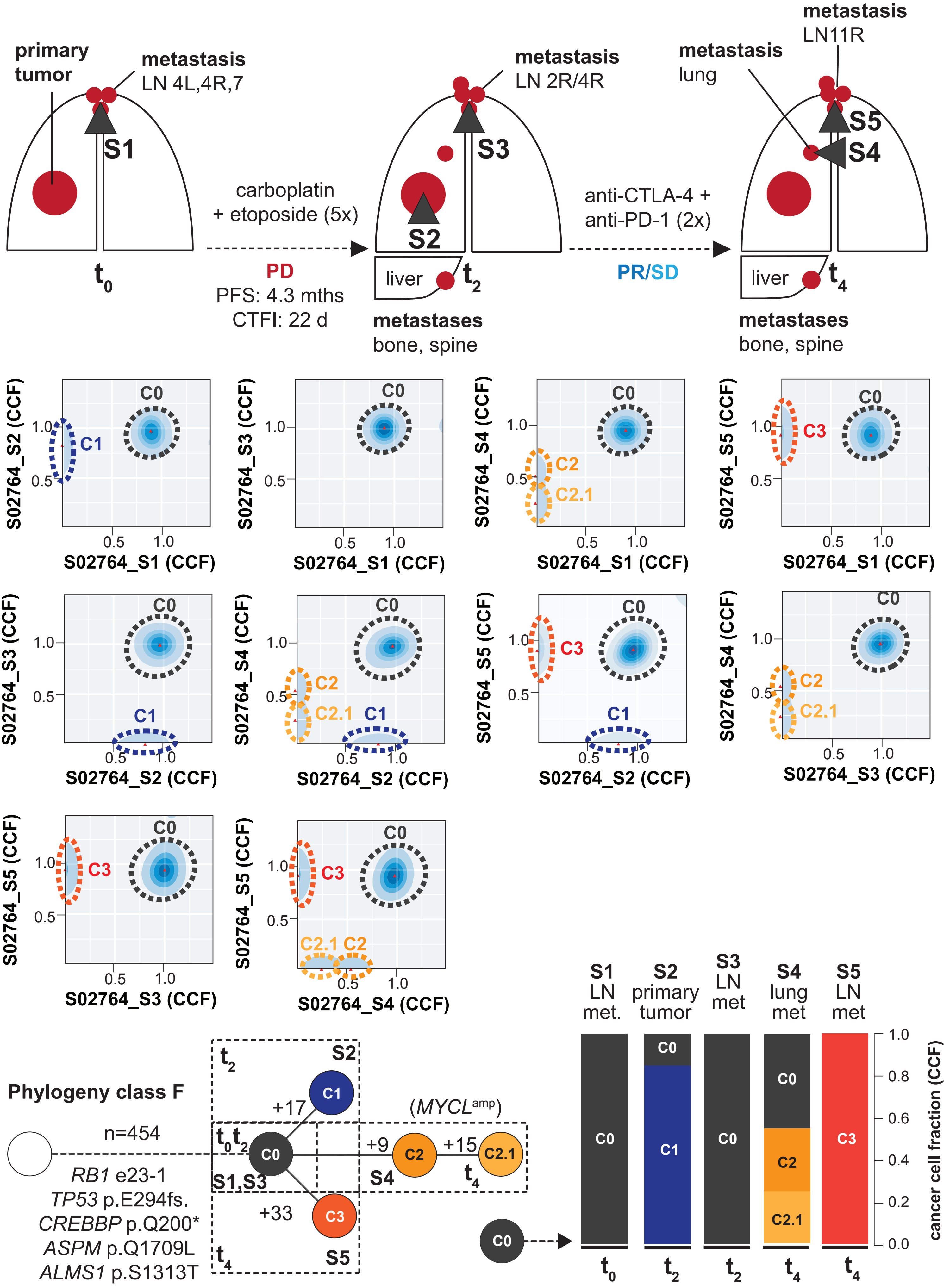

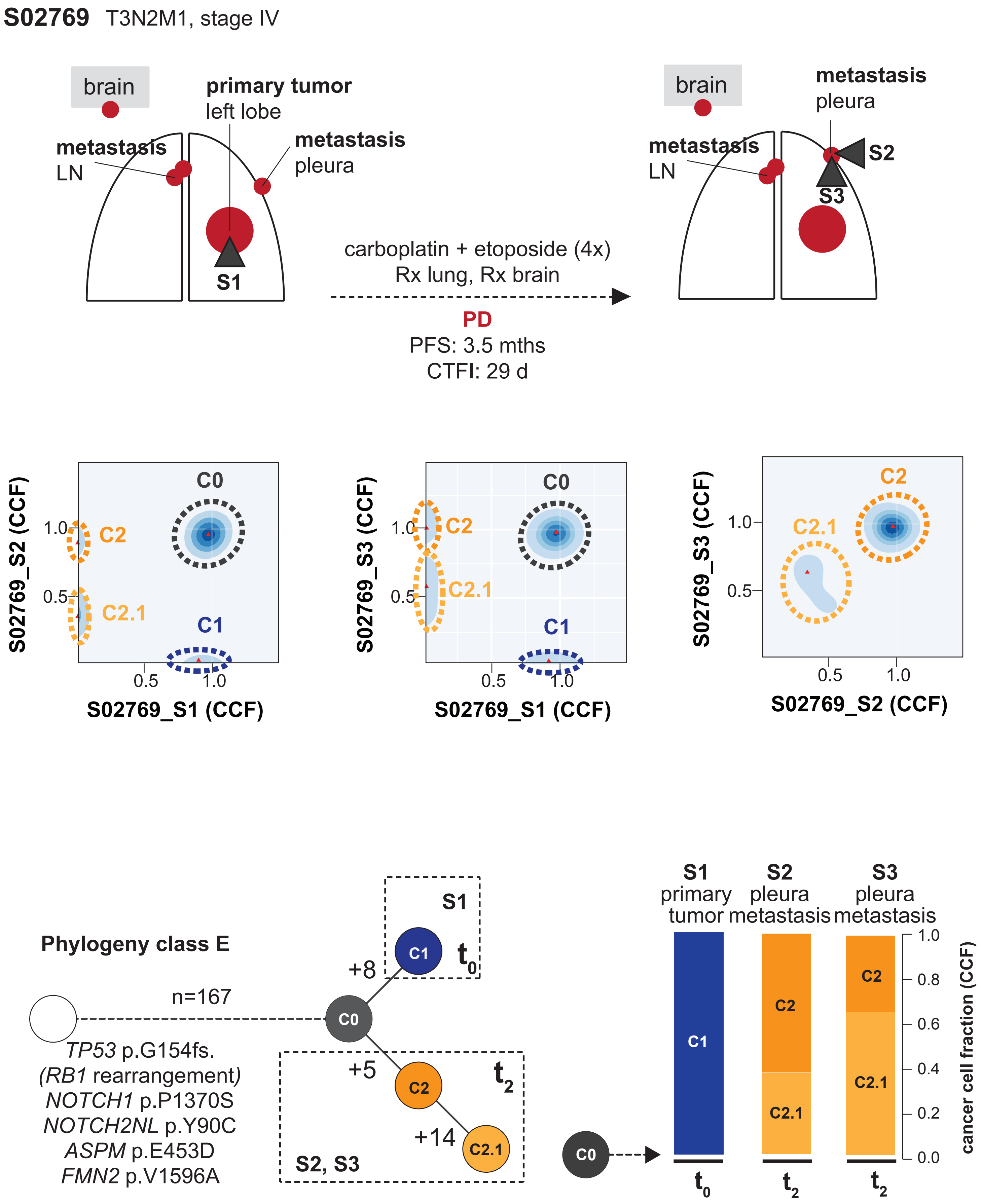
S02769 T3N2M1, stage IV
brain
primary tumor
left lobe
metastasis
LN
metastasis
pleura
S1
carboplatin + etoposide (4x)
Rx lung, Rx brain
PD
PFS: 3.5 mths
CTFI: 29 d
brain
metastasis
pleura
metastasis
LN
S2
S3
S02769_S2 (CCF)
S02769_S1 (CCF)
S02769_S3 (CCF)
C0
C1
C2
C2.1
Phylogeny class E
n=167
TP53 p.G154fs.
(RB1 rearrangement)
NOTCH1 p.P1370S
NOTCH2NL p.Y90C
ASPM p.E453D
FMN2 p.V1596A
+8
+5
+14
t0
t2
S2, S3
S1
primary tumor
S2
pleura metastasis
S3
pleura metastasis
cancer cell fraction (CCF)

S02774 T2N2M1, stage IV

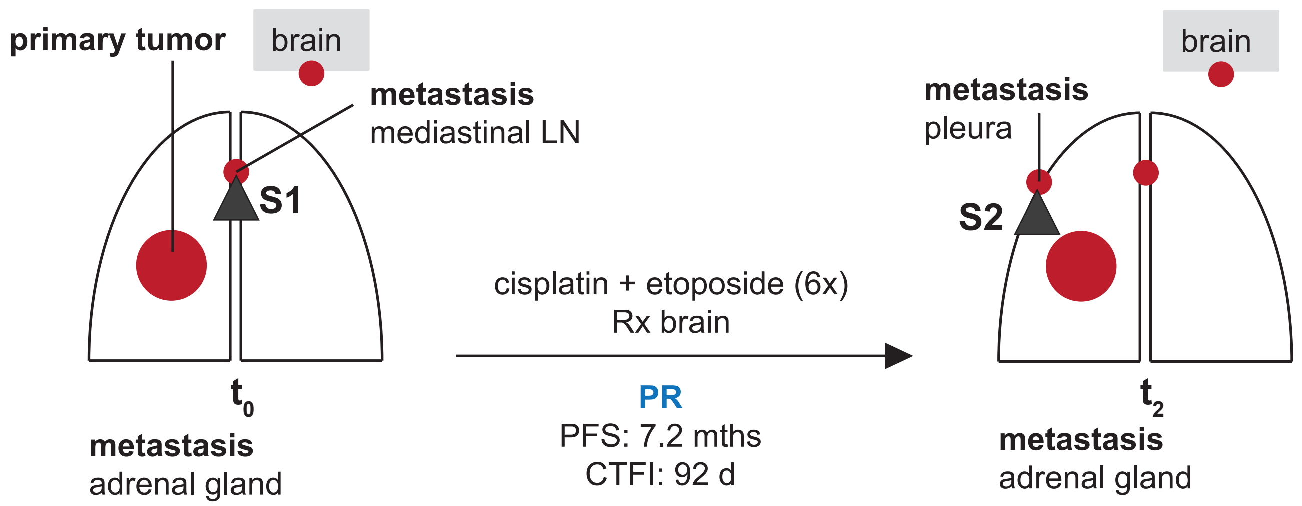

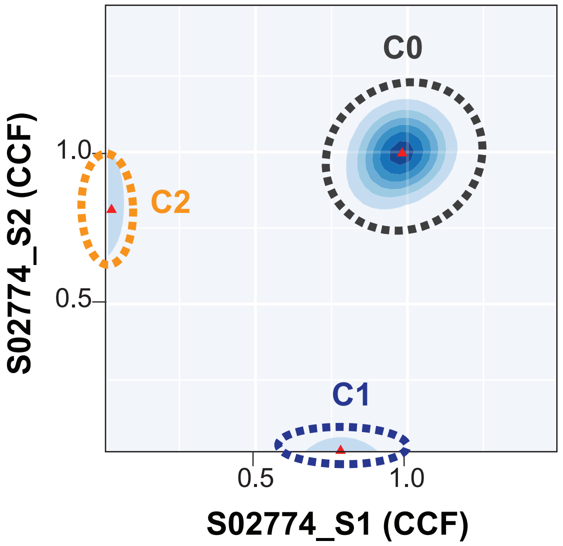

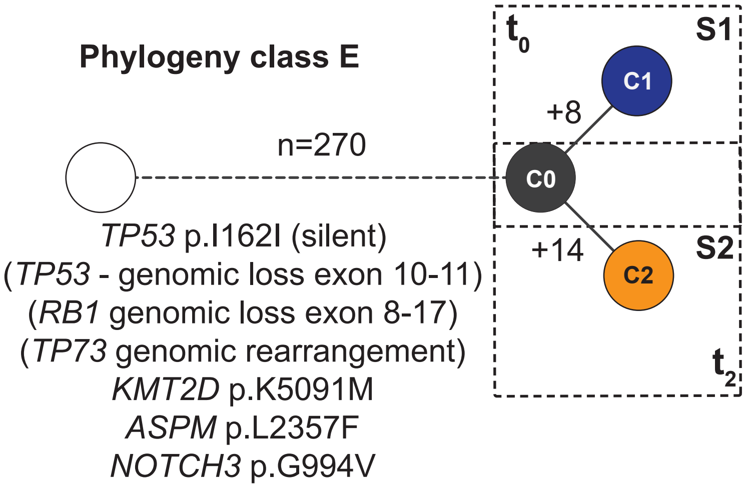

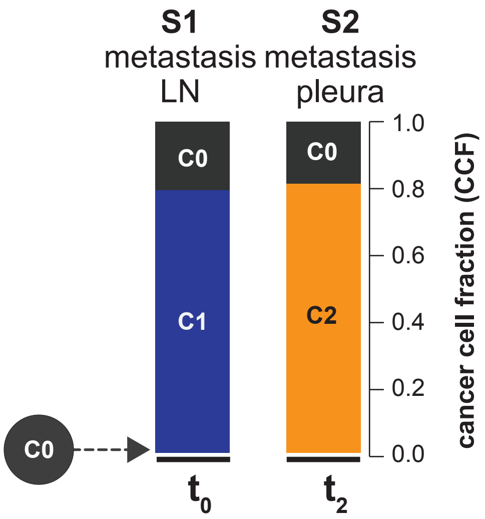

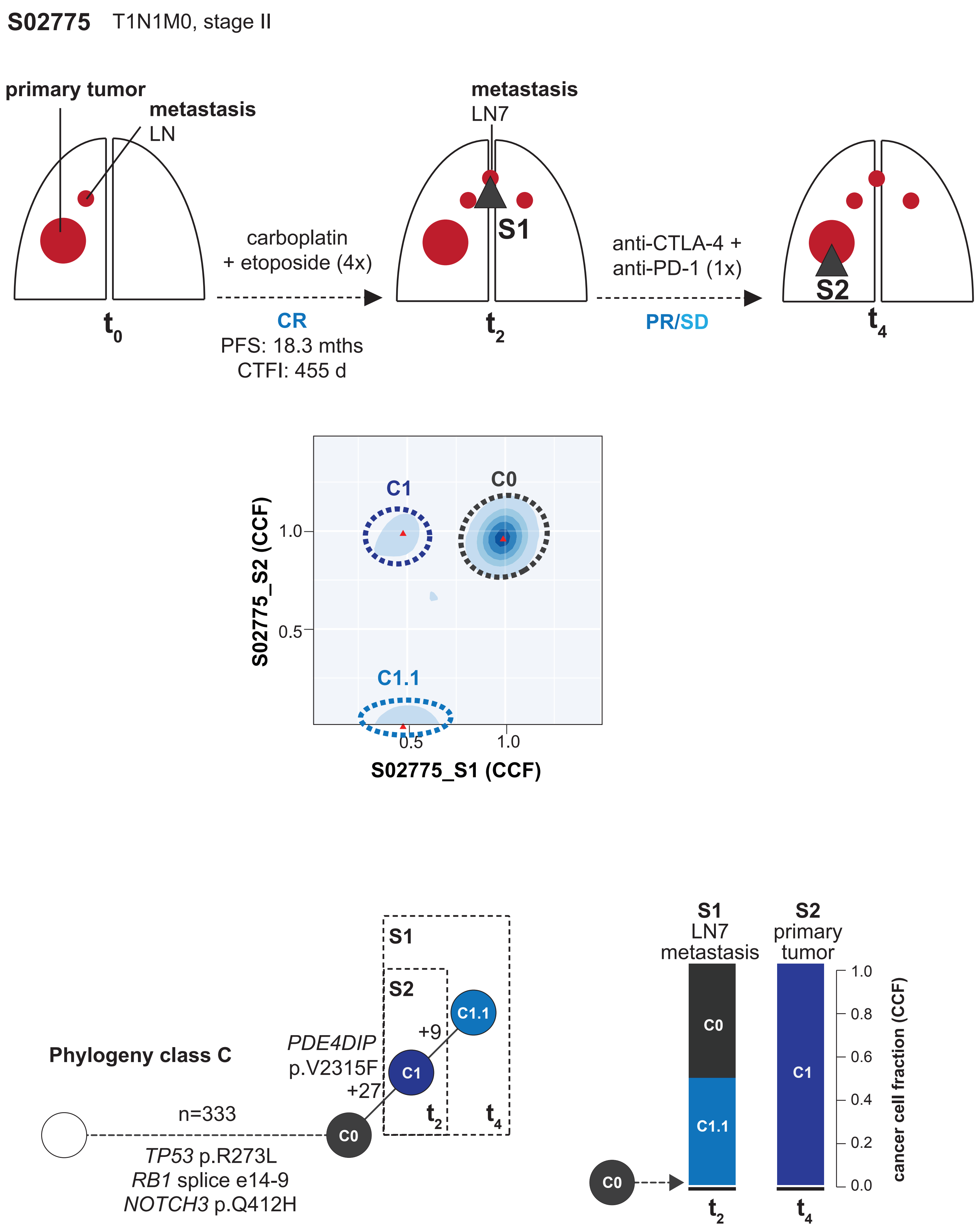
S02775 T1N1M0, stage II
primary tumor
metastasis
LN
t_0
carboplatin
+ etoposide (4x)
CR
PFS: 18.3 mths
CTFI: 455 d
metastasis
LN7
S1
t_2
anti-CTLA-4 +
anti-PD-1 (1x)
PR/SD
S2
t_4
C1
C0
C1.1
1.0
0.5
S02775_S2 (CCF)
S02775_S1 (CCF)
Phylogeny class C
n=333
TP53 p.R273L
RB1 splice e14-9
NOTCH3 p.Q412H
PDE4DIP
p.V2315F
+27
+9
S1
S2
t_2
t_4
C0
C1
C1.1
S1
LN7
metastasis
S2
primary
tumor
C0
C1.1
C1
1.0
0.8
0.6
0.4
0.2
0.0
cancer cell fraction (CCF)
t_2
t_4

S02782 T4N3M1, stage IV

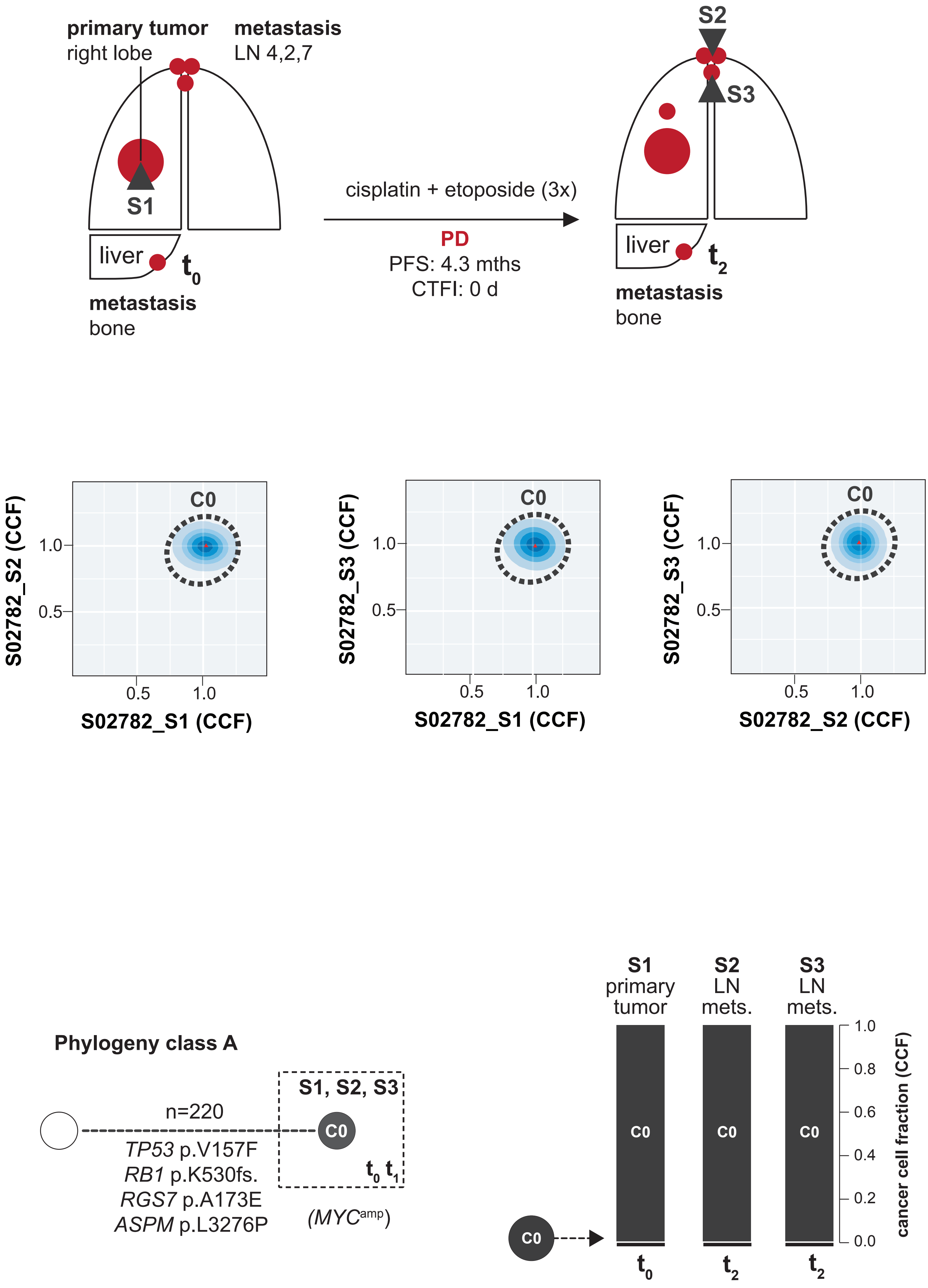

S02783 T2N1M0, stage II; lung resection >5 years ago

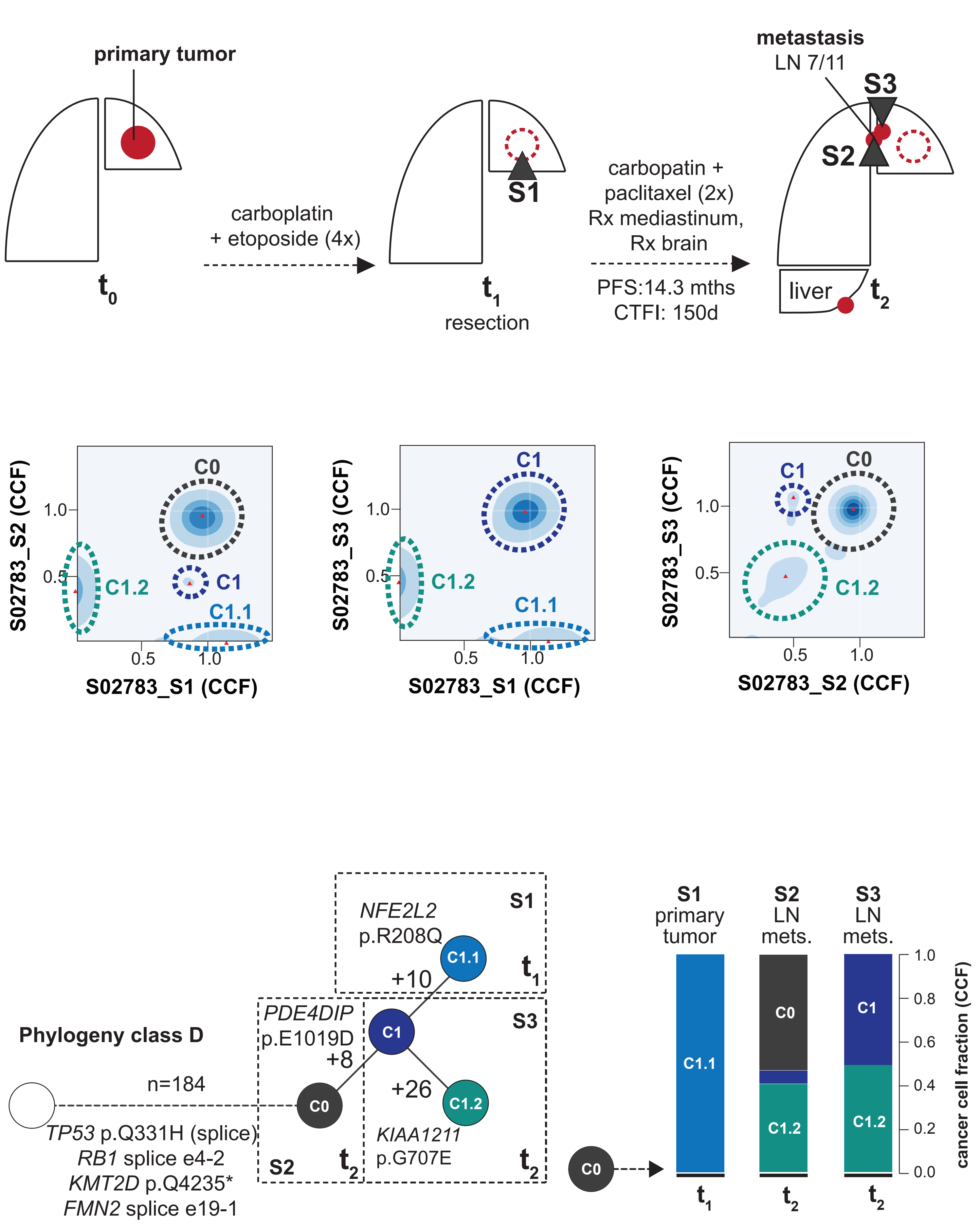

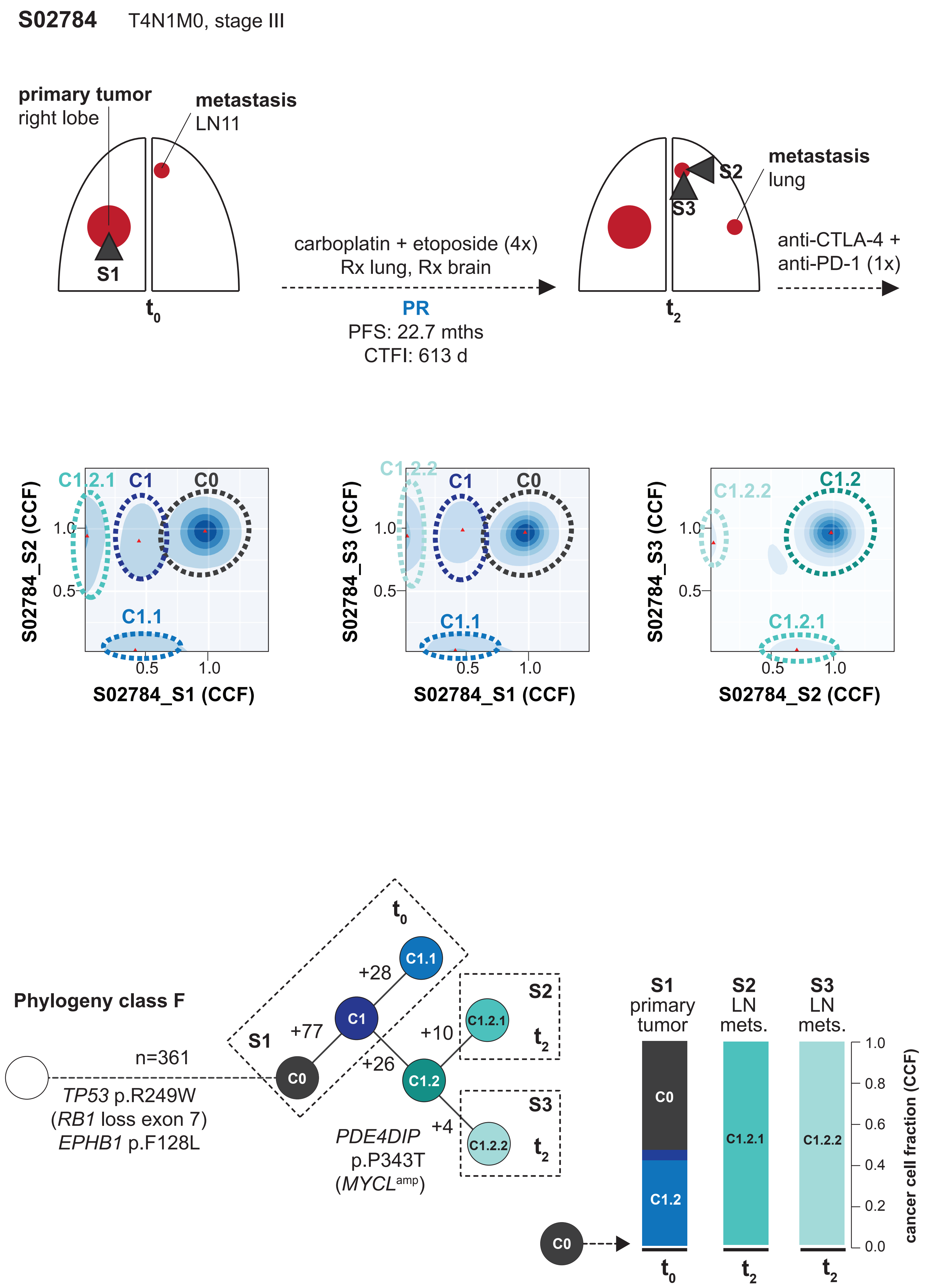

S02784 T4N1M0, stage III
primary tumor
right lobe
metastasis
LN11
S1
t0
carboplatin + etoposide (4x)
Rx lung, Rx brain
PR
PFS: 22.7 mths
CTFI: 613 d
S2
S3
metastasis
lung
t2
anti-CTLA-4 +
anti-PD-1 (1x)
C1.2.1
C1
C0
C1.1
S02784_S2 (CCF)
S02784_S1 (CCF)
C1.2.2
S02784_S3 (CCF)
C1.2
1.0
0.5
Phylogeny class F
n=361
TP53 p.R249W
(RB1 loss exon 7)
EPHB1 p.F128L
+77
+28
+26
+10
+4
C1.1
C1.2.1
C1.2.2
PDE4DIP
p.P343T
(MYCL amp)
S1
primary
tumor
S2
LN
mets.
S3
LN
mets.
cancer cell fraction (CCF)
0.0
0.2
0.4
0.6
0.8
1.0

S02790 T3N2M1, stage IV

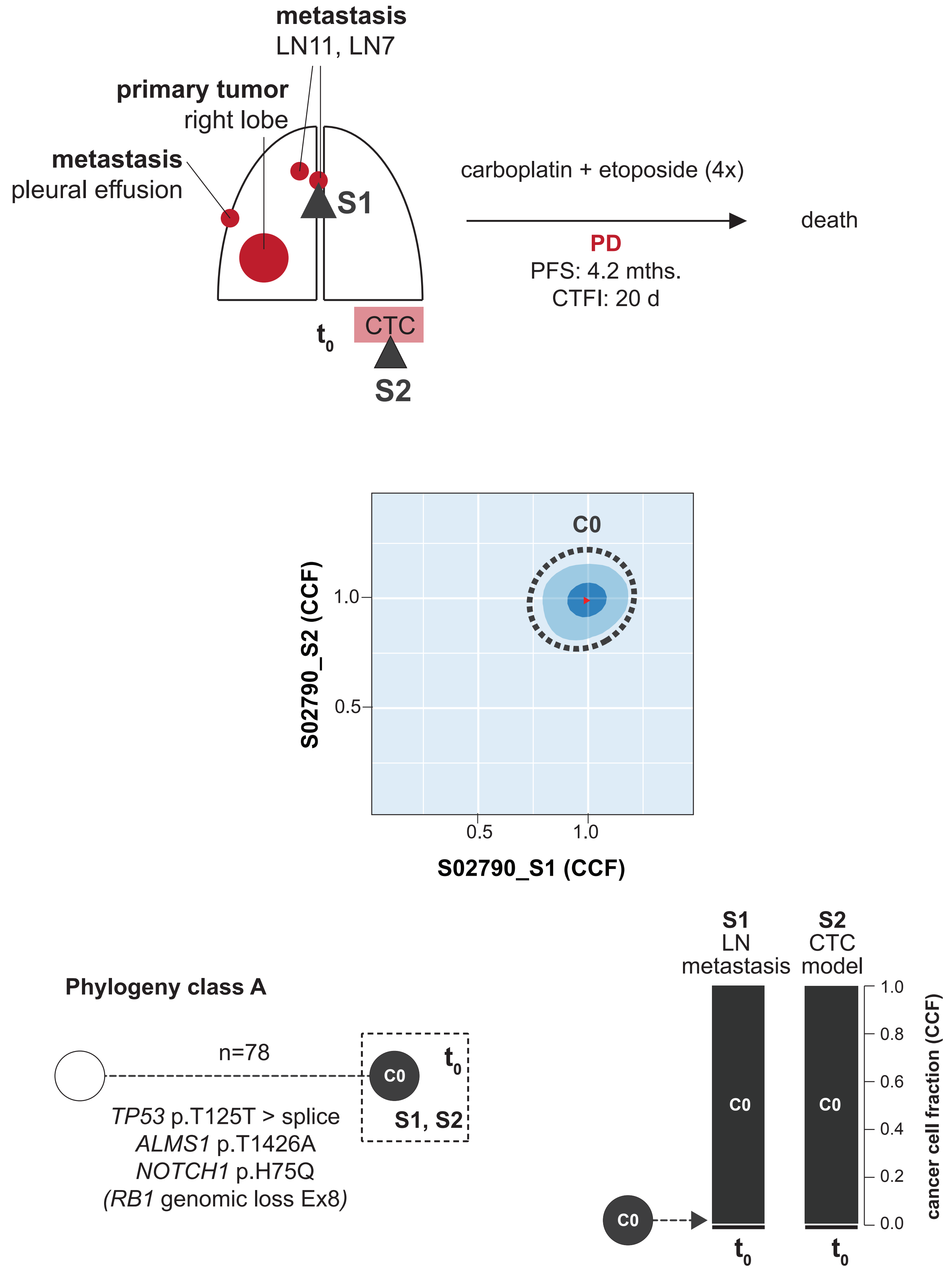

S02806 stage IV

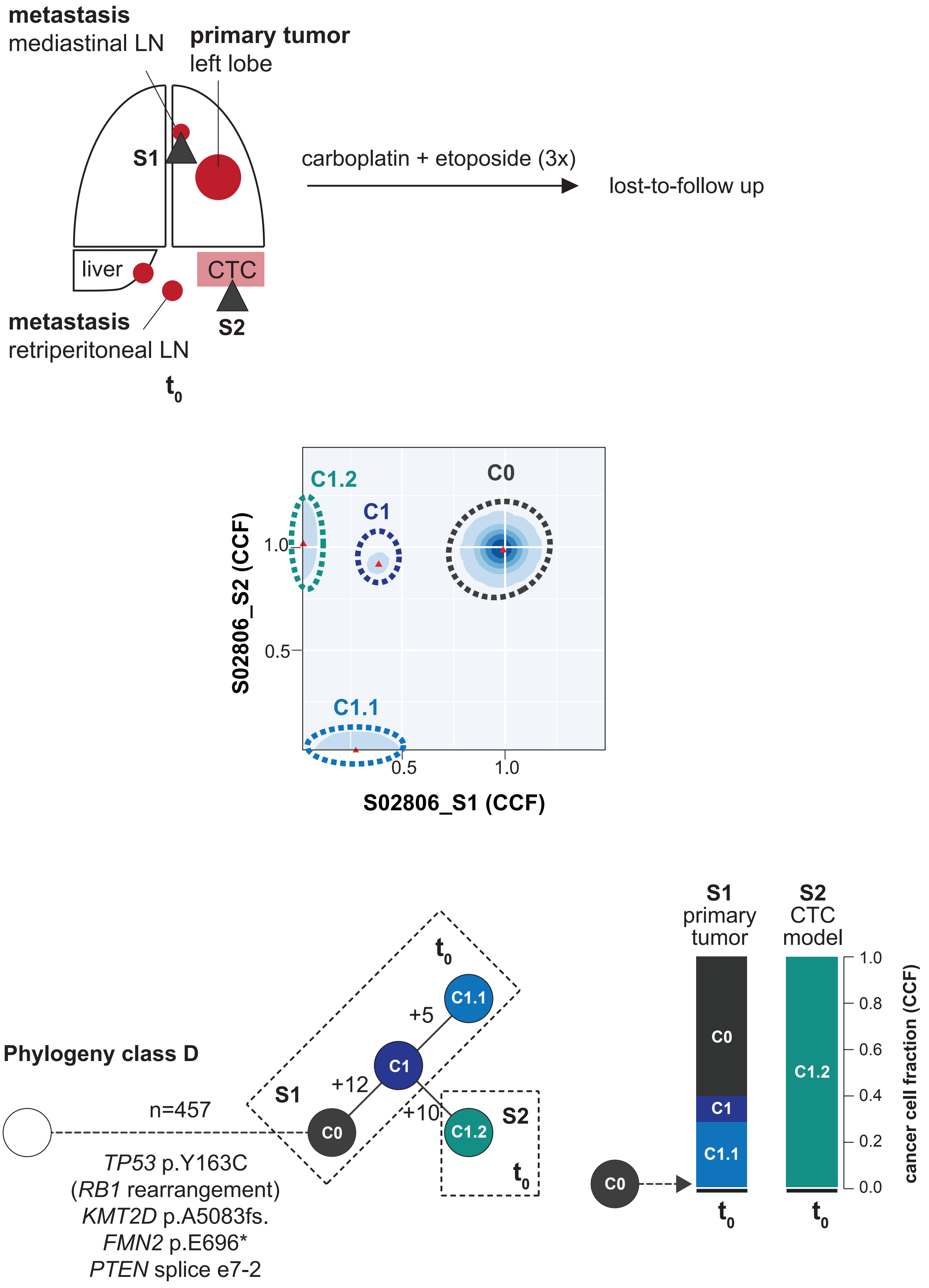

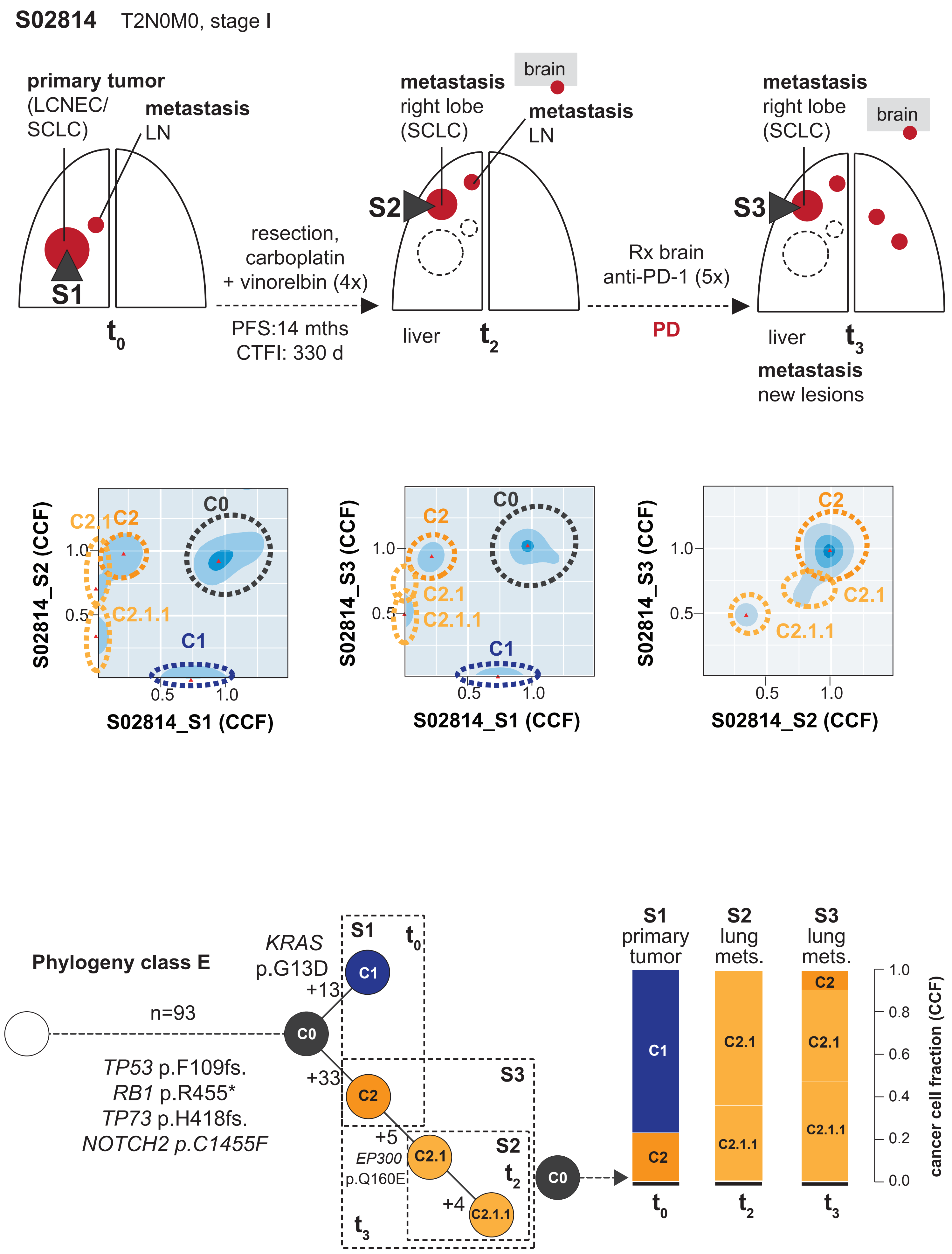
S02814
T2N0M0, stage I
primary tumor
(LCNEC/
SCLC)
metastasis
LN
S1
t_0
resection,
carboplatin
+ vinorelbin (4x)
PFS:14 mths
CTFI: 330 d
metastasis
right lobe
(SCLC)
brain
metastasis
LN
S2
liver
t_2
Rx brain
anti-PD-1 (5x)
PD
metastasis
right lobe
(SCLC)
brain
S3
liver
t_3
metastasis
new lesions
C0
C2.1
C2
1.0
0.5
C2.1.1
C1
0.5
1.0
S02814_S2 (CCF)
S02814_S1 (CCF)
C0
C2
1.0
C2.1
0.5
C2.1.1
C1
0.5
1.0
S02814_S3 (CCF)
S02814_S1 (CCF)
C2
1.0
C2.1
0.5
C2.1.1
0.5
1.0
S02814_S3 (CCF)
S02814_S2 (CCF)
Phylogeny class E
n=93
TP53 p.F109fs.
RB1 p.R455*
TP73 p.H418fs.
NOTCH2 p.C1455F
KRAS
p.G13D
+13
C0
C1
S1
t_0
+33
C2
S3
+5
EP300
p.Q160E
C2.1
S2
t_2
+4
C2.1.1
t_3
C0
S1
primary
tumor
S2
lung
mets.
S3
lung
mets.
C1
C2
C2.1
C2.1.1
C2
C2.1
C2.1.1
t_0
t_2
t_3
1.0
0.8
0.6
0.4
0.2
0.0
cancer cell fraction (CCF)

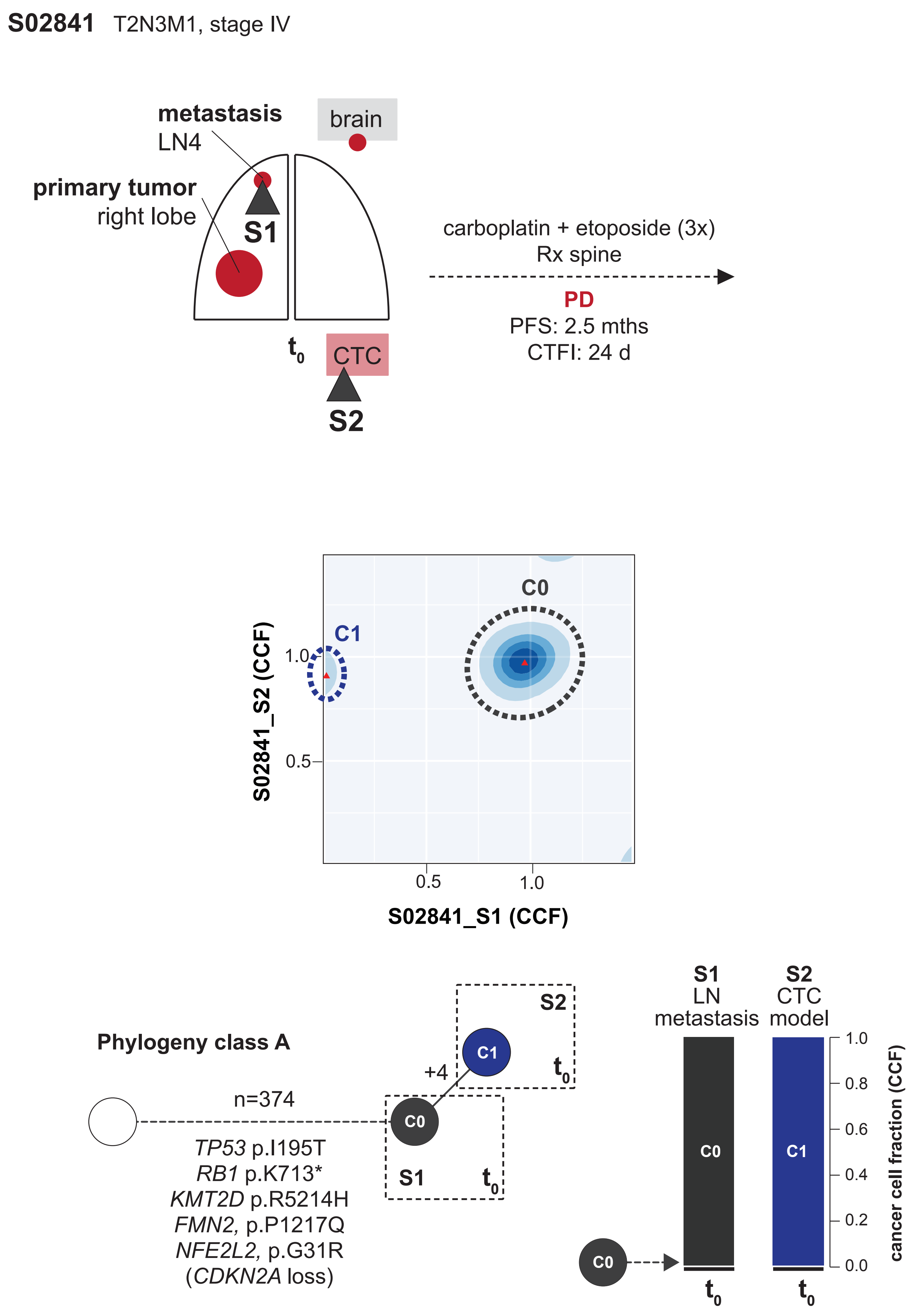
S02841 T2N3M1, stage IV
metastasis
LN4
brain
primary tumor
right lobe
S1
t0
CTC
S2
carboplatin + etoposide (3x)
Rx spine
PD
PFS: 2.5 mths
CTFI: 24 d
C0
C1
1.0
0.5
S02841_S2 (CCF)
0.5
1.0
S02841_S1 (CCF)
Phylogeny class A
n=374
TP53 p.I195T
RB1 p.K713*
KMT2D p.R5214H
FMN2, p.P1217Q
NFE2L2, p.G31R
(CDKN2A loss)
+4
C0
C1
S1
S2
t0
S1
LN
metastasis
S2
CTC
model
C0
C1
1.0
0.8
0.6
0.4
0.2
0.0
cancer cell fraction (CCF)
t0
t0

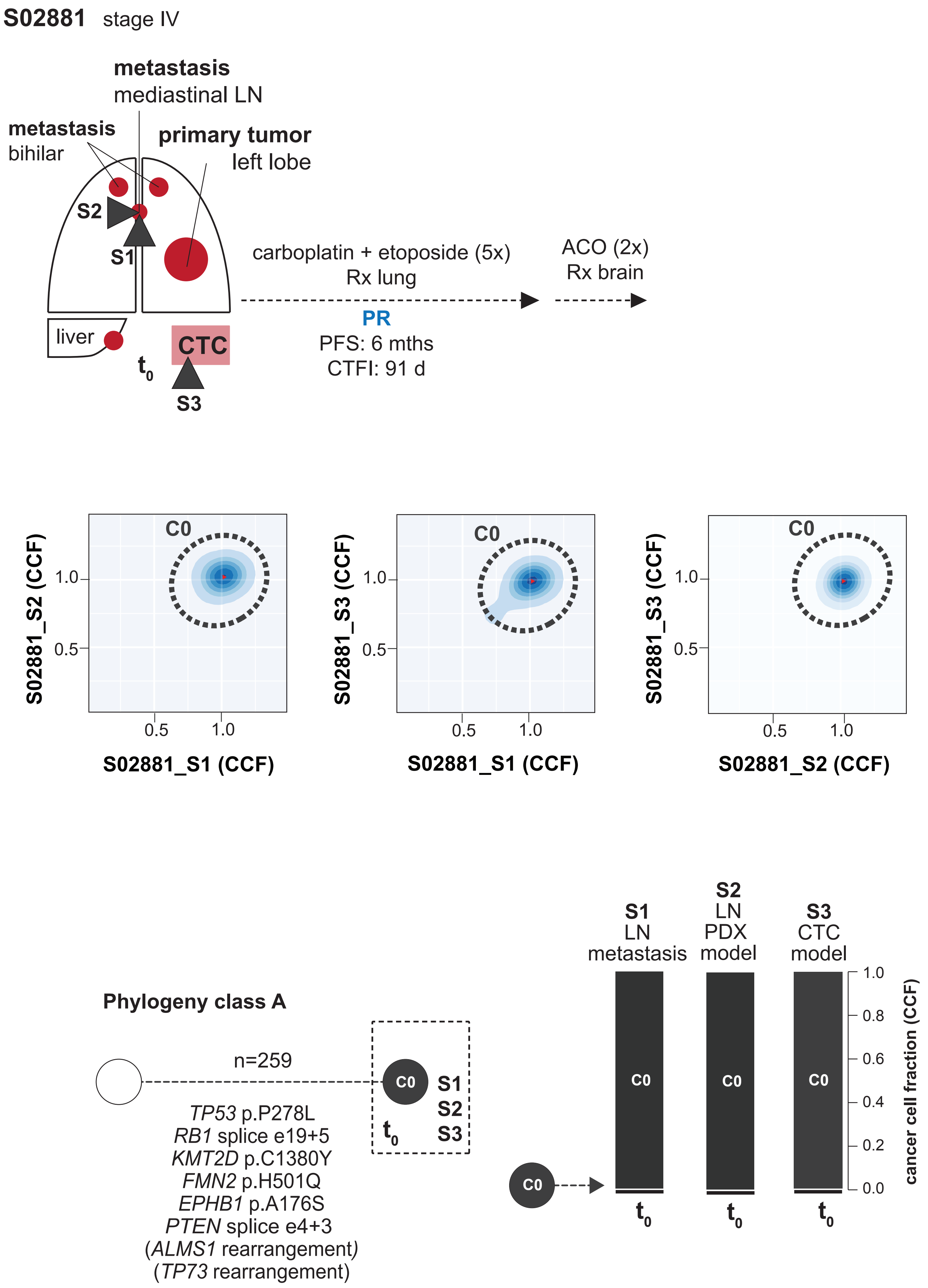

S02881 stage IV
metastasis mediastinal LN
metastasis bihilar
primary tumor left lobe
S2
S1
liver
CTC
t0
S3
carboplatin + etoposide (5x)
Rx lung
ACO (2x)
Rx brain
PR
PFS: 6 mths
CTFI: 91 d
C0
S02881_S2 (CCF)
S02881_S1 (CCF)
S02881_S3 (CCF)
S02881_S1 (CCF)
S02881_S3 (CCF)
S02881_S2 (CCF)
0.5
1.0
Phylogeny class A
n=259
TP53 p.P278L
RB1 splice e19+5
KMT2D p.C1380Y
FMN2 p.H501Q
EPHB1 p.A176S
PTEN splice e4+3
(ALMS1 rearrangement)
(TP73 rearrangement)
C0
S1
S2
S3
t0
S1 LN metastasis
S2 LN PDX model
S3 CTC model
cancer cell fraction (CCF)
0.0
0.2
0.4
0.6
0.8
1.0

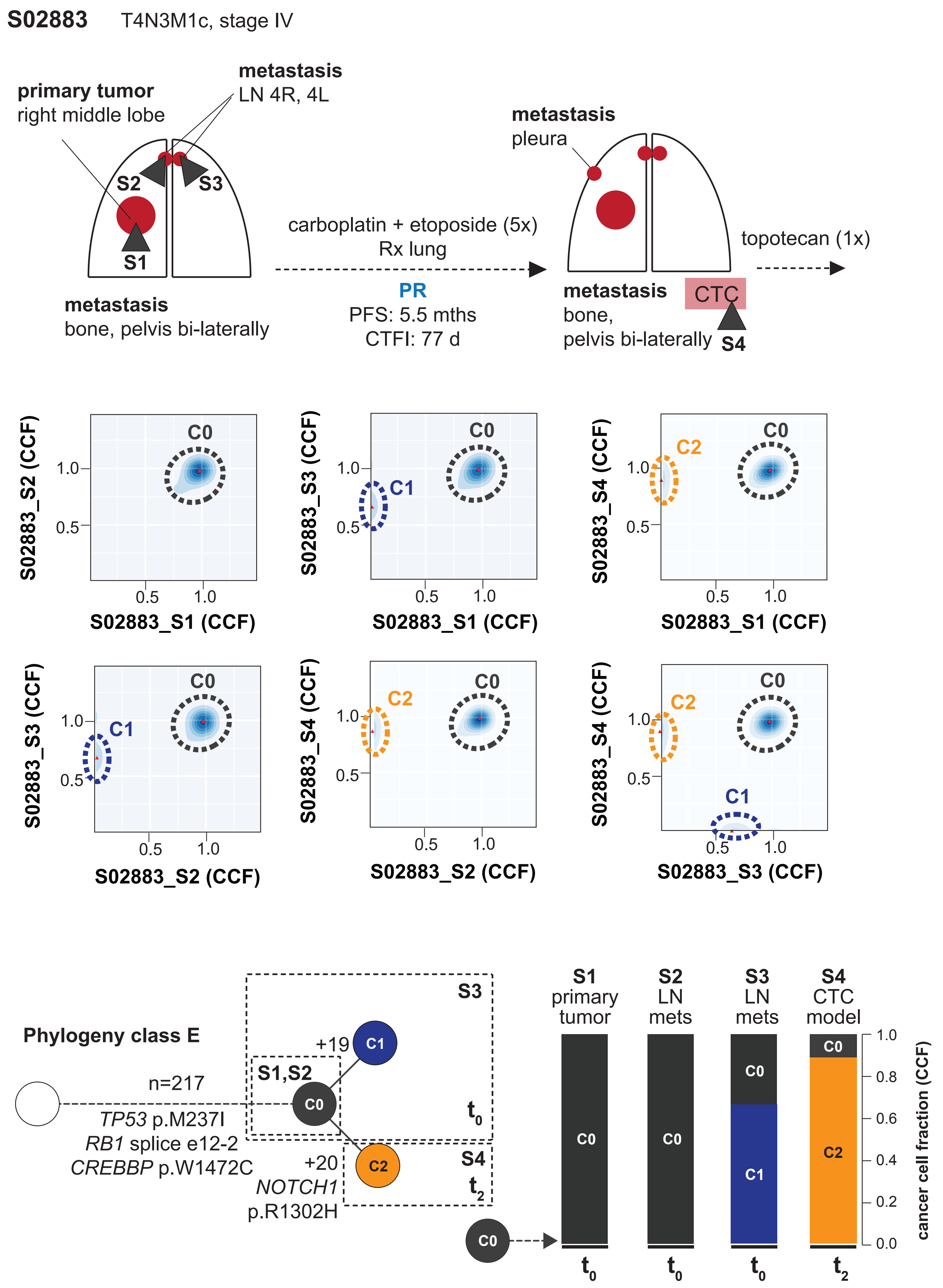

S02883
T4N3M1c, stage IV
primary tumor
right middle lobe
metastasis
LN 4R, 4L
S2
S3
S1
metastasis
bone, pelvis bi-laterally
carboplatin + etoposide (5x)
Rx lung
PR
PFS: 5.5 mths
CTFI: 77 d
metastasis
pleura
metastasis
bone,
pelvis bi-laterally
CTC
S4
topotecan (1x)
S02883_S2 (CCF)
S02883_S1 (CCF)
S02883_S3 (CCF)
S02883_S4 (CCF)
C0
C1
C2
1.0
0.5
S02883_S2 (CCF)
S02883_S3 (CCF)
Phylogeny class E
n=217
TP53 p.M237I
RB1 splice e12-2
CREBBP p.W1472C
S1,S2
S3
t0
+19
C1
C0
+20
C2
NOTCH1
p.R1302H
S4
t2
S1
primary
tumor
S2
LN
mets
S3
LN
mets
S4
CTC
model
cancer cell fraction (CCF)
1.0
0.8
0.6
0.4
0.2
0.0

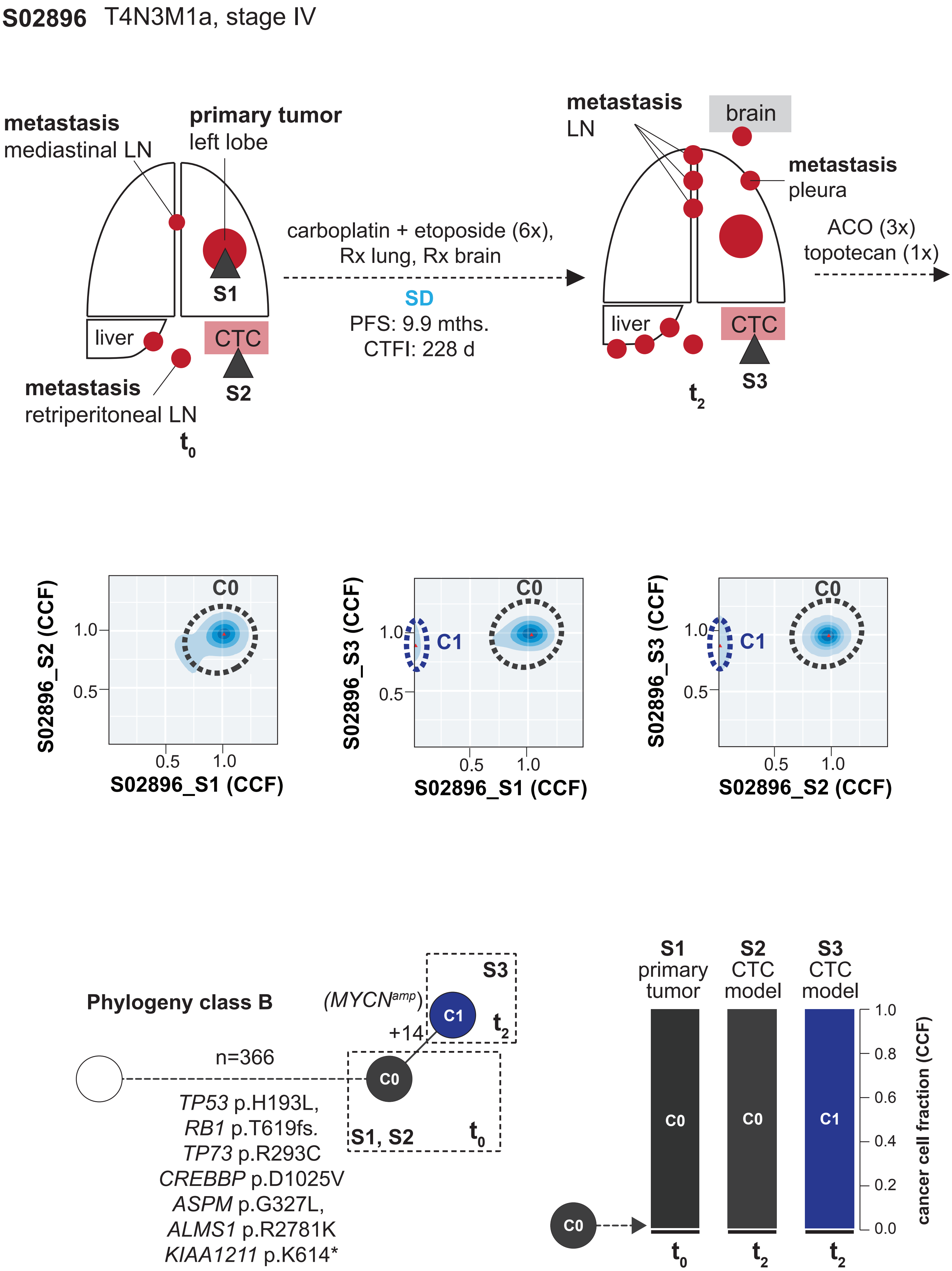

S02896 T4N3M1a, stage IV
metastasis
mediastinal LN
primary tumor
left lobe
S1
liver
CTC
S2
metastasis
retriperitoneal LN
t_0
carboplatin + etoposide (6x),
Rx lung, Rx brain
SD
PFS: 9.9 mths.
CTFI: 228 d
metastasis
LN
brain
metastasis
pleura
liver
CTC
S3
t_2
ACO (3x)
topotecan (1x)
S02896_S2 (CCF)
S02896_S1 (CCF)
C0
1.0
0.5
S02896_S3 (CCF)
S02896_S1 (CCF)
C0
C1
1.0
0.5
S02896_S3 (CCF)
S02896_S2 (CCF)
C0
C1
1.0
0.5
Phylogeny class B
n=366
TP53 p.H193L,
RB1 p.T619fs.
TP73 p.R293C
CREBBP p.D1025V
ASPM p.G327L,
ALMS1 p.R2781K
KIAA1211 p.K614*
(*MYCN*amp)
+14
C0
C1
S3
t_2
S1, S2
t_0
S1
primary
tumor
S2
CTC
model
S3
CTC
model
C0
C0
C1
C0
t_0
t_2
t_2
1.0
0.8
0.6
0.4
0.2
0.0
cancer cell fraction (CCF)

S02906 stage IV

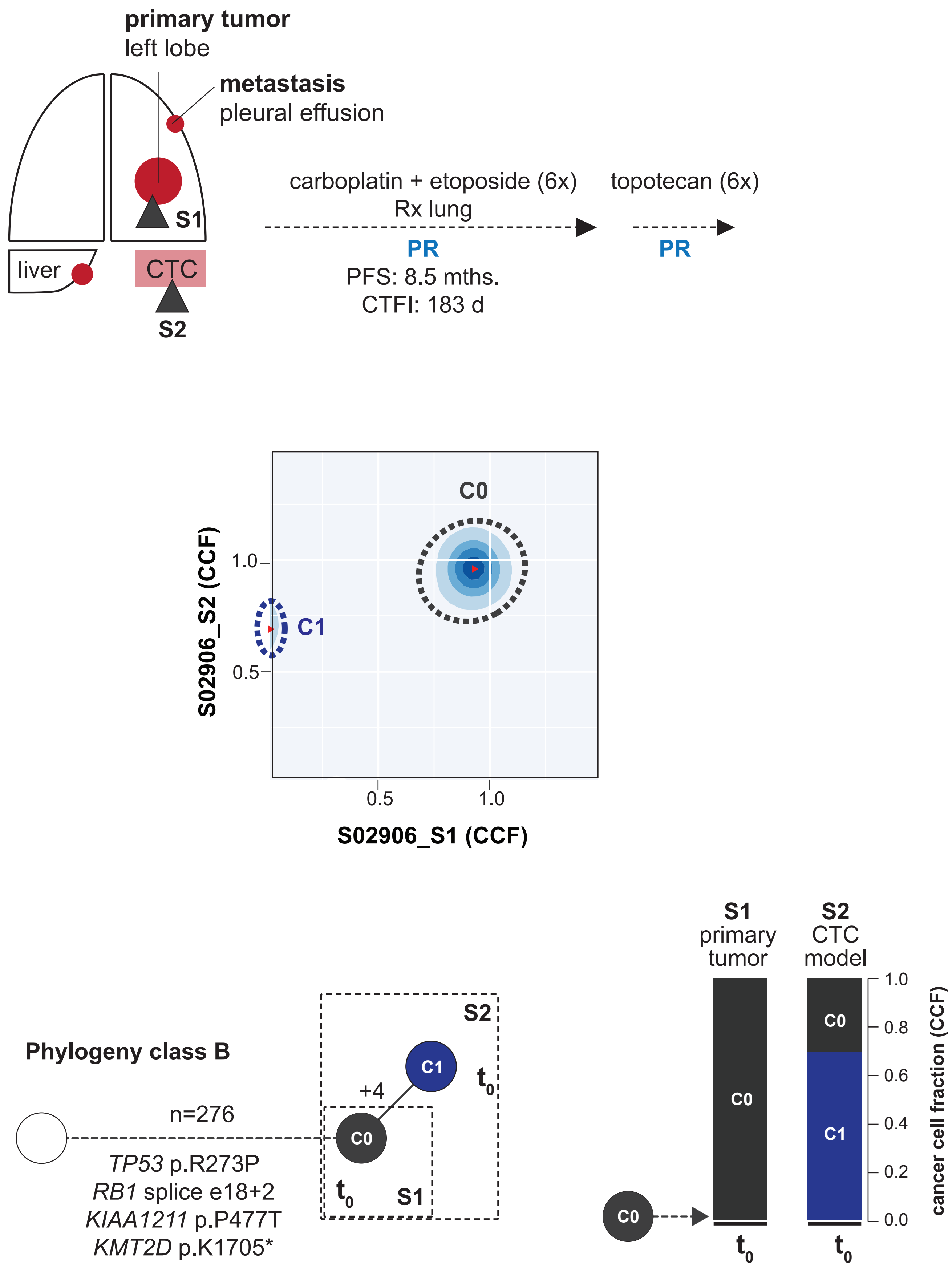

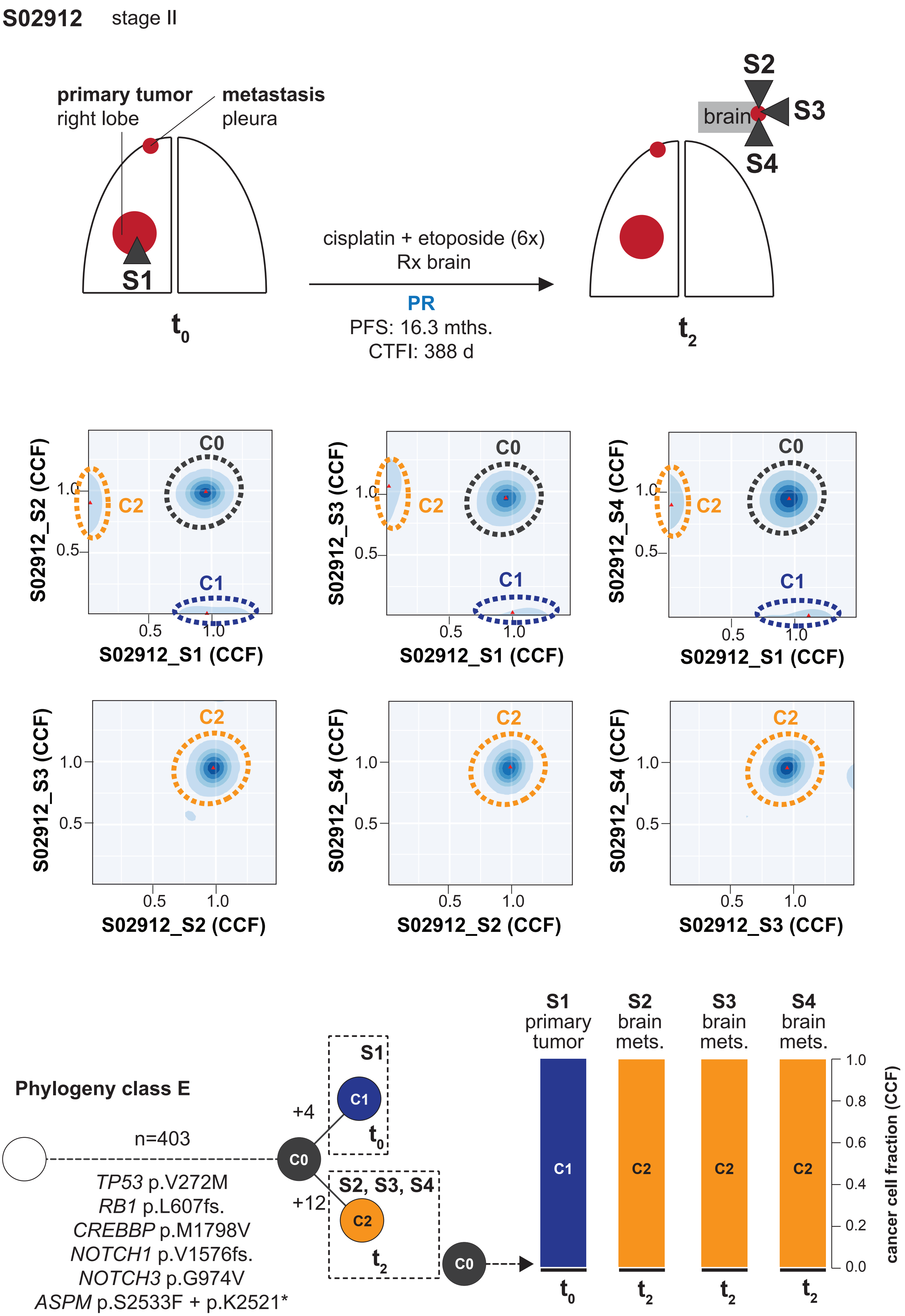
S02912 stage II
primary tumor
right lobe
metastasis
pleura
S1
t0
cisplatin + etoposide (6x)
Rx brain
PR
PFS: 16.3 mths.
CTFI: 388 d
brain
S2
S3
S4
t2
C0
C1
C2
S02912_S1 (CCF)
S02912_S2 (CCF)
S02912_S3 (CCF)
S02912_S4 (CCF)
0.5
1.0
Phylogeny class E
n=403
TP53 p.V272M
RB1 p.L607fs.
CREBBP p.M1798V
NOTCH1 p.V1576fs.
NOTCH3 p.G974V
ASPM p.S2533F + p.K2521*
+4
+12
S1
S2, S3, S4
S1 primary tumor
S2 brain mets.
S3 brain mets.
S4 brain mets.
cancer cell fraction (CCF)
0.0
0.2
0.4
0.6
0.8
1.0

S02917 T4N2M1, stage IV

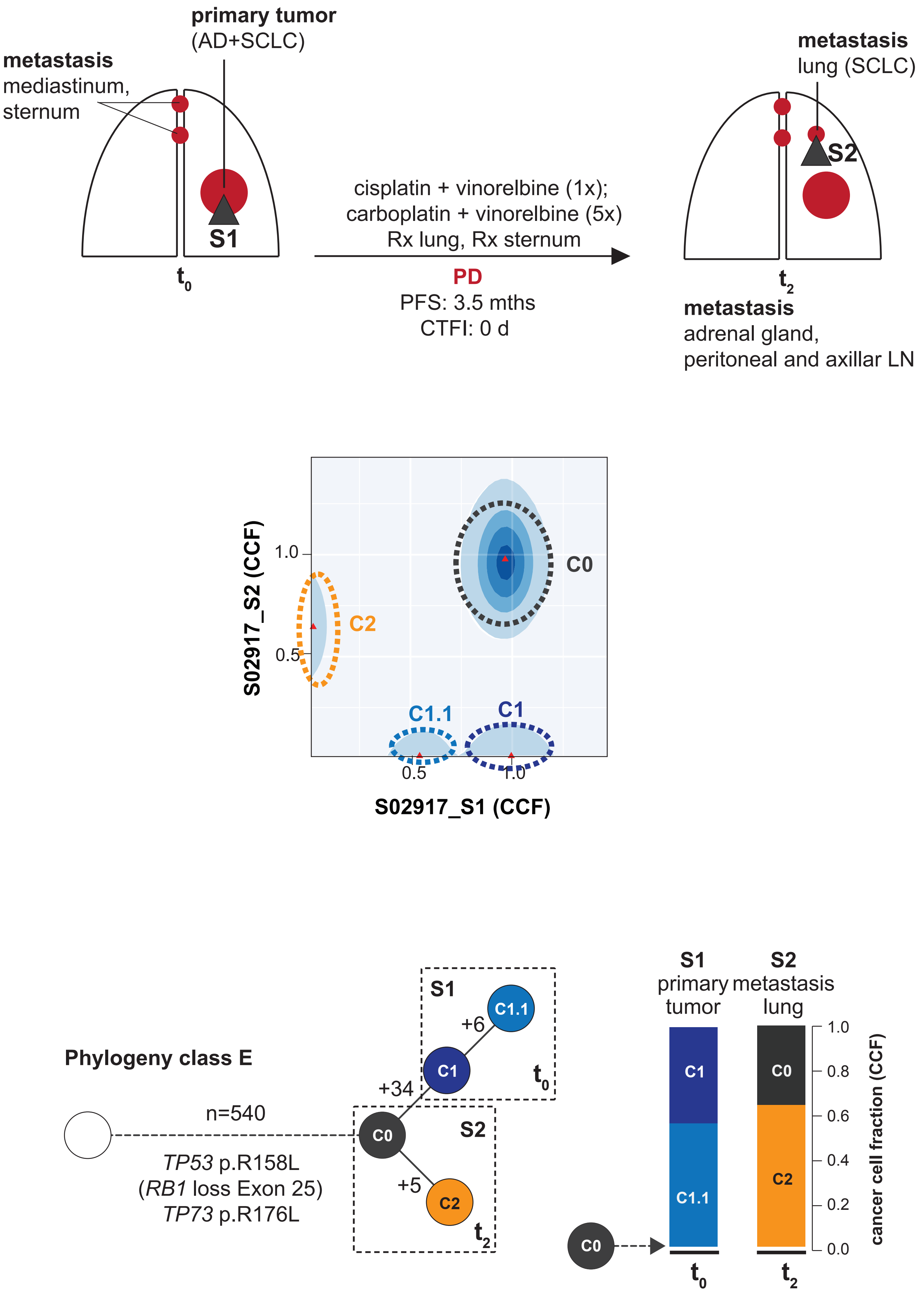

S02976 T2N2M1b, stage IV

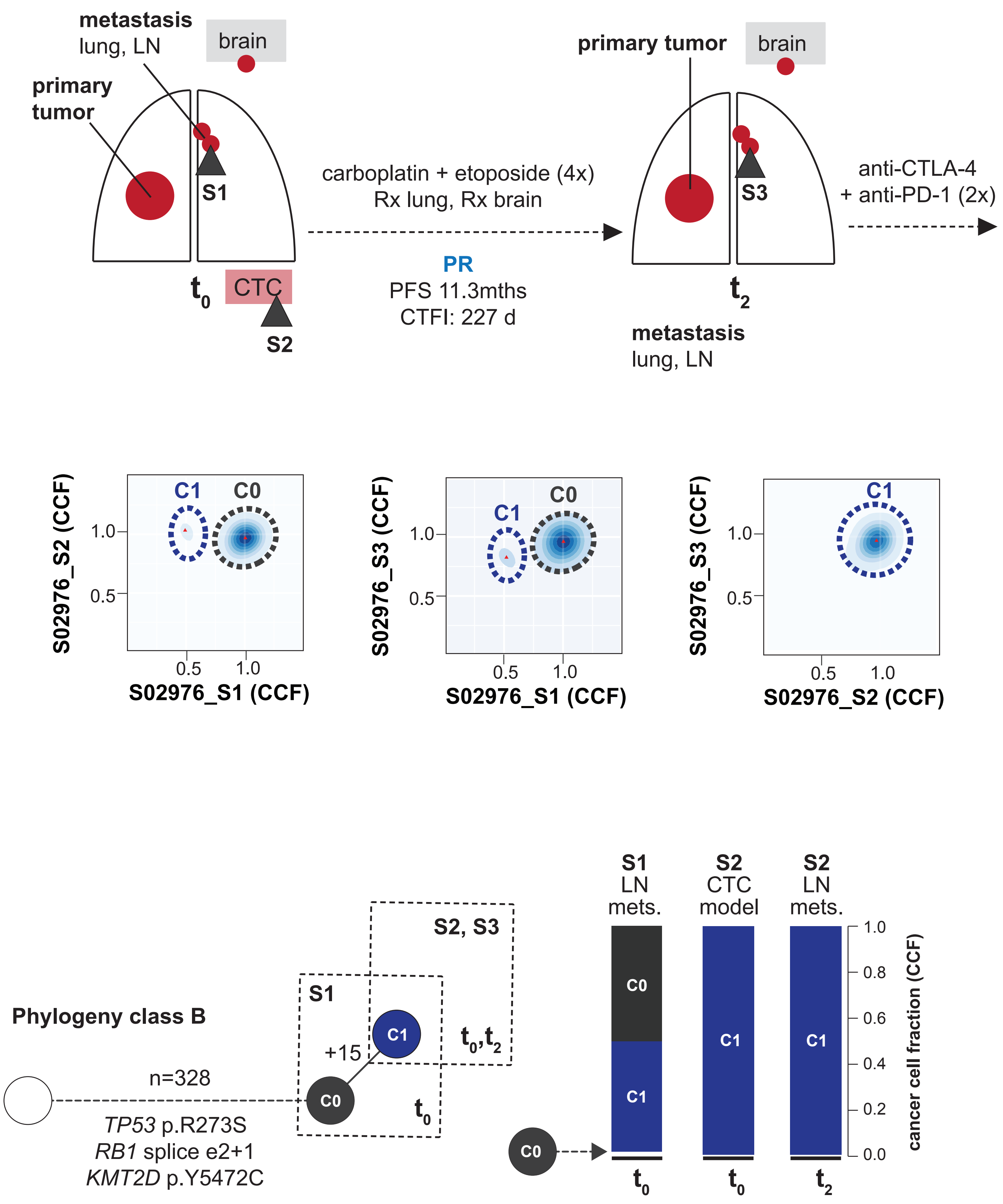

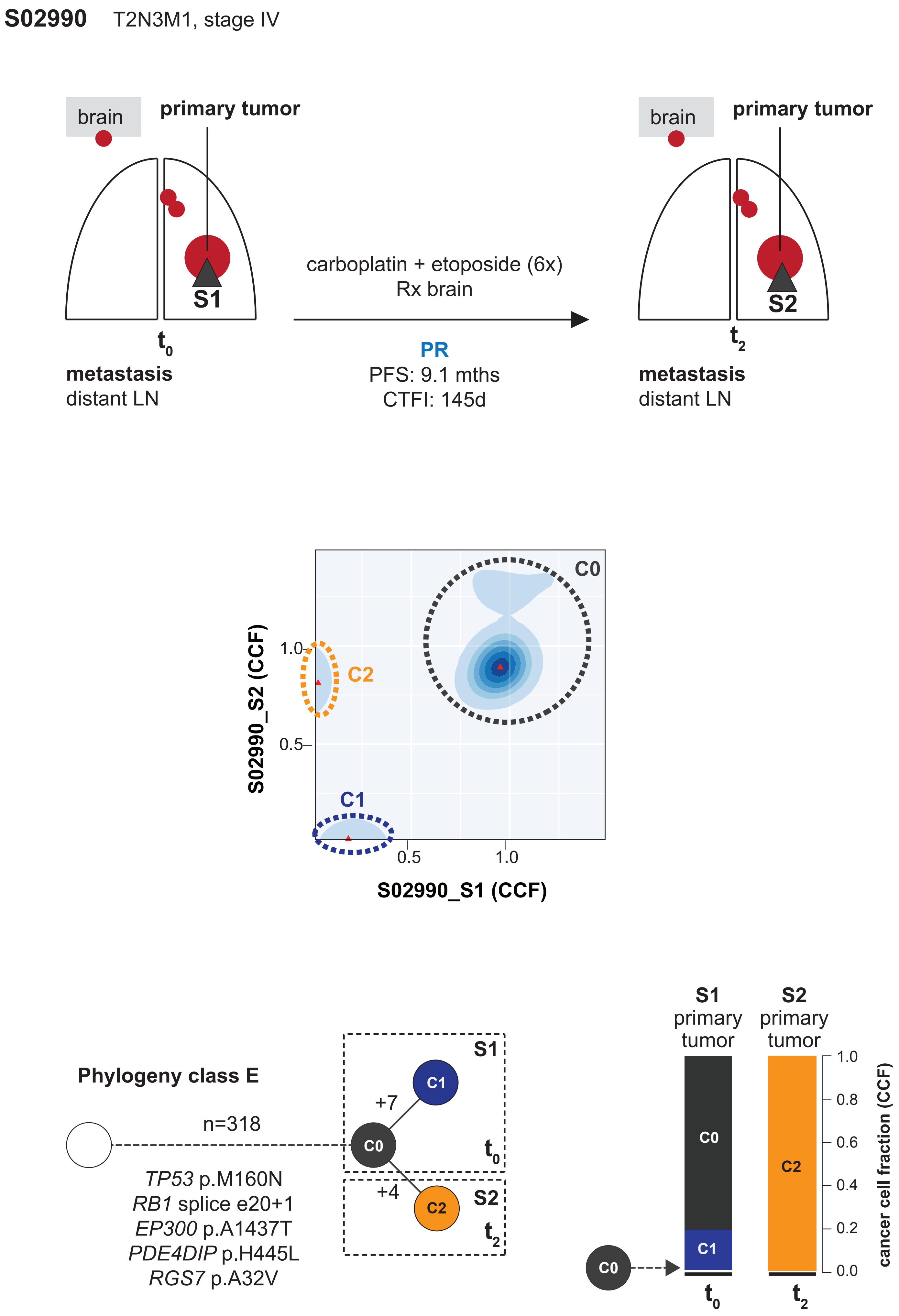

S02990 T2N3M1, stage IV
brain
primary tumor
S1
t_0
metastasis
distant LN
carboplatin + etoposide (6x)
Rx brain
PR
PFS: 9.1 mths
CTFI: 145d
brain
primary tumor
S2
t_2
metastasis
distant LN
C0
C2
C1
1.0
0.5
0.5
1.0
S02990_S2 (CCF)
S02990_S1 (CCF)
Phylogeny class E
n=318
TP53 p.M160N
RB1 splice e20+1
EP300 p.A1437T
PDE4DIP p.H445L
RGS7 p.A32V
S1
+7
C1
C0
t_0
+4
C2
S2
t_2
S1
primary
tumor
S2
primary
tumor
C0
C1
C2
C0
1.0
0.8
0.6
0.4
0.2
0.0
cancer cell fraction (CCF)
t_0
t_2

S02991 T4NXM1, stage IV

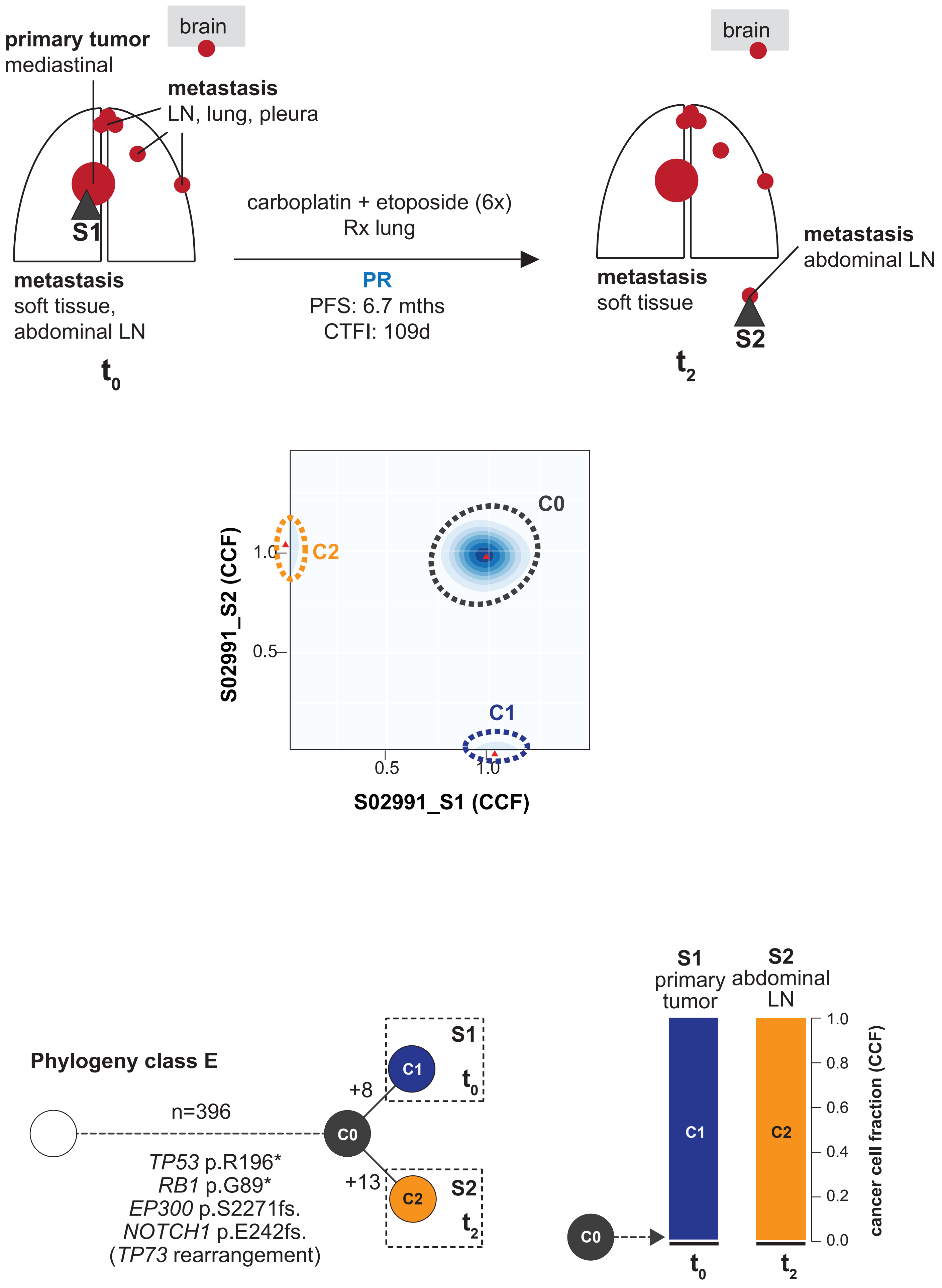

S03017 stage II

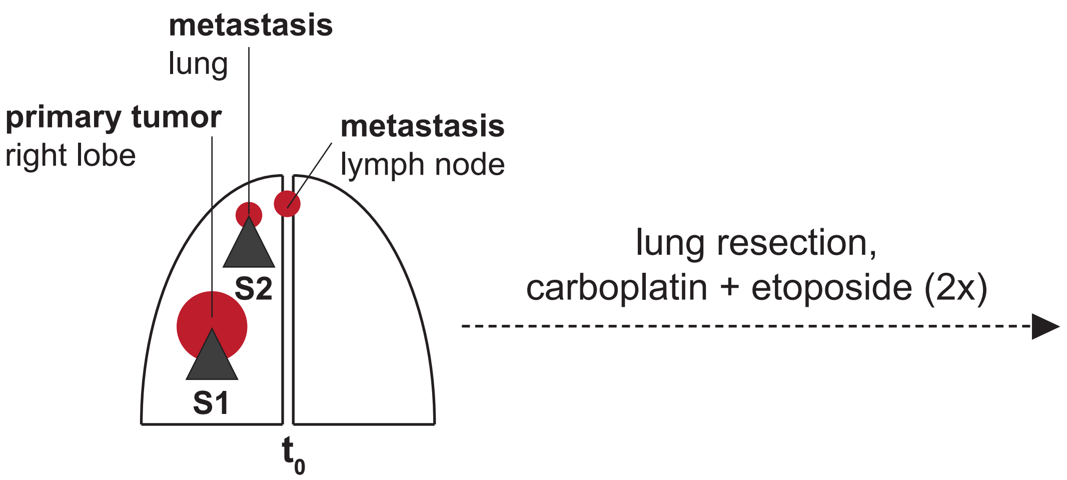

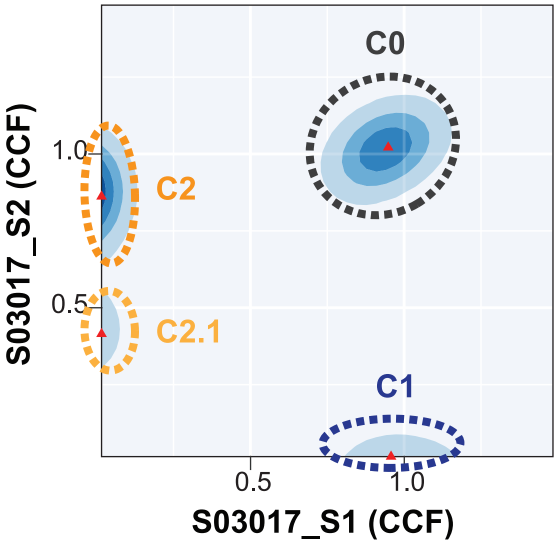

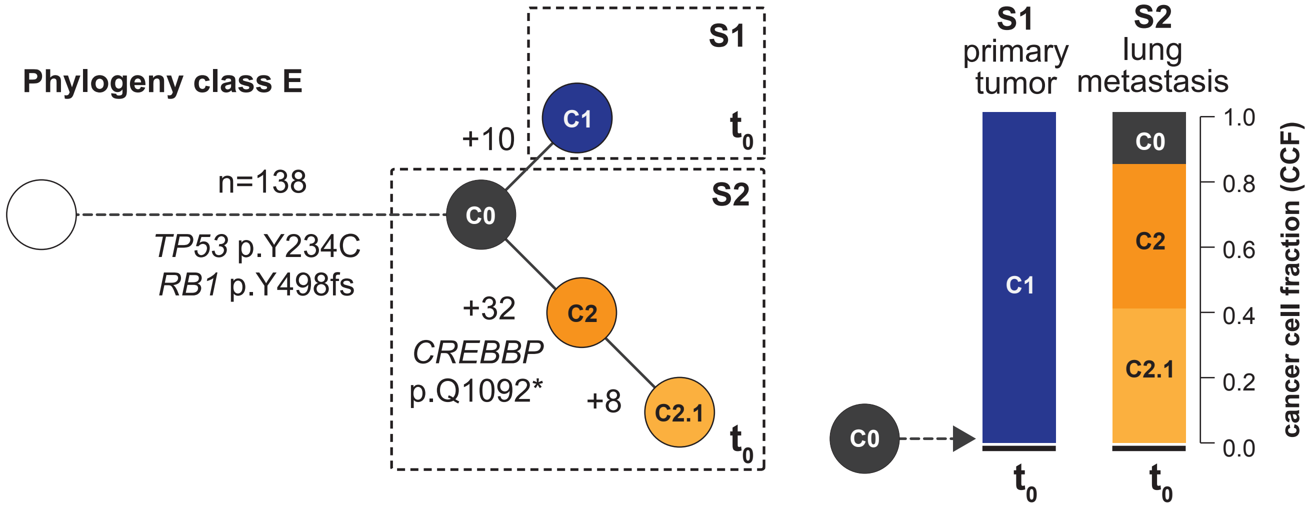

S03032 T4N1M1, stage IV

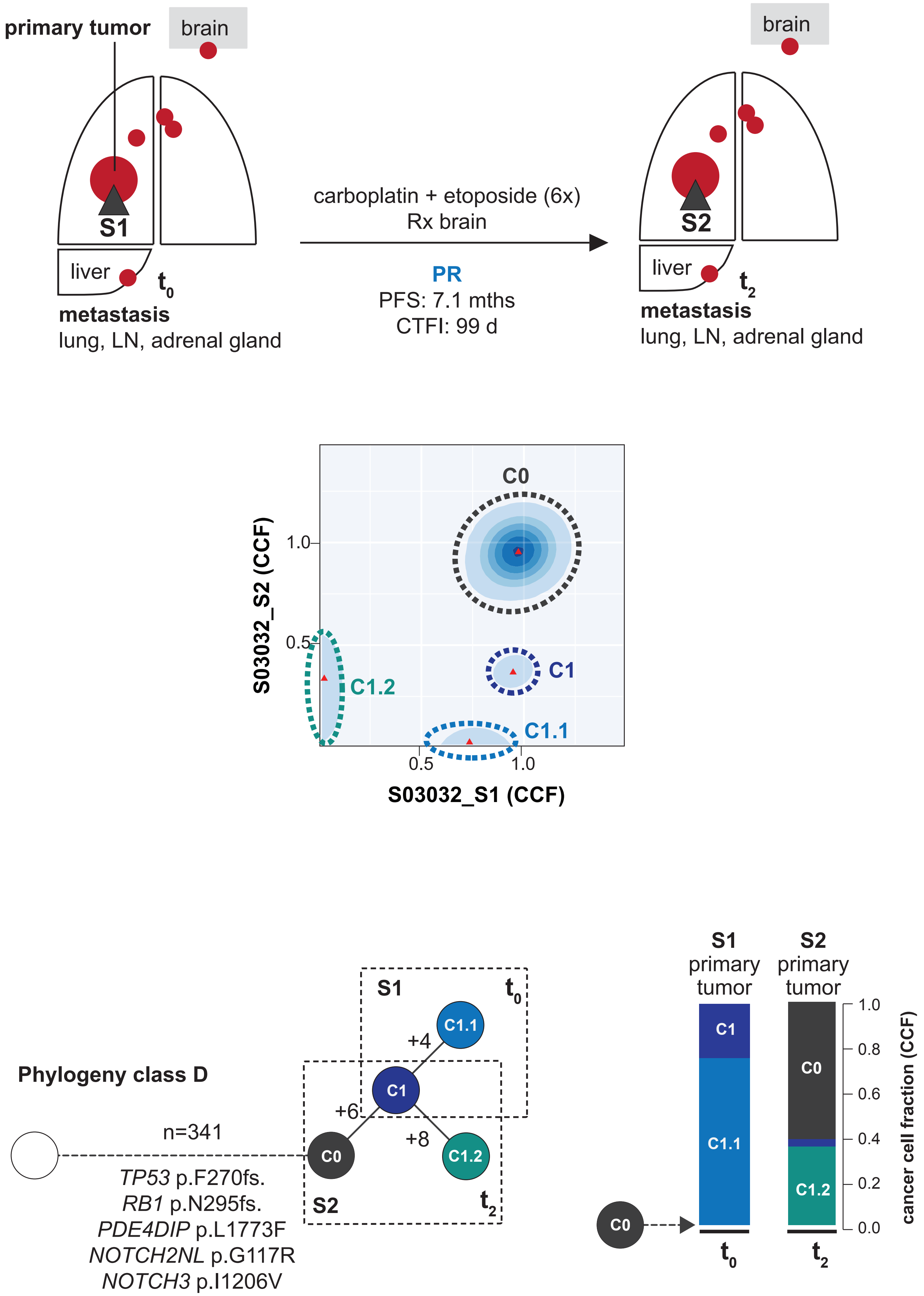

S03056 T3N2M0, stage III

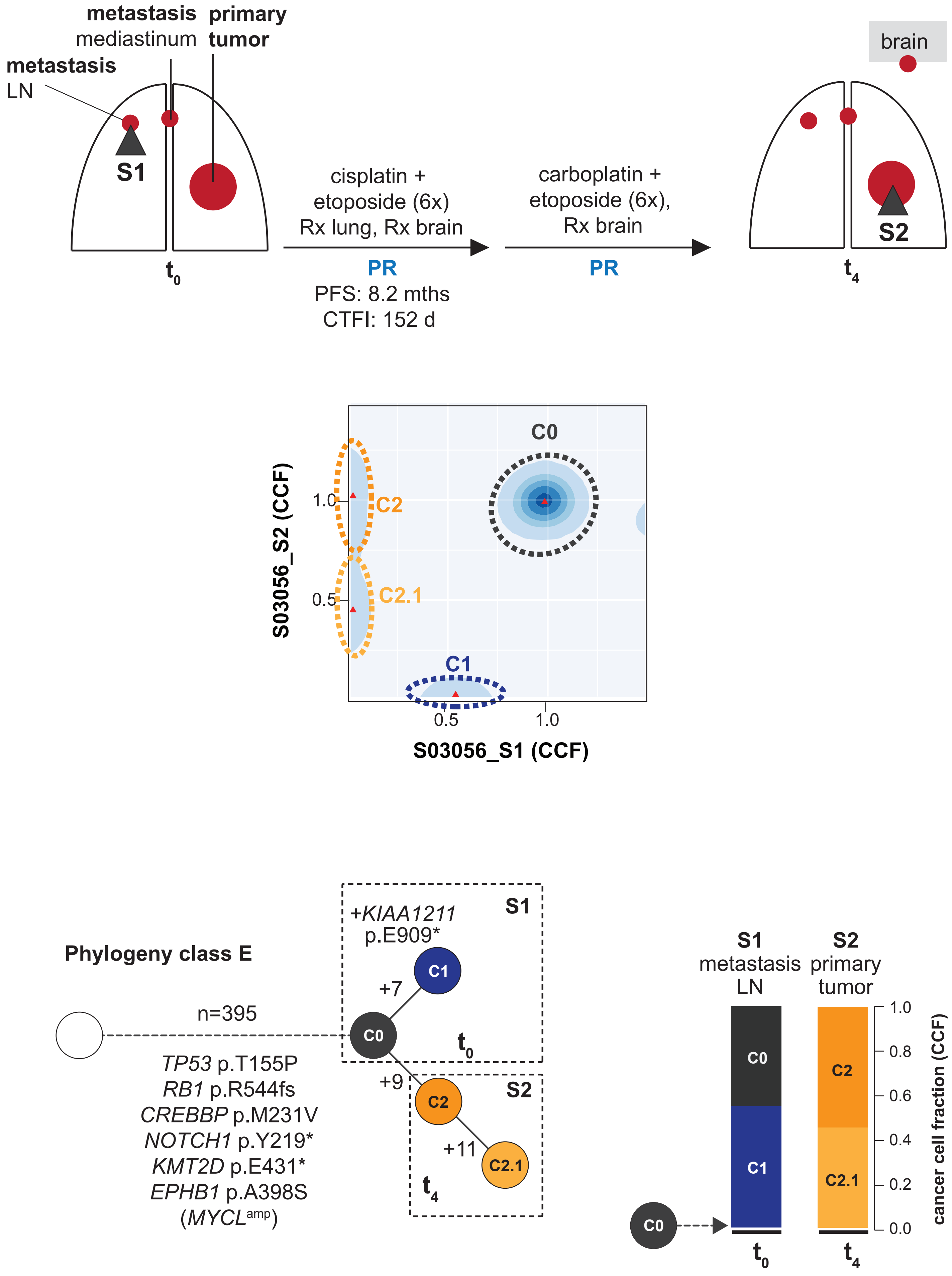

S03104 T3N2M0, stage III

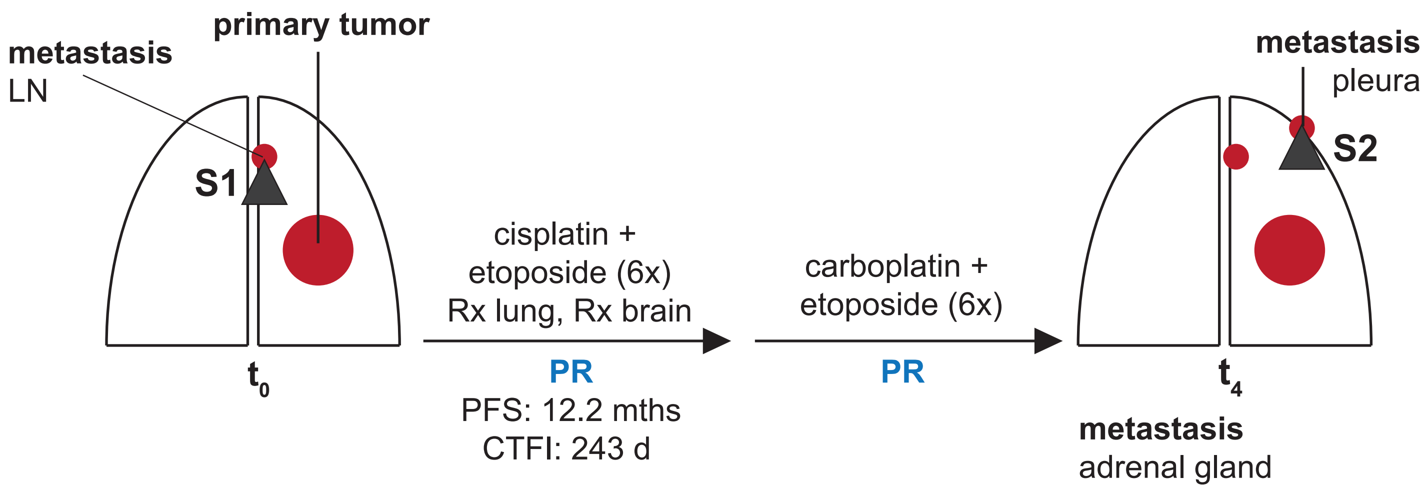

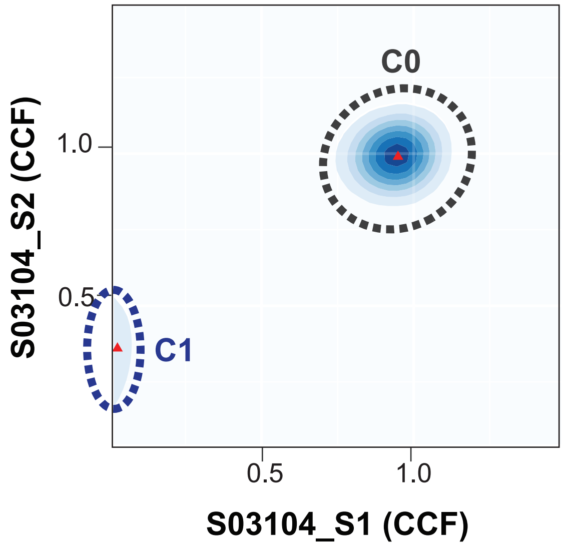

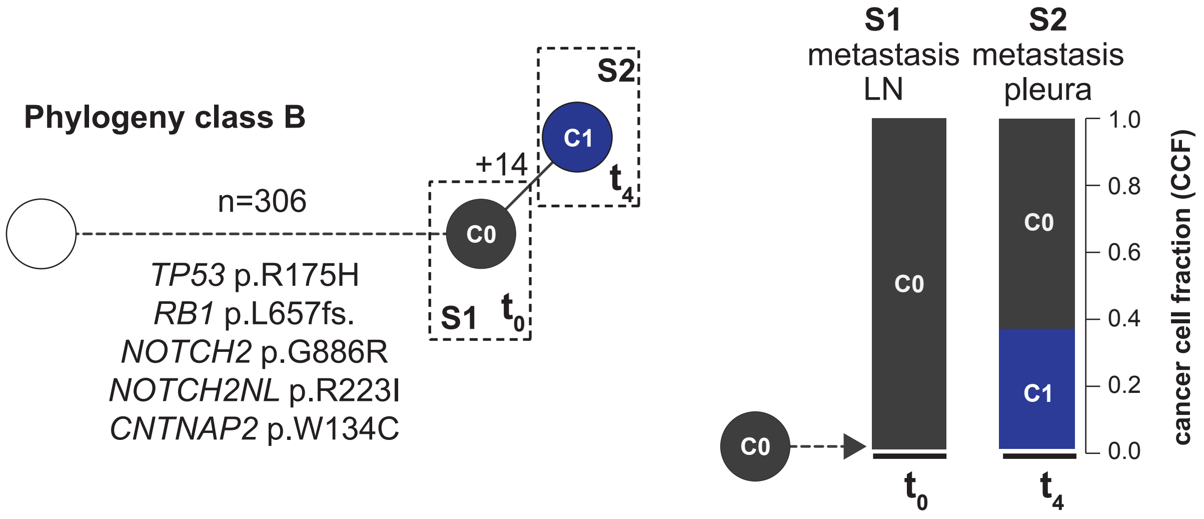

S03105 stage I

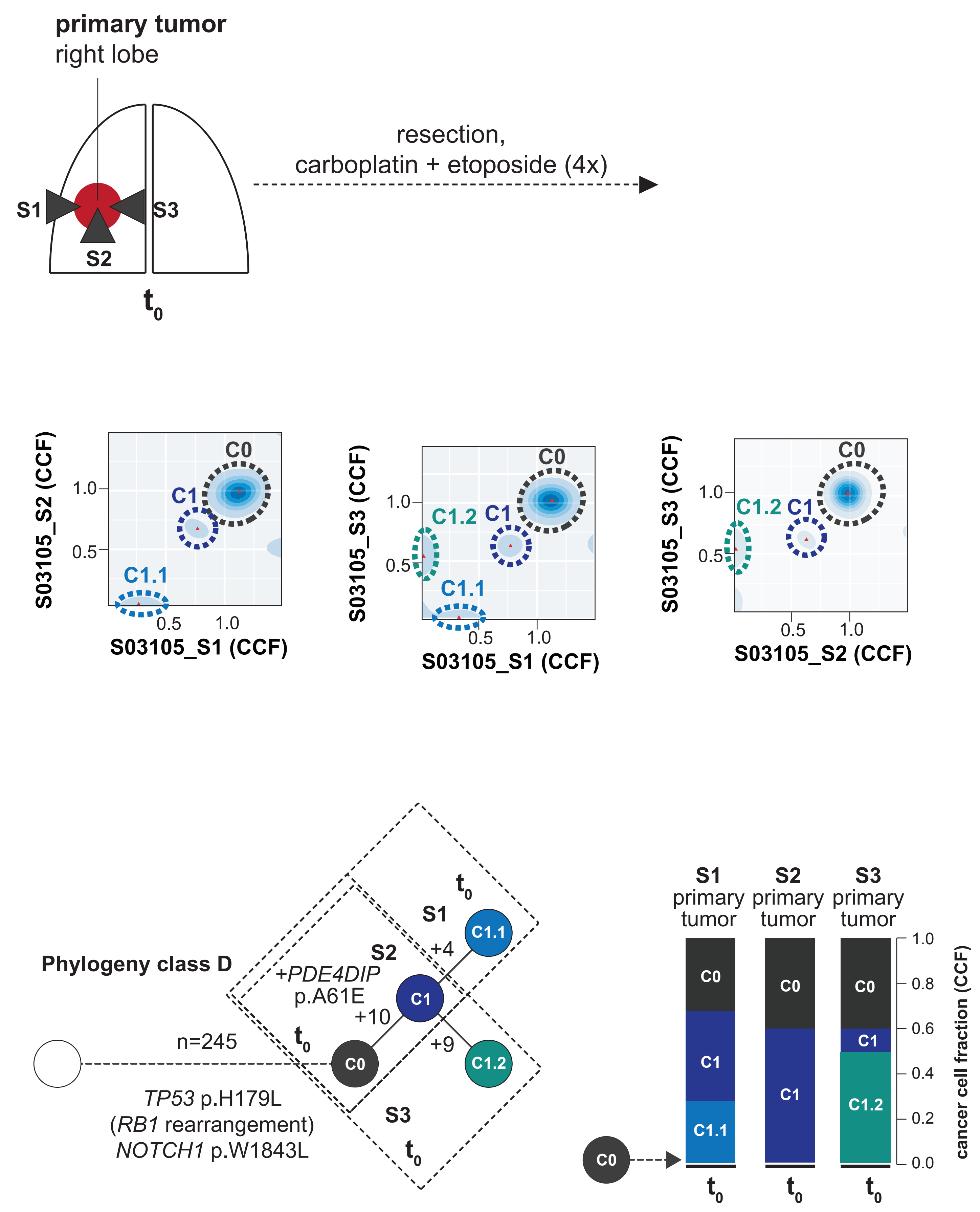

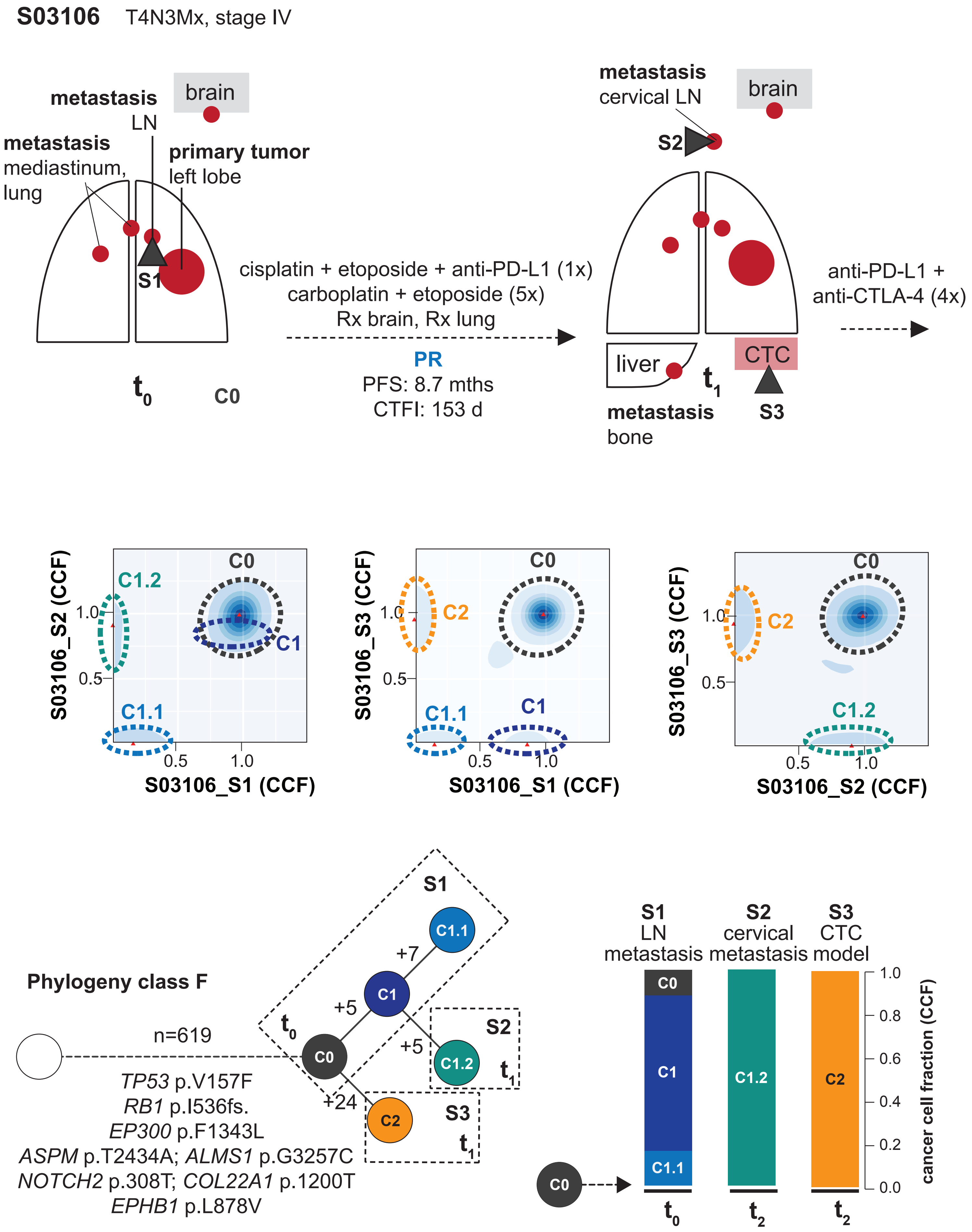
S03106 T4N3Mx, stage IV
metastasis
LN
brain
metastasis
mediastinum,
lung
primary tumor
left lobe
S1
t0
C0
cisplatin + etoposide + anti-PD-L1 (1x)
carboplatin + etoposide (5x)
Rx brain, Rx lung
PR
PFS: 8.7 mths
CTFI: 153 d
metastasis
cervical LN
brain
S2
liver
t1
CTC
S3
metastasis
bone
anti-PD-L1 +
anti-CTLA-4 (4x)
C0
C1.2
C1
C1.1
0.5
1.0
S03106_S2 (CCF)
S03106_S1 (CCF)
C0
C2
C1.1
C1
S03106_S3 (CCF)
S03106_S1 (CCF)
C0
C2
C1.2
S03106_S3 (CCF)
S03106_S2 (CCF)
Phylogeny class F
n=619
TP53 p.V157F
RB1 p.I536fs.
EP300 p.F1343L
ASPM p.T2434A; ALMS1 p.G3257C
NOTCH2 p.308T; COL22A1 p.1200T
EPHB1 p.L878V
C0
t0
+5
C1
+7
C1.1
S1
+5
C1.2
S2
t1
+24
C2
S3
t1
S1
LN
metastasis
S2
cervical
metastasis
S3
CTC
model
C0
C1
C1.1
C1.2
C2
t0
t2
t2
1.0
0.8
0.6
0.4
0.2
0.0
cancer cell fraction (CCF)

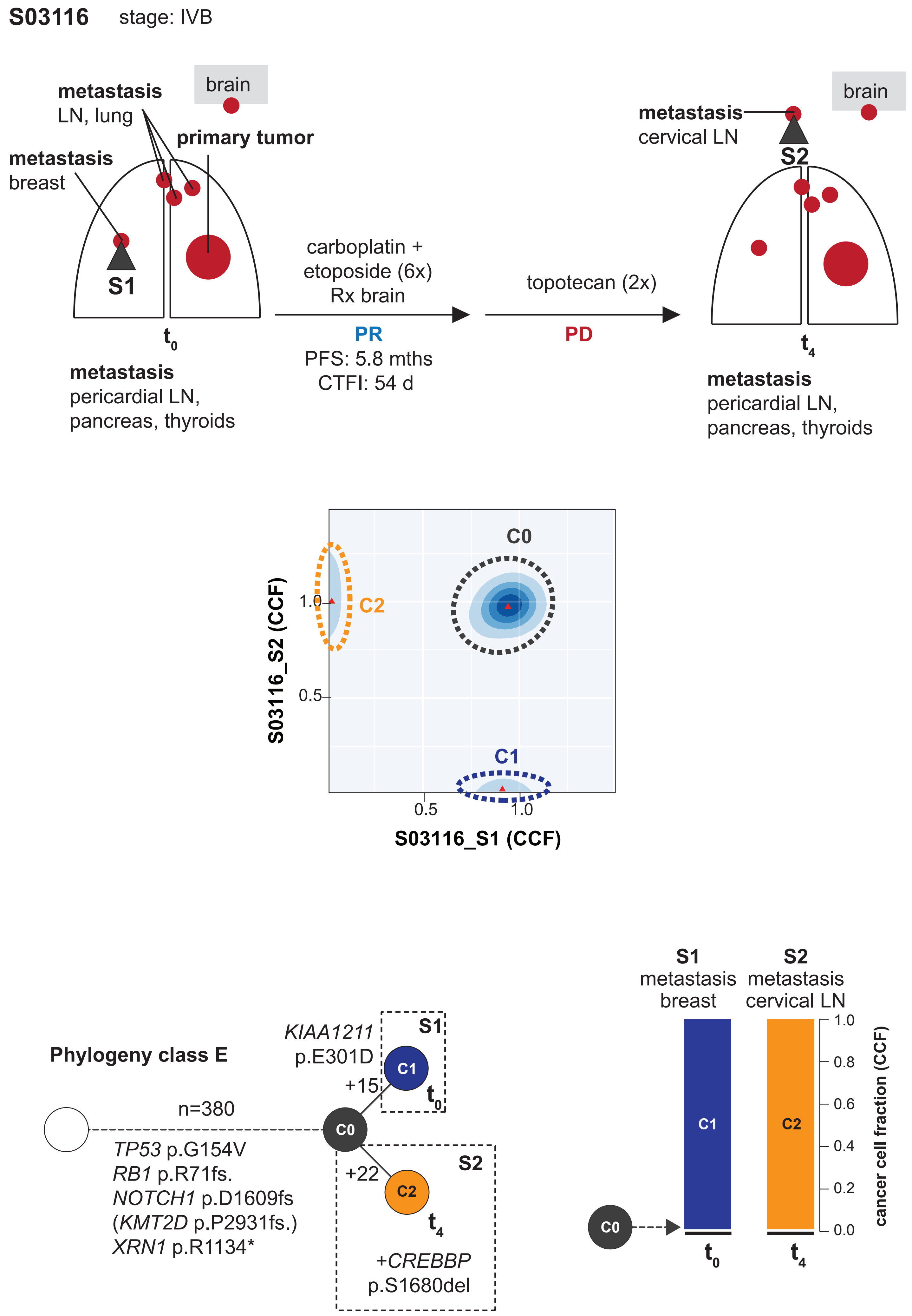
S03116 stage: IVB
metastasis
LN, lung
brain
primary tumor
metastasis
breast
S1
t_0
metastasis
pericardial LN,
pancreas, thyroids
carboplatin +
etoposide (6x)
Rx brain
PR
PFS: 5.8 mths
CTFI: 54 d
topotecan (2x)
PD
metastasis
cervical LN
brain
S2
t_4
metastasis
pericardial LN,
pancreas, thyroids
C0
C2
C1
1.0
0.5
0.5
1.0
S03116_S2 (CCF)
S03116_S1 (CCF)
Phylogeny class E
n=380
TP53 p.G154V
RB1 p.R71fs.
NOTCH1 p.D1609fs
(KMT2D p.P2931fs.)
XRN1 p.R1134*
C0
KIAA1211
p.E301D
+15
C1
S1
t_0
+22
C2
S2
t_4
+CREBBP
p.S1680del
S1
metastasis
breast
S2
metastasis
cervical LN
C1
C2
C0
t_0
t_4
1.0
0.8
0.6
0.4
0.2
0.0
cancer cell fraction (CCF)

S03119 T2N2M1, stage IV

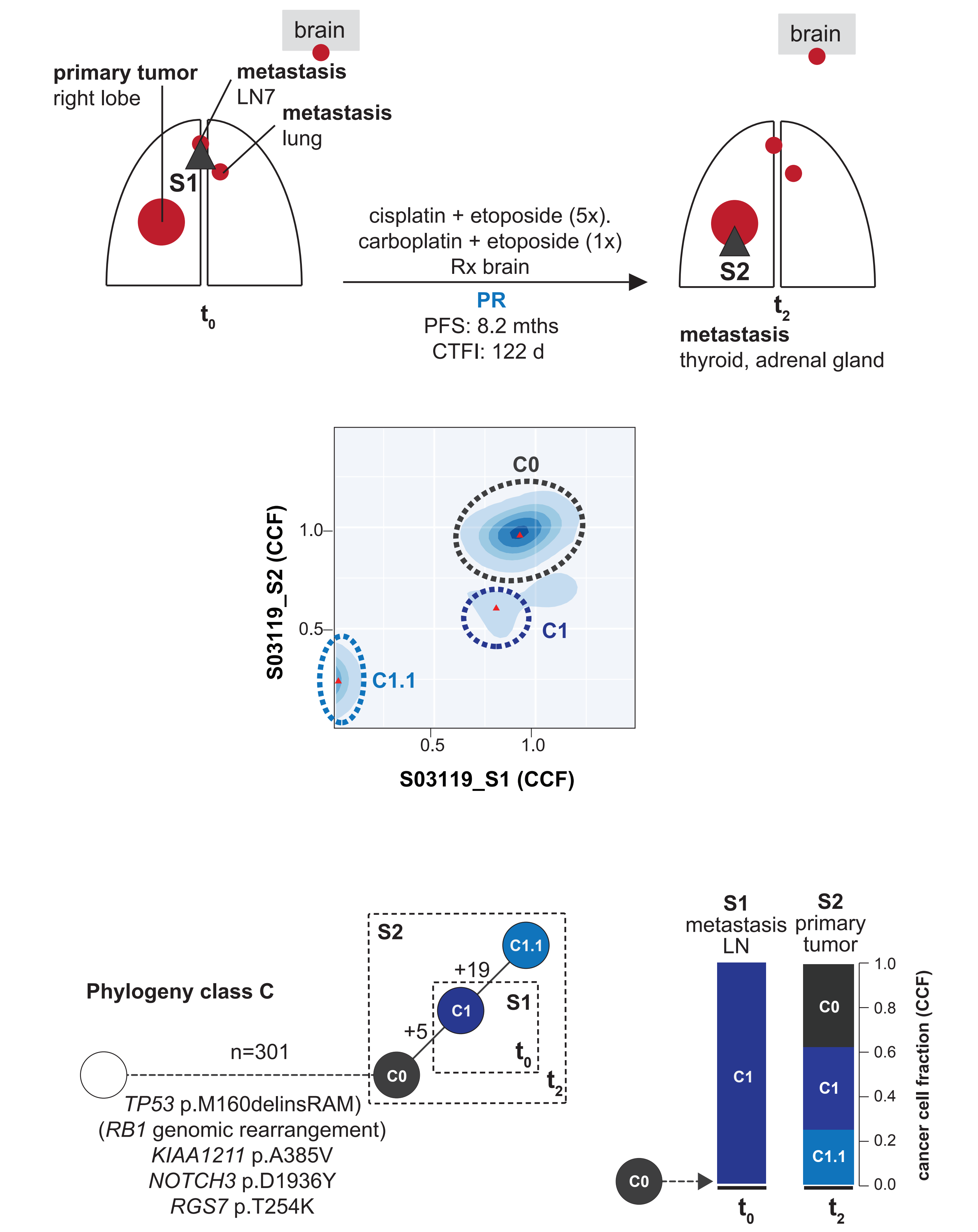

S03144 T1N0M1, stage IV

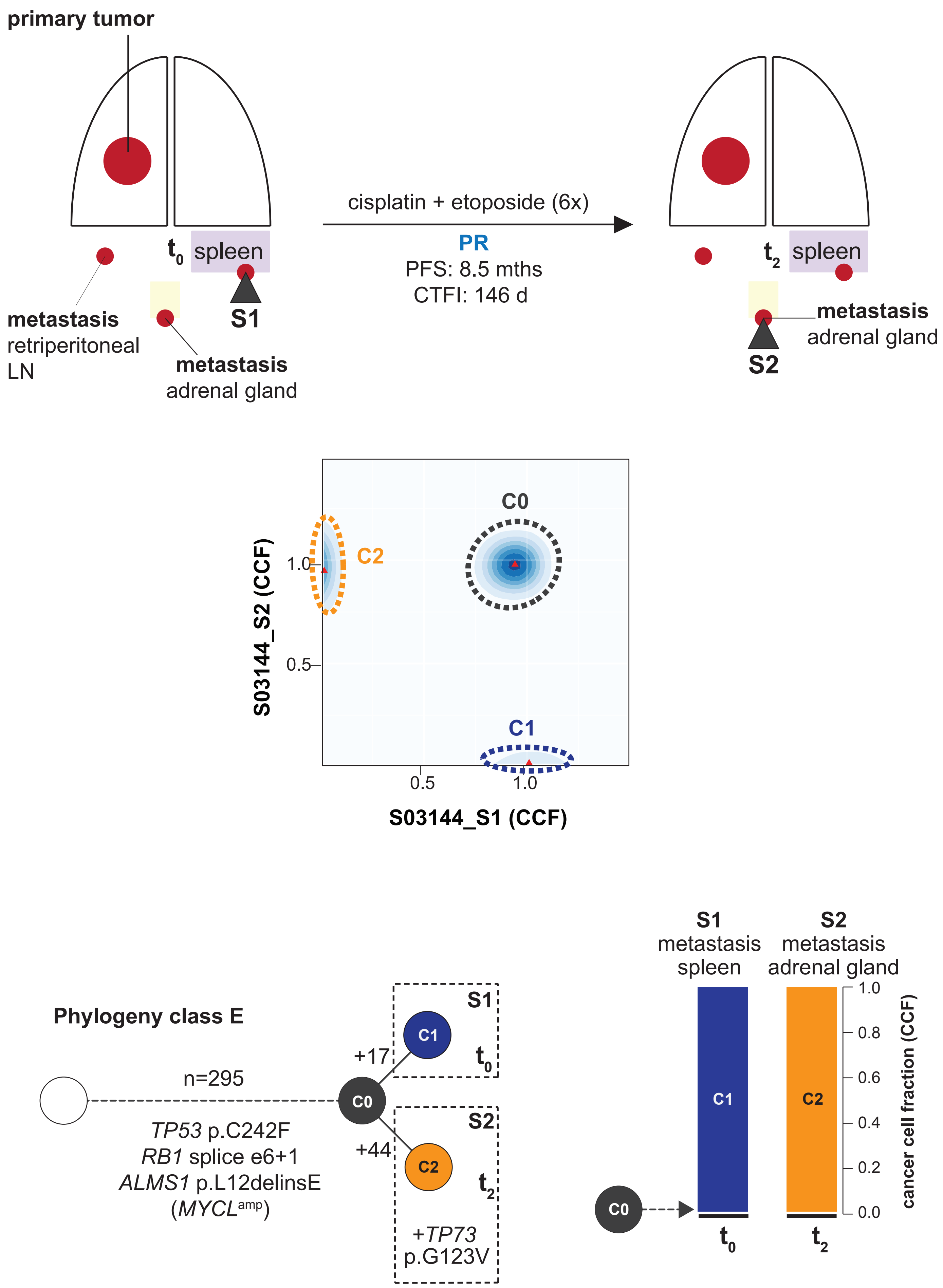

S03199 stage IV

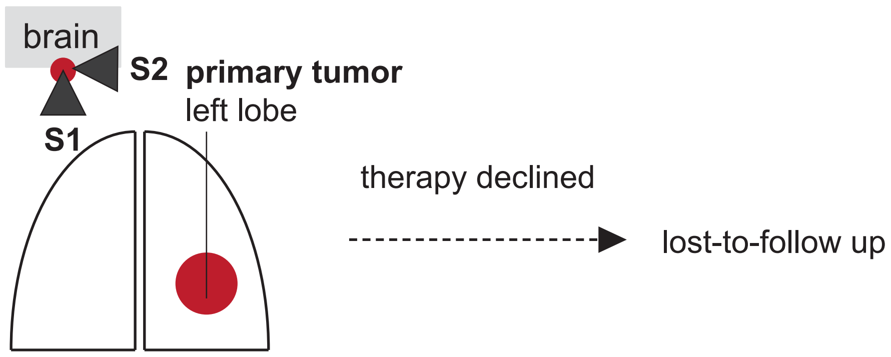

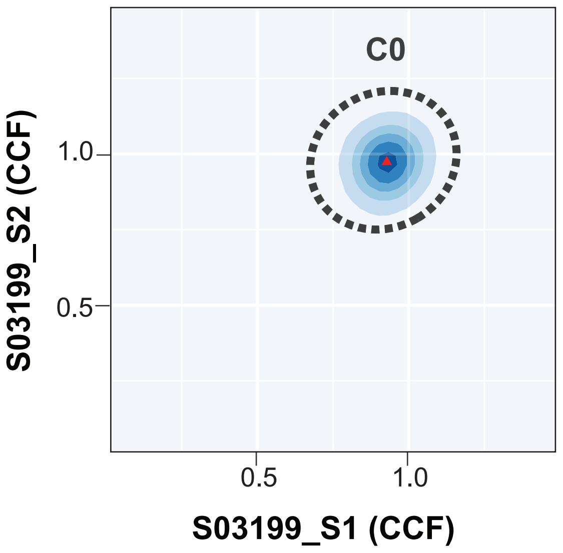

Phylogeny class A

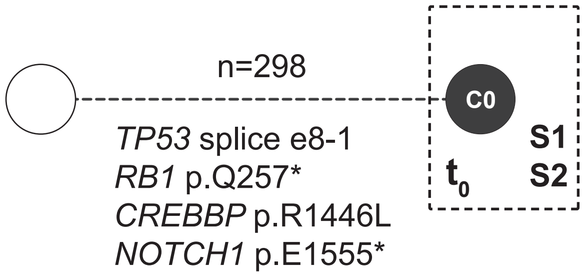

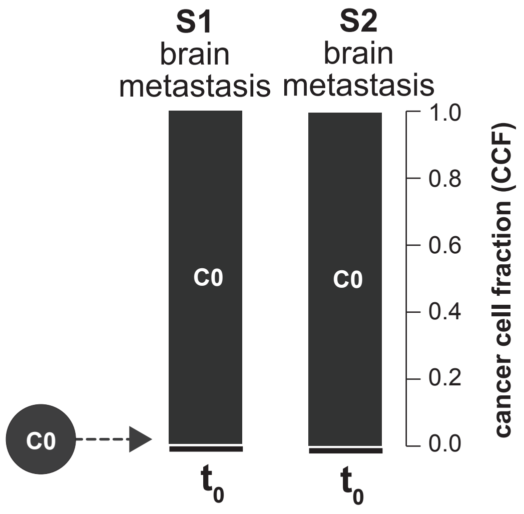

S03205 stage II

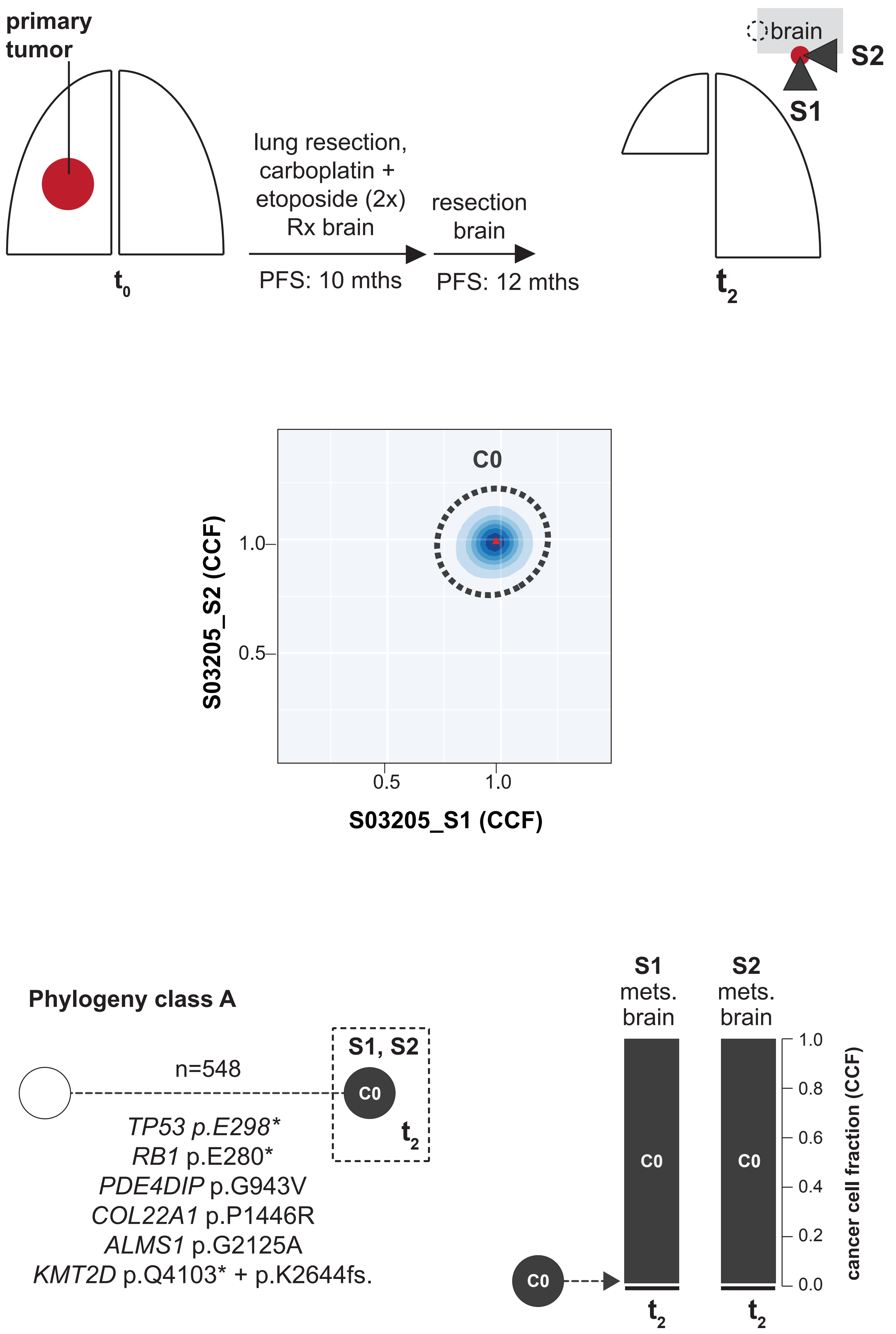

S03207 T2N2M0, stage III

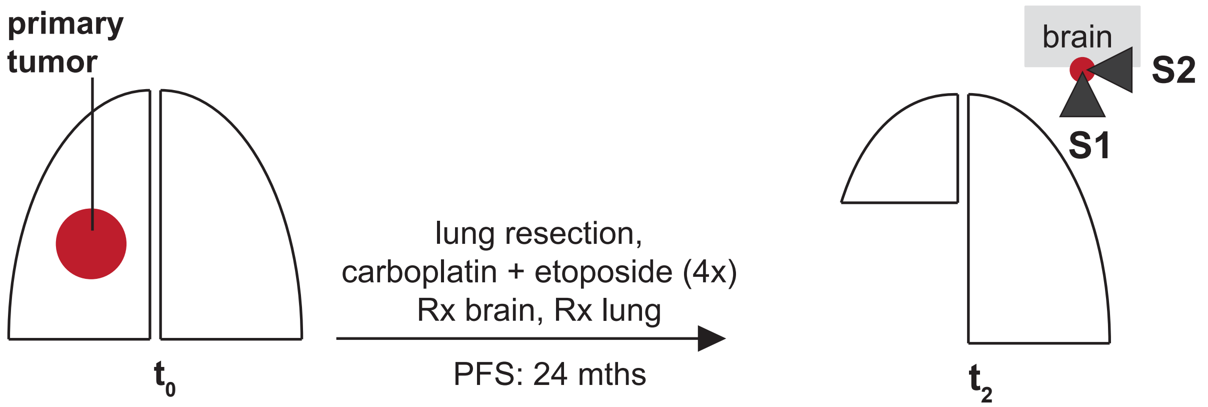

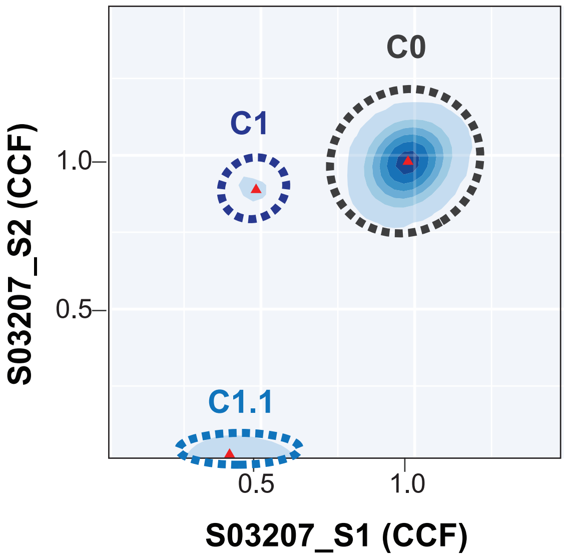

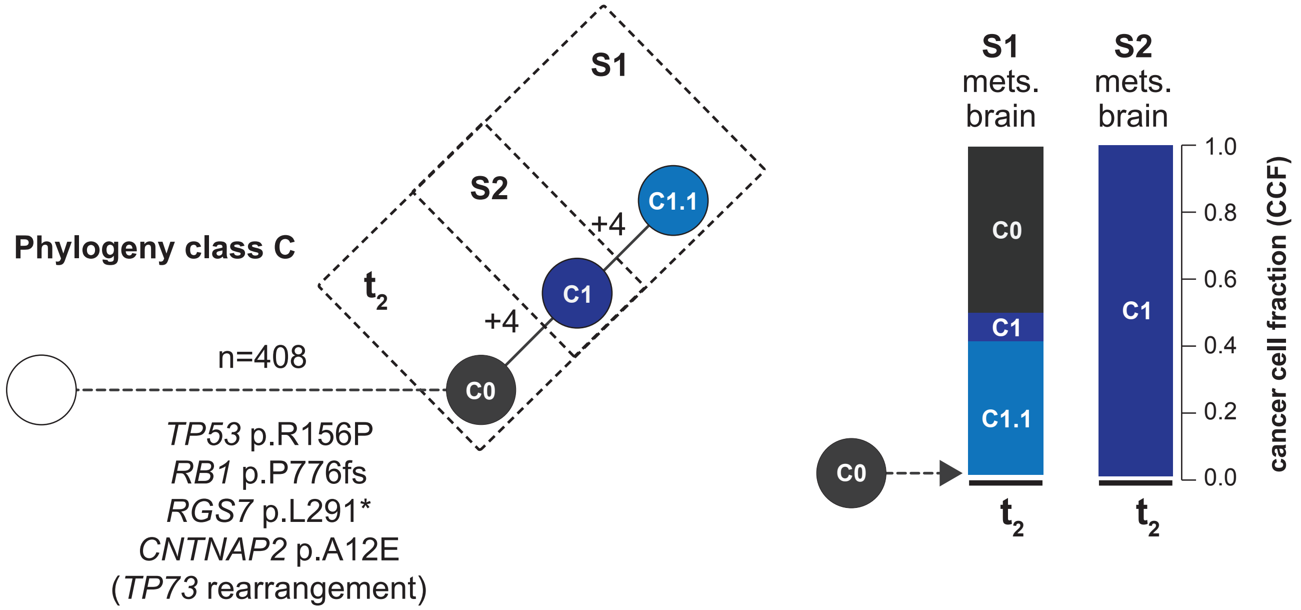

S03213 T2N2M1, stage IV

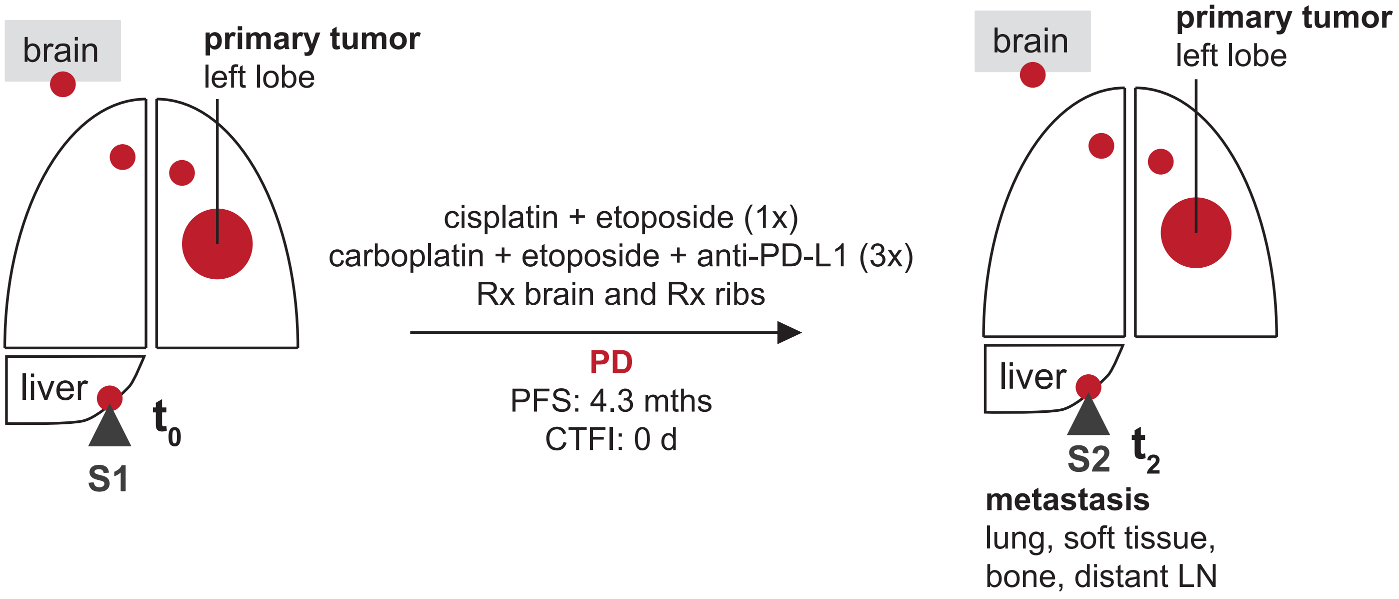

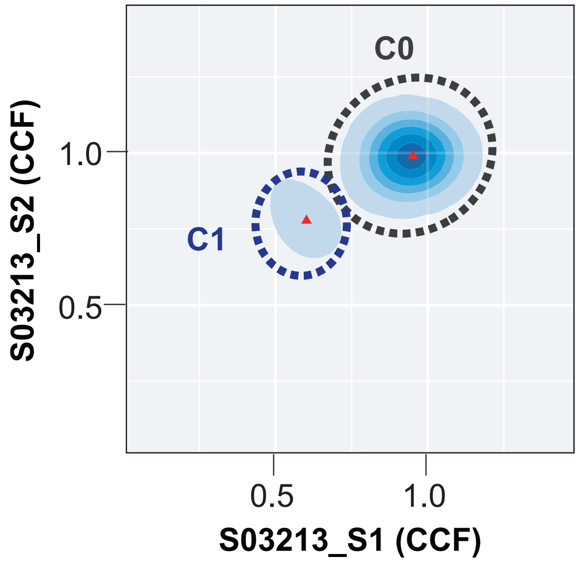

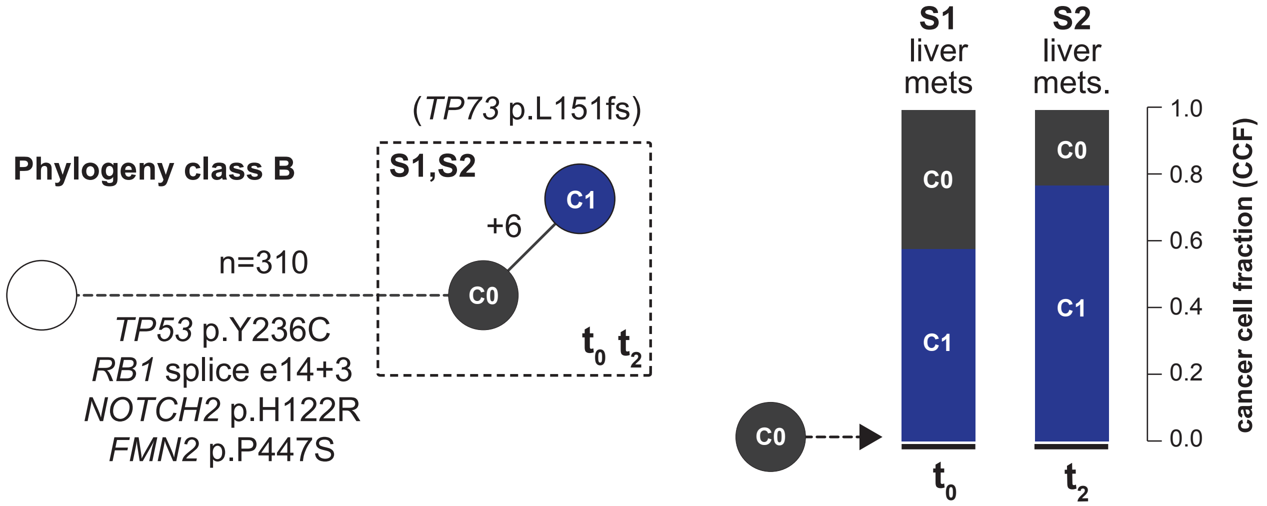

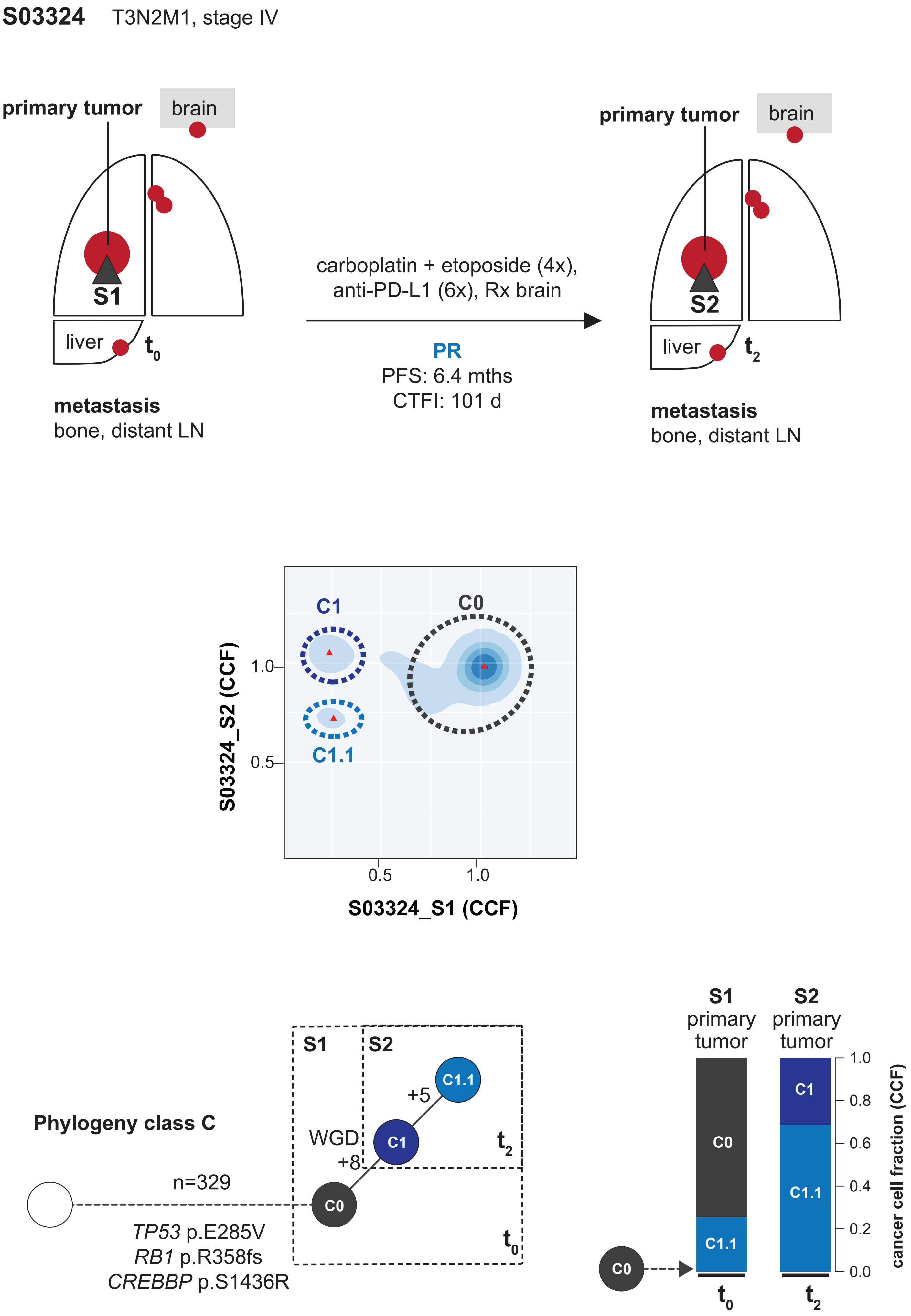

S03324 T3N2M1, stage IV
primary tumor
brain
S1
liver
t0
metastasis
bone, distant LN
carboplatin + etoposide (4x),
anti-PD-L1 (6x), Rx brain
PR
PFS: 6.4 mths
CTFI: 101 d
primary tumor
brain
S2
liver
t2
metastasis
bone, distant LN
C1
C0
C1.1
1.0
0.5
S03324_S2 (CCF)
0.5
1.0
S03324_S1 (CCF)
Phylogeny class C
n=329
TP53 p.E285V
RB1 p.R358fs
CREBBP p.S1436R
C0
WGD
+8
C1
+5
C1.1
S1
S2
t2
t0
S1
primary
tumor
S2
primary
tumor
C0
C1.1
C1
C1.1
C0
t0
t2
1.0
0.8
0.6
0.4
0.2
0.0
cancer cell fraction (CCF)

S03325 T3N2M1, stage IV

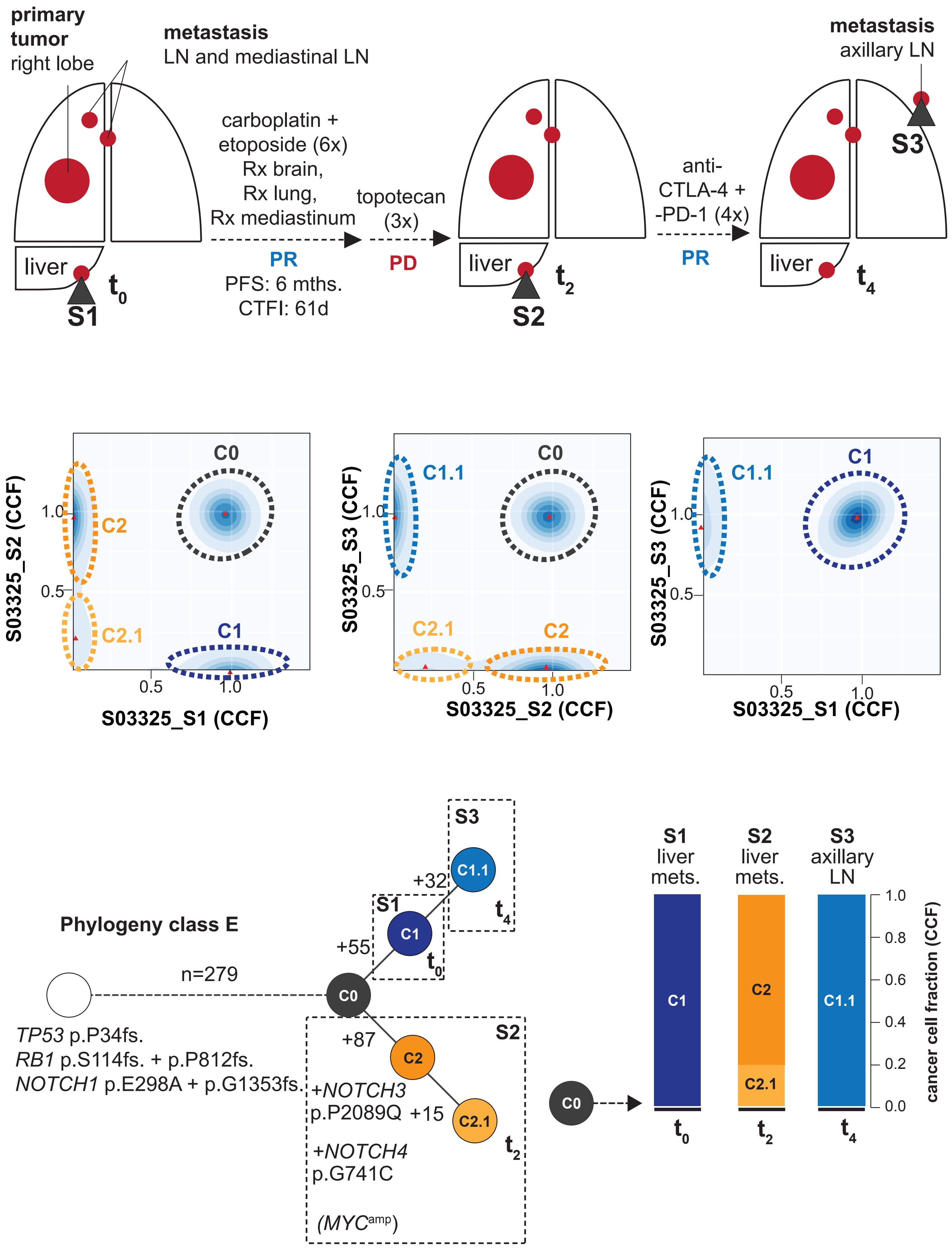

S03405 stage: T3N3M1, stage IV

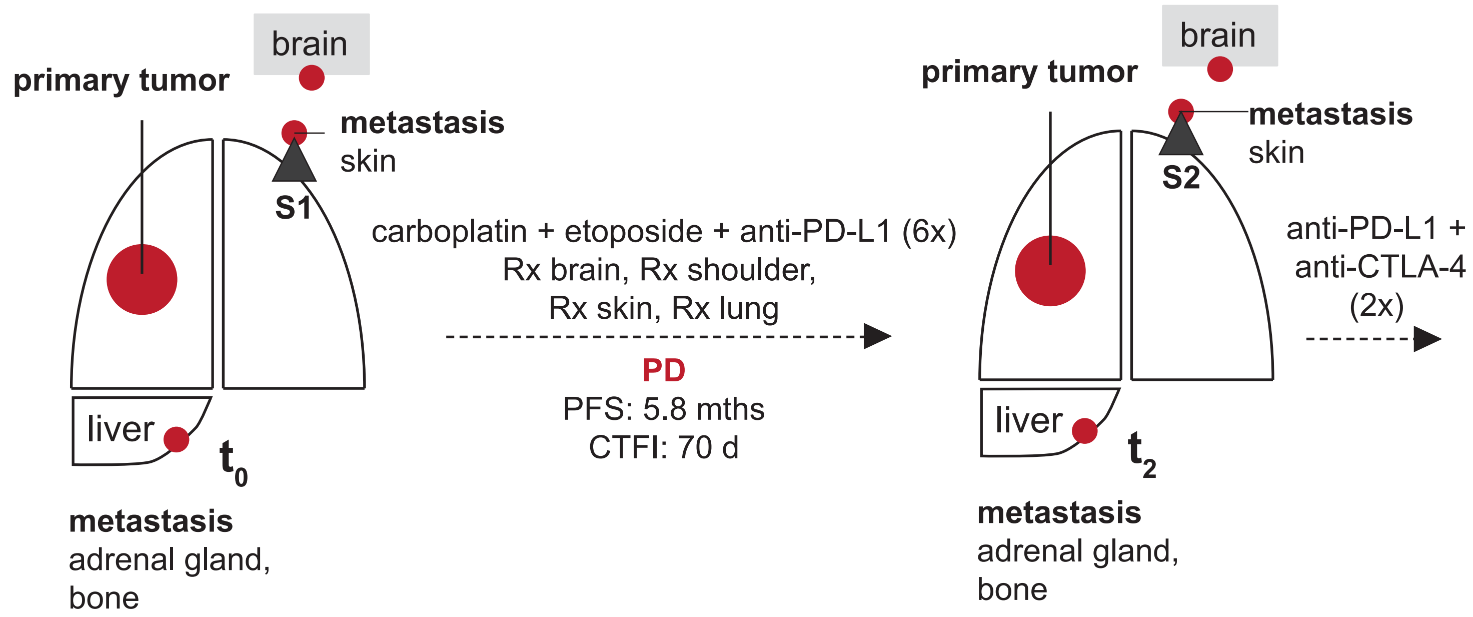

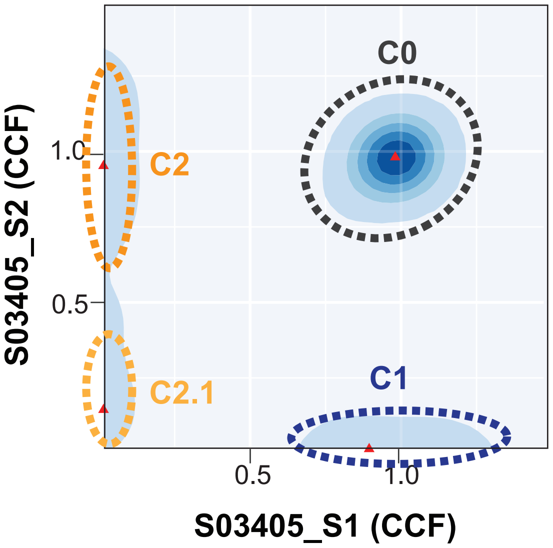

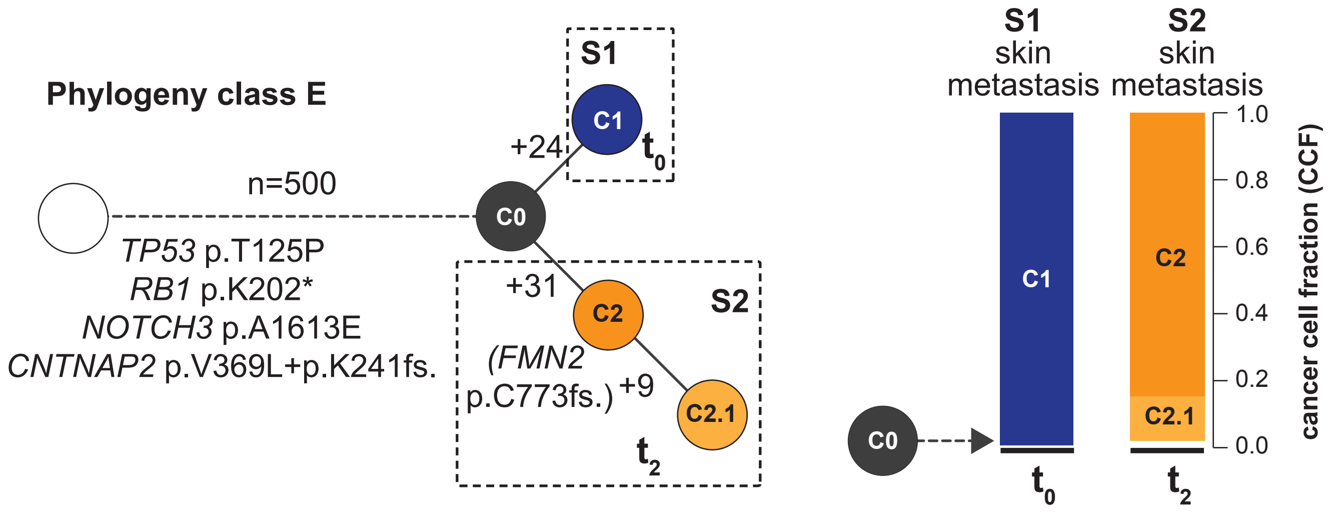

S03727 stage III

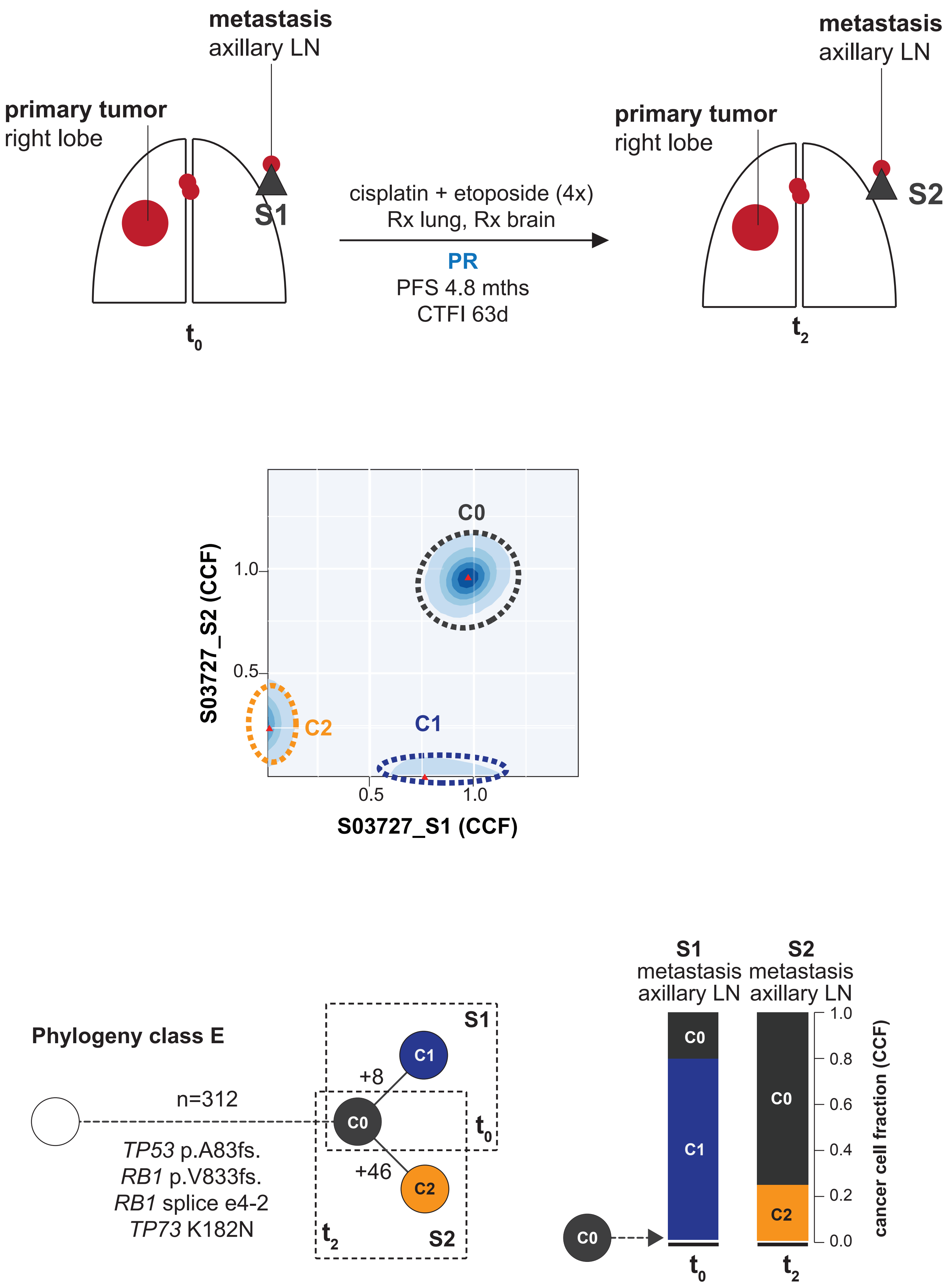

S03744 stage IV

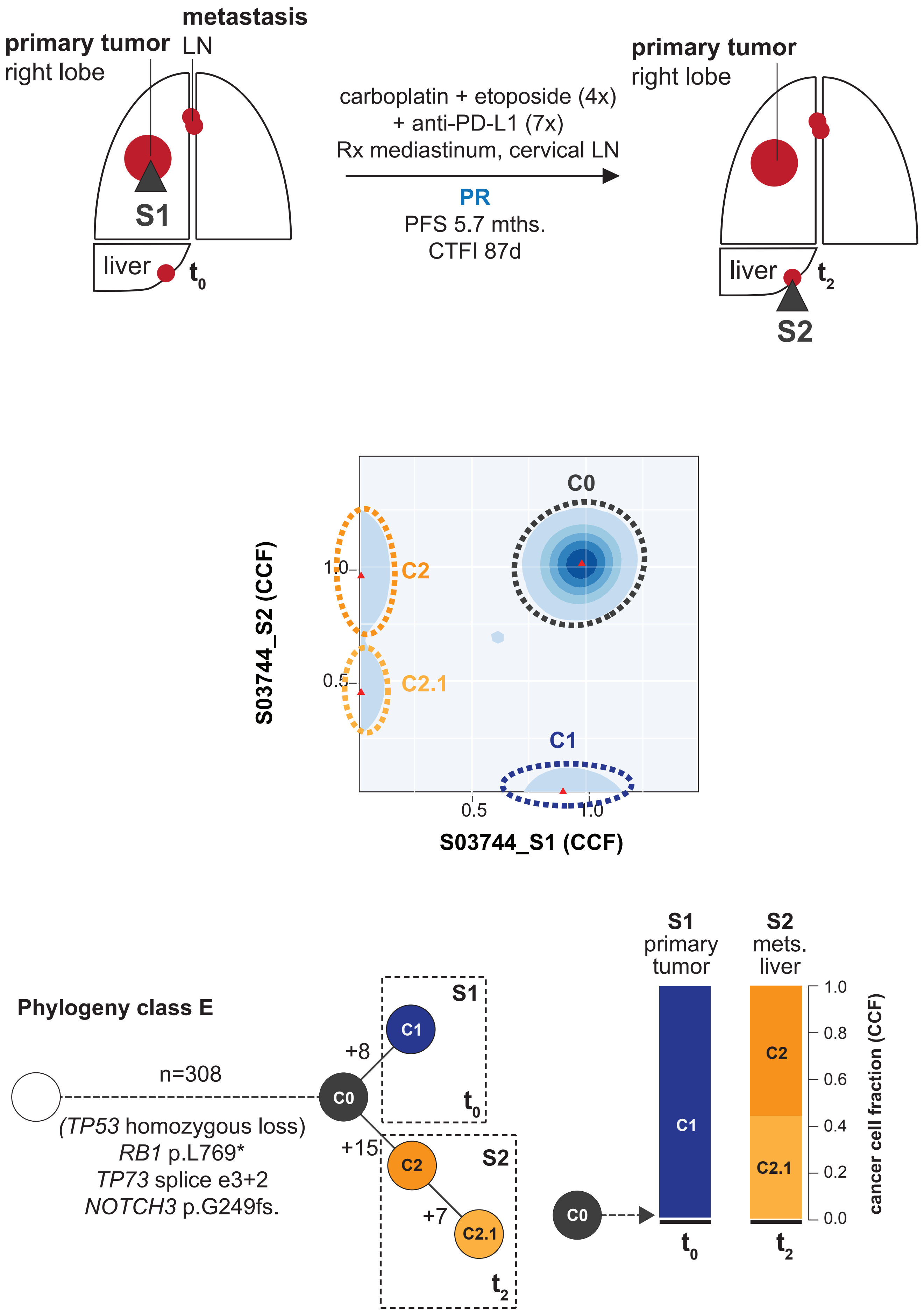

Supplement: Supplementary file 1 — Clinical course and tumour phylogeny determined for 65 patients with SCLC. [file 41586_2024_7177_MOESM1_ESM.pdf]
