# Supplementary material for: Evolutionary trajectories of small cell lung cancer under therapy
Source: Nature. 2024 Mar 13;627(8005):880–9. doi: 10.1038/s41586-024-07177-7 (PMC10972747; doi:10.1038/s41586-024-07177-7)
Supplement: Supplementary file 2 — Reporting Summary [file 41586_2024_7177_MOESM2_ESM.pdf]

nature portfolio

Corresponding author(s): Julie George, Martin Peifer, Roman Thomas

Last updated by author(s): 25th January 2024

Reporting Summary

Nature Portfolio wishes to improve the reproducibility of the work that we publish. This form provides structure for consistency and transparency in reporting. For further information on Nature Portfolio policies, see our Editorial Policies and the Editorial Policy Checklist.

Statistics

For all statistical analyses, confirm that the following items are present in the figure legend, table legend, main text, or Methods section.

n/a	Confirmed	
☐	☒	The exact sample size (n) for each experimental group/condition, given as a discrete number and unit of measurement
☐	☒	A statement on whether measurements were taken from distinct samples or whether the same sample was measured repeatedly
☐	☒	The statistical test(s) used AND whether they are one- or two-sided<br>*Only common tests should be described solely by name; describe more complex techniques in the Methods section.*
☐	☒	A description of all covariates tested
☐	☒	A description of any assumptions or corrections, such as tests of normality and adjustment for multiple comparisons
☐	☒	A full description of the statistical parameters including central tendency (e.g. means) or other basic estimates (e.g. regression coefficient) AND variation (e.g. standard deviation) or associated estimates of uncertainty (e.g. confidence intervals)
☐	☒	For null hypothesis testing, the test statistic (e.g. F, t, r) with confidence intervals, effect sizes, degrees of freedom and P value noted<br>*Give P values as exact values whenever suitable.*
☒	☐	For Bayesian analysis, information on the choice of priors and Markov chain Monte Carlo settings
☒	☐	For hierarchical and complex designs, identification of the appropriate level for tests and full reporting of outcomes
☐	☒	Estimates of effect sizes (e.g. Cohen's d, Pearson's r), indicating how they were calculated

Our web collection on statistics for biologists contains articles on many of the points above.

Software and code

Policy information about availability of computer code

Data collection	Sequencing data was generated as part of this study.
Data analysis	Data was analyzed with available tools described in Methods and with computational approaches published earlier as indicated in the manuscript. We provide our genome data processing workflow are software code for download. Sanger sequencing data was analyzed with Geneious version 8.

For manuscripts utilizing custom algorithms or software that are central to the research but not yet described in published literature, software must be made available to editors and reviewers. We strongly encourage code deposition in a community repository (e.g. GitHub). See the Nature Portfolio guidelines for submitting code & software for further information.

Data

Policy information about availability of data

All manuscripts must include a data availability statement. This statement should provide the following information, where applicable:

- Accession codes, unique identifiers, or web links for publicly available datasets
- A description of any restrictions on data availability
- For clinical datasets or third party data, please ensure that the statement adheres to our policy

Human genome sequencing data was analyzed referring to to the human reference genome (NCBI37/hg19). The raw sequencing data is deposited in the European Genome-Phenome Archive under the accession number: EGAS50000000169. Supporting data is provided as Supplementary Tables.

Research involving human participants, their data, or biological material

Policy information about studies with human participants or human data. See also policy information about sex, gender (identity/presentation), and sexual orientation and race, ethnicity and racism.

Reporting on sex and gender	The patients included in this study were were predominantly male (n=43/65, 66%)
Reporting on race, ethnicity, or other socially relevant groupings	There was no selection of patients based on race, ethnicity, or other socially relevant groupings.
Population characteristics	The median age at the time of first diagnosis was 64 years. Additional information is provided in Methods.
Recruitment	Patients were diagnosed with Small Cell Lung Cancer. No other specific criteria were applied to recruit patients (no clinical study).
Ethics oversight	University of Cologne

Note that full information on the approval of the study protocol must also be provided in the manuscript.

Field-specific reporting

Please select the one below that is the best fit for your research. If you are not sure, read the appropriate sections before making your selection.

☒ Life sciences ☐ Behavioural & social sciences ☐ Ecological, evolutionary & environmental sciences

For a reference copy of the document with all sections, see nature.com/documents/nr-reporting-summary-flat.pdf

Life sciences study design

All studies must disclose on these points even when the disclosure is negative.

Sample size	Study of 65 patients with SCLC focussing on the analysis of multi-regional tumor samples (at least n>=2). Patients were chosen based on the availability of multi-regional tumor material for sequencing purposes. This cohort of 65 patients provided sufficient number of cases for the analyses described (mostly n=5; at least n>3).
Data exclusions	No patient was excluded.
Replication	study cohort of n=65 patients; some findings were confirmed in an independent cohort of n=64 patients. We have performed a power calculation, and which indicated that at least 56 samples are required to validate the findings at a significance level of 5% and a power of 80%
Randomization	Randomization was not applicable in this study, because we describe an exploratory analysis in a discovery cohort.
Blinding	Blinding was not applicable to this study, because we did not perform a clinical study with a specific clinical question, and instead performed an exploratory analysis in a discovery cohort.

Reporting for specific materials, systems and methods

We require information from authors about some types of materials, experimental systems and methods used in many studies. Here, indicate whether each material, system or method listed is relevant to your study. If you are not sure if a list item applies to your research, read the appropriate section before selecting a response.

Materials & experimental systems

n/a	Involved in the study	
☐	☒	Antibodies
☐	☒	Eukaryotic cell lines
☒	☐	Palaeontology and archaeology
☐	☒	Animals and other organisms
☒	☐	Clinical data
☒	☐	Dual use research of concern
☒	☐	Plants

Methods

n/a	Involved in the study	
☒	☐	ChIP-seq
☒	☐	Flow cytometry
☒	☐	MRI-based neuroimaging

Antibodies

Antibodies used	anti-p53 (clone D07, Abcam, catalogue number: #ab80644, 1:1000 dilution), anti-HSP90 (clone C45G5, Cell Signaling, catalogue number: #4877, 1:1000 dilution), IRDye 800CW goat anti-mouse (LI-COR, catalogue number #926-32210, 1:10000 dilution), IRDye 800CW goat anti-rabbit (LI-COR, catalogue number #926-32211, 1:10000 dilution)
Validation	Antibodies are commonly used in routine diagnositics and for control stainings, and have been validated by the manufacturer. For anti-p53: manufacturer guarantess use for IP, WB, IHC, Flow Cyt and ICC/IF); anti-HSP: manufacturer's information - HSP90 (C45G5) Rabbit mAb detects endogenous levels of total HSP90 protein. This antibody does not cross-react with other HSPs.

Eukaryotic cell lines

Policy information about cell lines and Sex and Gender in Research

Cell line source(s)	A549 cell lines (NSCLC)
Authentication	We applied STR profiling to authenticate the cell line.
Mycoplasma contamination	Regular mycoplasma tests were performed and cell were confirmed to be negative for mycoplasma.
Commonly misidentified lines (See ICLAC register)	No commonly misidentified cell line was used in this study.

Animals and other research organisms

Policy information about studies involving animals; ARRIVE guidelines recommended for reporting animal research, and Sex and Gender in Research

Laboratory animals	Immune compromised NSG mice, 7-14 weeks old, All animals were housed in a specific-pathogen-free facility at ambient temperature and maintaining a 12h light/12h dark cycle.
Wild animals	No wild animals were used in this study.
Reporting on sex	Immune compromised NSG mice were used for establishing patient-derived xenotransplant models, male and female mice were used, no specific data has been collected for male and female mice used in this study.
Field-collected samples	No field-collected samples were used in this study.
Ethics oversight	State Agency for Nature, Environment and Consumer Protection (LANUV) of the State of North Rhine-Westphalia

Note that full information on the approval of the study protocol must also be provided in the manuscript.

Plants

Seed stocks	Not applicable
Novel plant genotypes	Not applicable
Authentication	Not applicable
