# Supplementary material for: Evolutionary trajectories of small cell lung cancer under therapy
Source: Nature. 2024 Mar 13;627(8005):880–9. doi: 10.1038/s41586-024-07177-7 (PMC10972747; doi:10.1038/s41586-024-07177-7)
Supplement: Supplementary file 3 — Uncropped immunoblots from Extended Data Fig. 10a. [file 41586_2024_7177_MOESM3_ESM.pdf]

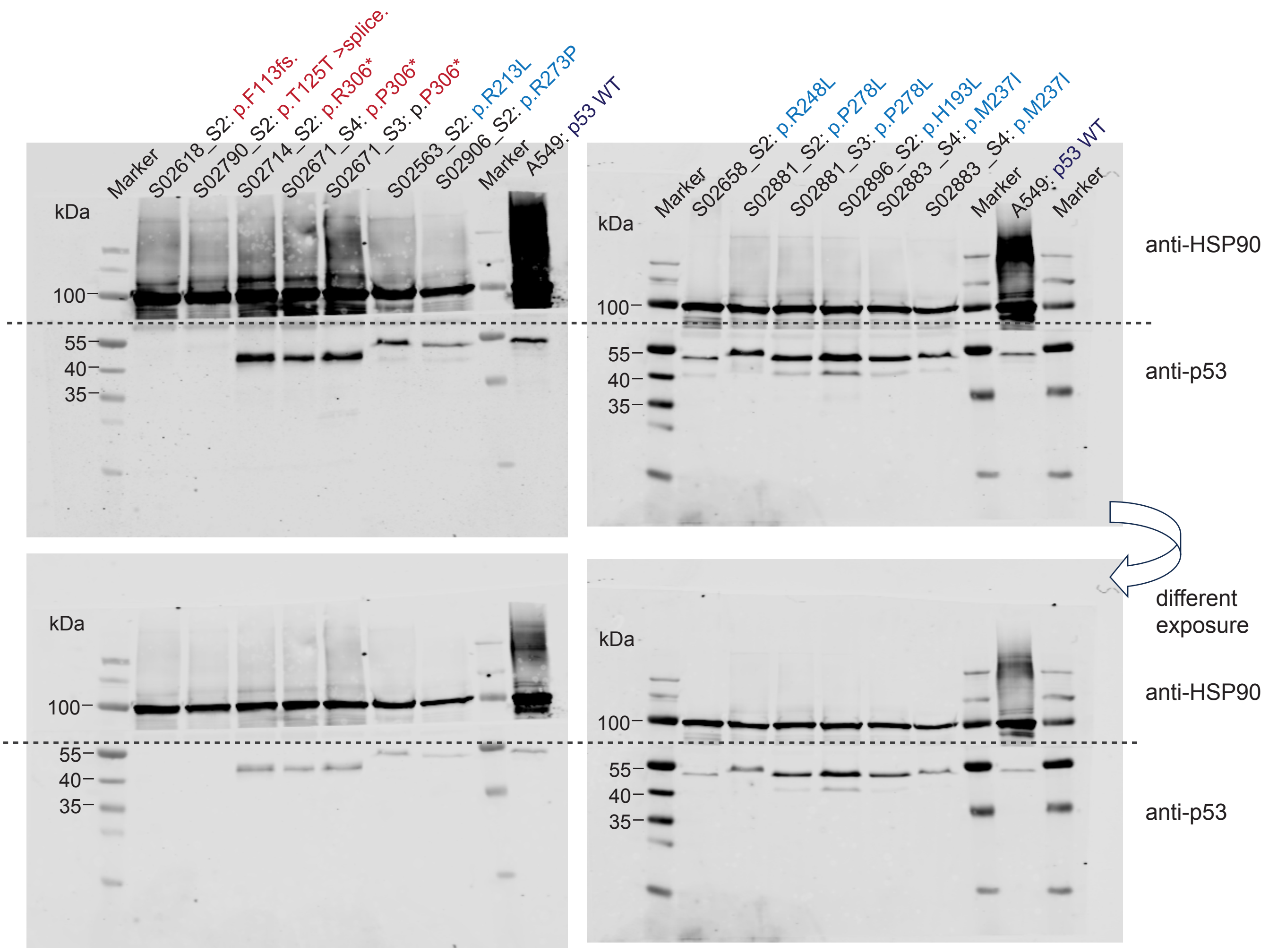

SI Figure 1 | Uncropped immunoblots from Extended Data Figure 9a

p53 protein expression in tumor cell lysates probing with anti-p53 and anti-HSP90 (loading control). *TP53* alterations resulting in point mutations (blue) or other gene damaging alterations (red) are indicated. Lysates of the NSCLC cell line A549 served as a control for wild-type p53 (right lane). Different exposures of both immunoblots are provided.
